# Benzoyl‐Xanthenoxanthenes: Versatile Chromophores for Light‐Engaging Applications

**DOI:** 10.1002/anie.202523349

**Published:** 2026-01-09

**Authors:** Cristian De Luca, El Czar Galleposo, Rúben R. Ferreira, Chiara Puccinelli, Herwig Peterlik, Pradip Kumar Mondal, Laurens van Dam, Johannes C. B. Dietschreit, Yoshimichi Shimomura, Gen‐ichi Konishi, Davide Bonifazi

**Affiliations:** ^1^ Institute of Organic Chemistry, Faculty of Chemistry University of Vienna Vienna 1090 Austria; ^2^ Vienna Doctoral School in Chemistry University of Vienna Währinger Straße 42 Vienna 1090 Austria; ^3^ Faculty Center for Nano Structure Research Faculty of Physics University of Vienna Wien 1090 Austria; ^4^ Elettra Sincrotrone Trieste S.C.p.A. Trieste 34149 Italy; ^5^ Institute of Theoretical Chemistry, Faculty of Chemistry University of Vienna Währinger Straße 17 Vienna 1090 Austria; ^6^ Department of Chemical Science and Engineering Institute of Science Tokyo Tokyo 152‐8552 Japan

**Keywords:** Heteroatom doping, Light‐harvesting system, NIR emitters, Polyaromatic hydrocarbons, π‐Conjugated architectures

## Abstract

In this work, we present a modular donor–acceptor strategy that produces oxidatively stable, benzoyl‐fused *peri*‐xanthenoxanthene (PXX) ribbons with near‐infrared (NIR) emission and light‐harvesting properties. Benzoyl fusion at the *pseudo‐peri* positions, achieved through intramolecular Friedel–Crafts planarization, creates the first benzoyl‐functionalized PXX monomers, dimers, and trimers that exhibit deep red–NIR absorption and emission without significantly raising the HOMO level. The ribbons display strong, tunable absorption and fluorescence bands, with up to 63% of emission in the NIR and an NIR fluorescence quantum yield reaching 0.36 in solution. Lewis adducts formed between boron‐based Lewis acids and carbonyl acceptor sites further enhance the electron‐accepting nature of the ketones, producing pure NIR emission ($\Phi_{F}^{\mathrm{NIR}}$ ≈ 0.15–0.16). Spectroelectrochemical investigations uncover reversible electrochromism and electrofluorochromism, allowing redox‐gated switching and NIR absorption signatures, useful for sensing and display technologies. When embedded in a nematic liquid crystal phase, the ribbons function as cascaded Förster resonance energy transfer (FRET) antennas, achieving near‐quantitative single‐step energy transfer (Φ_FRET_ = 0.97) and highly efficient two‐step transfer (Φ_FRET_ = 0.70), enabling directional energy funneling. Their complementary absorption broadens the excitation window to cover the entire visible spectrum, making them highly efficient panchromatic light‐harvesting materials.

## Graphical abstract

Oxidatively stable benzoyl‐fused *peri*‐xanthenoxanthene ribbons with NIR emission were synthesized. Their good spectral overlap enabled cascaded Förster resonance energy transfer (FRET) antennae in a nematic liquid crystal phase, achieving directional energy transfer. The final light‐harvesting system exhibits a two‐step FRET efficiency of 0.70 and extended visible absorption, providing efficient panchromatic light‐harvesting capability.

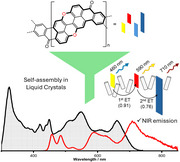

## Introduction

**Figure 1:**
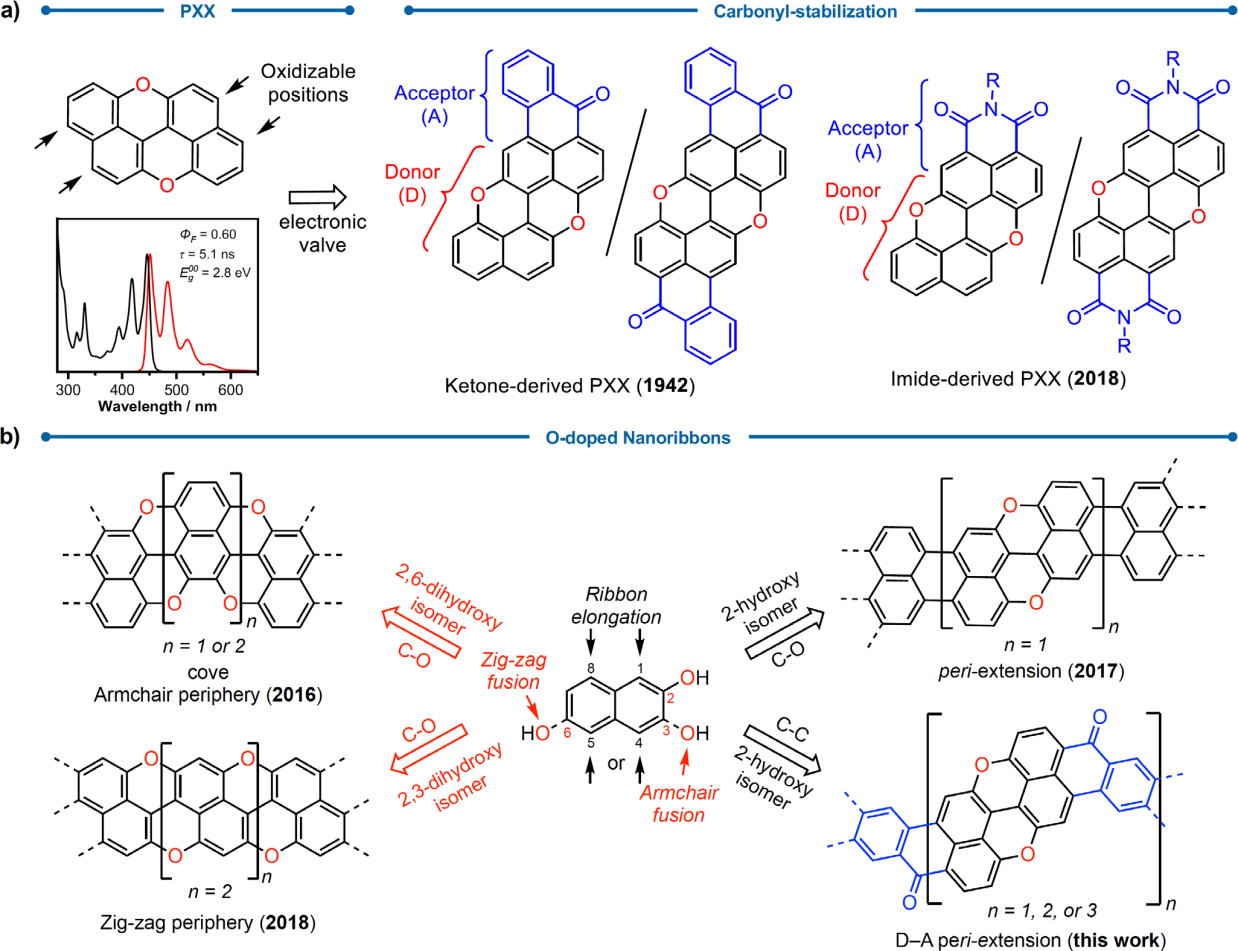
a) Peripheral functionalization of PXX with carbonyl moieties as electron‐withdrawing handles: ketone‐ and imide‐based approach. b) Synthetic strategies for the planarization of PXX‐based ribbons: previous intramolecular C─O bond formation to armchair‐, zig‐zag, and *peri*‐extended architectures, and current Friedel–Crafts‐type C─C bond formation for donor–acceptor molecular ribbons.

Expanding polycyclic aromatic hydrocarbons (PAHs) has emerged as an effective strategy for developing tailored, versatile chromophores. By increasing π‐conjugation length, anisotropic dimensionality, and structural rigidity, extended PAHs such as nanoribbons can support broader light absorption,^[^ ^1^, ^2^, ^3^, ^4^ ^]^ enhanced exciton delocalization and transport,^[^ ^5^, ^6^ ^]^ long‐range coherent exciton dynamics,^[^ ^7^ ^]^ and tunable energy levels^[^ ^8^, ^9^ ^]^— all of which are essential for directional energy flow and spectral overlap in light‐harvesting systems (LHS) applications.^[^ ^10^, ^11^, ^12^, ^13^, ^14^ ^]^ Driven by interest in creating advanced chromophoric platforms, our group has established a research program over the past few years to design and synthesize O‐doped PAHs. Specifically, we have explored the *p*‐type semiconductor *peri*‐xanthenoxanthene (PXX), an O‐containing analogue of anthanthrene.^[^ ^15^, ^16^, ^17^, ^18^, ^19^, ^20^ ^]^ Using mild oxidative synthetic protocols, we have expanded the PXX core and developed various O‐doped analogues, including frameworks featuring armchair and zigzag peripheries. In these systems, we observed that progressively extending the O‐annulated framework narrows the HOMO–LUMO gap, primarily by increasing the HOMO energy level.^[^ ^18^ ^]^ These electron‐rich scaffolds are promising chromophores for LHS, as a systematic narrowing of the HOMO–LUMO gap can promote directional energy transfer (ET) and excitonic coupling, enabling the antenna effect (AE) and multistep energy funneling toward electron‐deficient acceptors.^[^ ^21^ ^]^ However, these extended PXX derivatives display poor oxidative stability; their high HOMO levels make them vulnerable to degradation in the presence of O_2_,^[^ ^18^ ^]^ especially under light exposure, where ^1^O_2_ is generated via self‐photosensitization. This instability poses a significant challenge for their use as semiconductors and in functional LHS. To address this problem, oxidatively stable derivatives need to be designed by strategically replacing C─H bonds at vulnerable sites with electron‐withdrawing groups that act as electron‐accepting valves, thereby reducing the risk of oxidation. Among various approaches, using imide functionalities, as seen in perylene diimide (PDI) derivatives, has proven to be one of the most effective methods for creating functional and chemically durable chromophores. For instance, substituting the *peri* C─H bonds of PXX with imide groups (e.g., PXXMI and PXXDI, Figure 1a) has produced oxidation‐resistant chromophores.^[^ ^16^ ^]^ However, the difficulty of extending these systems into fully π‐conjugated oligomers, along with a significant decrease in the HOMO level upon addition of the imide, led us to explore the benzoyl group as an alternative electron‐withdrawing moiety.

During the design of these monomeric frameworks, we came across an early report by Pummerer,^[^ ^22^ ^]^ who proposed adding two benzoyl groups at the *pseudo*‐*peri* positions of PXX (Figure 1a, ketone‐derived PXX) and described isolating a red derivative through a low‐yielding (unspecified), two‐step C─C bond formation process under harsh conditions. However, no structural data or characterization were provided, and these types of compounds have remained elusive to date. Capitalizing on these considerations, we devised the synthesis of PXX derivatives peripherally doped with benzoyl units at the 3 and 4 positions to construct donor–acceptor (D–A) molecular ribbons (Figure 1). This strategy enables a systematic bathochromic shift in the absorption and emission spectra toward the NIR spectral region through molecular engineering, without significantly increasing the HOMO level, thereby reducing susceptibility to oxidation. Furthermore, we demonstrate the construction of efficient LHS in a nematic liquid‐crystalline (NLC) phase, where molecular dynamics and self‐assembly enhance ET.^[^ ^23^, ^24^, ^25^ ^]^ These features represent key advances toward the development of artificial photosynthetic systems capable of efficient energy harvesting and/or conversion.

## Results and Discussion

### Design and Synthesis

**Scheme 1:**
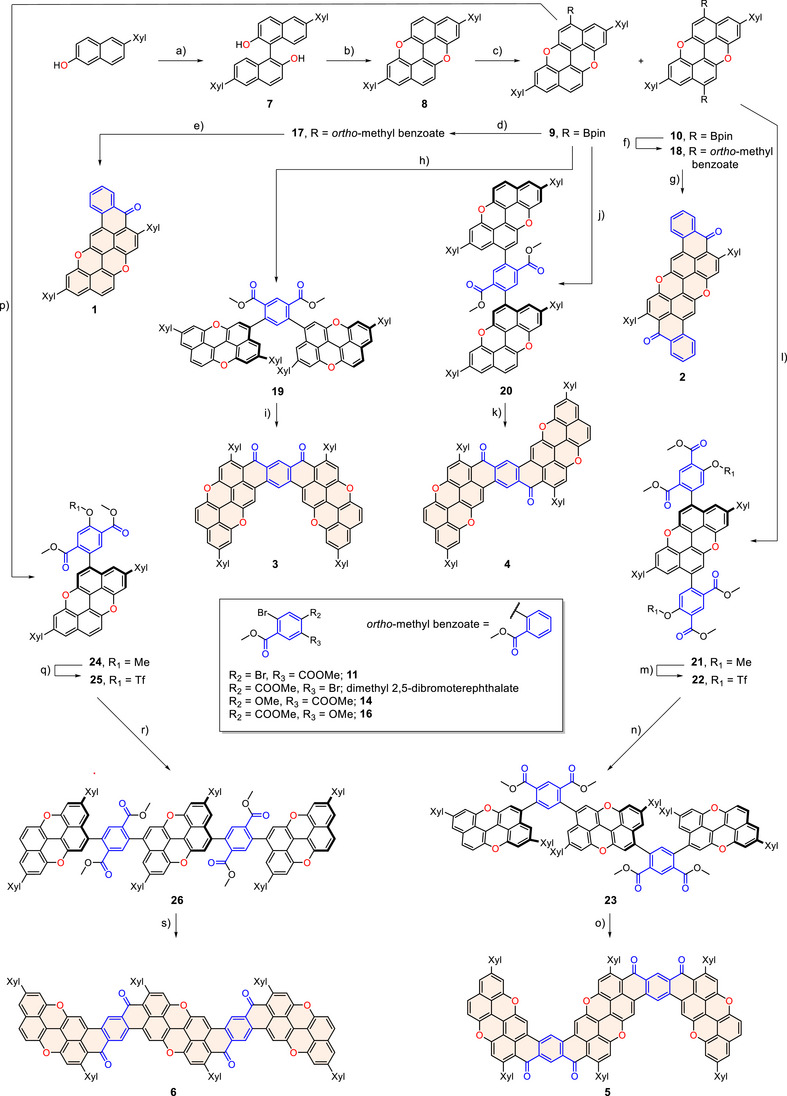
Synthesis of O‐doped ribbons **1**–**6**. Reagents and conditions: a) Cu‐TMEDA, CH_2_Cl_2_, RT, 4 h, *quant*.; b) CuCl, NMI, K_2_CO_3_, xylenes, 120 °C, 20 h, 60%; c) [Ni(cod)_2_], dcype, B_2_pin_2_, TFE, mesitylene, 170 °C, 18 h, **9** 36%, **10** 31%. d) Methyl 2‐bromobenzoate, [Pd(dppf)Cl_2_], Et_3_N, Kolliphor, H_2_O/PhMe, 60 °C, 18 h, 70%; e) [1] DDT, NaOH, NMP, 110 °C, 4 h; [2] ER, 70 °C, 87%; f) see conditions d), 60%; g) see conditions e), 63%; h) see conditions (d), dimethyl 4,6‐dibromoisophthalate, 60%; i) see conditions e), 54%; j) see conditions d), dimethyl 2,5‐dibromoterephthalate, 64%; k) see conditions (e), 66%; l) **14**, K_3_PO_3_, [Pd_2_(dba)_3_], PCy_3_, dioxane/H_2_O, 105 °C, 18 h, 74%; m) [1] AlCl_3_, DDT, CH_2_Cl_2_, RT, 1 h; [2] Et_3_N, CH_2_Cl_2_, Tf_2_O, RT, 2 h, 55%; n) **9**, [Pd(dppf)Cl_2_], CsF, dioxane/H_2_O, 120 °C, 43%; o) see conditions (e), 41%; p) see conditions (d), **16**, 79%; q) see conditions (m), 97%; r) see conditions (n), **10**, 58%; s) see conditions (e), 23%. DDT = 1‐dodecanthiol.

Based on the above considerations, PXX‐based molecular ribbons were designed with benzoyl groups to create D–A‐type molecular tapes (Figure 1). Typically, O‐doped PAHs are produced through Cu‐mediated oxidative C─O bond formation, a planarization route enabling the creation of pyranopyranyl substructures from preorganized hydroxy‐substituted PAH precursors.^[^ ^26^, ^27^, ^28^, ^29^ ^]^ However, in this study, we constructed the molecular tapes from two components: a PXX donor unit and a benzoyl‐based acceptor spacer. This led to the synthesis of benzoyl‐functionalized PXX derivatives, which are covalently linked into an oligomeric precursor and then planarized via an intramolecular Friedel–Crafts acylation (Scheme 1). To improve solubility, which can be limited by extended π‐conjugation, xylyl groups were attached to the PXX cores. The synthesis began with the preparation of key mono‐boryl and bis‐boryl PXX intermediates, **9** and **10**. Specifically, 6‐(2,6‐dimethylphenyl)naphthalen‐2‐ol^[^ ^30^ ^]^ was dimerized in quantitative yield via Cu(II)‐mediated oxidative coupling to form binaphthyl **7**, which was then planarized via C─O bond formation with CuCl, affording PXX derivative **8** in 60% yield. Modification of Kamei's Ni‐catalyzed borylation methodology^[^ ^31^ ^]^ delivered mono‐boryl and bis‐boryl PXX derivatives **9** and **10**, which were obtained in 36% and 31% yield, respectively. The strategies for synthesizing each PXX derivative varied depending on the specific target (Supporting Information, Section 2).

Initially, benzoyl chromophore **1** was prepared through Suzuki–Miyaura cross‐coupling^[^ ^32^ ^]^ of **9** with methyl 2‐bromobenzoate under micellar conditions, producing ester **17**, which was deprotected with 1‐dodecanthiolate (DDT) to form the corresponding carboxylic acid (not isolated, Supporting Information, Section 2). Friedel–Crafts‐type annulation with Eaton's reagent (ER),^[^ ^33^ ^]^ a saturated solution of P_2_O_5_ in MsOH, yielded benzoyl‐derived chromophore **1** in 87% overall yield over two steps.

**Figure 2:**
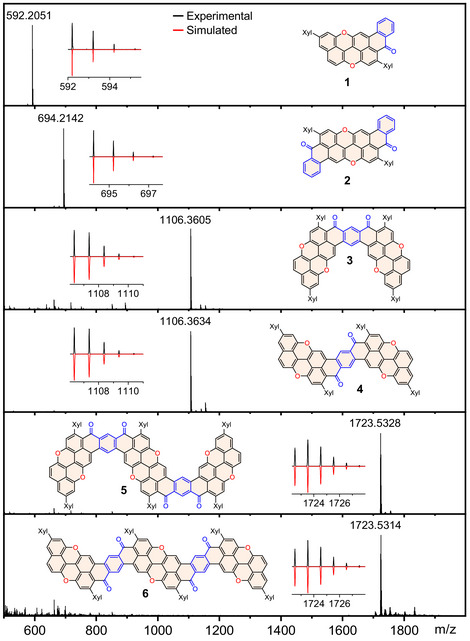
HRMS (MALDI) spectra for O‐doped derivatives **1**–**6** (matrix: DCTB). Inset: experimental (black) and simulated (red) isotopic pattern of the M^+^ peak.

**Figure 3:**
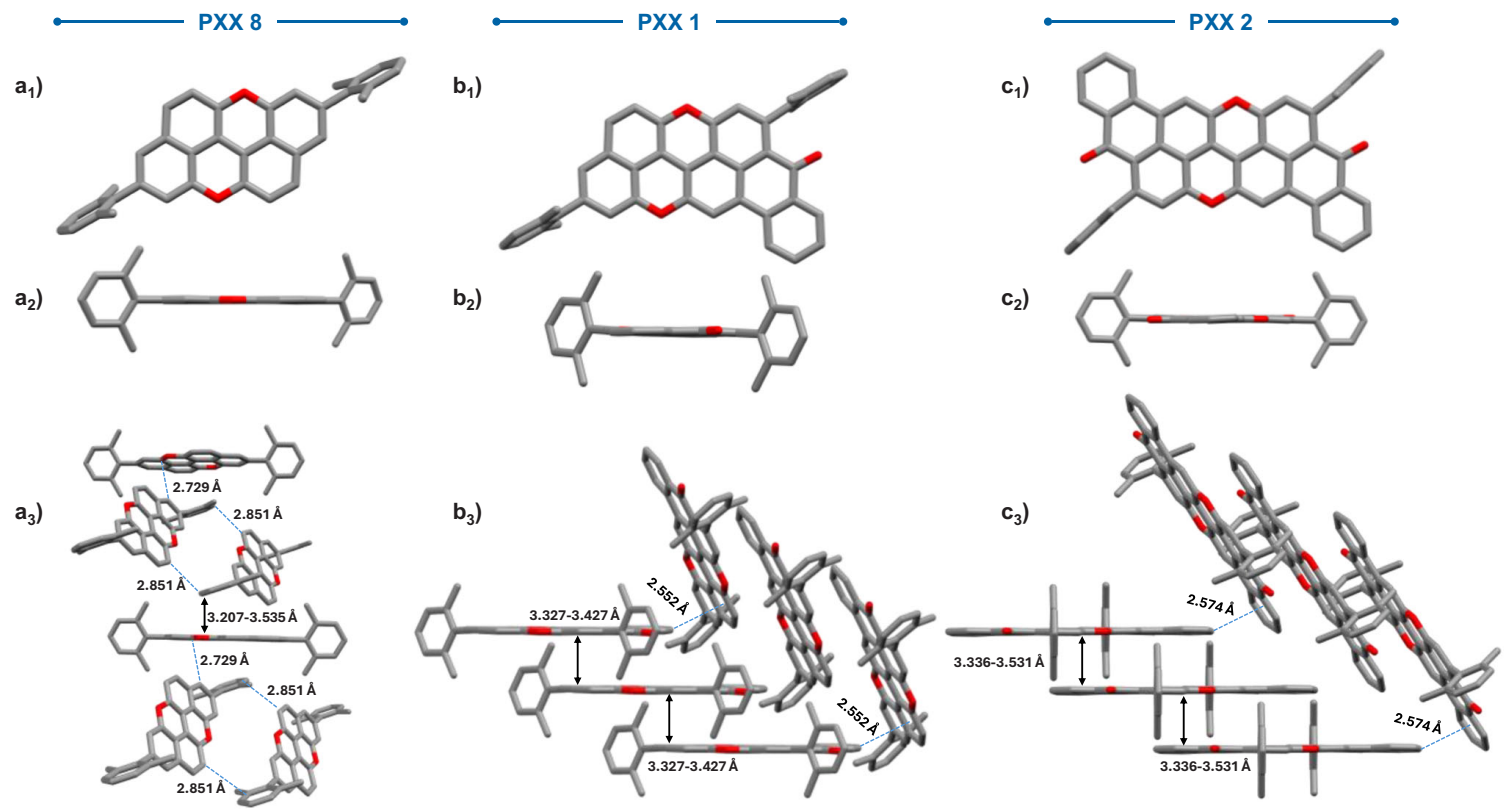
Single‐crystal X‐ray structures for **8**, **1**, and **2**. Space groups: R ‐3 (**8**), C c (**1**), C 2/c (**2**). Crystallization solvents: CH_2_Cl_2_/MeOH for **8** and **2**, and CHCl_3_/MeOH for **1**. ORTEP representation a_1_–c_1_) front view, a_2_–c_2_) side view, and a_3_–c_3_) solid‐state arrangements. Hydrogen atoms and solvent molecules are omitted for clarity. Atom colors: gray, C; red, O.

Similarly, the dibenzoyl PXX derivative **2** was synthesized starting from bis‐boryl PXX **10** and methyl 2‐bromobenzoate, giving diester **18** in 60% yield under classical Suzuki cross‐coupling conditions. DDT‐mediated deprotection and Friedel–Crafts annulation gave chromophore **2** in 63% yield. For compound **3**, dimethyl 4,6‐dibromoisophthalate **11** was prepared via Fischer esterification of 4,6‐dibromoisophthalic acid in 72% yield. Ester **19** was obtained in 80% yield by cross‐coupling reaction between **9** and **11**. Deprotection and Friedel–Crafts annulation of **19** gave *meta*‐dimer **3** in 54% yield. *para*‐Dimer **4** was prepared by reacting **9** with dimethyl 2,5‐dibromoterephthalate, yielding ester **20** (64%). Intermediate **21** was obtained in 74% yield from cross‐coupling between ester **14** (prepared from 1,5‐dibromo‐2,4‐dimethylbenzene in three steps, with an overall yield of 49%, Supporting Information, Section 2)^[^ ^34^ ^]^ and PXX derivative **10**. Selective deprotection with DDT and AlCl_3_, followed by triflation, gave intermediate **22** in 55% yield over 2 steps. Ester **23** was then synthesized in 43% yield by reacting **22** with **9** in the presence of [Pd(dppf)Cl_2_] and CsF. Deprotection and planarization of **23** afforded nanoribbon **5** (41%). Given the low solubility of nanoribbon **6** and its symmetric form, a different synthetic approach was used. Ester **24** was cross‐coupled by reacting **9** and methoxyterephthalate **16** (Supporting Information, Section 2), yielding the product in 47% yield. Deprotection and triflation of **24** yielded **25** in 97% yield. Successively, ester **26** was synthesized via Suzuki cross‐coupling between **22** and **9**, followed by deprotection and planarization, to give molecular tape **6** in 23% yield. Notably, only molecules **1** and **2** were sufficiently soluble for NMR characterization, while compounds **3**–**6** strongly aggregate and exhibit limited solubility in common organic solvents (solubility in CD_2_Cl_2_ <1 mg mL^−1^). As shown in Figure 2, the structure of the O‐doped ribbons could be confirmed by high‐resolution mass spectrometry, with molecular ions detected at *m*/*z* 592.2051 (M^+^, C_43_H_28_O_3_), 694.2142 (M^+^, C_50_H_30_O_4_), 1106.3605 (M^+^, C_80_H_50_O_6_), 1106.3634 (M^+^, C_80_H_50_O_6_), 1723.5328 (M^+^, C_124_H_74_O_10)_, and 1723.514 (M^+^, C_124_H_74_O_10_). To confirm their structures, suitable single crystals for X‐ray diffraction were obtained (Figure 3).^[^ ^35^ ^]^ The X‐ray analysis confirmed the regioselectivity of planarization and the flat structure of the fused backbone. While steric hindrance from PXX **8** due to the xylyl substituent favors an edge‐to‐face herringbone arrangement primarily stabilized by C─H⋯π interactions (2.729–2.851 Å), benzoyl PXX **1** and **2** organize differently, with π⋯π interactions (3.327–3.427 Å and 3.336–3.531 Å) governing their packing. Interestingly, the structural asymmetry of benzoyl‐PXX derivative **1** results in a head‐to‐tail herringbone pattern between columnar stacks, held by an additional C─H⋯π contact (2.552 Å). In contrast, the symmetric dibenzoyl‐PXX derivative **2** exhibits a typical herringbone‐type packing, stabilized by intercolumnar C─H⋯π interactions (2.574 Å).

### Optoelectronic Properties

**Figure 4:**
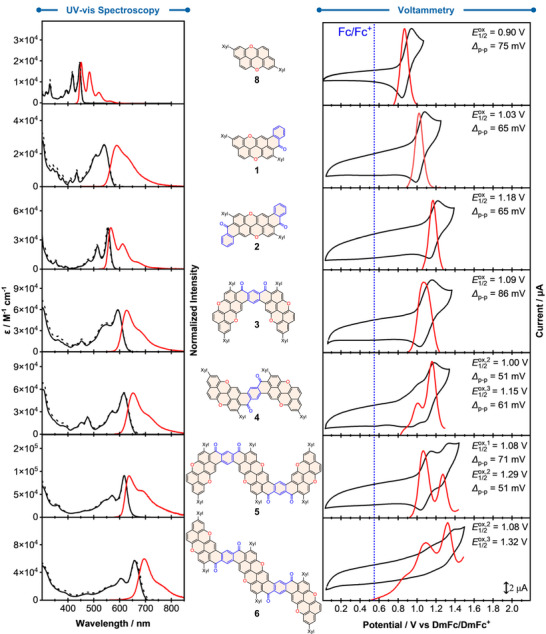
(Left) Absorption (solid black), normalized excitation (dashed black), and normalized fluorescence emission (solid red) spectra of PAHs **1**–**6** and reference **8** in air‐equilibrated CH_2_Cl_2_ at RT. (right) Cyclic (CV, black) and differential pulse (DPV, red) voltammetry of 0.2 mM **1**–**6** and reference **8** in CH_2_Cl_2_ at RT. Working electrode: 7 mm^2^ glassy carbon disk. Counter electrode: Pt wire. Reference electrode: Ag/AgCl. Decamethylferrocene (DmFc) was used as an internal standard. The $E_{1/2}^{\mathrm{ox}}$ of Fc/Fc^+^ couple (blue dashed line) is shown for comparison purposes. The peak‐to‐peak separation (*∆*_p‐p_) of the first oxidation process of **4** could not be resolved due to low peak current intensity; hence, $E_{1/2}^{\mathrm{ox}}$ it was determined using DPV. The reported oxidation potentials of **6** have not been confirmed to be reversible due to solubility issues.

The photophysical properties (Table 1) of derivatives **1**–**6** and reference molecule **8** were studied in CH_2_Cl_2_ (unless specified otherwise) at RT. The UV–vis absorption and emission spectra are reported in Figure 4 (left). First, we examined the effect of the benzoyl fusion. As expected, the absorption maximum of the PXX derivatives bathochromically shifts from 445 (**8**) to 543 and 555 nm upon the fusion of one (**1**) and two (**2**) benzoyl moieties, respectively. Interestingly, the insertion of a second benzoyl moiety seems to only have a limited effect on the optical gap ($E_{g}^{00}$), whereas the molar absorption coefficient value has doubled (25 034 and 44 973 M^−1^ cm^−1^ for molecules **1** and **2**, respectively). Similarly, a bathochromic shift was observed upon π‐extension, with *λ*_max_ shifting from 555 (**2**) to 660 nm (**6**). The *pseudo‐para* derivatives (**4** and **6**) are bathochromically shifted compared to their *pseudo‐meta* isomers (**3** and **5**), with shifts of 23 and 42 nm for the dimeric and trimeric species, respectively. Generally, the steady‐state emission spectra of **1**–**6** show the same trend in bathochromic shift and vibronic substructures as their corresponding absorption spectra.

**Table 1:** Summary of the photophysical properties of O‐doped PAHs **1**–**6** and reference **8** in air‐equilibrated CH_2_Cl_2_ at RT.

	*ε*/M^−1^ cm^−1^	*λ*_max_/nm	*λ*_em_/nm	Φ_F_	$\Phi_{F}^{\mathrm{NIR}}$ a)	*τ*_F_/ns	Φ_∆_	$E_{g}^{00}$/eVb)
**8**	18 324	445	451	0.52	0.00	5.4	0.13	2.76
**1**	25 034	543	589	0.87	0.07	8.6	0.05	2.21
**2**	44 973	555	566	0.66	0.03	3.5	0.20	2.21
**3**	59 635	594	627	0.63	0.12	4.8	0.22	2.03
**4**	54 627	617	650	0.62	0.19	5.4	0.22	1.96
**5**	89 552	618	637	0.31	0.11	2.2	0.34	1.98
**6**	59 289	660	695	0.58	0.36	4.2	0.28	1.83

Molar absorption coefficient (*ε*), absorption maximum wavelength (*λ*_max_), emission maximum wavelength (*λ*_em_), fluorescence quantum yield (Φ_F_), fluorescence quantum yield in the NIR region ($\Phi_{F}^{\mathrm{NIR}}$), fluorescence lifetime (*τ*_F_), singlet oxygen sensitization quantum yield (Φ_∆_), and optical band gap ($E_{g}^{00}$).

^a)^ The fluorescence quantum yield in the NIR region was calculated as follows, $\Phi_{F}^{\mathrm{NIR}}$ $=\frac{A_{\mathrm{NIR}}}{A_{F}}\times \Phi_{F}$, where *A*_NIR_ is the integral of the emission spectrum from 700 nm and *A*_F_ is the integral of the whole emission spectrum.

^b)^ Optical band gap, calculated as $E_{g}^{00}=\frac{1240}{\lambda_{\mathrm{int}}}$, where *λ*_int_ is the wavelength at the intersection of the normalized absorbance and emission spectra.

For all derivatives, large Stokes shifts were observed relative to **8**, ranging from 0.09 to 0.18 eV, indicating non‐negligible vibrational changes or solvent reorganization upon excitation, except for molecules **2** and **5**, which exhibited Stokes shifts of 0.04 and 0.06 eV, respectively. Fluorescence quantum yield (Φ_F_) measurements showed that attaching one benzoyl group, as in derivative **1**, increases the Φ_F_ value from 0.52 (**8**) to 0.87, while adding a second benzoyl group (**2**) reduces it to 0.66. Interestingly, similar Φ_F_ values were observed upon π‐extension (0.63, 0.62, and 0.58 for molecules **3**, **4**, and **6**, respectively), except for trimer **5**, which had the lowest Φ_F_ value (0.31). Consistent with the initial trend, attaching a single benzoyl group increased the fluorescence lifetime (*τ*_F_) from 5.4 ns (**8**) to 8.6 ns (**1**), whereas adding a second benzoyl group decreased the *τ*_F_ value to 3.5 ns (**2**). The *τ*_F_ values of the *pseudo‐meta* derivatives (4.8 and 2.2 ns for **3** and **5**, respectively) were lower than those of their *pseudo‐para* counterparts (5.4 and 4.2 ns for **4** and **6**, respectively). The singlet oxygen (^1^O_2_) sensitization quantum yield (Φ_∆_) of the PAH was also measured, using CHCl_3_ as solvent and C_60_ as reference (Φ_∆_ = 1.00). The ribbons showed Φ_∆_ values ranging from 0.05 (**1**) to 0.34 (**5**).

Since trimer **5** exhibits the highest Φ_∆_, one can hypothesize that in this derivative, the triplet energy level is highly populated, consistent with an increased intersystem crossing rate, resulting in a lower Φ_F_. The $E_{g}^{00}$ values of **1**–**6** were determined from the intersection of absorption and emission spectra.^[^ ^36^ ^]^ The $E_{g}^{00}$ decreases as the π‐conjugated frameworks expand, from **8** to **6**, reaching a minimum of 1.83 eV for the trimeric *pseudo‐para* derivative **6**. Notably, both dimeric and trimeric derivatives showed emission envelopes extending into the NIR region (*λ* ≥ 700 nm, Table 1).^[^ ^37^ ^]^ Among all derivatives, the *pseudo‐para* trimer **6** exhibited 63% of its emission spectral area in the NIR, with an exceptional fluorescence quantum yield in the NIR region ($\Phi_{F}^{\mathrm{NIR}}$)^[^ ^38^ ^]^ of 0.36.

The electrochemical properties of derivatives **1**–**6** and reference **8** were examined using cyclic voltammetry (CV) and differential pulse voltammetry (DPV) in CH_2_Cl_2_ at RT (Figure 4, right and Table 2). Reference **8** showed one reversible oxidation at 0.90 V (versus DmFc/DmFc^+^), consistent with literature data.^[^ ^10^ ^]^ As expected, the benzoyl fusions in **1** and **2** raise the potential of the first oxidation process from 0.90 V (for **8**) to 1.03 and 1.18 V, respectively. Extending the π‐system in the dimeric structures, **3** and **4**, reduces the first reversible oxidation potential to 1.09 and 0.86 V, respectively. Further π‐extension in the trimeric derivatives results in only *pseudo*‐*meta* trimer **5** showing a reversible oxidation, centered at 1.08 V. Overall, these results suggest that the initial oxidation is largely unaffected by π‐extension. Interestingly, the CV of dimer **4** shows additional reversible oxidations at 1.00 and 1.15 V, while trimer **5** exhibits a second reversible oxidation wave at 1.29 V. Due to the limited solubility of **6** in CH_2_Cl_2_, assessing the reversibility of its oxidation events was not possible. Compared to previously synthesized O‐doped PAHs,^[^ ^18^, ^19^ ^]^ these PXX ribbons exhibit higher oxidation potential (upon π‐extension), making them less susceptible to photooxidation in air. Photostability experiments of representative PXX ribbons (e.g., **8**, **1**, **2**, and **4**) revealed no detectable degradation after continuous irradiation for 100 min (Figure S112). No reversible reductions were observed within the electrochemical window of the solvent. The energies of frontier molecular orbitals were estimated through photophysical and electrochemical measurements (Table 2).^[^ ^33^ ^]^ The HOMO levels range from −5.08 to −5.43 eV, and the LUMO levels range from −3.35 to −2.38 eV. Benzoyl fusion causes both HOMO and LUMO levels to decrease progressively. The π‐extension in the dimeric derivatives increases the HOMO energy by 0.09 eV for **3** and 0.32 eV for **4**, while decreasing the LUMO by 0.09 eV for **3** and increasing it by 0.07 eV for **4**.

**Table 2:** Summary of oxidation potentials (versus DmFc/DmFc^+^) of **1**–**6** and reference **8** determined by cyclic (CV) and differential pulse (DPV) voltammetry and estimated frontier molecular orbital energies.

	$E_{1/2}^{\mathrm{ox},1}$/V		$E_{1/2}^{\mathrm{ox},2}$/V		$E_{1/2}^{\mathrm{ox},3}$/V		Energies/eV^a)^	
	CV	DPV	CV	DPV	CV	DPV	HOMO	LUMO
**8**	0.90	0.87	–	–	–	–	−5.15	−2.38
**1**	1.03	1.02	–	–	–	–	−5.28	−3.07
**2**	1.18	1.18	–	–	–	–	−5.43	−3.22
**3**	1.09	1.09	–	–	–	–	−5.34	−3.31
**4**	^b)^	0.86	1.00	0.99	1.15	1.15	−5.11	−3.15
**5**	1.08	1.08	1.29	1.27	–	–	−5.33	−3.35
**6**	^b)^	0.86	^b)^	1.08	^b)^	1.32	−5.08	−3.25

^a)^ Calculated using the formula *E*_HOMO_ = −($E_{1/2}^{\mathrm{ox}1}$ − 0.55) − 4.8 and *E*_LUMO_ = *E*_HOMO_ + $E_{g}^{00}$.^[^ ^39^ ^]^

^b)^ The peak‐to‐peak separation (*∆*_p‐p_) of the oxidation process could not be resolved due to low peak current intensity, hence $E_{1/2}^{\mathrm{ox}}$ was determined using DPV. Beyond the reported oxidation potentials, no reversible oxidation processes were observed (−).

**Figure 5:**
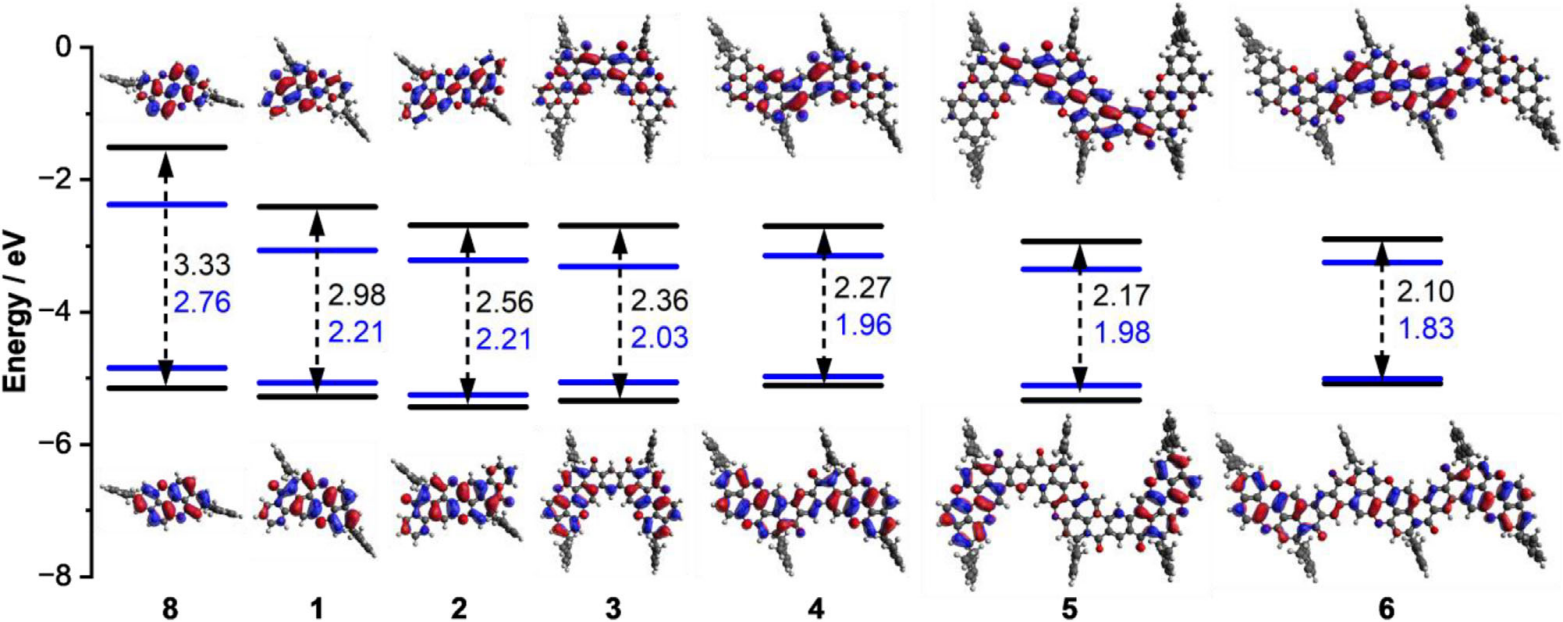
Calculated (black) and measured (blue) frontier orbital energy levels for **1–6** and reference **8** together with their HOMO and LUMO profiles at B3LYP/6–31G(d) level of theory (Gaussian16); HOMO energy was calculated using the formula, *E*_HOMO_ = −($E_{1/2}^{\mathrm{ox}1}$ − 0.55) − 4.8; LUMO energy was calculated using the optical bandgap: *E*_LUMO_ = *E*_HOMO_ + $E_{g}^{00}$.

Interestingly, going from the *pseudo‐meta* isomer (**3**) to the *pseudo‐para* isomer (**4**) resulted in the increase of the HOMO level and the concurrent decrease of the LUMO level, following the increase in π‐conjugation (*para* > *meta*). No significant changes were observed upon further π‐extension to the trimeric derivatives. To further evaluate the structure–property relationship, the HOMO and LUMO orbitals for all O‐doped PAHs were also calculated and compared with those of reference **8** (Figure 5). Derivatives **8** and **2** exhibited frontier molecular orbitals homogeneously distributed over the π‐surface, whereas for the others, the HOMOs are mainly located at the extremities, while the LUMOs lie on the central core of the structures. Benzoyl‐PXX **1** shows an electronic distribution typical of a D–A system, namely, HOMO localized on the more electron‐rich (donor) and LUMO on the more electron‐poor (acceptor) side of the ribbon. Calculated frontier orbital energy levels align with the experimental results, although it is worth noting a general overestimation of the bandgap, primarily due to the overestimation (ca. 0.2 eV) of the LUMO energy levels. The HOMO−1 and LUMO+1 energy levels were also calculated for derivatives **1**–**6** (Supporting Information, Section 6), showing a pronounced localization of the HOMO−1 on the extremities for O‐doped PAHs **1**, **2**, **5**, and **6**, and almost no difference for dimers **3** and **4**.

### Pure NIR Emission via Lewis Acid‐Adduct Formation

**Figure 6:**
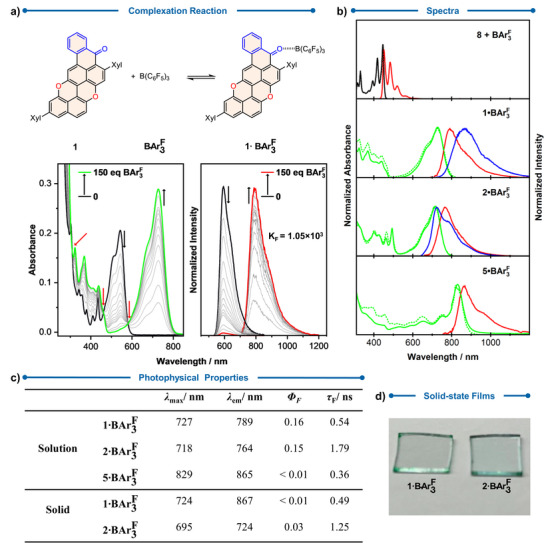
a) Complexation of **1** and ${\mathrm{BAr}}_{3}^{F}$ and the absorption (left) and normalized emission (right) of the titration of **1** (9.6 × 10^−6^ M) with an excess of ${\mathrm{BAr}}_{3}^{F}$ (up to 150 equiv). b) Absorption (solid black for **1** and green for **1·** ${\mathrm{BAr}}_{3}^{F}$, **2·** ${\mathrm{BAr}}_{3}^{F}$, and **5·** ${\mathrm{BAr}}_{3}^{F}$), normalized excitation (dashed black for **1** and green for **1·** ${\mathrm{BAr}}_{3}^{F}$, **2·** ${\mathrm{BAr}}_{3}^{F}$, and **5·** ${\mathrm{BAr}}_{3}^{F}$), and normalized fluorescence emission (solid red) spectra of the complexes in air equilibrated CH_2_Cl_2_ and emission spectrum (solid blue for **1·** ${\mathrm{BAr}}_{3}^{F}$ and **2·BAr_3_^F^**) of the complexes in the solid state at RT. The spectra of **8** (1.2 × 10^−5^ M) with an excess of ${\mathrm{BAr}}_{3}^{F}$ (up to 150 equiv) are shown as a reference. c) Summary of the photophysical properties of the complexes in air equilibrated CH_2_Cl_2_ and in the solid state at RT. d) Pictures of the spin‐coated **1·** ${\mathrm{BAr}}_{3}^{F}$ and **2·** ${\mathrm{BAr}}_{3}^{F}$. Red arrows = isosbestic points.

To achieve pure NIR emission (i.e., limited visible component), we envisaged strengthening the electron‐accepting nature of the carbonyl groups via complexation with a common Lewis acid, such as tris(pentafluorophenyl)borane (${\mathrm{BAr}}_{3}^{F}$).^[^ ^40^, ^41^ ^]^ Upon addition of an excess of ${\mathrm{BAr}}_{3}^{F}$, all benzoyl‐derived PXX derivatives **1**–**6** formed Lewis‐type complexes in solution through the C═O···B(C_6_F_5_)_3_ interactions, as evidenced by the appearance of NIR‐centered electronic transitions (Figure 6a and Supporting Information, Section 3). Control experiments showed no differences in the absorption spectra when ${\mathrm{BAr}}_{3}^{F}$ was added to a solution of **8** (Figure 6b), suggesting that no interaction is established with the pyranopyranyl O‐atoms. Since complexes $1\cdot {\mathrm{BAr}}_{3}^{F}$, $2\cdot {\mathrm{BAr}}_{3}^{F}$, and $5\cdot {\mathrm{BAr}}_{3}^{F}$ exhibited fluorescence (Figure 6b), we limited our discussion to these systems. Exceptionally, complexes $1\cdot {\mathrm{BAr}}_{3}^{F}$ (*K*_a_ = 1.05 × 10^3^ M^−1^ in CH_2_Cl_2_) and $2\cdot {\mathrm{BAr}}_{3}^{F}$ (*K*_F_ is not reported since the $2:{\mathrm{BAr}}_{3}^{F}$ ratio for this complex is uncertain) exhibited emissive envelopes entirely in the NIR region, with $\Phi_{F}^{\mathrm{NIR}}$ values of 0.16 and 0.15, respectively (Figure 6c). On the other hand, $5\cdot {\mathrm{BAr}}_{3}^{F}$ exhibited extremely weak emission, which made the measurement of the $\Phi_{F}^{\mathrm{NIR}}$ value not possible. The solid‐state emission of $1\cdot {\mathrm{BAr}}_{3}^{F}$ and $2\cdot {\mathrm{BAr}}_{3}^{F}$ in spin‐coated films on glass was also characterized (Figure 6d). Interestingly, thin films of $1\cdot {\mathrm{BAr}}_{3}^{F}$ and $2\cdot {\mathrm{BAr}}_{3}^{F}$ exhibited bathochromic and hypsochromic shifts in emission relative to their solution spectra, respectively. These shifts are consistent with distinct aggregation behaviors: adduct $1\cdot {\mathrm{BAr}}_{3}^{F}$ forms J‐aggregates, whereas $2\cdot {\mathrm{BAr}}_{3}^{F}$ forms H‐aggregates.^[^ ^42^ ^]^

**Figure 7:**
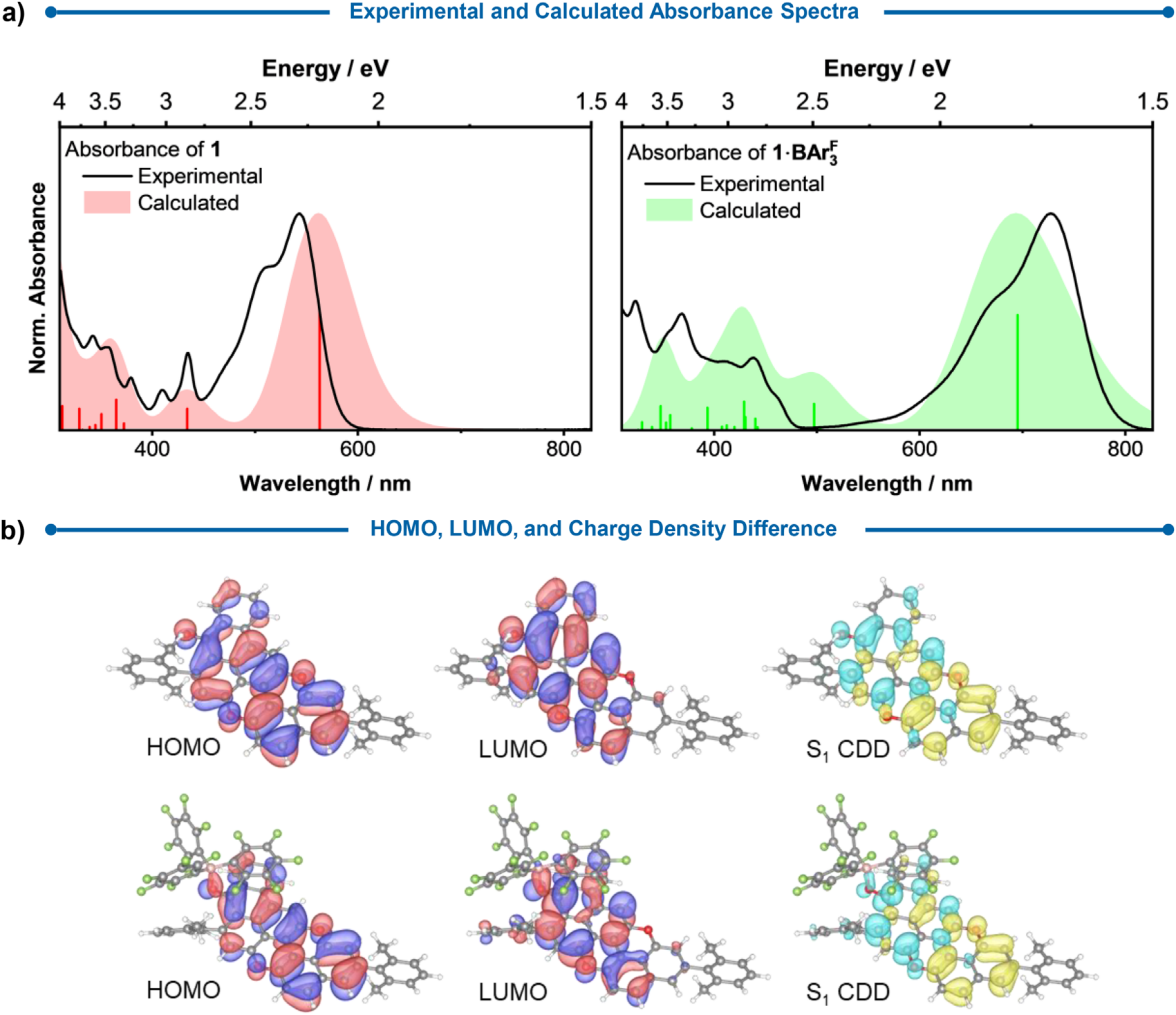
a) Comparison between the experimental absorption spectra and the computed spectra obtained at the B3LYP/6‐311G(d) level of theory with PCM(CH_2_Cl_2_) implicit solvation for compound **1** and its Lewis adduct $1\cdot {\mathrm{BAr}}_{3}^{F}$. b) HOMO, LUMO, and charge density difference (CDD) of the S_1_ excited state for **1** (top) and $1\cdot {\mathrm{BAr}}_{3}^{F}$ (bottom). Isovalues are set to 0.02 for the frontier orbitals and 0.001 for the CDD. Light blue indicates a gain in electron density, and yellow a decrease.

To gain deeper insight into the electronic transitions of the complexes, time‐dependent density functional theory (TD‐DFT) calculations were carried out for complex $1\cdot {\mathrm{BAr}}_{3}^{F}$ and $2\cdot {\mathrm{BAr}}_{3}^{F}$ (Figure 7 and Supporting Information, Section 6). For PXX **1**, the lowest‐energy absorption band originates from the S_1_ excitation, which corresponds to a 98.8% HOMO→LUMO transition with an excitation energy of 2.20 eV. In the case of complex $1\cdot {\mathrm{BAr}}_{3}^{F}$, the analogous S_1_ excitation consists of a 99.2% HOMO→LUMO transition with a significantly reduced excitation energy of 1.78 eV. The HOMO–LUMO gaps are consistently larger than the actual excitation energies. Orbital contributions for all computed transitions can be found in Section 6 of the Supporting Information. In both systems, the S_1_ states are bright π→π* excitations with a strong charge‐transfer (CT) character. The charge‐density difference plots illustrate the displacement of electron density from the opposite side of the molecule toward the ketone functional group upon excitation (Figure 7b). In the complex, the S_1_ transition includes a contribution that donates to the C═O···B(C_6_F_5_)_3_ bond, transferring electron density from the PXX‐moiety to ${\mathrm{BAr}}_{3}^{F}$, as shown in the charge density difference plot (Figure 7b). The LUMO is further stabilized by the electron‐withdrawing nature of the boron center, which facilitates charge transfer to the ketone, thereby lowering the excitation energy. The calculated absorption spectra reproduce the overall shape of the experimental spectra well, although vibrational fine structure is not captured, as the spectra were obtained from single‐point TD‐DFT calculations. The main absorption feature, originating from the S_1_ excitation, is shifted to lower excitation energies in the complex, reflecting the stabilization of the excited state upon complex formation. Some discrepancies persist for higher excitations, such as S_3_, which is responsible for the second lowest energy absorption band in the calculated spectrum of $1\cdot {\mathrm{BAr}}_{3}^{F}$ and appears shifted toward lower energies compared to experiment. This deviation might be caused by the incorrect long‐range behavior of the approximate exchange‐correlation functional, which tends to overstabilize CT states.^[^ ^43^ ^]^ The S_3_ excitation indeed shows a pronounced, more separated CT character, consisting of a 97.0% HOMO−2→LUMO transition, with the HOMO−2 mainly localized on one of the *m*‐xylyl rings. Alternatively, or in combination, the deviation may simply reflect the intrinsic error of TD‐DFT with the B3LYP functional, as the shift lies well within its typical accuracy range for excitation energies.^[^ ^44^ ^]^ Our experiments do not reveal spectroscopically distinct features for the 1:1 and 1:2 complexes of **2** ($2\cdot {\mathrm{BAr}}_{3}^{F}$ and $2\cdot {\left({\mathrm{BAr}}_{3}^{F}\right)}_{2}$), suggesting either a single species or two species with very similar absorption properties. Computational results indicate negligible differences in electronic transitions between the two potential complexes, supporting the difficulty of distinguishing them experimentally (Supporting Information, Section 6, Figure S205, Table S1 and S2). The observed isosbestic points in the complexation of **2** with ${\mathrm{BAr}}_{3}^{F}$ imply either a single dominant complex or two complexes with similar association constants, suggesting independent behavior of the carbonyl binding sites (Figure S102). Given that the NIR absorption and emission properties for both complexes are indistinguishable, the exact binding stoichiometry does not significantly impact our conclusions.

### Electrochromic and Electrofluorochromic Properties

**Figure 8:**
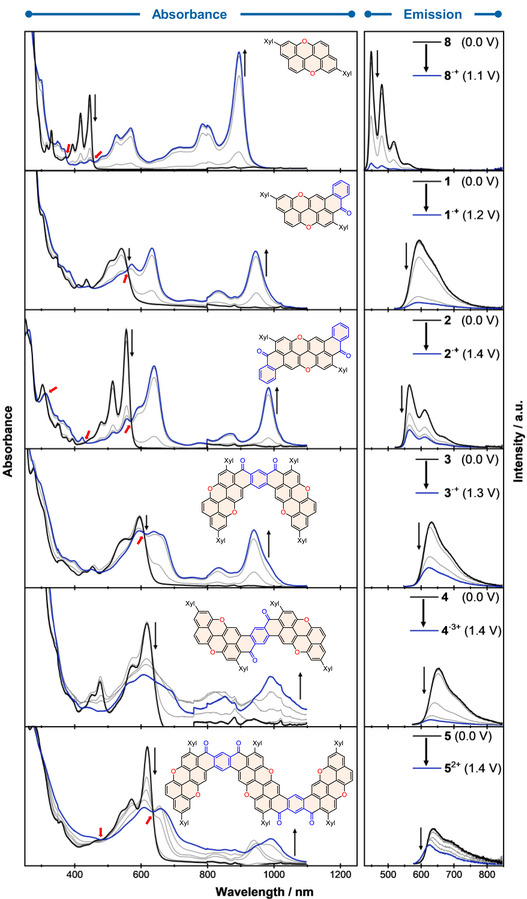
Electrochromism (left) and electrofluorochromism (right) of **1**–**5** and reference **8** in CH_2_Cl_2_ at RT. Working electrode: Pt grid. Counter electrode: Pt wire. Reference electrode: Ag/AgCl. Beyond 1100 nm, there is an artifact in the absorbance profile due to the Pt grid.

We studied the spectroelectrochemical properties of the PXX derivatives with confirmed reversible oxidation processes, **1**–**5**, and reference **8** to gain direct insight into the electronic changes caused by oxidation (Figure 8). All molecules showed a clear bleaching of their characteristic π–π* absorption bands upon oxidation, along with the appearance of broad, low‐energy transitions extending from the visible spectrum into the NIR region (*λ*_max _= 893, 947, 985, 942, 992, and 994 nm for compounds **8**, **1**, **2**, **3**, **4**, and **5**, respectively).

These features indicate the formation of delocalized radical cations, with their intensity and redshift increasing steadily with increasing molecular size and greater conjugation, reaching the full NIR region for the lowest‐energy transitions of the largest structures (**4** and **5**). Notably, *meta*‐dimer **4** did not show clear absorption peaks during the first and second oxidations. Since the first and second oxidation potentials of **4** are within 0.1 V, it is difficult to apply enough overpotential to form only one oxidized species; hence, the observed spectral features are a combination of both species. On the other hand, *meta*‐trimer **5** exhibited the distinct formation of the first oxidized species with an absorption maximum at 934 nm. Moreover, the observation of distinct isosbestic points for several compounds (e.g., **1**–**3**, **5**) suggests a direct, well‐defined conversion between neutral and oxidized states. At the same time, the strong fluorescence seen in the neutral forms is drastically reduced upon oxidation, often nearly fully quenched. The absence of fluorescence emission for the oxidized species indicates that nonradiative decay channels dominate in the radical‐ion states. Through oxidation, it is possible to switch from bright to non‐emissive states (reversibly), with a clear redox‐driven redistribution of the lowest‐energy transition into the NIR. This stark contrast between the two states highlights the potential of these systems for redox‐gated absorbance and fluorescence switching, electro(fluoro)chromic displays, and optical sensors, where reversible absorbance and emission modulation can be directly used for device applications.

### Engineering Artificial Antennae in Liquid Crystals

**Scheme 2:**
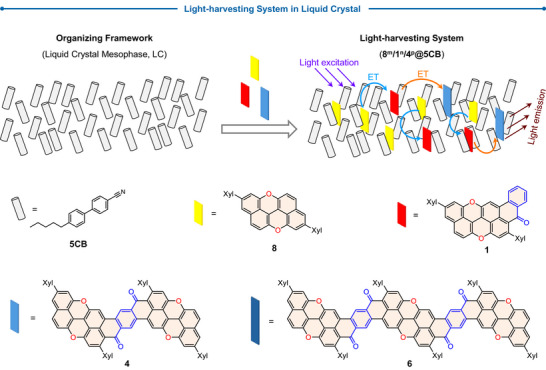
Schematic representation of the mesogenic light‐harvesting system proposed in this work.

Having established the tunability and oxidative stability of our O‐doped PAH scaffolds, we next explored their integration into artificial light‐harvesting systems. Supramolecular assemblies have typically relied on conventional dyes assembled within dendrimers, supramolecular polymers, nanoparticles, or biomolecular templates,^[^ ^45^, ^46^, ^47^, ^48^, ^49^, ^50^, ^51^, ^52^, ^53^, ^54^, ^55^, ^56^, ^57^, ^58^ ^]^ yet their performance remains constrained by limited dye diversity and insufficient long‐range order, often resulting in only moderate Förster resonance energy transfer (FRET) efficiencies (Φ_FRET_).^[^ ^59^, ^60^ ^]^ Natural photosynthetic complexes achieve efficient photon capture and transfer by embedding cascaded FRET networks within lyotropic liquid crystalline phases formed by protein–lipid membranes.^[^ ^50^, ^51^, ^61^, ^62^ ^]^ In these environments, the anisotropic order of the lyotropic matrix enforces chromophore alignment and excitonic coupling, ensuring short FRET donor–acceptor distances, and directional energy flow with minimal losses while also providing protection against environmental degradation.^[^ ^50^, ^51^, ^61^, ^62^ ^]^ Recently, liquid crystals (LCs) have been utilized both as a matrix and as a FRET donor in a one‐step LHS with Nile red as the FRET acceptor.^[^ ^48^, ^49^ ^]^ With the idea of creeping closer to natural antennae, which operate via multistep energy transfer processes between chromophores, we implemented a liquid crystal‐based strategy with our molecular ribbons (Scheme 2). The readily available nematic LC (NLC) host, 4‐cyano‐4′‐pentylbiphenyl (**5CB**), will be explored for its anisotropic order and ability to align chromophores,^[^ ^63^ ^]^ thereby promoting excitonic coupling and enabling cascaded FRET (Scheme 2), resulting in panchromatic utilization of the visible region.^[^ ^64^ ^]^ Among the different O‐doped molecules prepared, derivatives **8**, **1**, and **4**, as well as **8**, **1**, and **6**, exhibit good spectral overlap, making them ideal for implementation in a multi‐step LHS. In this design, photons are absorbed by the yellow primary FRET donor (**8**), which starts the migration of excitation energy through a red FRET relay acceptor (**1**) and ultimately to the red‐absorbing final FRET acceptors (**4** or **6**), resulting in directional energy transfer within the ordered NLC phase (Scheme 2).

**Figure 9:**
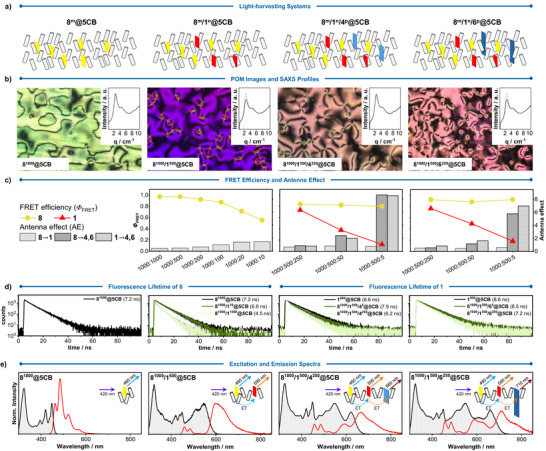
a) Schematic representation of the light‐harvesting systems. b) POM images and SAXS profiles (inset). c) Summary of FRET efficiency and AE values (x‐axis = *m*:*n*:*p*). d) Fluorescence lifetime of the primary FRET donor **8** (in **8^1000^@5CB** and **8^1000^/1*^n^* @5CB**) and relay acceptor **1** (in **8^1000^/1^500^/4*^p^* @5CB** and **8^1000^/1^500^/6*^p^* @5CB**). e) UV–vis excitation (black filled; *λ*_em_ varies depending on the last FRET acceptor) and emission (red line; *λ*_ex_ = 420 nm) spectra.

Since the chromophores are added to **5CB** at various molar ratios to optimize the energy transfer, the antennae materials will be labeled as ***α^m^*/*β^n^*/*γ^p^* @5CB**, where ***α***, ***β***, and ***γ*** represent the specific chromophores, ***m***, ***n***, and ***p*** indicate the ratios of the chromophores in 10^6^ **5CB** molecules (i.e., ***α***:**5CB **= ***m***:10^6^, ***β***:**5CB **= ***n***:10^6^, ***γ***:**5CB **= ***p***:10^6^), and **@5CB** signifies their presence in the NLC phase. For example, a system labeled as **8^1000^/1^500^/4^250^@5CB** has an **8**:**1**:**4**:**5CB** molar ratio of 1000:500:250:10^6^. Initially, the highest amount of primary FRET donor **8** into **5CB** was established at a 10^6^:1000 **5CB**/**8** ratio, where the NLC phase is retained as confirmed by polarized optical microscopy (POM; Figures 9b and S184). Based on this, a one‐step and two two‐step LHSs were constructed, namely **8^1000^/1*^n^* @5CB**, **8^1000^/1*^n^*/4*^p^* @5CB**, and **8^1000^/1*^n^*/6*^p^* @5CB**. POM confirmed successful integration of all chromophores in the NLC phase at RT (Figures 9b and S185–S188).

Small‐angle X‐ray scattering (SAXS) measurements were performed to assess how the addition of a given chromophore affects the order parameter of **5CB** (Figure 9b (insets) and S193). Addition of **8** alone leaves the oriented fraction essentially unchanged (63% for neat **5CB** versus 61% for **8^1000^@5CB**; Figure 9b, inset). In contrast, in **8^1000^/1^500^@5CB**, **8^1000^/1^500^/4^250^@5CB**, and **8^1000^/1^500^/6^250^@5CB** oriented fraction is reduced to 40 ± 10%, 53 ± 5%, and 42 ± 8%, respectively. In all samples, the main correlation peak remains at *q* = 2.51 nm^−1^, indicating unchanged lateral spacing, while its intensity decreases in line with the reduced oriented fraction of **5CB**. **8^1000^/1^500^@5CB** showed an unusually low order parameter than systems with higher chromophore doping. This phenomenon has been observed previously,^[^ ^65^, ^66^ ^]^ but it is difficult to determine the exact cause because various factors, such as the shape and size of dyes and intermolecular interactions, could be involved. It is hypothesized that the rod‐like features of PXX **4** could promote the enhancement of the order parameter.^[^ ^67^ ^]^ Additionally, no scattering attributable to the formation of nanoclusters was observed.

Next, we characterized the photophysical properties of the different LHSs. Starting with chromophore **8** alone, **8^1000^@5CB** exhibits an emission peak at 464 nm, Φ_F_ of 0.28, and *τ*_F_ of 7.2 ns (Figure 9d). Compared to the CH_2_Cl_2_ solution, the emission maximum exhibits a bathochromic shift from 451 to 464 nm, and a decrease in Φ_F_ from 0.52 to 0.28, both consistent with the inner‐filter effect (self‐absorption). This is evident in the excitation and emission spectra of **8^1000^@5CB** (Figure 9e). While the shape of the excitation spectra is identical to that of **8** in solution (Figure 4, left), the emission spectra show a substantial attenuation of the 0–0 emission band, as expected at high concentrations due to its strong overlap with the S_0_→S_1_ absorption band. On the other hand, the *τ*_F_ of **8** increased from 5.4 ns in CH_2_Cl_2_ to 7.2 ns in **8^1000^@5CB**. This longer *τ*_F_ can be attributed to the inner‐filter effect and/or a radiation‐trapping artifact in which emitted photons are reabsorbed and reemitted, lengthening the measured decay.

We next characterized the energy transfer behavior of the one‐step LHS **8^1000^/1*^n^* @5CB (** *n* = 1000, 500, 200, 100, 20, and 10). Steady‐state and time‐resolved photoluminescence were recorded to quantify donor quenching and acceptor sensitization, from which the FRET efficiency and the AE were determined. Upon excitation of the FRET donor **8** at 420 nm, donor emission (450–550 nm) decreases with increasing loading of the FRET acceptor **1** (from **8^1000^/1^10^@5CB** to **8^1000^/1^1000^@5CB**), while simultaneously the acceptor emission (580–750 nm) increases, confirming FRET from **8** to **1** (Figure 9c). This resulted in an increase in FRET efficiency of **8** (Φ_FRET,_ **_8_**) from 0.55 (**8^1000^/1^10^@5CB**) to 0.97 (**8^1000^/1^500^@5CB** and **8^1000^/1^1000^@5CB**). Additionally, lifetime measurements (Figure 9d) show a successive reduction of *τ*_F_ of **8** from 7.2 ns in **8^1000^/1^10^@5CB** to 4.4 and 4.5 ns in **8^1000^/1^500^@5CB** and **8^1000^/1^1000^@5CB**, respectively. This supports the trend of Φ_FRET,_ **_8_**, as the more efficient FRET process lowers the *τ*_F_ value for donor **8**. Moreover, upon heating of a representative LHS (**8^1000^/1^500^@5CB**) to 45 °C to achieve an isotropic liquid phase, the Φ_FRET,_ **_8_** decreased to 0.89, indicating that increased molecular mobility slightly reduces FRET efficiency (Figure S150). Notably, the Φ_FRET,_ **_8_** of **8^1000^/1^500^@5CB** was retained after a year, further underlying the chemical stability of these systems (Figure S151). On the other hand, AE value decreased from 1.4 (**8^1000^/1^10^@5CB**) to 0.4 (**8^1000^/1^1000^@5CB**) (Figure 9c and Supporting Information, Section 4). This decrease mainly reflects a lower relative amount of primary FRET donor **8**, which can FRET to acceptor **1**. No detectable AE was observed in **8^1000^/1^5^@5CB**, indicating that the system reaches maximum AE at a 1000:10 **8**:**1** ratio.

Moving toward the two‐step LHSs, two final FRET acceptors, namely, molecules **4** and **6**, were independently added into liquid crystals **8^1000^/1^500^@5CB** at various **1**:**4** and **1**:**6** ratios, obtaining **8^1000^/1^500^/4*^p^* @5CB** (*p* = 250, 50, and 5) and **8^1000^/1^500^/6*^p^* @5CB** (*p* = 250, 50, and 5), respectively. Between the primary FRET donor **8** and relay FRET acceptor **1**, the AE values remained between 0.5 and 0.6 in both LHSs. However, the Φ_FRET,_ **_8_** in **8^1000^/1^500^/6*^p^* @5CB**, slightly decreased to 0.91. This could be due to a competing, less efficient direct FRET from **8** to **6**, which can bypass the cascading FRET.^[^ ^68^ ^]^ Meanwhile, the decrease in Φ_FRET,_ **_8_** in **8^1000^/1^500^/4*^p^* @5CB** is more pronounced, decreasing up to 0.80, which could be due to a more favored competing direct FRET from **8** to **4**. This direct FRET is favored over the former because the emission spectrum of **8** overlaps with the S_0_–S_2_ absorption transition of **4** (Figure S133).^[^ ^69^, ^70^ ^]^ The AE values from the relay FRET donor **1** to the final FRET acceptors **4** and **6** decreased from 8.5 (**8^1000^/1^500^/4^5^@5CB**) to 0.8 (**8^1000^/1^500^/4^250^@5CB**) and from 7.0 (**8^1000^/1^500^/6^5^@5CB**), to 0.8 (**8^1000^/1^500^/6^250^@5CB**), upon increasing the relative amount of the final FRET acceptor. A similar decrease was also observed in the AE values from the primary FRET donor **8** to the final FRET acceptors **4** (8.7–0.9) and **6** (5.8–0.6). Meanwhile, the FRET efficiency of the relay acceptor **1** (Φ_FRET,_ **_1_**) increases from 0.13 (**8^1000^/1^500^/4^5^@5CB**) to 0.74 (**8^1000^/1^500^/4^250^@5CB**), and 0.18 (**8^1000^/1^500^/6^5^@5CB)** to 0.76 (**8^1000^/1^500^/6^250^@5CB**), upon increasing the amount of the final FRET acceptors. Similar to the one‐step LHS, this observation is also supported by the concomitant decrease in *τ*_F_ of **1** from 7.9 to 6.2 and 8.5 to 7.2 ns for the liquid crystal architectures **8^1000^/1^500^/4*^p^* @5CB** and **8^1000^/1^500^/6*^p^* @5CB**, respectively.

To confirm that the two‐step FRET cascade, with two LHSs based on the primary FRET donor **8** and the final FRET acceptors **4** and **6**, was constructed without the relay FRET acceptor **1**, namely **8^1000^/4^50^@5CB** and **8^1000^/6^50^@5CB**. These formulations were prepared to enable direct comparison of Φ_FRET_ and AE. Compared to the analogous systems containing the relay FRET acceptor **1**, **8^1000^/4^50^@5CB** and **8^1000^/6^50^@5CB** exhibited lower AE values of 0.6 and 0.7 (versus 2.4 and 1.16), respectively, and lower Φ_FRET,_ **_8_** of 0.18 and 0.05 (versus 0.81 and 0.91), respectively. The significantly lower AE and Φ_FRET,_ **_8_** values for these LHSs indicate that the antenna effect from **8** to the final acceptors (**4** or **6**) relies on the FRET cascade through relay **1**, confirming a two‐step FRET relay. Additionally, the *τ*_PF_ of **8** (7.2 ns) in both systems is similar to that of a system with only **8** in the NLC phase (7.2 ns). Conversely, the higher Φ_FRET,_ **_8_** in **8^1000^/4^50^@5CB** compared to **8^1000^/6^50^@5CB** confirms the previously mentioned good spectral overlap between the emission of **8** and the S_0_ to S_2_ absorption transition of acceptor **4** (Figure S133).

From **8^1000^@5CB** to **8^1000^/1^500^/6^250^@5CB**, it is apparent that the excitation wavelength range that could be utilized in the LHSs was expanded from 300–460 nm to 300–740 nm (Figure 9e). Herein, the LHS **8^1000^/1^500^/6^250^@5CB** exhibits an excitation wavelength range spanning the entire visible spectrum. These results suggest that further π‐extension, from **4** to **6**, results in the achievement of an LHS that has better overall two‐step Φ_FRET_ and a broader excitation wavelength range. A three‐step LHS was also constructed based on **8** as the primary FRET donor, **1** and **4** as the relay FRET acceptors, and **6** as the final FRET acceptor (Figures S178–180). However, the excitation wavelength that could be used is similar to that of **8^1000^/1^500^/6^250^@5CB**.

## Conclusions

In this work, we introduce a general donor–acceptor design that unlocks oxidatively stable, oxygen‐doped *peri*‐xanthenoxanthene ribbons with unprecedented near‐infrared (NIR) photophysics and enables their functional integration into liquid‐crystal‐based light‐harvesting systems. By fusing benzoyl groups at the *pseudo‐peri* positions, we access the first benzoyl‐functionalized PXX derivatives, which combine *i)* strong bathochromic absorption and emission extending deep into the NIR, *ii)* high fluorescence quantum yields ($\Phi_{F}^{\mathrm{NIR}}$ up to 0.36), and *iii)* clean, reversible electrochromic and electrofluorochromic switching. Remarkably, Lewis‐acid adducts of these ribbons deliver *pure* NIR emission ($\Phi_{F}^{\mathrm{NIR}}$ ≈ 0.15–0.16), opening routes to redox‐gated NIR emitters. The different absorbance envelopes provide good spectral overlap between molecules **8**, **1**, and **4**, as well as between **8**, **1**, and **6**, making them suitable for multi‐step FRET. Integrating them into NLC hosts, we successfully constructed one‐ and two‐step light‐harvesting systems. An excellent Φ_FRET_ of 0.97 was achieved in a one‐step LHS (**8^1000^/1^500^@5CB**). For the two‐step FRET systems, LHSs **8^1000^/1^500^/4^250^@5CB** and **8^1000^/1^500^/6^250^@5CB** afforded overall Φ_FRET_ values of 0.62 and 0.70, respectively. The maximum AE values from the primary FRET donor **8** to the final FRET acceptors **4** and **6** were 8.7 and 5.8, respectively. Notably, a panchromatic excitation range of 300–740 nm could be utilized via a FRET relay in the **8^1000^/1^500^/6^250^@5CB**. Overall, our system translates the natural principle of LHS in lyotropic liquid crystalline organization into a synthetic platform, combining the dynamic order of LCs with the tunability of O‐doped ribbons to enable robust, spectrally programmable artificial antennae. These benzoyl‐functionalized PXX platforms combine redox‐tunable NIR emitters with mesogenic light‐harvesting architectures, advancing applications in artificial photosynthesis and NIR optoelectronics.

## Supporting Information

Supporting Information includes experimental methods, synthetic procedures, characterization data, NMR and HR‐MS spectra, single‐crystal XRD results, and extended photophysical characterization, as well as a description of the computational methods, orbital visualizations, and an extended discussion of the TD‐DFT results. The authors have cited additional references within the Supporting Information.^[^ ^71^, ^72^, ^73^, ^74^, ^75^, ^76^, ^77^, ^78^, ^79^, ^80^, ^81^, ^82^, ^83^, ^84^, ^85^, ^86^, ^87^, ^88^, ^89^, ^90^, ^91^, ^92^ ^]^

## Author Contributions

C.D.L.: Project design; synthesis; investigation; methodology; data curation; and writing the original manuscript. E.C.G.: Project design; spectroscopic characterization; spectroelectrochemistry; methodology; light‐harvesting studies; writing the original manuscript; and data curation. R.R.F.: Spectroscopic characterization; data curation; review; and editing. C.P.: SAXS characterization; methodology; writing; and data curation. H.P.: Supervision and interpretation of SAXS. P.K.M.: X‐ray characterization; data curation. L. v D.: Theoretical calculations; methodology; formal analysis; and writing. J.C.B.D.: Supervision of theory; review; and editing. Y.S.: Liquid‐crystals POM and DSC characterization; data curation; review; and editing. G.‐i. K.: Supervision of liquid‐crystal characterization and data curation. D.B.: Conceptualization; project design; project administration; supervision; writing the original manuscript; review; and funding acquisition. All authors have approved the final version of the manuscript.

## Conflict of Interests

The authors declare no conflict of interest.

## Supporting information



Supporting Information

Supporting Information

## Data Availability

The data that support the findings of this study are available in the Supporting Information of this article.
