# Supplementary material for: Benzoyl‐Xanthenoxanthenes: Versatile Chromophores for Light‐Engaging Applications
Source: Angew Chem Int Ed Engl. 2026 Jan 9;65(7):e23349. doi: 10.1002/anie.202523349 (PMC12887625; doi:10.1002/anie.202523349)
Supplement: Supplementary file 1 — Supporting Information [file ANIE-65-e23349-s001.pdf]

Supporting Information

Benzoyl-xanthenoxanthenes: Versatile Chromophores for Light-Engaging Applications

Cristian De Luca,† El Czar Galleposo,† Rúben R. Ferreira, Chiara Puccinelli, Herwig Peterlik, Pradip Kumar Mondal, Laurens van Dam, Johannes C. B. Dietschreit, Yoshimichi Shimomura, Gen-ichi Konishi, and Davide Bonifazi*

[*] Dr. C. De Luca, E. C. Galleposo, C. Puccinelli, R. Ferreira, and Prof. Dr. D. Bonifazi.

Institute of Organic Chemistry, Faculty of Chemistry, University of Vienna, 1090 Vienna, Austria

Email: davide.bonifazi@univie.ac.at

Prof. Mag. Dr. H. Peterlik, Faculty Center for Nano Structure Research, Faculty of Physics, University of Vienna, 1090 Wien.

L. van Dam, Dr. J. C. B. Dietschreit. Institute of Theoretical Chemistry, Faculty of Chemistry, University of Vienna, Währinger Straße 17, 1090 Vienna, Austria.

Y. Shimomura, Prof. Dr. G. Konishi, Department of Chemical Science and Engineering, Institute of Science Tokyo, Tokyo 152-8552, Japan.

Dr. P. K. Mondal − Elettra Sincrotrone Trieste S.C.p.A., Trieste 34149, Italy

[†] These authors contributed equally

Table of contents

1. General remarks 3
   1.1. Instrumentation 3
   1.2. Materials and methods 6
2. Synthetic procedures 7
2.1. NMR and HRMS spectra 23
3. Optoelectronic characterization 73
   3.1. Photophysical properties 73
   3.2. Electrochemical properties 96
4. Preparation and characterization of the light-harvesting systems (LHS) 106
   4.1 Photophysical characterization of nanoribbons in 5CB 106
   4.2 Photophysical characterization of light-harvesting systems 113
   4.3 Structure and thermal properties of the light-harvesting systems 135
5. Crystallographic data 140
6. Computational studies 144
7. References 170

1. General remarks
1.1. Instrumentation

Thin layer chromatography (TLC) was conducted on pre-coated aluminum sheets with 0.20 mm Merck Millipore Silica gel 60 with fluorescent indicator F254. TLC plates were visualized by exposure to ultraviolet light (254 or 366 nm).

Column chromatography was carried out using Merck Geduran silica gel 60 (particle size 40-63 μm).

Melting points (M.P.) were measured on a Buchi M-560 melting point apparatus equipped with a heating block (T_{max} = 300 °C) on open capillary tubes, under air, and are uncorrected. According to the limitations of the apparatus, the melting point of compounds which did not melt or decompose (dec) up to 300 °C are presented as "> 300 °C".

Nuclear magnetic resonance (NMR) characterizations were performed at the NMR Centre of the University of Vienna. NMR spectra were recorded on Bruker spectrometer AV III HD 700, AV III 600 or AV NEO 400. ^{1}H NMR spectra were obtained at 700, 600 or 400 MHz, ^{13}C NMR spectra at 176, 151 or 101 MHz, ^{19}F NMR spectra at 659 or 565 MHz, ^{11}B NMR spectra at 193 MHz in quartz NMR tube. All spectra were obtained at room temperature. Carbon spectra were acquired with a complete decoupling for the proton. Proton and carbon chemical shifts are reported in parts per million (ppm, δ scale) according to tetramethylsilane ($\delta_H = \delta_C = 0$ ppm) using the solvent residual signal as an internal reference ($CDCl_3$: δ_H = 7.26 ppm, δ_C = 77.16 ppm; Acetone-d_6: δ_H = 2.05 ppm, δ_C = 29.87 ppm; DMSO-d_6: δ_H = 2.50 ppm, δ_C = 39.52 ppm; C_6D_6: δ_H = 7.16 ppm, δ_C = 128.06 ppm; CD_2Cl_2: δ_H = 5.32 ppm, δ_C = 54.00 ppm). Boron chemical shifts are reported in ppm, referenced to the external standard boron signal of $BF_3 \cdot Et_2O$ (δ_B = 0 ppm). Coupling constants (*J*) are given in Hz. Resonance multiplicity is described as s (singlet), d (doublet), dd (doublet of doublets), ddd (doublet of doublets of doublets), t (triplet), td (triplet of doublets), q (quartet), p (pentet), sept (septet) m (multiplet) and bs (broad signal).

Infrared spectra (IR) were recorded on a Bruker Alpha FT-IR spectrometer in ATR mode. Selected absorption bands are reported in wavenumber (cm^{-1}).

High-resolution mass spectrometry (HRMS) analyses were performed at the Mass Spectrometry Centre of the University of Vienna. ESI mass spectra were obtained on a Thermo Fisher Scientific Orbitrap Exploris 120™ Mass Spectrometer in the positive or negative ion mode, GC mass spectra on an Agilent 7200B GC/Q-TOF mass spectrometer, LD and MALDI mass spectra on a Bruker Autoflex Speed LD-timsTOF or MALDI-timsTOF (matrix: 2-[(2E)-3-(4-tert-butylphenyl)-2-methylprop-2-enylidene]malononitrile (DCTB) in CH_2Cl_2) mass spectrometer.

Preparative Gel Permeation Chromatography was carried out using Bio-Rad Bio-beads® S-X1 (200-400 mesh).

Preparative Recycling Gel Permeation Chromatography was performed on a Japan Analytical Industry Co, Ltd. LaboACE LC-7080-Plus liquid chromatograph equipped with UV detector (from 200 nm to 800 nm), two columns (JAIGEL-2 HR) and precolumn JAIGEL-HR-P (ID 8 mm × 40 mm). Standard operating conditions were as follows: eluent DCM, flow rate 10 mL/min, column pressure ≤ 5 MPa, injection volume < 5 mL, ambient temperature.

Ultraviolet–Visible (UV-vis) **absorption spectroscopy** was recorded using Agilent Cary 5000 UV–vis–NIR Spectrophotometer running in double beam mode with a matched pair of quartz absorbance cuvettes (1 × 1 cm). All absorption measurements were performed at room temperature, unless specified otherwise. The molar attenuation coefficient (ε) was determined by dissolving a known amount of compound (typically 1–2 mg) and diluting the resulting stock solution to achieve 5 solutions with an appropriate concentration for measurements. The plot of absorbance versus concentration was fitted with a linear function and the molar attenuation coefficient was obtained from the slope.

UV–vis–NIR emission spectroscopy: The photoluminescence (PL) excitation and emission spectra, absolute quantum yield, and decay curves were recorded on a FLS1000 photoluminescence spectrometer (Edinburgh Instruments, UK). The spectrometer was equipped with excitation and emission double grating Czerny-Turner monochromators, a photomultiplier detector with extended near-infrared sensitivity (PMT-980), photon-counting NIR detector (PMT-1700), fitted with a gating circuit and thermoelectrically cooled to −20 °C with a fan-assisted Peltier element, and a high-speed PMT detector with a response width < 180 ps operating at 0 °C. All samples were prepared in air-equilibrated dry CH_2Cl_2, unless otherwise stated. The maximum absorbance of all solutions was adjusted to c.a. 0.1 to avoid inner filter effect. While liquid crystal samples were placed between two quartz slides. For steady-state measurements, the samples were excited using a 450 W ozone-free continuous Xenon arc lamp. Time-resolved measurements in the ns range were performed by irradiating the samples with a suitable nano-pulsed lasers, EPL-375, EPL-405 or EPL-505 and acquired using the High Speed PMT detector in Time-Correlated Single Photon Counting (TCSPC) mode. The tail portion of the decay curves were fitted using the FAST software (Edinburgh Instruments, UK), following a single exponential model with y-offset (background-offset):

$$I(t)=A+B\cdot e^{\frac{-t}{\tau}} \quad (1)$$

where A is the y-offset, B the pre-exponential factor, and τ the lifetime. For decays close to the pulse width of the light source, the instrument response function (IRF) was measured using a Ludox® solution (at room temperature) or using the sample itself (at low temperature). In both cases the count rate was adjusted using a computer-controlled neutral density filter wheel in order to match the count rate of the sample emission. In these cases, the decay lifetime was obtained by performing a reconvolution fit using the FAST software. Absolute quantum yields were measured using an integrating sphere (internal diameter 120 mm) fitted on the FLS1000 sample chamber. The samples and blank reference (solvent) were placed in a 1 × 1 cm fluorescence quartz cuvette (liquid crystal samples and blank reference (blanking plug) were measured in a solid sample holder) and the calculations were done using the "direct excitation" method following the equation:

$$\Phi=\frac{E_B - E_A}{S_A - S_B} \quad (2)$$

where E_B and E_A correspond to the integrated fluorescence emission of the sample and blank reference (solvent), respectively. S_A and S_B refer to the integrated excitation scatter region of the reference and the sample, respectively. For measuring the scatter region, and avoid detector saturation, a neutral density filter (OD = 1 or 2) was placed between the integrating sphere exit and the detector in order to attenuate the signal. An excitation bandwidth of 3 – 10 nm was used to ensure the determination of the sample absorption with high accuracy (step = 1 nm), while the emission bandwidth was chosen in order to obtain a strong sample emission signal (peak emission > 10^4 cps).

The optical bandgap E_g^{00}, was calculated using the intercept of the excitation and emission spectra (λ_{00}) following equation:

$$E_g^{00}(eV)=\frac{1240\ eV\cdot nm}{\lambda_{int}} \quad (4)$$

Electrochemical analysis: Cyclic voltammetry experiments were performed at room temperature in dry CH_2Cl_2, using an Autolab PGSTAT204 potentiostat (Metrohm, DE). A conventional three-electrode electrochemical cell connected to an argon source and an oil bubbler was used. Dry argon gas was bubbled through the sample solution for at least 15 min prior to each measurement and the headspace was continuously flushed throughout the experiment. A pre-bubbler filled with solvent was used in order to prevent evaporation. Glassy carbon disk (7 mm^2 diameter) was used as a working electrode, Pt wire as an auxiliary electrode, and Ag/AgCl as a reference electrode.

The glassy carbon working electrode was polished on a pad using alumina slurry and washed with deionized H_2O before each experiment; the Pt wire was flame-cleaned. Tetrabutylammonium hexafluorophosphate (Alfa Aesar, $TBAPF_6$) was twice recrystallized from absolute ethanol prior to use and it was added to the solution as a supporting electrolyte at a concentration of 0.1 M. Decamethylferrocene (Sigma Aldrich) or Ferrocene (Sigma Aldrich) was used as an internal reference. The formal redox potentials (half-wave potentials) were calculated using the formula:

$$E_{1/2}=\frac{E_{\mathrm{pa}}+E_{\mathrm{pc}}}{2} \qquad (6)$$

where E_{pa} is the peak anodic potential and E_{pc} is the peak cathodic potential. The energy of the HOMO was estimated using the following equation:

$$E_{\mathrm{HOMO}}(\mathrm{eV})=-\left(E_{1/2}^{\mathrm{ox}}-0.55\ \mathrm{V}\right)-4.8 \qquad (7)$$

where E_{ox} is the half-wave oxidation potential (vs Fc/Fc^+) and 4.8 is the HOMO energy of ferrocene in vacuum. To reference the oxidation potentials vs Fc/Fc^+, we experimentally determined the potential of the $DmFc/DmFc^+$ redox couple to be −0.55 V vs Fc/Fc^+.

Spectroelectrochemical and spectrofluoroelectrochemical characterization was performed using a thin layer quartz cuvette (path length of 1 mm) equipped with an optically transparent platinum minigrid working electrode, a platinum wire auxiliary electrode, and an Ag/AgCl reference electrode.

X-ray measurements: of **1**, and **8** were performed at the Centre for X-ray Structure Analysis of the University of Vienna. X-ray intensity data were measured at 100 K on a STOE Stadivari diffractometer equipped with dual radiation source Mo and Cu Kα, and a Dectris EIGER2 R 500K detector. The structures were solved ab initio and refined by full-matrix least-squares techniques. Hydrogen atoms were inserted at calculated positions using AFIX instructions, while all other atoms were refined with anisotropic displacement parameters.

Data collections for **2** and **10** were performed at the XRD1 beamline of the Elettra Synchrotron, Trieste (Italy).[1] The crystals were dipped in NVH oil (Jena Bioscience, Jena, Germany) and mounted on the goniometer head with nylon loops (MiTeGen, Ithaca, USA). Complete datasets were collected at 100 K (nitrogen stream supplied through an Oxford Cryostream 700). Data were acquired using a monochromatic wavelength of 0.70 Å through the rotating crystal method on a Pilatus 2M hybrid-pixel area detector (DECTRIS Ltd., Baden-Daettwil, Switzerland). The diffraction data were indexed and integrated using XDS.[2] The structure was solved with Olex2[3] by using ShelXT[4] structure solution program by Intrinsic Phasing and refined with the ShelXL[5] refinement package using least-squares minimization. In the last cycles of refinement, non-hydrogen atoms were refined anisotropically. Hydrogen atoms were included in calculated positions, and a riding model was used for their refinement.

Quantum chemical calculations of molecules **1**, **2**, **3**, **4**, **5**, **6** and **8** were performed on the Vienna Scientific Cluster (VSC), by using the program package "Gaussian16, Revision A.03".[6] The geometry of the input molecules was pre-optimized with the method UFF by using Avogadro. The geometry optimizations were carried out using the hybrid functional B3LYP, in combination with the

basis set 6-31G(d). The solvent effect (CH_2Cl_2) was included in the calculation by using the implicit polarizable continuum model (PCM). The energy of the first 64 electronically excited singlet state was calculated using the TD-DFT method, adopting the same functional and basis set used for geometry optimization. Even in this case, the solvent effect was taken into account by introducing the PCM in the calculation.

Differential scanning calorimetry (DSC) was conducted using a TA Discovery DSC instrument (TA Instrument) under N_2 atmosphere at temperatures between −40 and 60 °C with a ramp rate of 10 °C/min.

Polarized optical microscopy (POM) was conducted using a Leica DM2500 LED optical microscope, equipped with polarizer, analyzer, and lambda plate at 20× magnification.

Small-angle X-ray scattering (SAXS) measurements were performed with CuKα radiation generated by a microfocus source (Incoatec High Brilliance), equipped with a pinhole camera and an area detector (Bruker Nanostar and Vantec 2000, Bruker AXS). The samples were measured in transmission at 28 cm. The X-ray data were then radially averaged to obtain the scattering intensities in dependence on the scattering vector $q = 4\pi/\lambda \sin(\theta)$, with 2θ being the scattering angle and $\lambda = 0.1542$ nm the x-ray wavelength. Measurements were done at four different positions for one hour.

1.2. Materials and methods

6-(2,6-dimethylphenyl)naphthalen-2-ol was prepared according to literature procedures.[7] Eaton's reagent (ER) was prepared following the original protocol.[8] Freshly prepared ER was stored under Argon at 5 °C for not more than 30 days. Chemicals were purchased from Sigma Aldrich, Acros Organics, TCI, Alfa Aesar, Fluorochem, Thermo Fisher Scientific and BLDpharm and used without further purification. Mesitylene was distilled over CaH_2 and stored in a Strauss flask under Argon atmosphere. Anhydrous toluene and tetrahydrofuran (THF) were dried on a MBraun SPS-800 solvent purification system, degassed and stored over activated 4 Å molecular sieves. Deuterated solvents were purchased from Eurisotop and Apollo scientific. Anhydrous conditions were achieved by drying glassware in oven at 120 °C for at least 12 h and by flame-frying the reaction vessels with a heat gun under vacuum and purging with Argon. The inert atmosphere was maintained using Argon-filled balloons equipped with a syringe and needle that was used to penetrate the silicon septa used to close the flask's necks. Addition of liquid reagents was performed using Argon-purged plastic or glass syringes. Alternative to the use of Schlenk line techniques, inert conditions were achieved by using an Argon-filled MBraun LabStar glove box when stated. Degassing of solutions was performed by bubbling Argon or *freeze-pump-thaw* procedure: solutions were frozen in liquid Nitrogen and kept under vacuum for 10-15 min before thawing. 0 °C baths were prepared using ice/H_2O. When heating in a closed flask, the reactions were performed in Schlenk tubes filled with argon and closed with oven-dried glass stoppers and Glindemann PFTE sealing rings for gas-tight joints.

2. Synthetic procedures

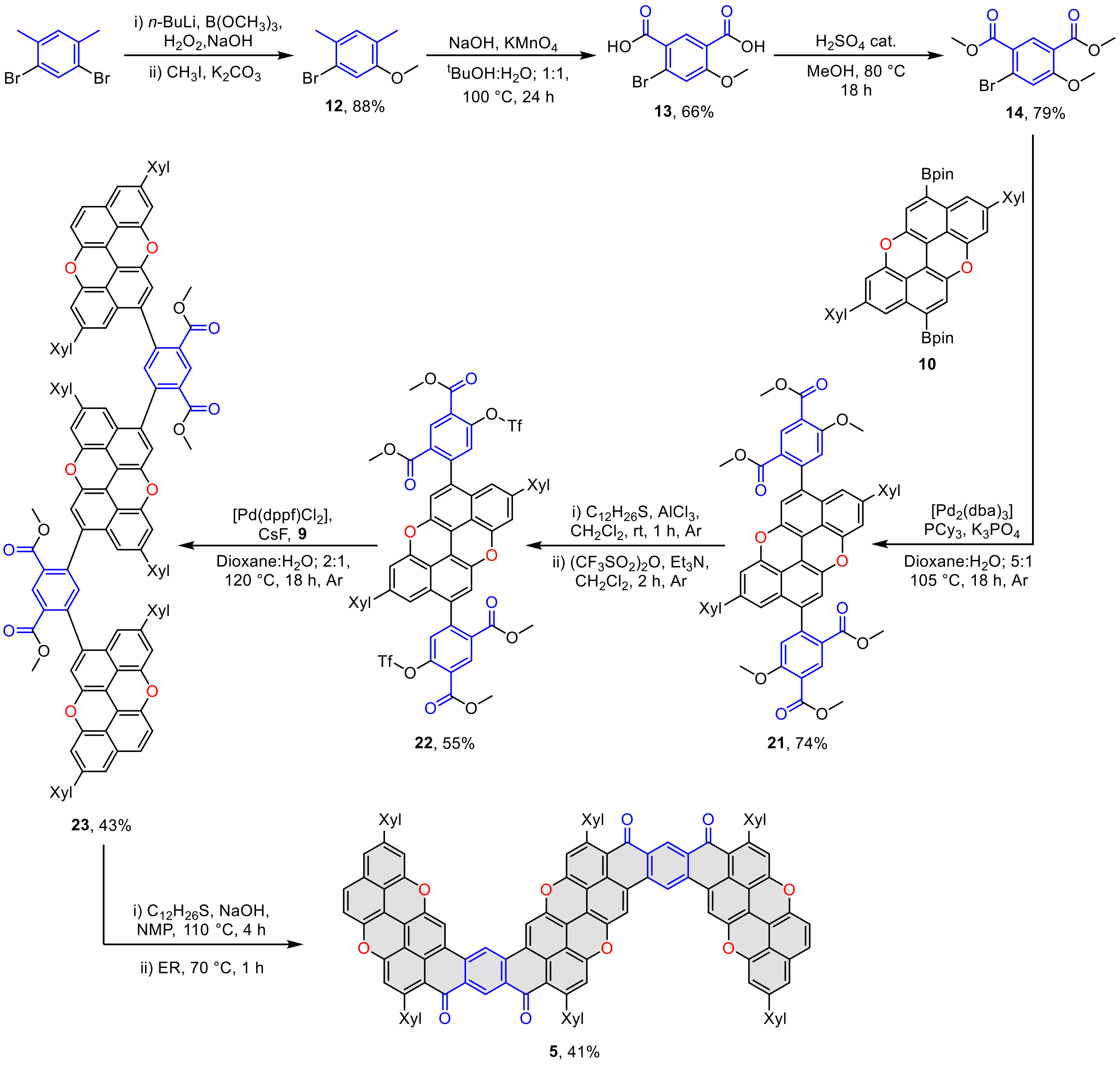

Scheme S1. Synthetic pathway adopted for the synthesis of compound **5**.

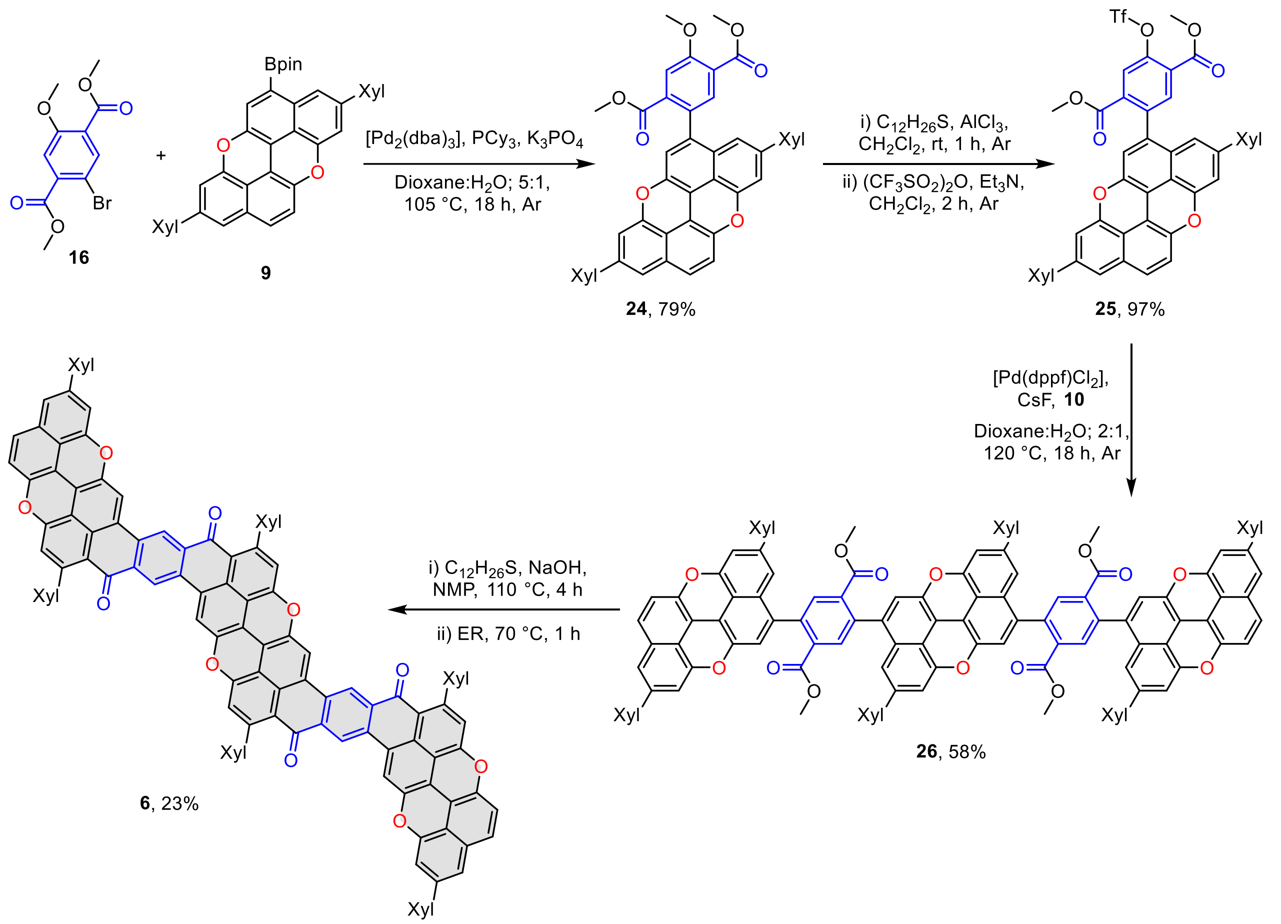

Scheme S2. Synthetic pathway adopted for the synthesis of compound **6**.

6,6'-bis(2,6-dimethylphenyl)-[1,1'-binaphthalene]-2,2'-diol (7)

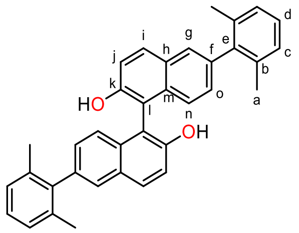

Procedure adapted from literature.[9]

To a 100 mL round-bottomed flask, CH_2Cl_2 (25.6 mL) was added to a mixture of Cu-TMEDA catalyst (0.02 eq, 238 mg, 0.512 mmol) and 6-(2,6-dimethylphenyl)naphthalen-2-ol (6.36 g, 25.6 mmol) and the resulting dark green mixture was stirred at room temperature for 4 h. Concentration followed by column chromatography (SiO_2, CH_2Cl_2/heptane 7:3) afforded **7** (yield = quant., 6.30 g) as white solid. **M.P.** 158 – 160 °C. **^{1}H NMR** (600 MHz, $CDCl_3$) δ 7.98 (d, J = 8.9 Hz, 2H, H_i), 7.69 (s, 2H, H_g), 7.43 (d, J = 8.9 Hz, 2H, H_j), 7.34 (d, J = 8.5 Hz, 2H, H_o), 7.22 – 7.17 (m, 4H, H_n, H_d), 7.16 – 7.11 (m, 2H, H_c), 5.13 (bs s, 2H, OH), 2.08 (s, 12H, H_a). **^{13}C NMR** (151 MHz, $CDCl_3$) δ 152.88 (C_k), 141.51 (C_f), 137.01 (C_b), 136.53 (C_e), 136.49 ($C_{e'}$), 132.23 (C_h), 131.58 (C_i), 129.77 (C_m), 129.50 (C_d), 128.42 (C_g), 127.51 (C_c), 127.33 (C_n), 124.44 (C_o), 118.05 (C_j), 111.03 (C_l), 21.22 (C_a). **IR** (neat): 3530, 3504, 3463, 3420, 3365, 3054, 2950, 2848, 2420, 2364, 2208, 1920, 1891, 1621, 1597, 1498, 1462, 1442, 1405, 1379, 1350, 1309, 1251, 1213, 1173, 1127, 1100, 1030, 986, 966, 935, 890, 827, 812, 768, 728, 691, 622, 588, 557, 545, 529. **HRMS** (ESI-orbitrap): m/z calcd for $C_{36}H_{29}O_2^-$: 493.2173 $[M]^-$; found: 493.2173.

2,8-bis(2,6-dimethylphenyl)xantheno[2,1,9,8-klmna]xanthene (8)

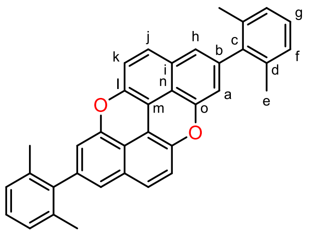

Procedure adapted from literature.[10]

To a 50 mL round-bottomed flask under ambient air, 6,6'-bis(2,6-dimethylphenyl)-[1,1'-binaphthalene]-2,2'-diol **7** (1.13 g, 2.22 mmol), K_2CO_3 (2 eq, 614 mg, 4.44 mmol), CuCl (0.3 eq, 66 mg, 0.67 mmol)and xylenes (isomeric mixture, 10 mL) were added, followed by *N*-methylimidazole (0.6 eq, 110 µL, 1.33 mmol). The mixture was heated at 120 °C for 20 h. After let cooling the reaction mixture to room temperature, it was poured into H_2O (50 mL) and extracted with CH_2Cl_2 (2 × 30 mL). The combined organic layers were filtered through a short pad of celite, dried over $MgSO_4$ and evaporated in *vacuo*. The crude material was chromatographed (SiO_2, CH_2Cl_2/heptane 5:95) to give **8** (yield = 60%, 784 mg) as a yellow solid. **M.P.** >300 °C. **^{1}H NMR** (600 MHz, CD_2Cl_2) δ 7.27 (d, J = 9.0 Hz, 2H, H_j), 7.12 – 7.04 (m, 2H, H_g), 7.05 – 7.00 (m, 4H, H_f), 6.90 (d, J = 9.0 Hz, 2H, H_k), 6.83 (d, J = 1.0 Hz, 2H, H_h), 6.43 (d, J = 1.0 Hz, 2H, H_a), 2.03 (s, 6H, H_e). **^{13}C NMR** (151 MHz, CD_2Cl_2) δ 153.20 (C_l), 144.68 (C_o), 141.75 (C_i), 140.92 (C_b), 136.37 (C_d), 131.88 (C_n), 127.69 (C_f), 127.58 (C_g), 126.86 (C_j), 120.77 (C_c), 120.58 (C_h), 117.89 (C_k), 111.86 (C_m), 110.85 (C_a), 20.87 (C_e). **IR** (neat): 2956, 2922, 2849, 1728, 1630, 1601, 1581, 1496, 1411, 1377, 1347, 1273, 1225, 1207, 1182, 1128, 1098, 1076, 1032, 950, 920, 860, 823, 799, 763, 727, 713, 695, 676, 639, 580, 539. **HRMS** (LD-timsTOF): *m/z* calcd for $C_{36}H_{26}O_2^+$: 490.1927 [M]$^+$; found: 490.1916. Single crystals suitable for X-ray diffraction were obtained by slow diffusion of MeOH in CH_2Cl_2. (CCDC #2386241) For X-ray diffraction data see Section 4.

2-(2,8-bis(2,6-dimethylphenyl)xantheno[2,1,9,8-klmna]xanthen-4-yl)-4,4,5,5-tetramethyl-1,3,2-dioxaborolane and 2,2'-(2,8-bis(2,6-dimethylphenyl)xantheno[2,1,9,8-klmna]xanthene-4,10-diyl)bis(4,4,5,5-tetramethyl-1,3,2-dioxaborolane) (9 and 10)

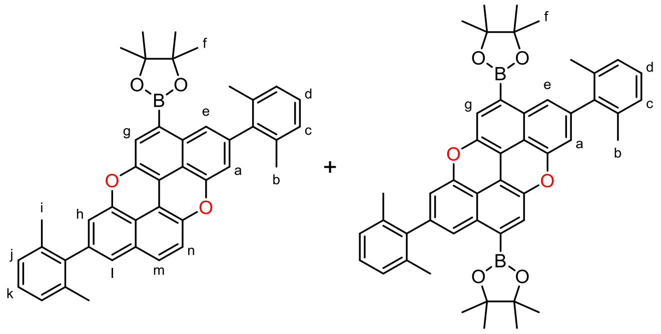

9 **10**

Procedure adapted from literature.[11]

In an Argon-filled glovebox, a 250 mL Schlenk tube was charged with **8** (245 mg, 0.5 mmol), B_2pin_2 (4 eq, 508 mg, 2 mmol), [Ni(cod)$_2$] (0.15 eq, 21 mg, 0.075 mmol) and dcype (0.2 eq, 42 mg, 0.1 mmol) were added in the same tube. Outside the glovebox 2 mL of freshly distilled and degassed mesitylene were added to the mixture. The mixture was stirred at room temperature for 10 min; then freshly distilled and degassed TFE (4 eq, 144 µL, 2 mmol) was added. The reaction vessel was sealed and then heated at 170 °C for 20 h. The mixture was allowed to cool to room temperature and diluted with CH_2Cl_2 (20 mL), the organic layer was washed with a sat. aq. solution of $NaHCO_3$ (2 × 20 mL).

The organic layer was dried over $MgSO_4$, filtered over a Celite® pad and concentrated in *vacuo*. The crude was chromatographed (SiO_2, CH_2Cl_2/heptane 5:95 to CH_2Cl_2/heptane 1:1) to give at first unreacted starting material, successively **9** (yield = 36%, 133 mg) as a yellow solid and **10** (yield = 31%, 96 mg) as a dark yellow solid.
9: **M.P.** (dec) 233 °C. **^{1}H NMR** (600 MHz, CD_2Cl_2) δ 7.77 (d, J = 1.1 Hz, 1H, H_e), 7.55 (s, 1H, H_g), 7.40 (d, J = 9.0 Hz, 1H, H_m), 7.23 – 7.06 (m, 6H, H_c, H_d, H_j, H_k), 7.00 (d, J = 9.0 Hz, 1H, H_n), 6.91 (d, J = 0.8 Hz, 1H, H_l), 6.55 (d, J = 1.1 Hz, 1H, H_a), 6.53 (d, J = 0.8 Hz, 1H, H_h), 2.14 (s, 6H, H_b), 2.12 (s, 6H, H_i), 1.34 (s, 12H, H_f). **^{13}C NMR** (151 MHz, CD_2Cl_2) δ 153.14, 152.92, 145.11, 144.09, 142.17, 141.75, 140.97, 140.67, 136.46, 136.37, 135.54, 131.76, 127.82 (C_c, C_d, C_j, C_k), 127.68 (C_c, C_d, C_j, C_k), 127.60 (C_c, C_d, C_j, C_k), 127.58 (C_c, C_d, C_j, C_k), 127.45 (C_m), 126.78 (C_g), 122.14 (C_e), 120.87 (C_l), 120.59, 120.47, 117.79 (C_n), 114.97, 111.56, 110.96 (C_h), 110.90 (C_a), 84.32, 25.02 (C_f), 21.03 (C_b), 20.87 (C_i), one signal missing due to ^{11}B-induced quadrupolar relaxation. **^{11}B NMR** (193 MHz, CD_2Cl_2) δ 30.43. **IR** (neat): 2963, 2922, 1629, 1596, 1580, 1470, 1402, 1368, 1318, 1305, 1260, 1233, 1211, 1185, 1163, 1142, 1087, 1043, 1016, 966, 956, 924, 896, 850, 794, 768, 704, 673, 527. **HRMS** (LD-timsTOF): *m/z* calcd for $C_{42}H_{37}BO_4^+$: 616.2787 $[M]^+$; found: 616.2781.
10: **M.P.** >300 °C. **^{1}H NMR** (600 MHz, CD_2Cl_2) δ 7.76 (d, J = 1.2 Hz, 2H, H_e), 7.56 (s, 2H, H_g), 7.20 – 7.16 (m, 2H, H_d), 7.16 – 7.12 (m, 4H, H_c), 6.55 (d, J = 1.2 Hz, 2H, H_a), 2.14 (s, 12H, H_b), 1.34 (s, 24H, H_f). **^{13}C NMR** (151 MHz, CD_2Cl_2) δ 152.86, 144.47, 142.16, 140.68, 136.46, 135.39, 127.81 (C_d), 127.44 (C_c), 126.67 (C_g), 122.02 (C_e), 120.70, 114.60, 111.00 (C_a), 84.36, 25.01 (C_f), 21.03 (C_b), one signal missing due to ^{11}B-induced quadrupolar relaxation. **^{11}B NMR** (193 MHz, CD_2Cl_2) δ 30.60. **IR** (neat): 2959, 2923, 2855, 1725, 1629, 1581, 1500, 1465, 1375, 1261, 1184, 1163, 1139, 1073, 1031, 978, 924, 852, 800, 768, 742, 705, 672, 569. **HRMS** (MALDI-timsTOF): *m/z* calcd for $C_{48}H_{48}B_2O_6^+$: 742.3647 $[M]^+$; found: 742.3649. Single crystals suitable for X-ray diffraction were obtained by slow diffusion of MeOH in CH_2Cl_2. (CCDC #2306818) For X-ray diffraction data see Section 4.

dimethyl 4,6-dibromoisophthalate (11)

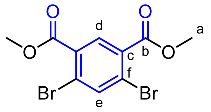

5 drops of conc. H_2SO_4 were added to a solution of 4,6-dibromoisophthalic acid (648 mg, 2 mmol) in dry MeOH (10 mL) in a 25 mL round-bottomed flask. The reaction mixture was heated at 80 °C for 18 h. The mixture was allowed to cool to room temperature and poured into H_2O (20 mL), basified with a solution 25% $NH_4OH_{(aq)}$ (pH ≃ 10) and extracted with Et_2O (3 × 50 mL). The combined organic layers were dried over anhydrous $MgSO_4$, and the solvent removed in *vacuo* to give **11** (yield = 72%, 506 mg) as a white solid. **M.P.** 99.8 – 101.6 °C. **^{1}H NMR** (700 MHz, $CDCl_3$) δ 8.29 (s, 1H, H_d), 8.02 (s, 1H, H_e), 3.94 (s, 6H, H_a). **^{13}C NMR** (176 MHz, $CDCl_3$) δ 165.02 (C_b), 140.00 (C_d), 134.18 (C_e), 130.87 (C_c), 126.05 (C_f), 52.94 (C_a). **IR** (neat): 2958, 2920, 1731, 1578, 1541, 1449, 1424, 1333, 1279, 1234, 1187, 1115, 1050, 979, 946, 911, 885, 868, 807, 768, 696, 568, 501, 489, 473, 460, 428, 416. **HRMS** (ESI-orbitrap): *m/z* calcd for $C_{10}H_7Br_2O_4^-$: 348.8717 $[M]^-$; found: 348.8717.

1-bromo-5-methoxy-2,4-dimethylbenzene (12)

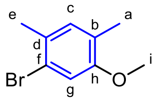

Procedure adapted from literature.[12]

n-butyllithium (1.6 M in hexanes, 1.1 eq, 2.6 mL, 4.18 mmol) was added dropwise to a solution of 1,5-dibromo-2,4-dimethylbenzene (1.00 g, 3.8 mmol) in dry THF (14 mL) at – 78 °C. After stirring at – 78 °C for 2 h, $B(OCH_3)_3$ (1 eq, 424 µL, 3.8 mmol) was added dropwise. The reaction mixture was allowed to slowly warmto room temperature and stirring was maintained for 18 h. Volatiles were removed in *vacuo.* The resulting pale-yellow waxy solid was dissolved in THF (10 mL) and $H_2O_{2(aq)}$ (30%, 1.3 mL) and $NaOH_{(aq)}$ (1 M, 2.5 mL) were sequentially added, and the reaction mixture stirred at room temperature for 2 h. The reaction mixture was quenched with a sat. $NH_4Cl_{(aq)}$ solution and extracted with Et_2O (2 × 20 mL). The combined organic layers were dried over $MgSO_4$ and concentrated in *vacuo*. The obtained yellow waxy solid was redissolved in dry DMF (2 mL), successively K_2CO_3 (2.5 eq, 1.31 g, 9.5 mmol) and CH_3I (2.5 eq, 591 µL, 9.5 mmol) were added, and the reaction heated at 100 °C for 18 h. The mixture was allowed to cool to room temperature and poured into H_2O (25 mL) and extracted with Et_2O (2 × 20 mL). The combined organic layers were dried over $MgSO_4$ and concentrated in *vacuo*. The resulting pale-yellow solid was chromatographed (SiO_2, CH_2Cl_2/heptane 7:3) to give **12** (yield = 88%, 712 mg) as a colorless oil. **^{1}H NMR** (700 MHz, $CDCl_3$) δ 7.00 (s, 1H, H_g), 6.99 (s, 1H, H_c), 3.81 (s, 3H, H_i), 2.32 (s, 3H, H_e), 2.16 (s, 3H, H_a). **^{13}C NMR** (176 MHz, $CDCl_3$) δ 156.39 (C_h), 132.50 (C_c), 129.09 (C_d), 125.85 (C_b), 121.50 (C_f), 114.13 (C_g), 55.69 (C_i), 21.76 (C_e), 15.83 (C_a). **IR** (neat): 2949, 2921, 2835, 1606, 1574, 1492, 1462, 1434, 1385, 1299, 1250, 1207, 1183, 1156, 1037, 957, 873, 834, 782, 716, 606, 482, 445. **HRMS** (GC-QTOF): *m/z* calcd for $C_9H_{11}BrO$: 213.9993 [M]; found: 213.9980.

4-bromo-6-methoxyisophthalic acid (13)

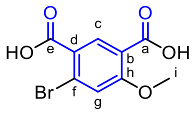

12 (4.32 g, 20 mmol) and sodium hydroxide (1.8 eq, 1.45 g, 36 mmol) were dissolved in a mixture of *tert*-butanol/ H_2O (1:1, 320 mL). The reaction mixture was heated to 100 °C while stirring, and a first portion (12,6 g) of $KMnO_4$ (total: 8 eq, 25.2 g, 160 mmol) was added to the solution. After stirring for 8 h a second portion (12.6 g) of $KMnO_4$ was added and the deep purple solution kept at 100 °C for another 18 h. The excess of $KMnO_4$ was reduced by addition of sodium thiosulfate pentahydrate (2 g) and the hot solution immediately filtered over a Celite® pad. The resulting solid was washed with boiling H_2O (600 mL). The aqueous filtrate was concentrated to eliminate the *tert*-butanol and acidified to pH = 1 with conc. HCl. The white precipitate formed was further collected by filtration, washed with cold H_2O (1 L) and dried in an oven at 100 °C for 18 h to give the **13** (yield = 70%, 3.84 g) as a white solid. **M.P.** 249.7 – 250.6 °C. **^{1}H NMR** (700 MHz, Acetone-*d*6) δ 8.47 (s, 1H, H_c), 7.51 (s, 1H, H_g), 4.07 (s, 3H, H_i), missing the 2 acidic protons. **^{13}C NMR** (176 MHz, Acetone-*d*6) δ 165.73 (C_e), 165.50 (C_a), 161.84 (C_h), 136.25 (C_c), 128.31 (C_f), 124.54 (C_d), 120.10 (C_b), 119.53 (C_g), 57.35 (C_i). **IR** (neat): 2981, 2895, 1698, 1595, 1551, 1495, 1406, 1297, 1249, 1189, 1143, 1101, 1021, 941, 891, 847, 769, 687, 637, 541, 469, 446, 434, 417. **HRMS** (ESI-orbitrap): *m/z* calcd for $C_9H_6BrO_5^-$: 272.9404 $[M]^-$; found: 272.9403.

dimethyl 4-bromo-6-methoxyisophthalate (14)

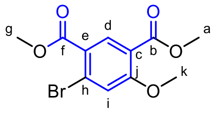

5 drops of conc. H_2SO_4 were added to a solution of **13** (1.00 g, 3.65 mmol) in dry MeOH (8 mL) in a 25 mL round-bottomed flask. The reaction mixture was heated at 80 °C for 18 h. The mixture was allowed to cool to room temperature and poured into H_2O (20 mL), basified with a solution 25%

$NH_4OH_{(aq)}$ (pH ≃ 10) and extracted with Et_2O (3 × 50 mL). The combined organic layers were dried over $MgSO_4$, and the solvent removed in *vacuo* to give **14** (yield = 79%, 878 mg) as a white solid. **M.P.** 169.1 – 170.8 °C. **^{1}H NMR** (600 MHz, DMSO-*d*6) δ 8.17 (s, 1H, H_d), 7.52 (s, 1H, H_i), 3.93 (s, 3H, H_k), 3.84 (s, 3H, H_g), 3.80 (s, 3H, H_a). **^{13}C NMR** (151 MHz, DMSO-*d*6) δ 164.49 (C_b), 164.48 (C_f), 160.50 (C_j), 133.97 (C_d), 126.81 (C_h), 122.67 (C_e), 118.85 (C_c), 118.70 (C_i), 56.86 (C_k), 52.46 (C_g), 52.30 (C_a). **IR** (neat): 2921, 1715, 1693, 1589, 1547, 1487, 1464, 1454, 1427, 1372, 1311, 1275, 1227, 1195, 1182, 1120 ,1104, 1016, 997, 966, 934, 900, 837, 807, 766, 691, 642, 555, 513, 451, 425. **HRMS** (ESI-orbitrap): *m/z* calcd for $C_{11}H_{11}BrO_5Na^+$: 324.9682 $[M]^+$; found: 324.9677.

2-bromo-5-methoxyterephthalic acid (15)

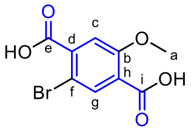

1-bromo-4-methoxy-2,5-dimethylbenzene (5.10 g, 23.7 mmol) and sodium hydroxide (1.8 eq, 1.71 g, 42.7 mmol) were dissolved in a mixture of *tert*-butanol/ H_2O (1:1, 350 mL). The reaction mixture was heated to 100 °C while stirring, and a first portion (15 g) of $KMnO_4$ (total: 8 eq, 30 g, 190 mmol) was added to the solution. After stirring for 8 h a second portion (15 g) of $KMnO_4$ was added and the deep purple solution kept at 100 °C for another 18 h. The excess of $KMnO_4$ was reduced by addition of sodium thiosulfate pentahydrate (2.5 g) and the hot solution immediately filtered over a Celite® pad. The resulting solid was washed with boiling H_2O (600 mL). The aqueous filtrate was concentrated to eliminate the *tert*-butanol and acidified to pH = 1 with conc. HCl. The white precipitate formed was further collected by filtration, washed with cold H_2O (1 L) and dried in an oven at 100 °C for 18 h to give **15** (yield = 47%, 3.09 g) as a white solid. **M.P.** 262.8 – 265.0 °C. **^{1}H NMR** (700 MHz, DMSO-*d*6) δ 13.44 (br s, 2H, COOH), 7.83 (s, 1H, H_g), 7.41 (s, 1H, H_c), 3.85 (s, 3H, H_a).**^{13}C NMR** (176 MHz, DMSO-*d6*) δ 166.86 (C_i), 165.50 (C_e), 156.89 (C_b), 137.51 (C_d), 134.73 (C_g), 124.97 (C_h), 114.23 (C_c), 109.16 (C_f), 56.31 (C_a). **IR** (neat): 2973, 2849, 1694, 1602, 1550, 1487, 1454, 1413, 1364, 1297, 1242, 1184, 1103, 1030, 948, 912, 873, 791, 781, 753, 662, 483, 446, 434, 423, 411. **HRMS** (ESI-orbitrap): *m/z* calcd for $C_9H_6BrO_5^-$: 272.9404 $[M]^-$; found: 272.9402.

dimethyl 2-bromo-5-methoxyterephthalate (16)

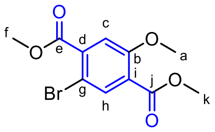

5 drops of conc. H_2SO_4 were added to a solution of **15** (1.00 g, 3.65 mmol) in dry MeOH (8 mL) in a 25 mL round-bottomed flask. The reaction mixture was heated at 80 °C for 18 h. The mixture was allowed to cool to room temperature and poured into H_2O (20 mL), basified with a solution 25% $NH_4OH_{(aq)}$ (pH ≃ 10) and extracted with Et_2O (3 × 50 mL). The combined organic layers were dried over $MgSO_4$, and the solvent removed in *vacuo* to give **16** (yield = *quant.* 1.11 g) as a white solid. **M.P.** 97.0 – 98.8 °C. **^{1}H NMR** (700 MHz, $CDCl_3$) δ 8.02 (s, 1H, H_h), 7.36 (s, 1H, H_c), 3.95 (s, 3H, H_k), 3.92 (s, 3H, H_a), 3.90 (s, 3H, H_f).**^{13}C NMR** (176 MHz, $CDCl_3$) δ 166.04 (C_j), 164.76 (C_e), 157.86 (C_b), 136.95 (C_h), 136.11 (C_d), 124.01 (C_i), 114.87 (C_c), 111.37 (C_g), 56.57 (C_a), 52.92 (C_k), 52.64 (C_f). **IR** (neat): 2958, 2844, 1696, 1602, 1547, 1482, 1445, 1433, 1371, 1296, 1232, 1190, 1171, 1133, 1103, 1033, 985, 960, 923, 905, 874, 826, 787, 776, 690, 655, 604, 586, 524, 446.**HRMS** (ESI-timsTOF): *m/z* calcd for $C_{11}H_{11}BrO_5Na^+$: 324.9682 $[M]^+$; found: 324.9683.

methyl 2-(2,8-bis(2,6-dimethylphenyl)xantheno[2,1,9,8-klmna]xanthen-4-yl)benzoate (17)

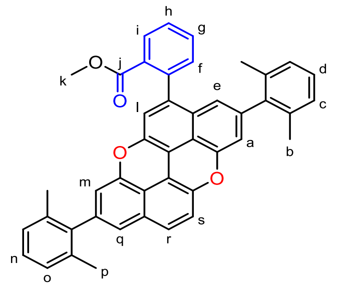

Procedure adapted from literature.[13]
To a 50 mL round-bottomed flask, **9** (154 mg, 0.25 mmol), methyl 2-bromobenzoate (1.5 eq, 81 mg, 0.375 mmol), [Pd(dppf)Cl_2] (0.2 eq, 37 mg, 0.05 mmol), Kolliphor (1 µL) and a mixture H_2O /toluene (9:1, 5 mL) were added. The reaction mixture was degassed bubbling Argon in a sonicator for 20 min. To the resulting yellow mixture was added Et_3N (3 eq, 105 µL, 0.75 mmol) and the mixture was heated at 60 °C for 16 h. After letting the reaction mixture to cooldown to room temperature, it was poured into CH_2Cl_2 (50 mL) and filter through a pad of SiO_2/ Celite® (1:1 $_{w/w}$). The solution was then washed with brine (3 × 50 mL) and finally the organic layer was dried over $MgSO_4$, filtered and evaporated in *vacuo*. The crude material was chromatographed (SiO_2, CH_2Cl_2/heptane 1:1) to give **17** (yield = 70%, 110 mg) as a yellow-orange solid. **M.P.** (dec) 260 °C. **^{1}H NMR** (700 MHz, CD_2Cl_2) δ 7.98 (dd, J = 7.9, 1.1 Hz, 1H, H_f), 7.60 (m, 1H, H_g), 7.46 (m, 1H, H_h), 7.40 – 7.33 (m, 2H, H_i, H_r), 7.20 – 7.15 (m, 1H, H_d), 7.14 – 7.11 (m, 2H, H_c), 7.10 (d, J = 7.6 Hz, 1H, H_n), 7.07 – 7.03 (m, 2H, H_o), 7.02 (d, J = 8.9 Hz, 1H, H_s), 6.94 (d, J = 1.0 Hz, 1H, H_q), 6.87 (s, 1H, H_e), 6.54 (m, 2H, H_a, H_m), 6.46 (d, J = 1.1 Hz, 1H, H_l), 3.57 (s, 3H, H_k), 2.15 (s, 3H, H_b or $H_{b'}$), 2.14 (s, 3H H_b or $H_{b'}$), 2.06 (s, 3H H_p or $H_{p'}$), 2.03 (s, 3H H_p or $H_{p'}$). **^{13}C NMR** (176 MHz, CD_2Cl_2) δ 167.53 (C_j), 153.36, 153.26, 144.55, 144.30, 141.77, 140.93, 140.73, 140.53, 140.03, 136.39, 136.33, 136.30, 136.21, 132.63 (C_g), 131.90, 131.69 (C_i), 131.32, 131.05, 130.81 (C_f), 128.37 (C_h), (127.71, 127.70, 127.63, 127.59, 127.52 (C_c, C_d, C_n, C_o)), 126.76 (C_r), 120.76, 120.59 (C_q), 120.45, 118.79 (C_l), 117.82 (C_s), 117.74 (C_e), 111.97, 111.13, 110.89 (C_a or C_m), 110.86 (C_a or C_m), 52.13 (C_k), 20.90 (C_b or $C_{b'}$), 20.88 (C_b or $C_{b'}$), 20.84 (C_p or $C_{p'}$), 20.77(C_p or $C_{p'}$). **IR** (neat): 2920, 2850, 1731, 1697, 1603, 1462, 1433, 1390, 1294, 1274, 1228, 1188, 1154, 1114, 1090, 1053, 1043, 1011, 968, 916, 870, 780, 766, 698. **HRMS** (LD-timsTOF): *m/z* calcd for $C_{44}H_{32}O_4^+$: 624.2295 [M]$^+$; found: 624.2282.

dimethyl 2,2'-(2,8-bis(2,6-dimethylphenyl)xantheno[2,1,9,8-klmna]xanthene-4,10-diyl)dibenzoate (18)

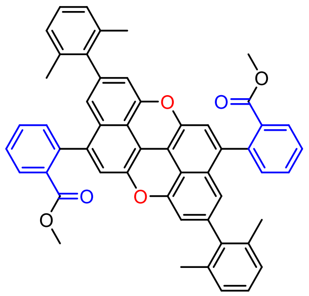

To a 50 mL round-bottomed flask, **10** (186 mg, 0.25 mmol), methyl 2-bromobenzoate (2.5 eq, 134 mg, 0.625 mmol), [Pd(dppf)Cl_2] (0.4 eq, 65 mg, 0.1 mmol), Kolliphor (1 µL) and a mixture H_2O /toluene (9:1, 6 mL) were added. The reaction mixture was degassed bubbling Argon in a sonicator for 20 min. To the resulting yellow mixture was added Et_3N (6 eq, 209 µL, 1.5 mmol) and the mixture was heated at 60 °C for 16 h. After letting the reaction mixture to cooldown to room temperature, it was poured into CH_2Cl_2 (75 mL) and filter through a pad of SiO_2/ Celite® (1:1 $_{w/w}$). The solution was then washed with brine (3 × 50 mL) and finally the organic layer was dried over $MgSO_4$, filtered and

evaporated in *vacuo*. The crude material was chromatographed (SiO_2, CH_2Cl_2/heptane 1:1) to give **18** (mixture of atropoisomers, yield = 60%, 113 mg) as a yellow-orange solid. **M.P.** > 300 °C. **^{1}H NMR** (700 MHz, CD_2Cl_2) δ 8.61 – 7.89 (m, 2H), 7.71 – 6.38 (m, 18H), 3.66 – 3.41 (m, *J* = 1.2 Hz, 6H), 2.03 (m, 12H).**^{13}C NMR** (176 MHz, CD_2Cl_2) δ 167.65, 167.59, 153.40, 144.16, 141.79, 140.73, 140.54, 140.49, 139.94, 136.34, 136.29, 136.26, 136.19, 132.63, 131.74, 131.68, 131.41, 131.36, 131.05, 130.82, 130.80, 129.77, 128.48, 128.37, 127.62, 127.60, 127.57, 127.56, 127.50, 126.53, 125.80, 120.44, 118.79, 117.63, 111.21, 110.87, 52.17, 52.14, 20.81, 20.80, 20.75, 20.73. **IR** (neat): 2920, 2850, 1731 ,1697, 1603, 1462, 1433, 1390, 1294, 1274, 1228, 1188, 1154, 1114, 1090, 1053, 1043, 1011, 968, 916, 870, 780, 766, 698. **HRMS** (LD-timsTOF): *m/z* calcd for $C_{52}H_{38}O_6^+$: 758.2663 $[M]^+$; found: 758.2655.

dimethyl 4,6-bis(2,8-bis(2,6-dimethylphenyl)xantheno[2,1,9,8-klmna]xanthen-4-yl)isophthalate (19)

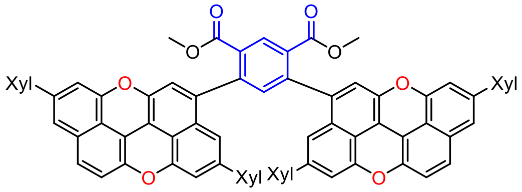

To a 50 mL round-bottomed flask, **9** (2 eq, 123 mg, 0.2 mmol), **11** (1 eq, 35 mg, 0.1 mmol), [Pd(dppf)Cl_2] (0.4 eq, 29 mg, 0.04 mmol), Kolliphor (1 µL) and a mixture H_2O /toluene (9:1, 4 mL) were added. The reaction mixture was degassed bubbling argon in a sonicator for 20 min. To the resulting yellow mixture was added Et_3N (6 eq, 84 µL, 0.6 mmol) and the mixture was heated at 60 °C for 16 h. After letting the reaction mixture to cooldown to room temperature, it was poured into CH_2Cl_2 (40 mL) and filter through a pad of SiO_2/ Celite® (1:1 $_{w/w}$). The solution was then washed with brine (3 × 40 mL) and finally the organic layer was dried over $MgSO_4$, filtered and evaporated in *vacuo*. The crude material was chromatographed (SiO_2, CH_2Cl_2/heptane 1:1) to give **19** (mixture of atropoisomers, yield = 80%, 93 mg) as an orange solid. **M.P.** 256.2 – 257.7 °C. **^{1}H NMR** (600 MHz, CD_2Cl_2) δ 8.56 (d, *J* = 7.7 Hz, 1H), 7.44 – 7.36 (m, 3H), 7.18 – 6.82 (m, 18H), 6.57 – 6.49 (m, 5H), 6.44 – 6.41 (m, 1H), 3.61 – 3.57 (m, 6H), 2.11 (s, 12H), 2.08 – 1.76 (m, 12H). **^{13}C NMR** (151 MHz, CD_2Cl_2) δ 166.35, 166.33, 153.37, 153.29, 153.18, 144.63, 144.62, 144.58, 144.40, 144.27, 144.25, 141.73, 141.72, 141.63, 141.48, 141.10, 141.08, 140.97, 140.96, 138.47, 138.30, 136.41, 136.39, 136.32, 136.28, 136.24, 136.18, 136.10, 135.13, 134.92, 133.40, 133.28, 131.88, 130.98, 130.91, 130.62, 127.69, 127.68, 127.66, 127.63, 127.62, 127.59, 127.48, 127.00, 120.74, 120.73, 120.65, 120.46, 120.35, 118.51, 118.40, 117.81, 117.74, 117.67, 111.78, 111.77, 111.75, 111.09, 111.05, 110.98, 110.96, 52.44, 20.92, 20.86, 20.84, 20.76, 20.74, 20.71. **IR** (neat): 2948, 2915, 2367, 2319, 2234, 2109, 2091, 2072, 1923, 1731, 1629, 1595, 1539, 1496, 1469, 1432, 1403, 1377, 1347, 1314, 1274, 1228, 1201, 1159, 1098, 1076, 1041, 1004, 954, 922, 857, 821, 796, 767, 727, 681, 653, 629, 610, 588, 569, 534, 524, 504, 487, 464, 448, 412. **HRMS** (MALDI-timsTOF): *m/z* calcd for $C_{82}H_{58}O_8^+$: 1170.4126 $[M]^+$; found: 1170.4142.

dimethyl 2,5-bis(2,8-bis(2,6-dimethylphenyl)xantheno[2,1,9,8-klmna]xanthen-4-yl)terephthalate (20)

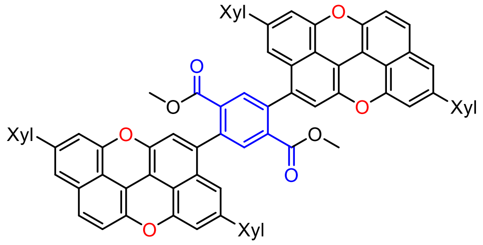

To a 50 mL round-bottomed flask, **9** (2 eq, 123 mg, 0.2 mmol), dimethyl 2,5-dibromoterephthalate (35 mg, 0.1 mmol), [Pd(dppf)Cl_2] (0.4 eq, 29 mg, 0.04 mmol), Kolliphor (1 µL) and a mixture H_2O /toluene (9:1, 4 mL) were added. The reaction mixture was degassed bubbling Argon in a sonicator for 20 min. To the resulting yellow mixture was added Et_3N (6 eq, 84 µL, 0.6 mmol) and the mixture was heated at 60 °C for 16 h. After letting the reaction mixture to cooldown to room temperature, it was poured into CH_2Cl_2 (50 mL) and filter through a pad of SiO_2/ Celite® (1:1 $_{w/w}$). The solution was then washed with brine (3 × 40 mL) and finally the organic layer was dried over $MgSO_4$, filtered and evaporated in *vacuo*. The crude material was chromatographed (SiO_2, CH_2Cl_2/heptane 1:1) to give **20** (mixture of atropoisomers, yield = 64%, 75 mg) as an orange solid. **M.P.** > 300 °C. **^{1}H NMR** (700 MHz, CD_2Cl_2) δ 8.01 – 7.94 (m, 2H), 7.39 (d, *J* = 8.9 Hz, 2H), 7.19 – 7.00 (m, 14H), 6.97 – 6.88 (m, 4H), 6.65 – 6.51 (m, 6H), 3.58 – 3.55 (m, 6H), 2.14 – 2.11 (m, 12H), 2.08 – 1.97 (m, 12H).**^{13}C NMR** (176 MHz, CD_2Cl_2) δ 166.61, 166.59, 153.42, 153.31, 153.21, 153.19, 144.64, 144.62, 144.33, 144.28, 141.75, 141.74, 141.73, 141.59, 140.98, 140.98, 140.93, 140.20, 139.96, 138.38, 138.32, 136.39, 136.36, 136.35, 136.30, 136.24, 134.52, 134.46, 134.00, 133.97, 131.90, 130.87, 127.69, 127.65, 127.63, 127.62, 127.59, 127.53, 126.98, 126.95, 120.76, 120.66, 120.61, 120.55, 120.51, 118.81, 118.55, 118.42, 118.12, 117.81, 111.85, 111.80, 111.71, 111.64, 111.18, 110.98, 52.48, 52.47, 20.87, 20.85, 20.83, 20.80, 20.79, 20.77. **IR** (neat): 2915, 2358, 2191, 2170, 2142, 2120, 2105, 2061, 2020, 1734, 1721, 1630, 1598, 1582, 1495, 1470, 1440, 1403, 1376, 1337, 1315, 1276, 1244, 1203, 1161, 1123, 1101, 1081, 1043, 988, 970, 955, 923, 879, 857, 799, 768, 726, 698, 688, 676, 637, 568, 554, 536, 525, 503, 494, 475, 463, 449, 433, 417, 407. **HRMS** (MALDI-timsTOF): *m/z* calcd for $C_{82}H_{58}O_8^+$: 1170.4126 $[M]^+$; found: 1170.4142.

tetramethyl 6,6'-(2,8-bis(2,6-dimethylphenyl)xantheno[2,1,9,8-klmna]xanthene-4,10-diyl)bis(4-methoxyisophthalate) (21)

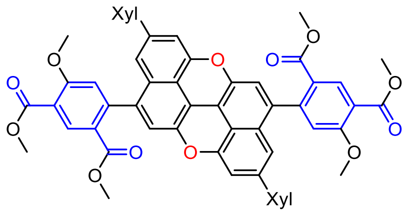

To a 50 mL round-bottomed flask, **10** (156 mg, 0.21 mmol), **14** (2.1 eq, 134 mg, 0.441 mmol), K_3PO_4 (4 eq, 178 mg, 0.84 mmol) and a mixture dioxane/ H_2O (5:1, 4.8 mL) were added. The reaction mixture was degassed bubbling Argon in a sonicator for 20 min. To the resulting yellow mixture were added [$Pd_2(dba)_3$] (0.2 eq, 39 mg, 0.042 mmol) and tricyclohexylphosphine (0.4 eq, 24 mg, 0.042 mmol) and the mixture was heated at 105 °C for 18 h. After letting the reaction mixture to cool down to room temperature, it was diluted with CH_2Cl_2 (50 mL) and washed with brine (3 × 50 mL). The combined H_2O layers were extracted with CH_2Cl_2 (50 mL). Finally, the combined organic layers were dried over $MgSO_4$, filtered through a pad of Celite® and evaporated in *vacuo*. The crude material

was chromatographed (SiO_2, CH_2Cl_2/heptane 7:3) to give **21** (mixture of atropoisomers, yield = 74%, 145 mg) as an orange solid. **M.P.** > 300 °C. **^{1}H NMR** (600 MHz, CD_2Cl_2) δ 8.44 (s, 2H), 7.13 – 6.89 (m, 10H), 6.51 (m, 4H), 3.92 (m, 6H), 3.87 (s, 6H), 3.58 (m, 6H), 2.06 – 1.98 (m, 12H). **^{13}C NMR** (151 MHz, CD_2Cl_2) δ 166.13, 166.00, 165.71, 165.69, 161.97, 141.59, 136.32, 136.25, 136.15, 136.02, 135.14, 135.10, 127.70, 127.67, 127.63, 127.60, 120.01, 118.70, 117.27, 115.45, 115.32, 111.51, 111.09, 56.77, 56.72, 52.47, 52.12, 52.08, 20.84, 20.83, 20.74, 20.72. **IR** (neat): 2920, 2850, 1731, 1697, 1603, 1462, 1433, 1390, 1294, 1274, 1228, 1188, 1154, 1114, 1090, 1053, 1043, 1011, 968, 916, 870, 780, 766, 698.**HRMS** (MALDI-timsTOF): *m/z* calcd for $C_{58}H_{46}O_{12}^+$: 934.2984 $[M]^+$; found: 934.2981.

tetramethyl 6,6'-(2,8-bis(2,6-dimethylphenyl)xantheno[2,1,9,8-klmna]xanthene-4,10-diyl)bis(4-(((trifluoromethyl)sulfonyl)oxy)isophthalate) (22)

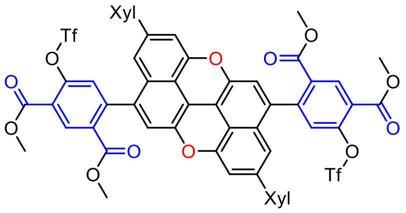

To a 50 mL round-bottomed flask, **21** (131 mg, 0.14 mmol), anhydrous $AlCl_3$ (12 eq, 224 mg, 1.68 mmol), and dry CH_2Cl_2 (4 mL) were added. The reaction mixture was degassed bubbling Argon in a sonicator for 20 min. To the resulting dark green mixture was added dropwise 1-dodecanthiol (6 eq, 202 µL, 0.84 mmol) and the mixture was kept at room temperature. The reaction was monitored by TLC ($AlCl_3$ was quenched by methanol). After reaction time (ca. 1 h), the reaction mixture was poured into H_2O (25 mL), acidified with $HCl_{(aq)}$ (1 M) and extracted with CH_2Cl_2 (2 × 25 mL) The combined organic layers were dried over $MgSO_4$, filtered through a pad of Celite® and evaporated in *vacuo*. The crude material was chromatographed (SiO_2, CH_2Cl_2/THF 99:1) to give the corresponding bis-phenol **21-OH** (not isolated and not characterized due to the low solubility in common organic solvents) as a yellow solid. To an Argon-filled 25 mL round-bottomed flask, **21-OH** (131 mg, 0.14 mmol*), Et_3N (4 eq, 78 µL, 0.56 mmol), and dry CH_2Cl_2 (4 mL) were added. The reaction mixture was degassed bubbling Argon in a sonicator for 20 min. To the resulting yellow mixture was added dropwise $(CF_3SO_2)_2O$ (2.4 eq, 56 µL, 0.336 mmol) and the mixture kept at room temperature for 2 h. Concentration followed by column chromatography (SiO_2, CH_2Cl_2/heptane 8:2) afforded **21** (mixture of atropoisomers, yield = 55%, over 2 steps, 63 mg) as a red solid. **M.P.** (dec) 150 °C. **^{1}H NMR** (400 MHz, CD_2Cl_2) δ 8.58 (s, 2H), 7.28 (s, 2H), 7.05 – 6.99 (m, 2H), 6.99 – 6.93 (m, 4H), 6.83 (s, 2H), 6.51 (s, 2H), 6.32 (s, 2H), 3.89 (s, 6H), 3.57 – 3.52 (m, 6H), 1.97 – 1.94 (m, 6H), 1.93 – 1.90 (m, 6H). (the compound degrades faster than the acquisition of the ^{13}C NMR in CD_2Cl_2) **^{19}F NMR** (565 MHz, CD_2Cl_2) δ -73.58, -73.59. **^{1}H NMR** (700 MHz, C_6D_6) δ 8.59 (d, J = 4.6 Hz, 2H), 7.07 – 7.01 (m, 5H), 7.00 – 6.92 (m, 3H), 6.67 – 6.60 (m, 6H), 3.51 – 3.46 (m, 6H), 3.21 – 3.15 (m, 6H), 2.14 – 2.06 (m, 12H). **^{13}C NMR** (176 MHz, C_6D_6) δ 164.71, 164.67, 162.93, 162.89, 153.43, 150.19, 150.14, 146.68, 146.56, 144.40, 144.28, 142.15, 142.02, 141.34, 141.29, 136.95, 136.71, 135.85, 135.84, 135.81, 135.76, 135.65, 131.43, 131.39, 130.46, 130.32, 128.43, 128.36, 126.12, 126.05, 124.18, 124.12, 120.74, 120.69, 118.11, 118.01, 117.99, 117.91, 112.39, 112.28, 111.71, 111.51, 52.40, 51.98, 51.97, 20.84, 20.77, 20.71, 20.71. **^{19}F NMR** (659 MHz, C_6D_6) δ -73.71, -73.72. **IR** (neat): 2955, 2213, 2166, 2088, 2057, 2018, 1734, 1612, 1429, 1395, 1295, 1245, 1210, 1138, 1103, 1029, 970, 941, 921, 857, 829, 811, 783, 756, 684, 606, 573, 526, 506, 485, 470, 456, 428, 415. **HRMS** (MALDI-timsTOF): *m/z* calcd for $C_{58}H_{40}O_{16}F_6S_2^+$: 1170.1656 $[M]^+$; found: 1170.1660.

* Assuming quantitative the first deprotection.

tetramethyl 6,6'-(2,8-bis(2,6-dimethylphenyl)xantheno[2,1,9,8-klmna]xanthene-4,10-diyl)bis(4-(2,8-bis(2,6-dimethylphenyl)xantheno[2,1,9,8-klmna]xanthen-4-yl)isophthalate) (23)

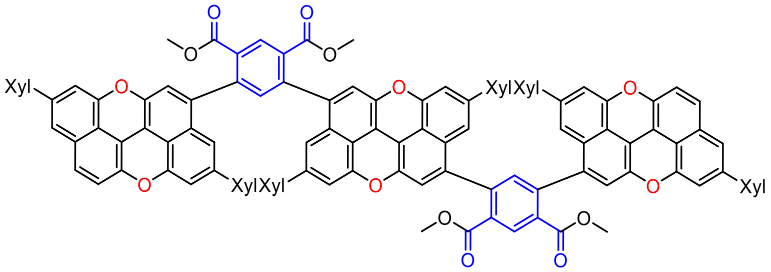

To a 25 mL round-bottomed flask, **22** (23 mg, 0.025 mmol), **9** (3 eq, 46 mg, 0.075 mmol), CsF (5 eq, 19 mg, 0.125 mmol) and a mixture dioxane/ H_2O (2:1, 1.5 mL) were added. The reaction mixture was degassed bubbling Argon in a sonicator for 20 min. To the resulting orange mixture was added [Pd(dppf)Cl_2] (0.1 eq, 2 mg, 0.0025 mmol) and the mixture was heated at 120 °C for 18 h. After letting the reaction mixture to cool down to room temperature, it was diluted with CH_2Cl_2 (50 mL) and washed with brine (3 × 50 mL). The combined H_2O layers were extracted with CH_2Cl_2 (50 mL). Finally, the combined organic layers were dried over $MgSO_4$, filtered through a pad of Celite® and evaporated in *vacuo*. The crude material was chromatographed (SiO_2, CH_2Cl_2/heptane 9:1) to give **23** (mixture of atropoisomers, yield = 43%, 20 mg) as an orange solid. **M.P.** > 300 °C. **^{1}H NMR** (600 MHz, CD_2Cl_2/CS_2 5:1) δ 8.69 – 6.34 (m, 42H), 3.82 – 3.57 (m, 12H), 2.28 – 1.76 (m, 36H). ^{13}C NMR could not be recorded due to insufficient solubility of product. **IR** (neat): 2942, 2922, 2852, 2363, 2339, 2293, 2222, 2204, 2193, 2058, 2009, 1731, 1630, 1597, 1497, 1470, 1434, 1398, 1349, 1310, 1275, 1235, 1206, 1190, 1156, 1104, 1075, 1044, 1015, 956, 917, 860, 768, 741, 717, 670, 634, 610, 587, 567, 541, 529, 504, 487, 464, 443, 431, 417, 406. **HRMS** (MALDI-timsTOF): *m/z* calcd for $C_{128}H_{90}O_{14}^+$: 1851.6359 [M]$^+$; found: 1851.6417.

dimethyl 2-(2,8-bis(2,6-dimethylphenyl)xantheno[2,1,9,8-klmna]xanthen-4-yl)-5-methoxyterephthalate (24)

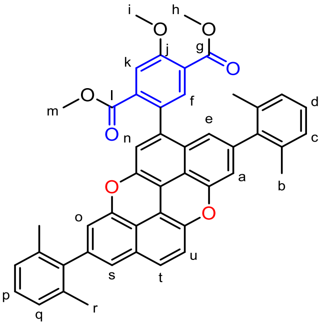

To a 50 mL round-bottomed flask, **9** (154 mg, 0.25 mmol), **16** (1.1 eq, 83 mg, 0.25 mmol), K_3PO_4 (2 eq, 106 mg, 0.5 mmol) and a mixture dioxane/ H_2O (5:1, 6 mL) were added. The reaction mixture was degassed bubbling argon in a sonicator for 20 min. To the resulting yellow-orange mixture were added [$Pd_2(dba)_3$] (0.5 eq, 114 mg, 0.125 mmol) and tricyclohexylphosphine (1 eq, 70 mg, 0.25 mmol) and the mixture was heated at 105 °C for 18 h. After letting the reaction mixture to cooldown to room temperature, it was diluted with CH_2Cl_2 (40 mL) and washed with brine (3 × 40 mL). The combined H_2O layers were extracted with CH_2Cl_2 (40 mL). Finally, the combined organic layers were dried over $MgSO_4$, filtered through a pad of Celite® and evaporated in *vacuo*. The crude material was chromatographed (SiO_2, CH_2Cl_2/heptane 6:4) to give **24** (yield = 79%, 140 mg) as an orange solid. **M.P.** (dec) 280 °C. **^{1}H NMR** (600 MHz, CD_2Cl_2) δ 7.65 (s, 1H, H_f), 7.47 (s, 1H, H_k), 7.30 (d, J = 9.0 Hz, 1H, H_t), 7.10 – 7.05 (m, 1H, H_d), 7.05 – 7.00 (m, 3H, H_c, H_p), 6.98 – 6.92 (m, 3H, H_q,

H_u), 6.85 (s, 1H, H_s), 6.79 (s, 1H, H_e), 6.47 – 6.42 (m, 2H, H_a, H_o), 6.35 (d, J = 0.6 Hz, 1H, H_n), 3.86 (s, 3H, H_h), 3.75 (s, 3H, H_m), 3.47 (s, 3H, H_i), 2.05 – 2.02 (m, 6H, H_b), 1.97 – 1.89 (m, 6H, H_r). **^{13}C NMR** (151 MHz, CD_2Cl_2) δ 166.94 (C_g), 165.89 (C_l), 158.56 (C_j), 153.38, 153.23, 144.57, 144.29, 141.75, 141.73, 140.95, 140.84, 138.63, 136.38, 136.36, 136.32, 136.27, 135.39, 134.70 (C_f), 132.00, 131.90, 131.20, (127.69, 127.63, 127.58, 127.55 (C_c, C_d, C_p, C_q)), 126.84 (C_t), 123.92, 120.75, 120.62 (C_s), 120.46, 118.65 (C_n), 118.12 (C_e), 117.81 (C_u), 114.32 (C_k), 111.91, 111.30, 110.92 (C_a or C_o), 110.90 (C_a or C_o), 56.71 (C_i), 52.51 (C_h or C_m), 52.49 (C_h or C_m), 20.87 (C_b or $C_{b'}$), 20.85 (C_b or $C_{b'}$), 20.79 (C_r or $C_{r'}$), 20.76 (C_r or $C_{r'}$). **IR** (neat): 2920, 2849, 1730, 1629, 1597, 1582, 1495, 1460, 1432, 1403, 1379, 1347, 1315, 1301, 1273, 1224, 1201, 1161, 1098, 1082, 1065, 1039, 1003, 968, 953, 917, 857, 795, 767, 696, 631, 568, 549, 533, 505, 486, 480, 466, 451, 438, 429, 420. **HRMS** (LD-timsTOF): *m/z* calcd for $C_{47}H_{36}O_7^+$: 934.2984 $[M]^+$; found: 934.2981.

dimethyl 2-(2,8-bis(2,6-dimethylphenyl)xantheno[2,1,9,8-klmna]xanthen-4-yl)-5-(((trifluoromethyl)sulfonyl)oxy)terephthalate (25)

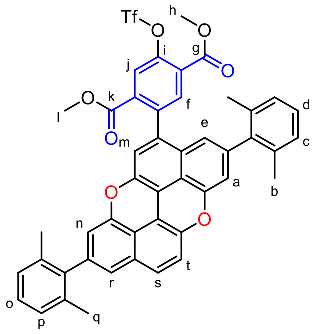

To a 50 mL round-bottomed flask, **24** (143 mg, 0.2 mmol), anhydrous $AlCl_3$ (6 eq, 160 mg, 1.2 mmol), and dry CH_2Cl_2 (6 mL) were added. The reaction mixture was degassed bubbling Argon in a sonicator for 20 min. To the resulting dark green mixture was added dropwise 1-dodecanthiol (3 eq, 145 µL, 0.6 mmol) and the mixture was kept at room temperature. The reaction was monitored by TLC ($AlCl_3$ was quenched by methanol before spotting on TLC). After reaction time (ca. 10 min), the reaction mixture was poured into H_2O (25 mL), acidified with $HCl_{(aq)}$ (1 M) and extracted with CH_2Cl_2 (2 × 25 mL) The combined organic layers were dried over $MgSO_4$, filtered through a pad of Celite® and evaporated in *vacuo*. The crude material was chromatographed (SiO_2, CH_2Cl_2/THF 99:1) to give the corresponding phenol (**24-OH**) (not isolated and not characterized) as a yellow solid. To an Argon-filled 25 mL round-bottomed flask, **24-OH** (140 mg, 0.2 mmol*), Et_3N (2 eq, 56 µL, 0.4 mmol), and dry CH_2Cl_2 (8 mL) were added. The reaction mixture was degassed bubbling Argon in a sonicator for 20 min. To the resulting yellow mixture was added dropwise $(CF_3SO_2)_2O$ (1.2 eq, 40 µL, 0.24 mmol) and the mixture kept at room temperature for 2 h. Concentration followed by column chromatography (SiO_2, CH_2Cl_2/heptane 8:2) afforded **25** (yield = 97%, over 2 steps, 161 mg) as a red solid. **M.P.** (dec) 154 °C. **^{1}H NMR** (600 MHz, CD_2Cl_2) δ 8.11 (s, 1H, H_f), 7.89 (s, 1H, H_j), 7.41 (d, J = 9.0 Hz, 1H, H_s), 7.19 – 7.15 (m, 1H, H_d), 7.14 – 7.10 (m, 3H, H_c, H_o), 7.06 – 7.02 (m, 3H, H_p, H_t), 6.95 (d, J = 0.8 Hz, 1H, H_r), 6.87 (s, 1H, H_e), 6.58 (d, J = 0.8 Hz, 1H, H_n), 6.55 (d, J = 0.9 Hz, 1H, H_a), 6.42 (d, J = 0.9 Hz, 1H, H_m), 3.95 (s, 3H, H_h), 3.62 (s, 3H, H_l), 2.16 – 2.10 (m, 6H, H_b), 2.08 – 2.03 (m, 6H, H_q). **^{13}C NMR** (151 MHz, CD_2Cl_2) δ 165.18 (C_g), 163.55 (C_k), 153.45, 153.10, 147.63 (C_i), 144.72, 144.21, 141.67, 141.50, 141.43, 141.07, 140.72, 136.70, 136.45, 136.36, 136.33, 136.27, 136.24 (C_f), 136.21, 131.90, 130.51, (127.71, 127.68, 127.66, 127.63 (C_c, C_d, C_o, C_p)), 127.27 (C_s), 125.31, 120.77, 120.74, 120.56, 120.17, 118.32 (C_e), 118.10 (C_m), 118.05, 117.84 (C_t), 112.30, 111.60, 111.26 (C_n), 111.08 (C_a), 53.31 (C_h), 53.02 (C_l), 20.88 (C_b or $C_{b'}$), 20.85 (C_b or $C_{b'}$), 20.81 (C_q or $C_{q'}$), 20.75 (C_q or $C_{q'}$). **^{19}F NMR** (565 MHz, CD_2Cl_2) δ -73.68. **IR** (neat): 2954, 1736, 1630, 1597, 1583, 1492, 1470, 1428, 1405, 1378, 1345, 1315, 1275, 1243, 1204, 1154, 1138, 1103, 1081,

1054, 1013, 967, 954, 928, 892, 865, 827, 795, 768, 746, 667, 648, 598, 567, 542, 504, 448, 418. **HRMS** (MALDI-timsTOF): *m/z* calcd for $C_{47}H_{33}O_9F_3S^+$: 830.1792 [M]$^+$; found: 830.1796.
* Assuming quantitative the first deprotection.

tetramethyl 5,5'-(2,8-bis(2,6-dimethylphenyl)xantheno[2,1,9,8-klmna]xanthene-4,10-diyl)bis(2-(2,8-bis(2,6-dimethylphenyl)xantheno[2,1,9,8-klmna]xanthen-4-yl)terephthalate) (26)

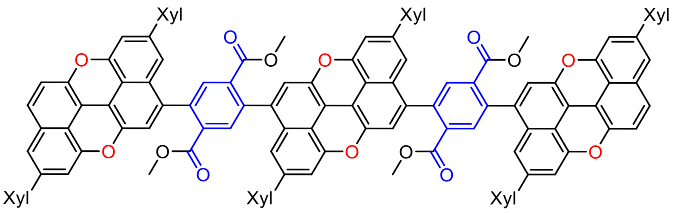

To a 25 mL round-bottomed flask, **25** (2.2 eq, 165 mg, 0.198 mmol), **10** (67 mg, 0.09 mmol), CsF (5 eq, 68 mg, 0.45 mmol) and a mixture dioxane/ H_2O (2:1, 6 mL) were added. The reaction mixture was degassed bubbling argon in a sonicator for 20 min. To the resulting orange mixture was added [Pd(dppf)Cl_2] (0.1 eq, 7 mg, 0.009 mmol) and the mixture was heated at 120 °C for 18 h. After letting the reaction mixture cool down to room temperature, it was diluted with CH_2Cl_2 (50 mL) and washed with brine (3 × 50 mL). The combined H_2O layers were extracted with CH_2Cl_2 (50 mL). Finally, the combined organic layers were dried over $MgSO_4$, filtered through a pad of Celite® and evaporated in *vacuo*. The crude material was chromatographed (SiO_2, CH_2Cl_2/heptane 9:1) to give **26** (mixture of atropoisomers, yield = 58%, 96 mg) as an orange solid. **M.P.** > 300 °C. **^{1}H NMR** (600 MHz, CD_2Cl_2) δ 8.11 – 7.93 (m, 4H), 7.65 – 6.21 (m, 38H), 3.63 – 3.51 (m, *J* = 4.8 Hz, 12H), 2.14 – 1.97 (m, 36H). ^{13}C NMR could not be recorded due to insufficient solubility of product. **IR** (neat): 2951, 2919, 2121, 1720, 1630, 1599, 1582, 1497, 1470, 1434, 1394, 1381, 1336, 1315, 1272, 1248, 1232, 1218, 1205, 1159, 1121, 1081, 1043, 988, 978, 955, 940, 923, 873, 860, 823, 812, 796, 769, 749, 727, 697, 673, 643, 599, 567, 550, 538, 504, 493, 468, 457, 446, 413.**HRMS** (MALDI-timsTOF): *m/z* calcd for $C_{128}H_{90}O_{14}^+$: 1851.6359 [M]$^+$; found: 1851.6366.

General procedure for the intramolecular Friedel-Crafts reaction (a)
To a round-bottomed flask, PXX-ester derivative (1 eq), NaOH (5 eq per ester group), and NMP were added. To the resulting mixture was added dropwise 1-dodecanthiol (2.5 eq per ester group) and the mixture was heated to 110 °C. The reaction was monitored by TLC. After reaction time (ca. 4 h), the reaction mixture was poured into H_2O, acidified with $HCl_{(aq)}$ (1 M) and extracted with CH_2Cl_2. The combined organic layers were dried over $MgSO_4$, filtered through a pad of Celite® and evaporated in *vacuo*. The crude material was chromatographed (SiO_2, CH_2Cl_2/THF) to give the corresponding acid (not isolated and not characterized). To an Argon-filled round-bottomed flask, the collected acid (1 eq*) and Eatons's reagent (ER) were added. The reaction mixture was sonicated for 20 min, allowing the acid to disperse in the ER. The resulting mixture was heated at 70 °C (in a pre-heated oil bath) for 1 to 2 h (monitored by mass analysis, MALDI. An aliquot of reaction mixture was quenched with sat. $NaHCO_{3(aq)}$ before being extracted with CH_2Cl_2). After letting the reaction mixture to cooldown to room temperature, it was poured into crushed ice and quenched slowly with $NaOH_{(aq)}$ (50%$_{w/w}$). The formed H_2O phase was extracted with CH_2Cl_2. Finally, the combined organic layers were dried over $MgSO_4$, filtered through a pad of Celite® and evaporated in *vacuo*.
*Assuming quantitative the first deprotection.

4,13-bis(2,6-dimethylphenyl)-12H-anthra[9,1,2-hij]benzo[8,1]isochromeno[5,4,3-cde]isochromen-12-one (1)

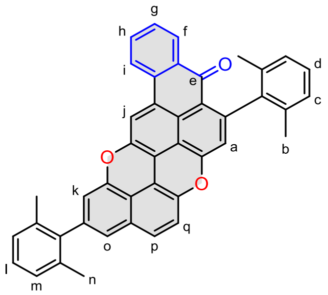

Following the general procedure (a). 100 mL round-bottomed flask, **17** (109 mg, 0.175 mmol), NaOH (5 eq, 35 mg, 0.875 mmol), NMP (25 mL), and decanethiol (2.5 eq, 105 µL, 0.262 mmol). Then 4 mL ER.

The crude material was chromatographed (SiO_2, CH_2Cl_2/heptane 1:1) to give **1** (yield = 87%, over two steps, 90 mg) as a red solid. **M.P.** > 300 °C. **^{1}H NMR** (600 MHz, CD_2Cl_2) δ 8.30 (dd, *J* = 8.0, 1.0 Hz, 1H, H_i), 8.24 (d, *J* = 8.2 Hz, 1H, H_f), 8.08 (s, 1H, H_j), 7.70 (ddd, *J* = 8.0, 7.1, 1.2 Hz, 1H, H_h), 7.53 – 7.48 (m, 1H, H_g), 7.46 (d, *J* = 9.0 Hz, 1H, H_p), 7.25 – 7.14 (m, 6H, H_c, H_d, H_l, H_m), 7.13 (d, *J* = 9.0 Hz, 1H, H_q), 7.03 (s, 1H, H_o), 6.84 (s, 1H, H_k), 6.69 (s, 1H, H_a), 2.16 (s, 6H, H_b), 2.03 (s, 6H, H_n).**^{13}C NMR** (151 MHz, CD_2Cl_2) δ 181.06 (C_e), 157.51, 153.16, 149.62, 145.71, 144.14, 143.35, 141.87, 141.52, 136.33, 134.52, 134.28, 132.67 (C_h), 132.33, 132.18, 128.56 (C_g), 128.28 (C_i), 128.18 (C_p), (127.79, 127.64, 126.96 (C_c, C_d, C_l, C_m)), 127.30, 126.88, 122.74 (C_f), 121.04 (C_o), 120.46, 120.23, 119.52, 117.77 (C_q), 114.53 (C_j, C_k), 114.19, 111.70 (C_a), 111.43, 20.96 (C_b), 20.61 (C_n). two signals (quaternary carbons) missing due to overlap. **IR** (neat): 3648, 2332, 2163, 2130, 2063, 2034, 1942, 1625, 1596, 1499, 1470, 1404, 1376, 1366, 1337, 1295, 1267, 1226, 1204, 1181, 1143, 1079, 1010, 954, 919, 856, 790, 755, 721, 699, 689, 671, 637, 613, 594, 577, 559, 550, 525, 514, 486, 474, 450, 434. **HRMS** (MALDI-timsTOF): *m/z* calcd for $C_{43}H_{28}O_3^+$: 592.2033 $[M]^+$; found: 592.2051. Single crystals suitable for X-ray diffraction were obtained by slow diffusion of MeOH in $CHCl_3$. (CCDC #2425204) For X-ray diffraction data see Section 4.

6,15-bis(2,6-dimethylphenyl)anthra[9',1',2':7,8,1]isochromeno[5,4,3-cde]anthra[9,1,2-hij]isochromene-5,14-dione (2)

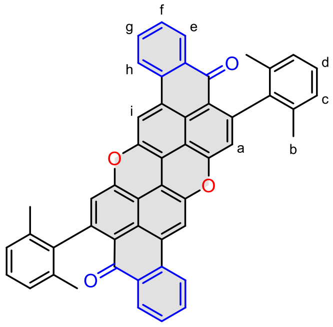

Following the general procedure (a). 100 mL round-bottomed flask, **18** (87 mg, 0.115 mmol), NaOH (10 eq, 22 mg, 1.15 mmol), NMP (20 mL), and decanethiol (5 eq, 139 µL, 0.575 mmol). Then 4 mL ER.

The crude material was chromatographed (SiO_2, CH_2Cl_2/heptane 7:3) to give **2** (yield = 63%, over two steps, 50 mg) as a dark red solid. **M.P.** > 300 °C. **^{1}H NMR** (600 MHz, CD_2Cl_2) δ 8.38 – 8.31 (m, 6H, H_h, H_e, H_i), 7.76 (t, *J* = 7.4 Hz, 2H, H_g), 7.56 (t, *J* = 7.5 Hz, 2H, H_f), 7.29 – 7.24 (m, 2H, H_d), 7.21 – 7.21 (m, *J* = 7.5 Hz, 4H, H_c), 7.02 (s, 2H, H_a), 2.05 (s, 12H, H_b). ^{13}C NMR could not be

recorded due to insufficient solubility of product. **HRMS** (MALDI-timsTOF): *m/z* calcd for $C_{50}H_{30}O_4^+$: 694.2139 $[M]^+$; found: 694.2142. Single crystals suitable for X-ray diffraction were obtained by slow diffusion of MeOH in CH_2Cl_2. (CCDC #2306817) For X-ray diffraction data see Section 4.

PXX-Meta-Dimer (3)

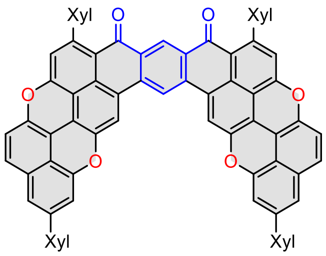

Following the general procedure (a). 50 mL round-bottomed flask, **19** (53 mg, 0.045 mmol), NaOH (10 eq, 18 mg, 0.45 mmol), NMP (8 mL), and decanethiol (5 eq, 54 µL, 0.225 mmol). Then 2 mL ER.
The crude material was sonicated in heptane for 30 min then filtered. The collected solid was chromatographed (recycling GPC, CH_2Cl_2) to give **3** (yield = 54%, over two steps, 27 mg) as a purple solid. **HRMS** (MALDI-timsTOF): *m/z* calcd for $C_{80}H_{50}O_6^+$: 1106.3602 $[M]^+$; found: 1106.3605.

PXX-Para-Dimer (4)

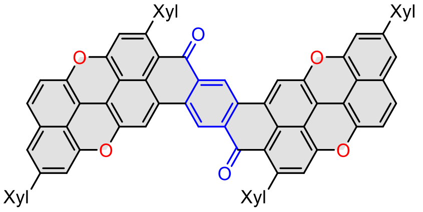

Following the general procedure (a). 50 mL round-bottomed flask, **20** (31 mg, 0.026 mmol), NaOH (10 eq, 11 mg, 0.26 mmol), NMP (6 mL), and decanethiol (5 eq, 31 µL, 0.13 mmol). Then 2 mL ER. The crude material was sonicated in heptane for 30 min then filtered. The collected solid was chromatographed (recycling GPC, CH_2Cl_2) to give **3** (yield = 66%, over two steps, 19 mg) as a grey-greenish solid. **HRMS** (MALDI-timsTOF): *m/z* calcd for $C_{80}H_{50}O_6^+$: 1106.3602 $[M]^+$; found: 1106.3634.

PXX-Meta-Trimer (5)

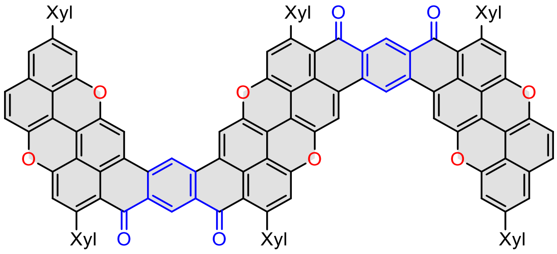

Following the general procedure (a). 50 mL round-bottomed flask, **23** (37 mg, 0.02 mmol), NaOH (20 eq, 16 mg, 0.4 mmol), NMP (8 mL), and decanethiol (10 eq, 48 µL, 0.2 mmol). Then 2 mL ER.

The crude material was sonicated in heptane for 30 min then filtered. The collected solid was chromatographed (recycling GPC, CH_2Cl_2) to give **5** (yield = 41%, over two steps, 14 mg) as a purple-blueish solid. **HRMS** (MALDI-timsTOF): *m/z* calcd for $C_{124}H_{74}O_{10}^+$: 1723.5310 $[M]^+$; found: 1723.5328.

PXX-Para-Trimer (6)

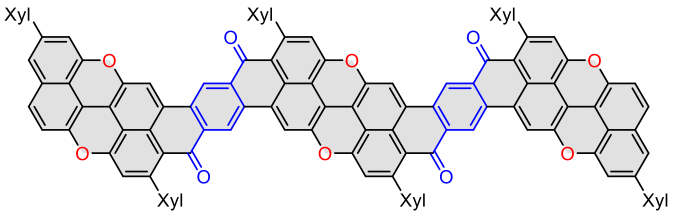

Following the general procedure (a). 50 mL round-bottomed flask, **26** (19 mg, 0.01 mmol), NaOH (20 eq, 8 mg, 0.2 mmol), NMP (8 mL), and decanethiol (10 eq, 24 µL, 0.1 mmol). Then 2 mL ER. The crude material was sonicated in heptane for 30 min then filtered. The collected solid was chromatographed (GPC, CH_2Cl_2) to give **6** (yield = 23%, over two steps, 4 mg) as a blue solid. **HRMS** (MALDI-timsTOF): *m/z* calcd for $C_{124}H_{74}O_{10}^+$: 1723.5310 $[M]^+$; found: 1723.5314

2.1. NMR and HRMS spectra

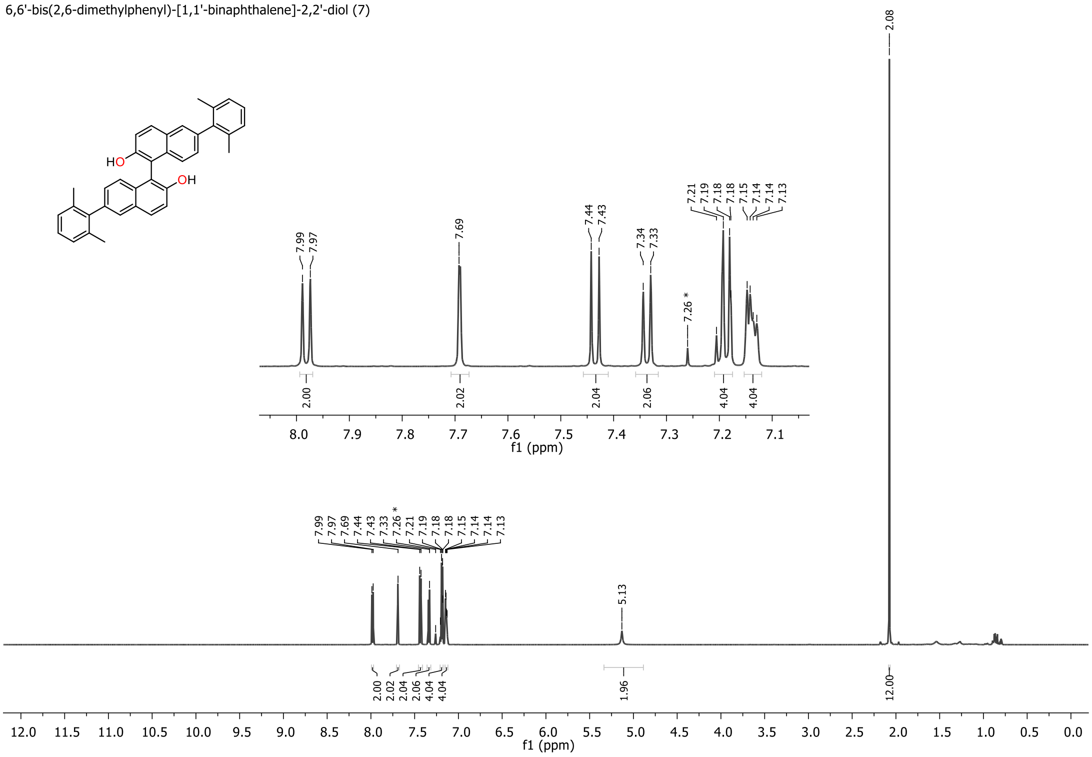

Figure S1. **^{1}H NMR (600 MHz, *$CDCl_3$) spectrum of 7.**

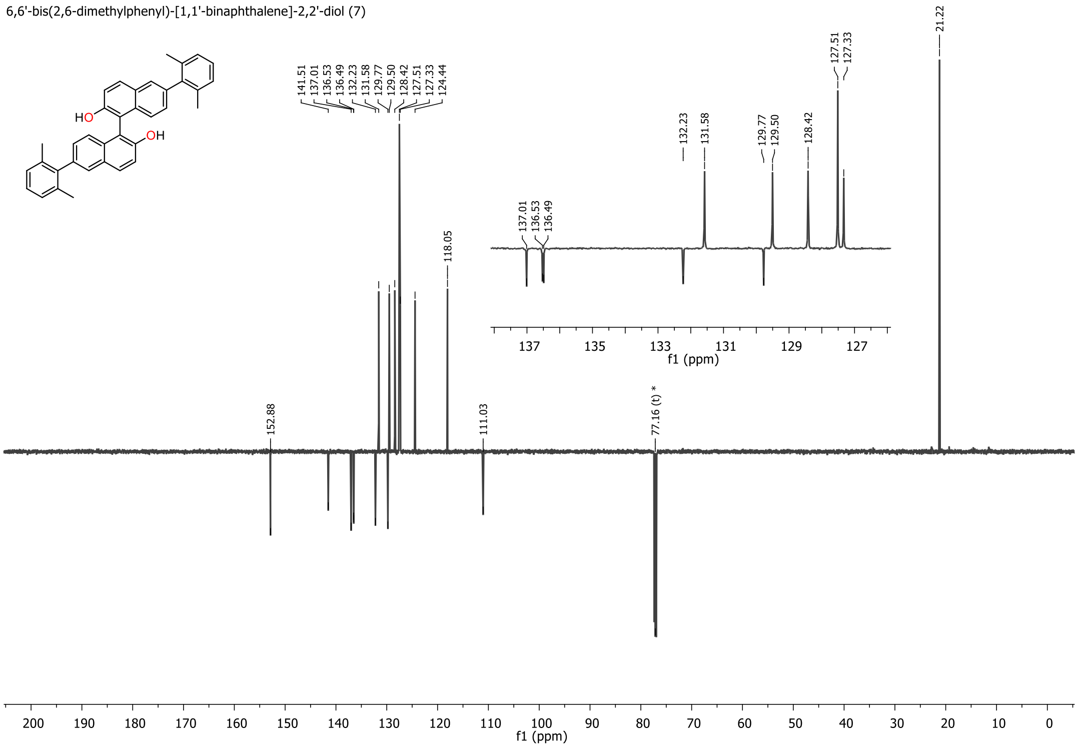

Figure S2. ^{13}C NMR (151 MHz, *$CDCl_3$) spectrum of **7**.

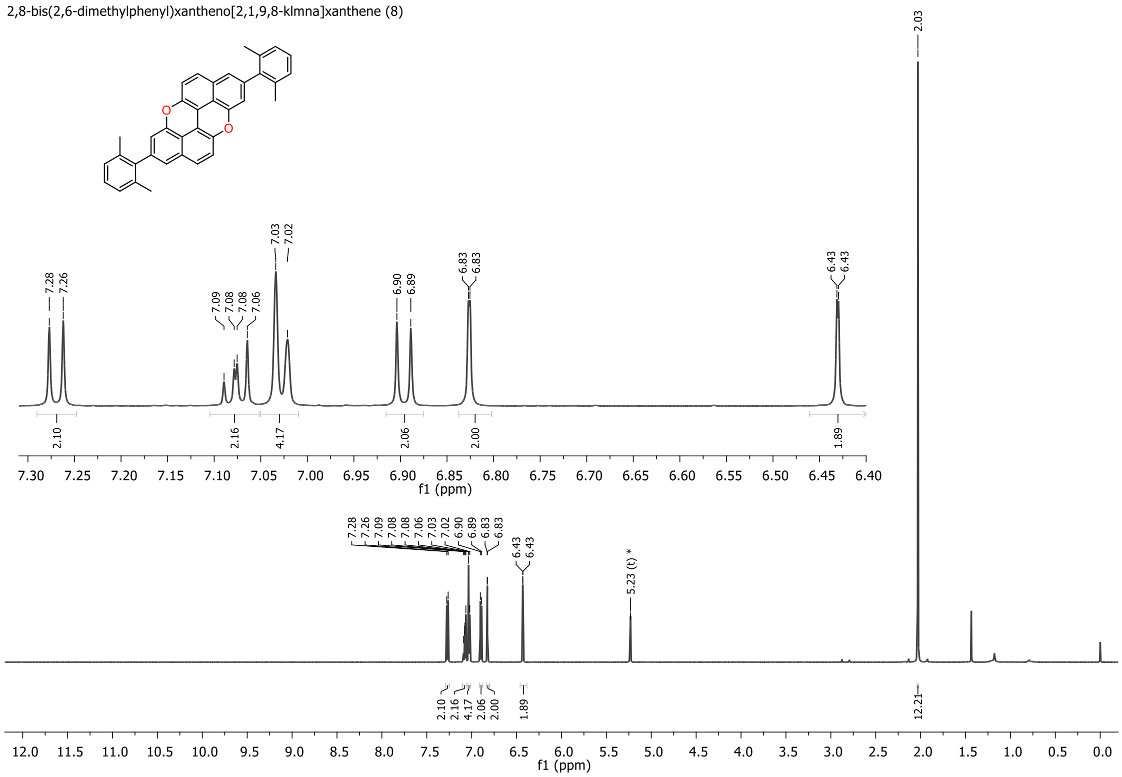

Figure S3. ^{1}H NMR (600 MHz, *CD_2Cl_2) spectrum of **10**.

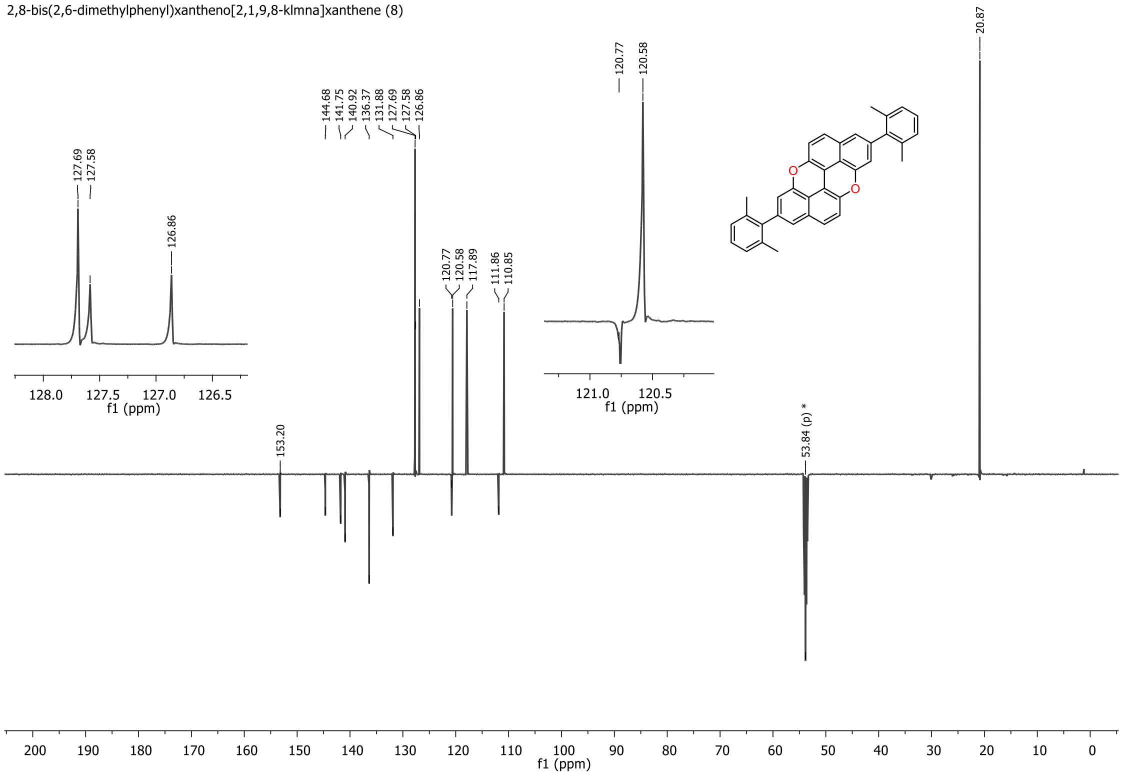

Figure S4. ^{13}C NMR (151 MHz, *CD_2Cl_2) spectrum of **10**.

2-(2,8-bis(2,6-dimethylphenyl)xantheno[2,1,9,8-klmna]xanthen-4-yl)-4,4,5,5-tetramethyl-1,3,2-dioxaborolane (9)

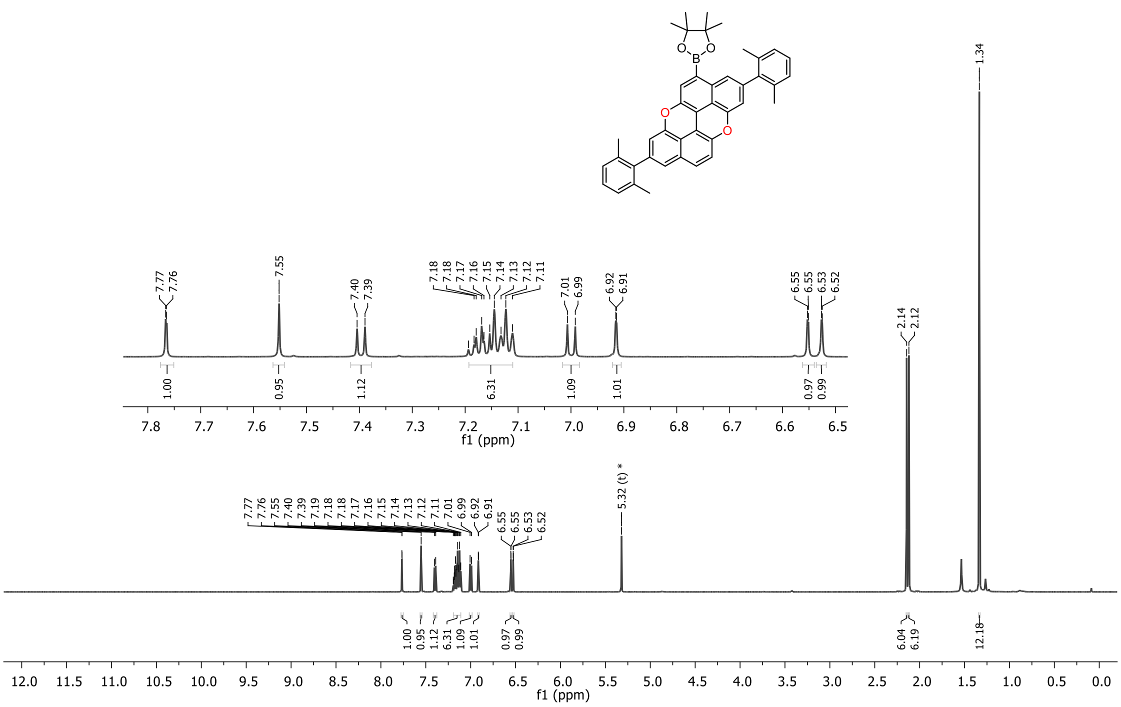

Figure S5. ^{1}H NMR (600 MHz, $^*CD_2Cl_2$) spectrum of **9**.

2-(2,8-bis(2,6-dimethylphenyl)xantheno[2,1,9,8-klmna]xanthen-4-yl)-4,4,5,5-tetramethyl-1,3,2-dioxaborolane (9)

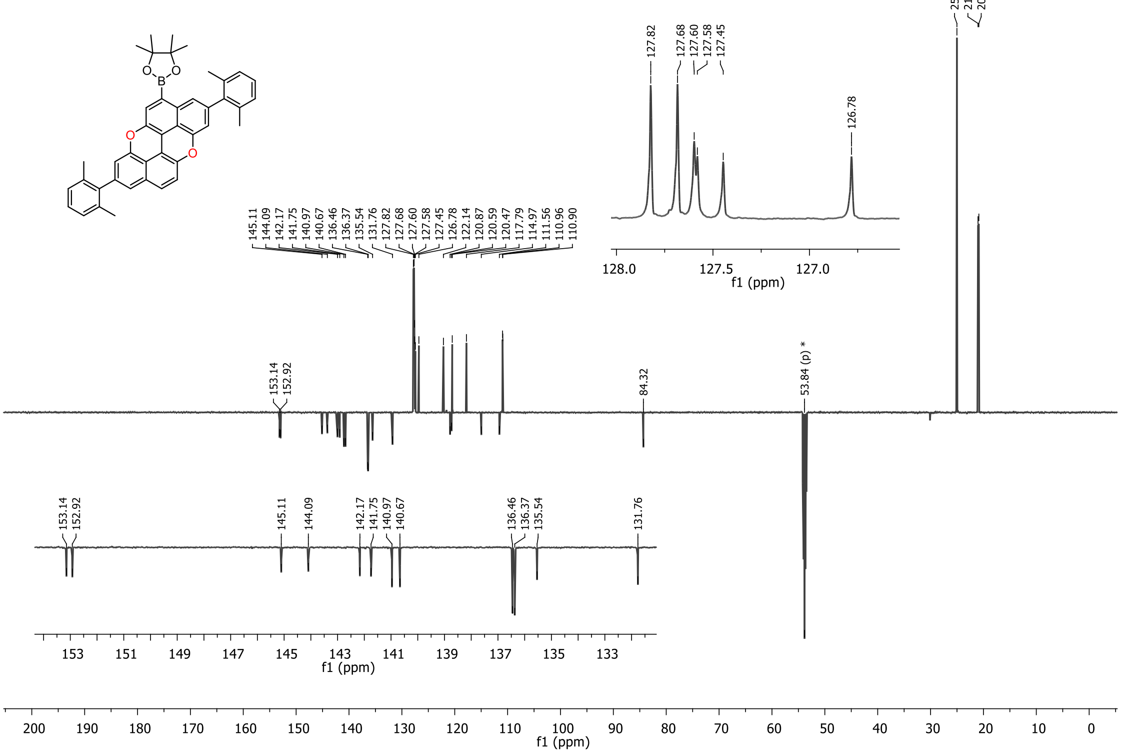

Figure S6. ^{13}C NMR (151 MHz, $^*CD_2Cl_2$) spectrum of **9**.

2-(2,8-bis(2,6-dimethylphenyl)xantheno[2,1,9,8-klmna]xanthen-4-yl)-4,4,5,5-tetramethyl-1,3,2-dioxaborolane (9)

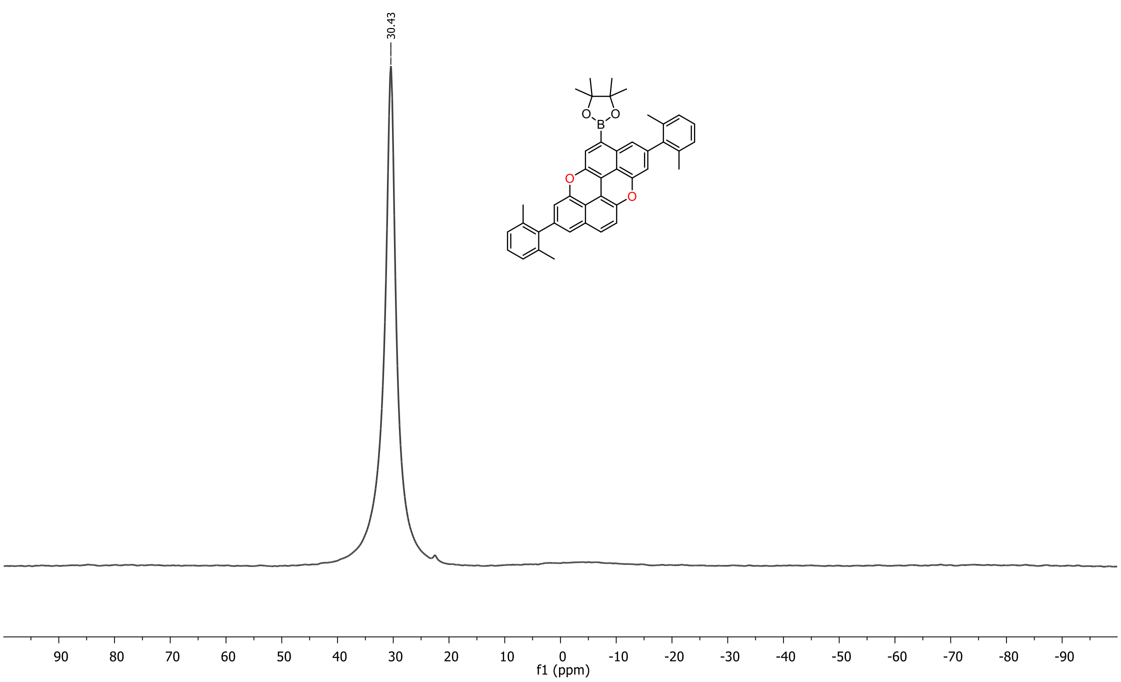

Figure S7. ^{11}B NMR (193 MHz, in CD_2Cl_2) spectrum of **9**.

2,2'-(2,8-bis(2,6-dimethylphenyl)xantheno[2,1,9,8-klmna]xanthene-4,10-diyl)bis(4,4,5,5-tetramethyl-1,3,2-dioxaborolane) (10)

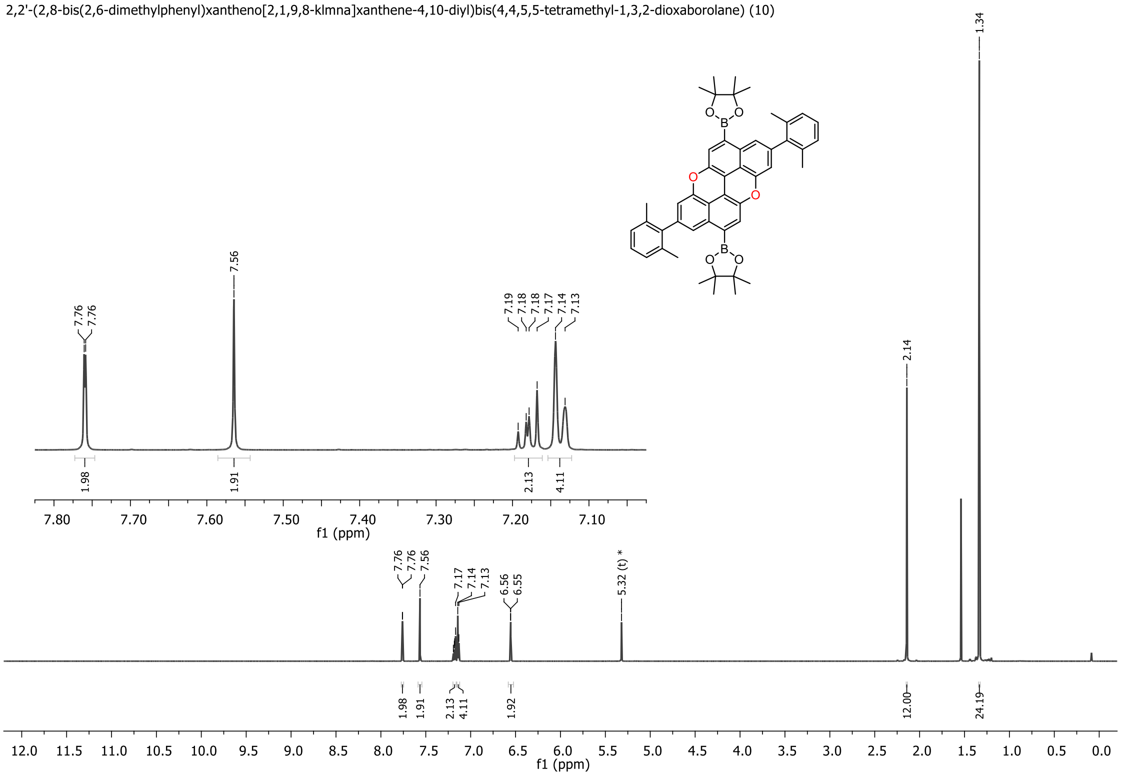

Figure S8. ^{1}H NMR (600 MHz, *CD_2Cl_2) spectrum of **10**.

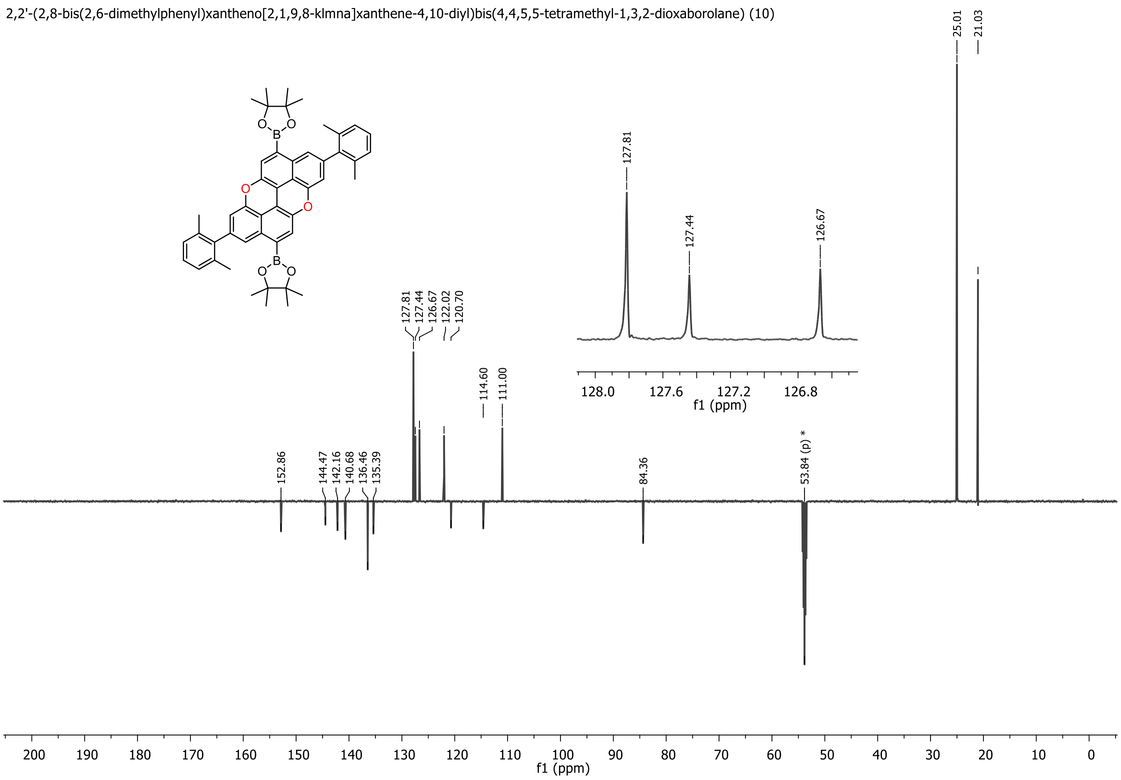

Figure S9. ^{13}C NMR (151 MHz, *CD_2Cl_2) spectrum of **10**.

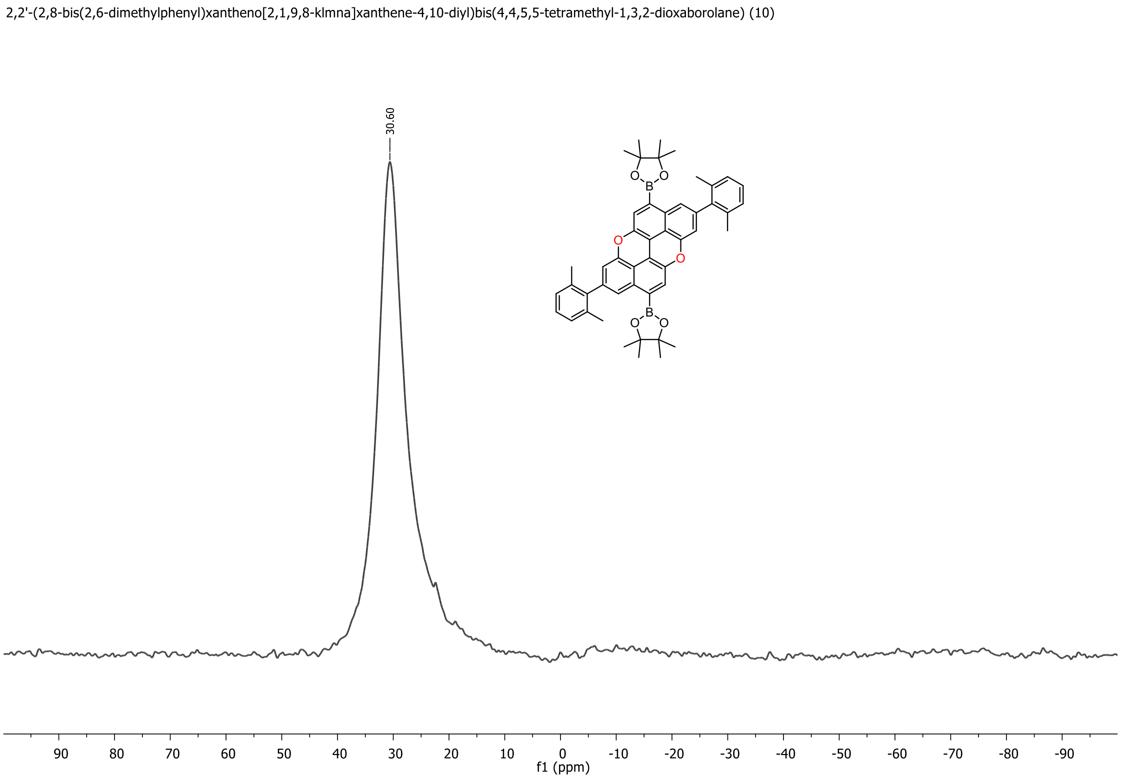

Figure S10. ^{11}B NMR (193 MHz, in CD_2Cl_2) spectrum of **10**.

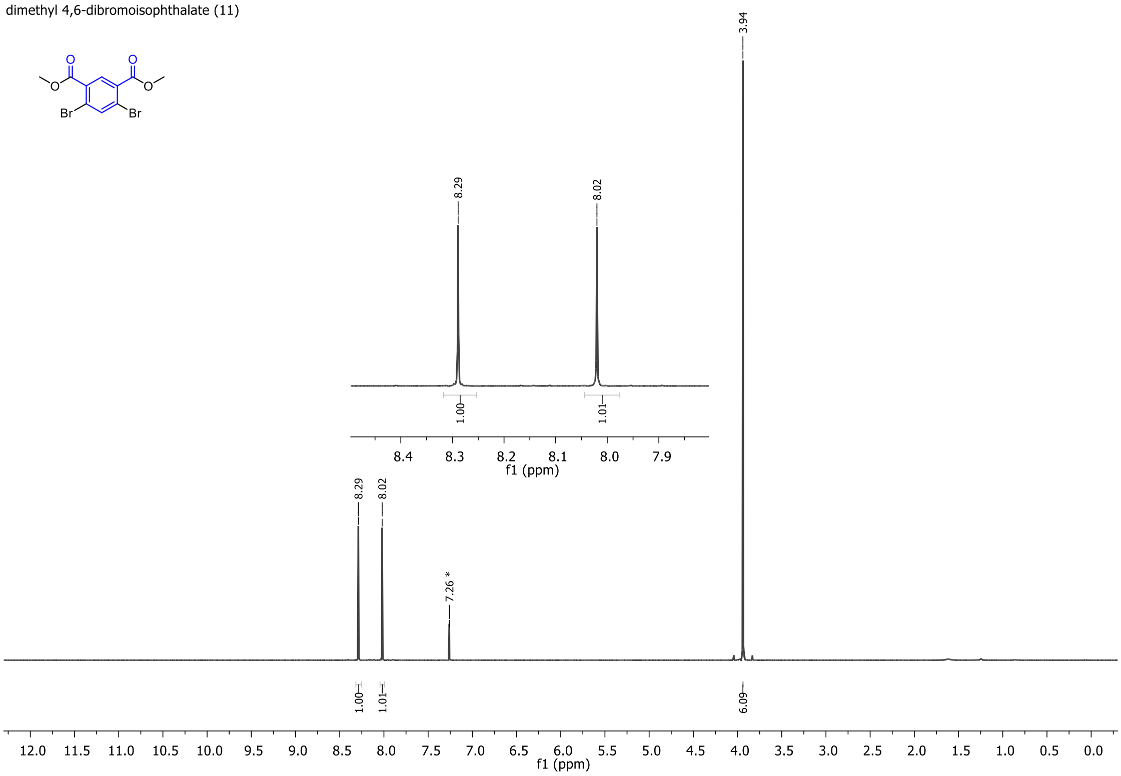

Figure S11. ^{1}H NMR (700 MHz, *$CDCl_3$) spectrum of **11**.

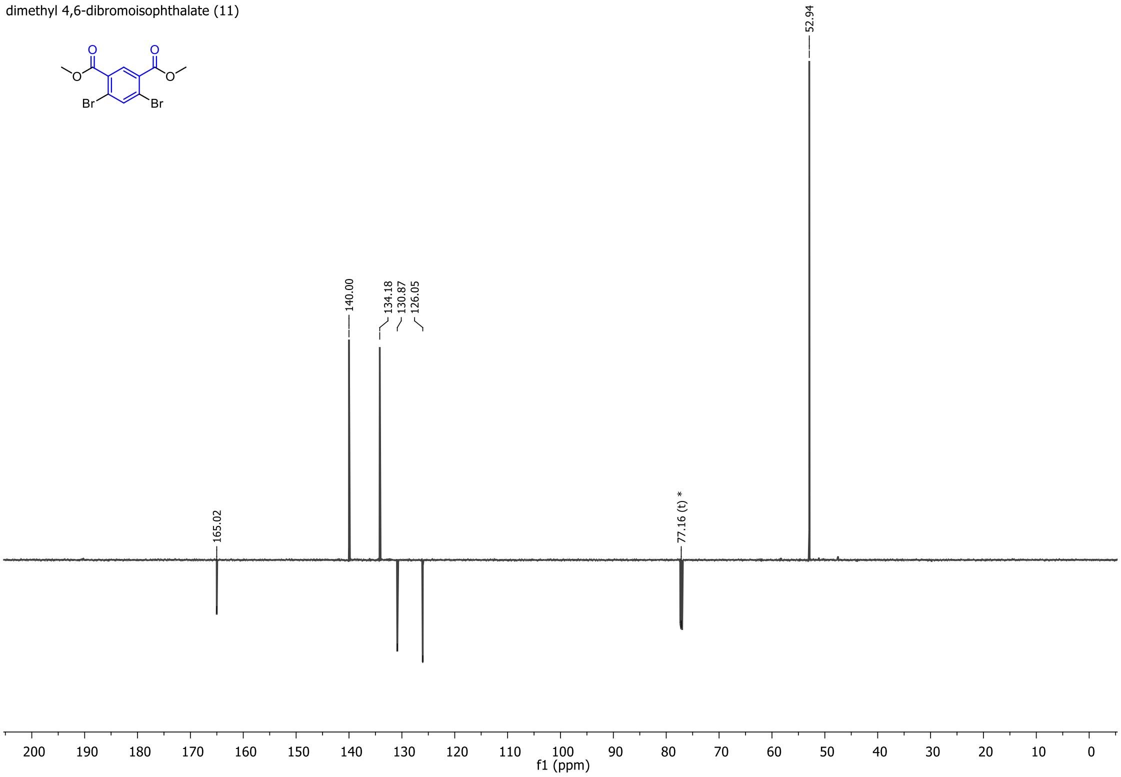

Figure S12. ^{13}C NMR (176 MHz, *$CDCl_3$) spectrum of **11**.

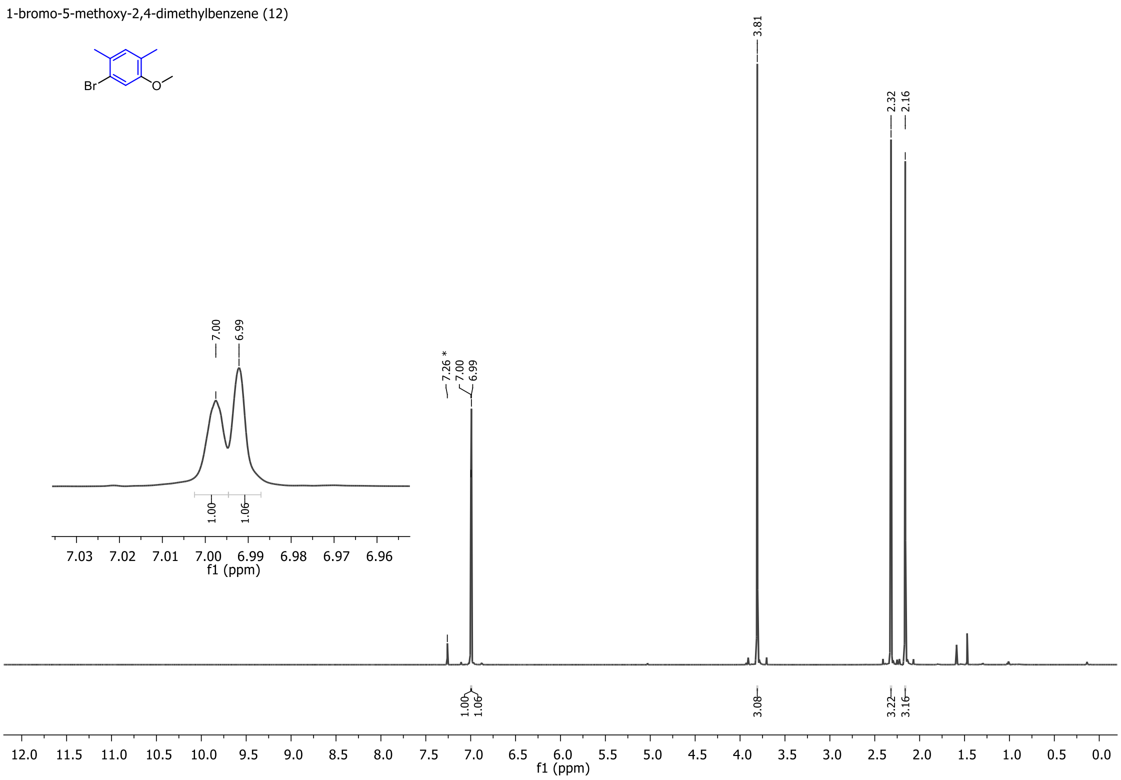

Figure S13. ^{1}H NMR (700 MHz, *$CDCl_3$) spectrum of **12**.

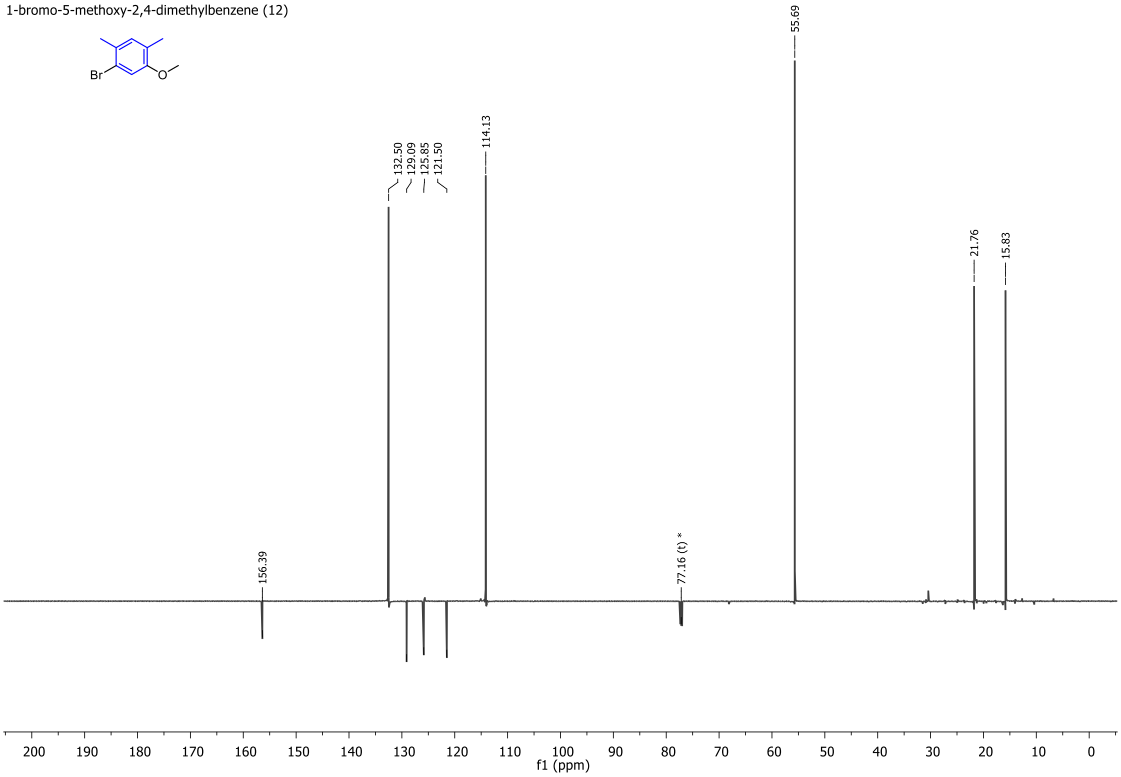

Figure S14. ^{13}C NMR (176 MHz, *$CDCl_3$) spectrum of **12**.

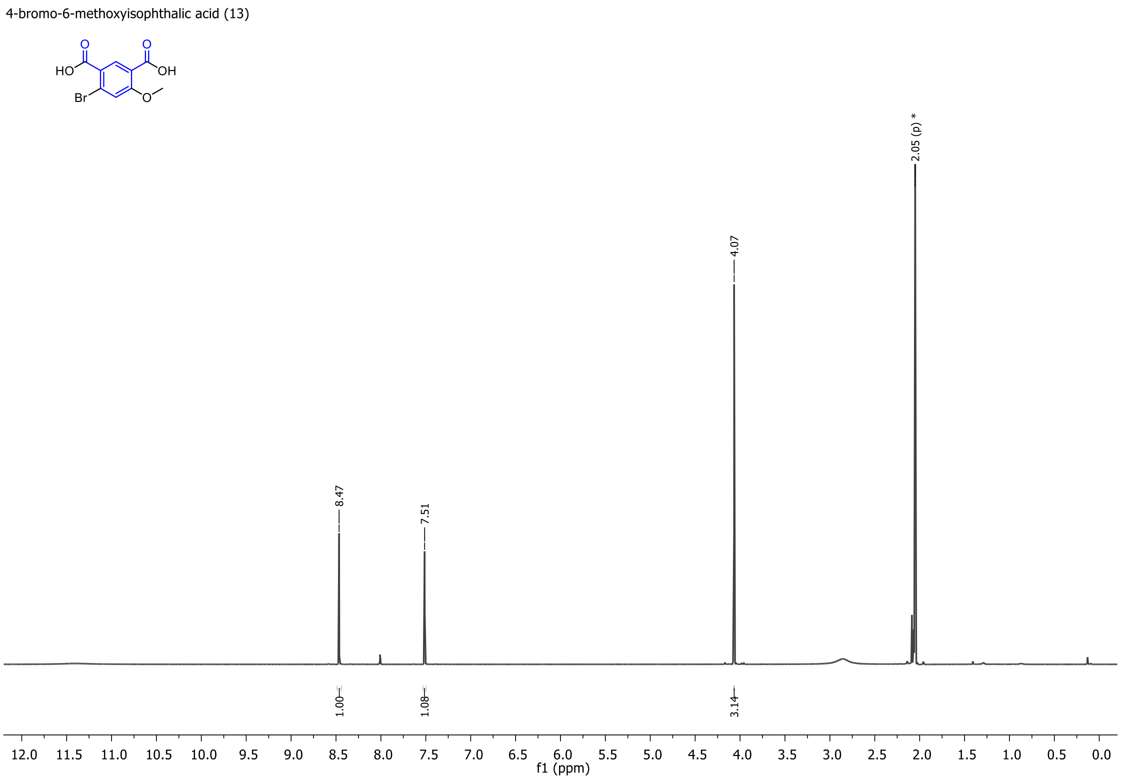

Figure S15. ^{1}H NMR (700 MHz, *Acetone-*d6*) spectrum of **13**.

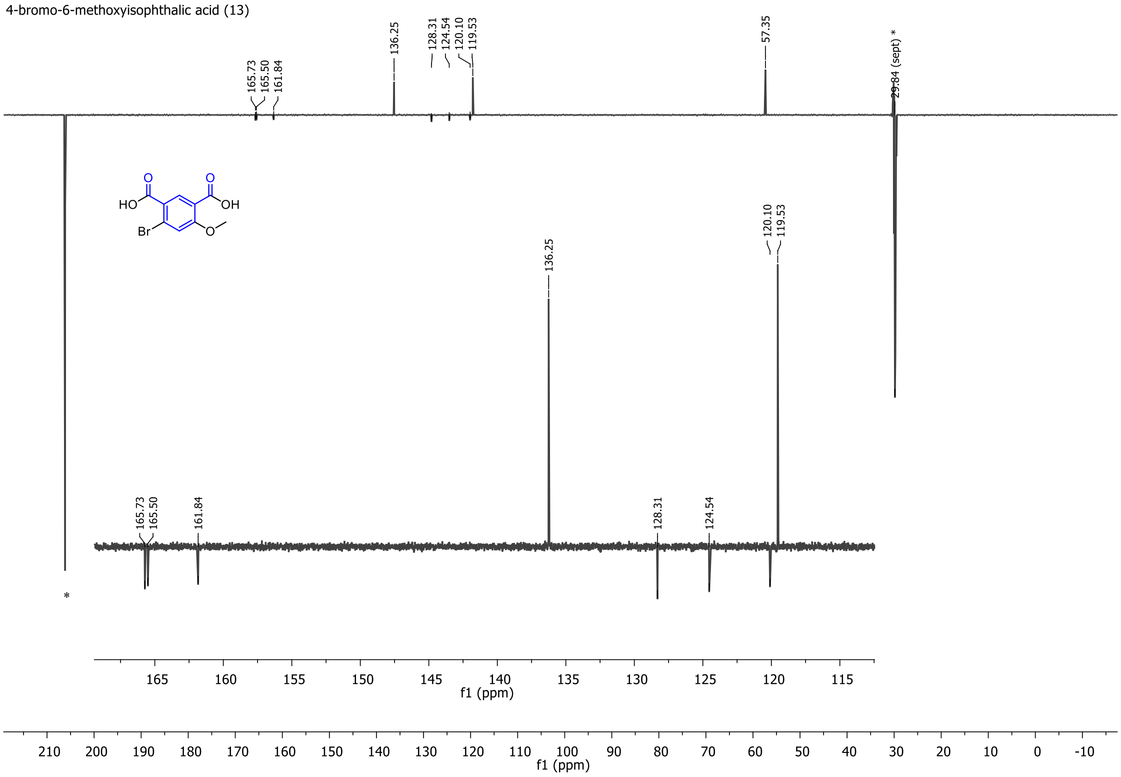

Figure S16. ^{13}C NMR (176 MHz, *Acetone-*d6*) spectrum of **13**.

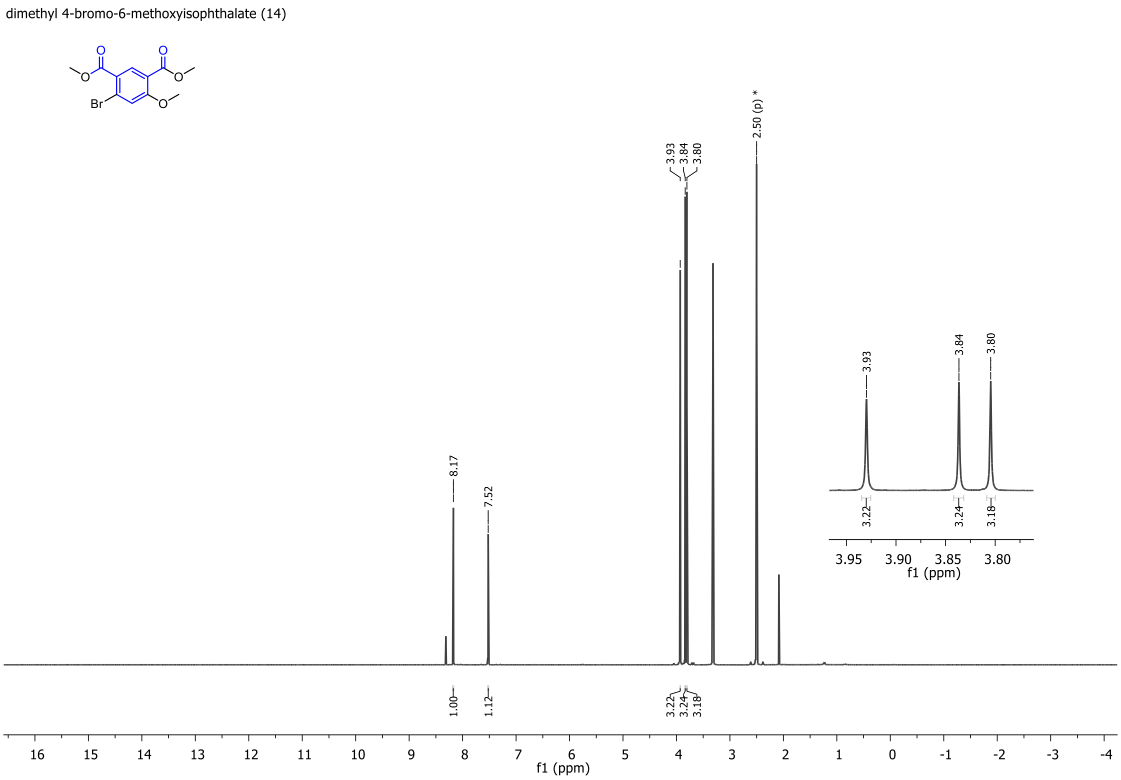

Figure S17. ^{1}H NMR (600 MHz, *DMSO-*d6*) spectrum of **14**.

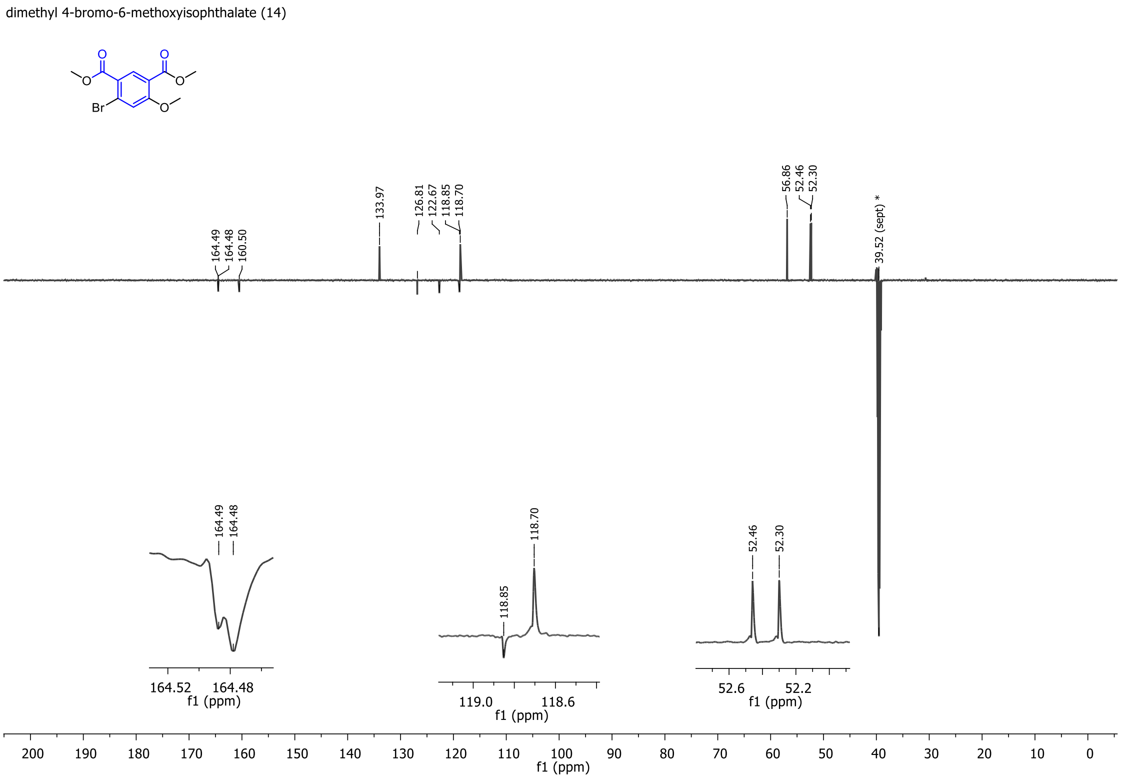

Figure S18. ^{13}C NMR (151 MHz, *DMSO-*d6*) spectrum of **14**.

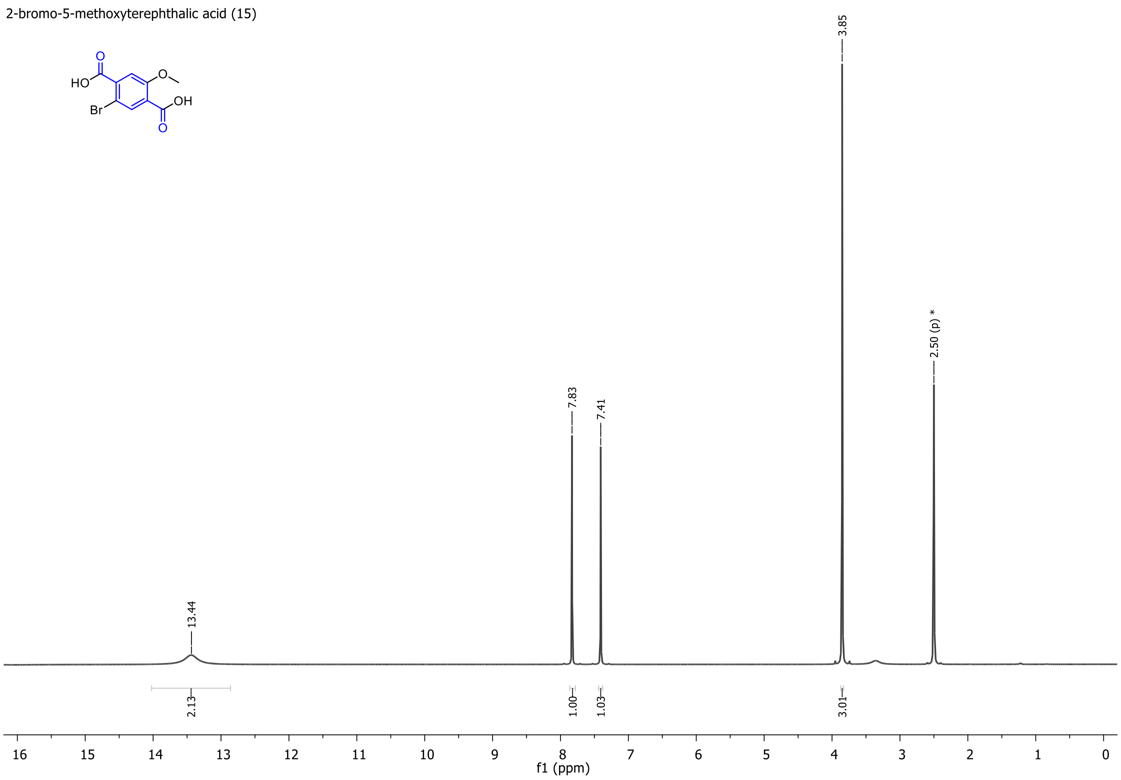

Figure S19. ^{1}H NMR (700 MHz, *DMSO-*d6*) spectrum of **15**.

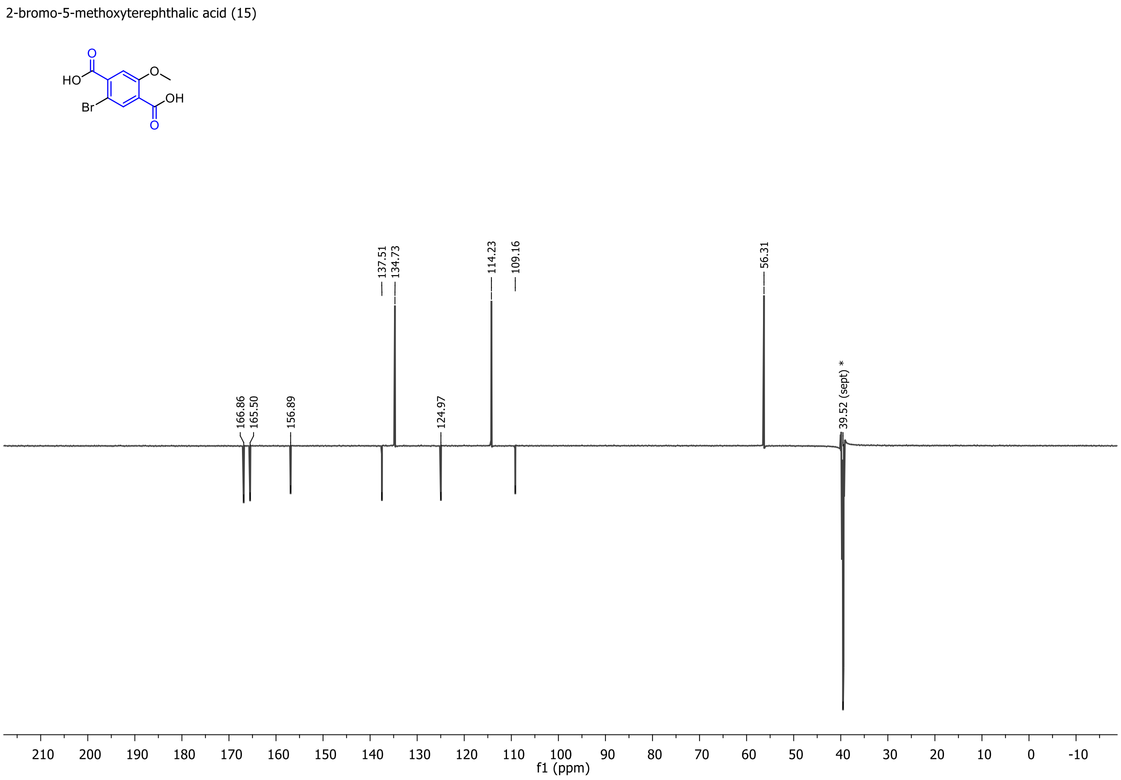

Figure S20. ^{13}C NMR (176 MHz, *DMSO-*d6*) spectrum of **15**.

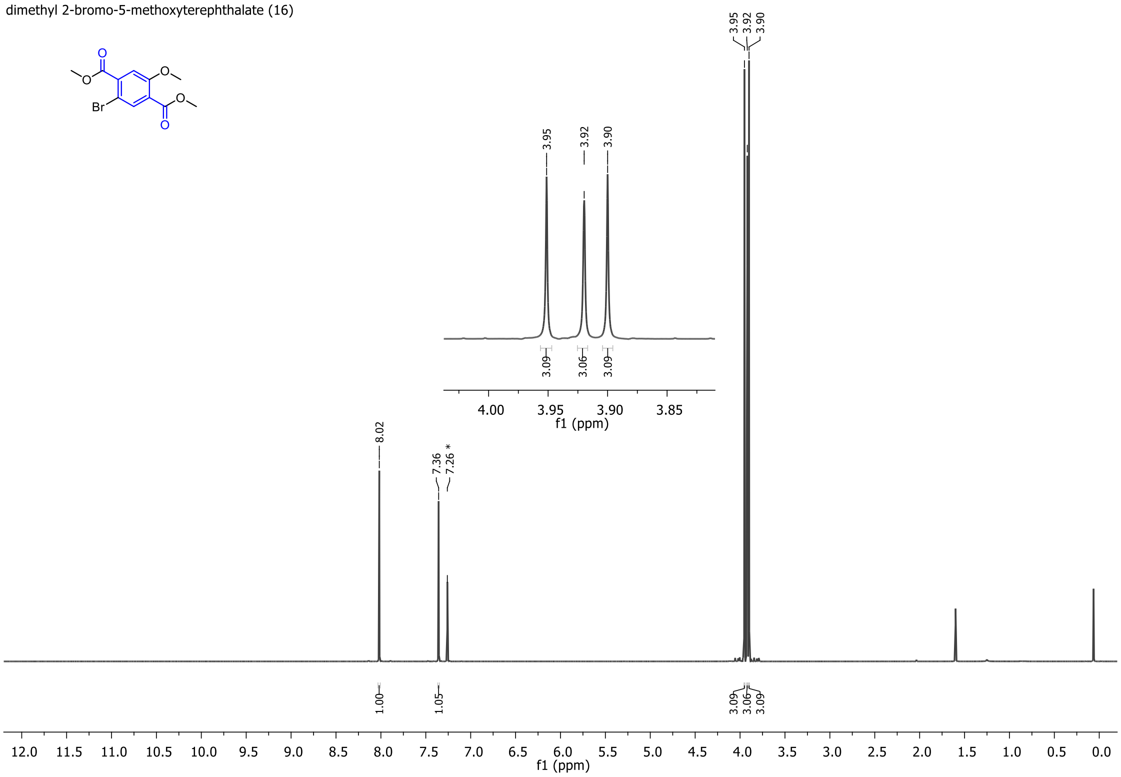

Figure S21. ^{1}H NMR (700 MHz, *$CDCl_3$) spectrum of **16**.

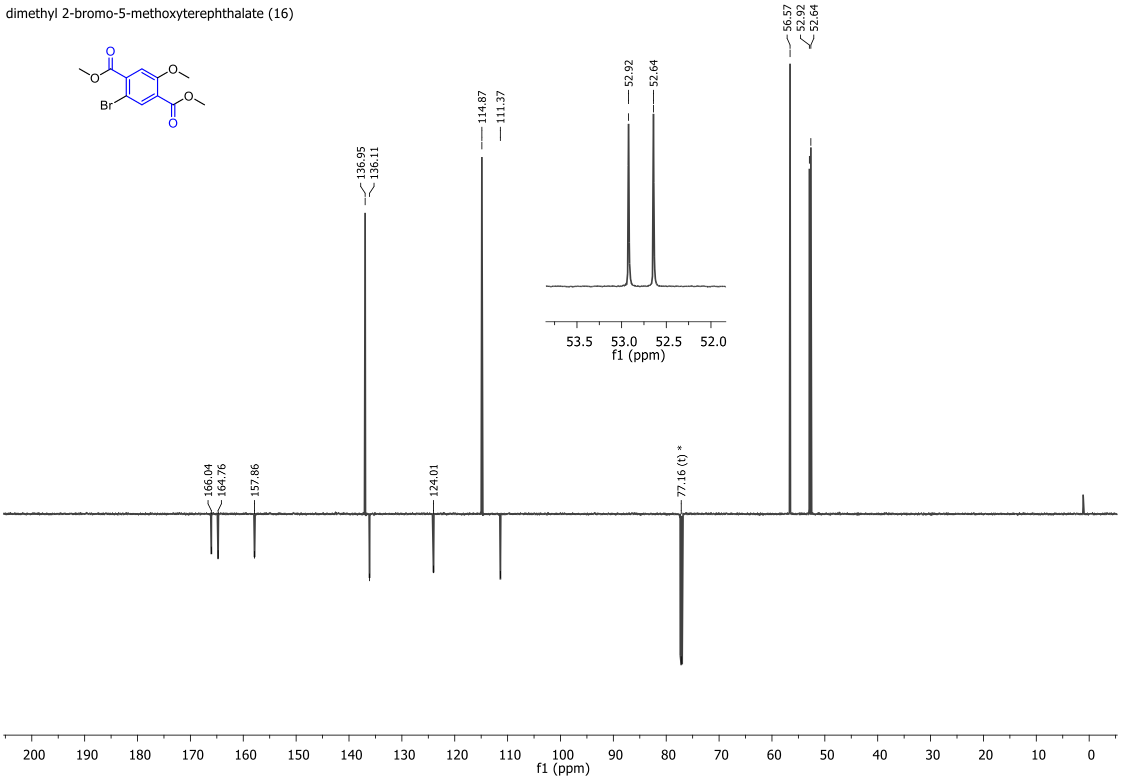

Figure S22. ^{13}C NMR (176 MHz, *$CDCl_3$) spectrum of **16**.

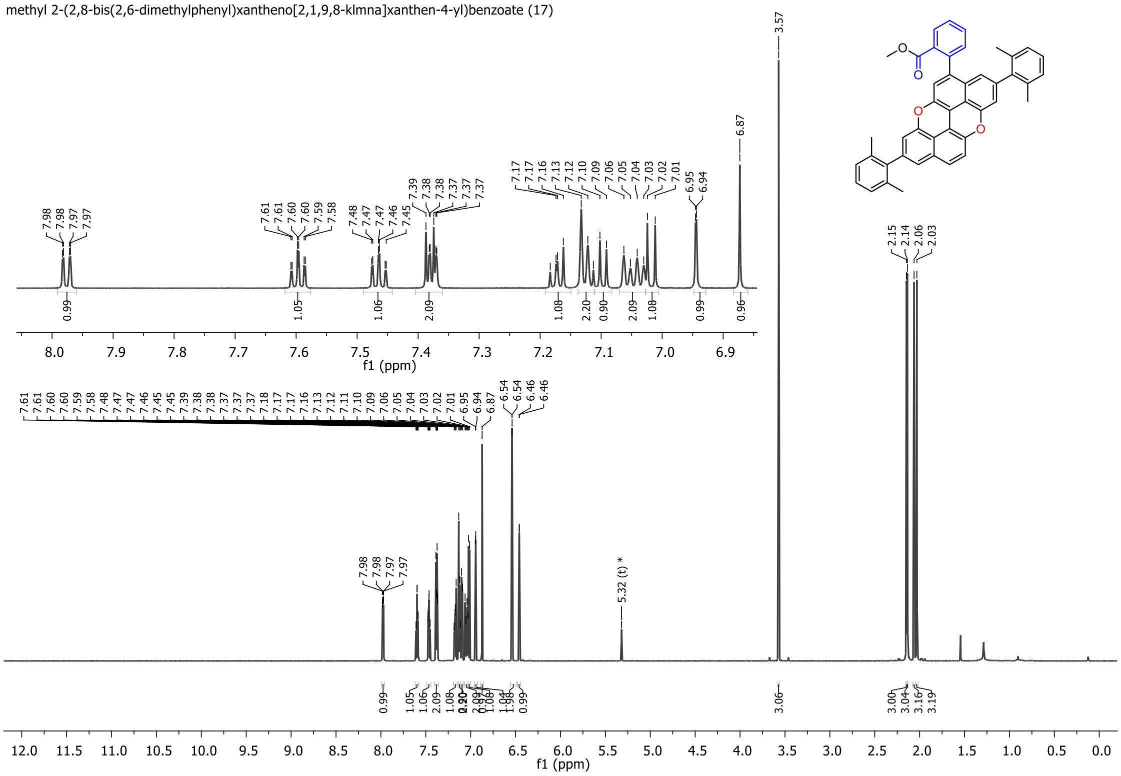

Figure S23. ^{1}H NMR (700 MHz, *CD_2Cl_2) spectrum of **17**.

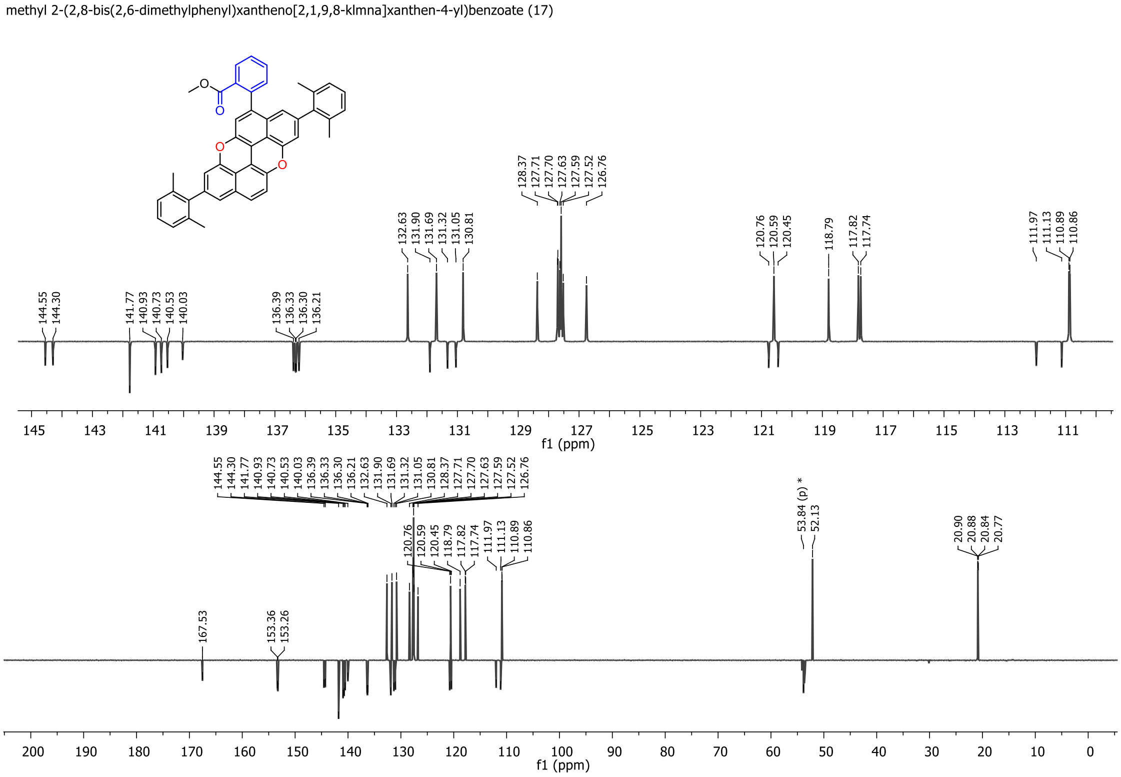

Figure S24. ^{13}C NMR (176 MHz, *CD_2Cl_2) spectrum of **17**.

dimethyl 2,2'-(2,8-bis(2,6-dimethylphenyl)xantheno[2,1,9,8-klmna]xanthene-4,10-diyl)dibenzoate (18)

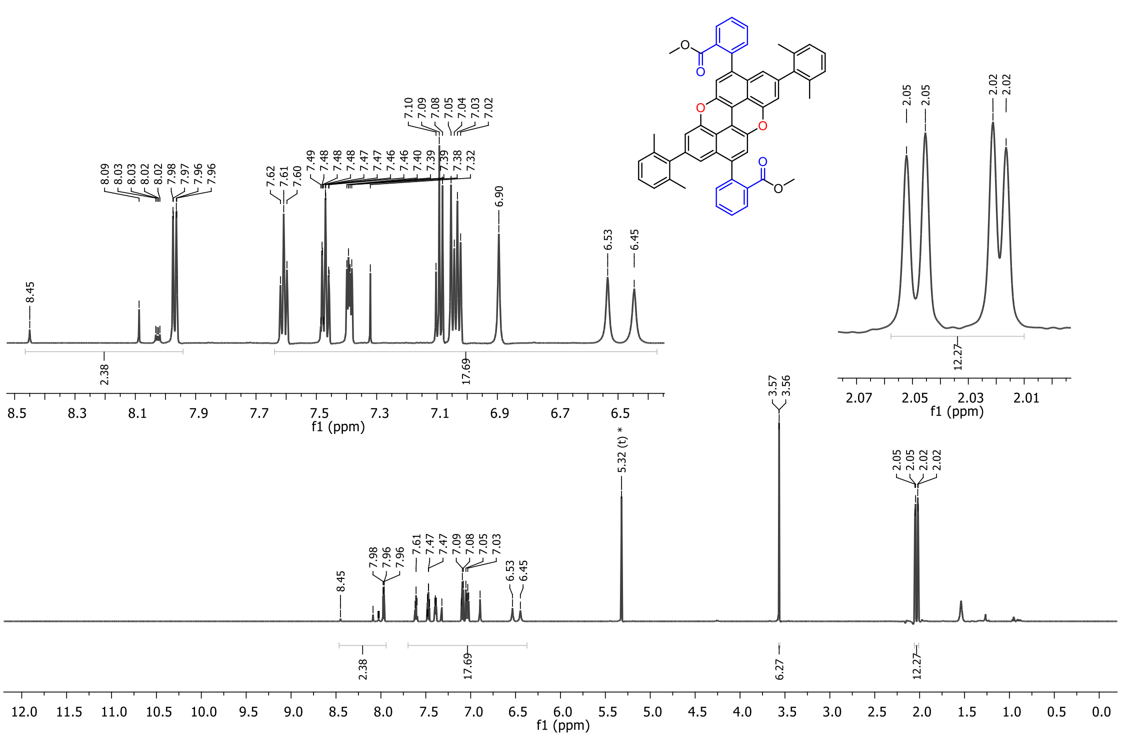

Figure S25. ^{1}H NMR (700 MHz, *CD_2Cl_2) spectrum of **18**.

dimethyl 2,2'-(2,8-bis(2,6-dimethylphenyl)xantheno[2,1,9,8-klmna]xanthene-4,10-diyl)dibenzoate (18)

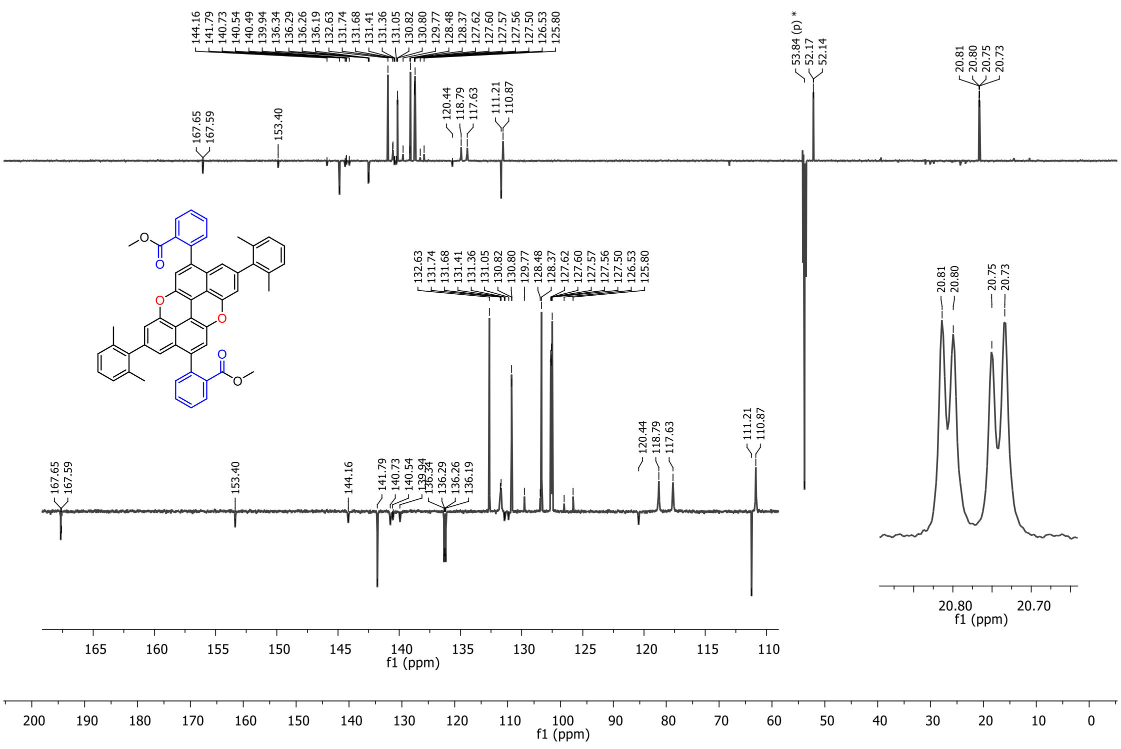

Figure S26. ^{13}C NMR (176 MHz, *CD_2Cl_2) spectrum of **18**.

dimethyl 4,6-bis(2,8-bis(2,6-dimethylphenyl)xantheno[2,1,9,8-klmna]xanthen-4-yl)isophthalate (19)

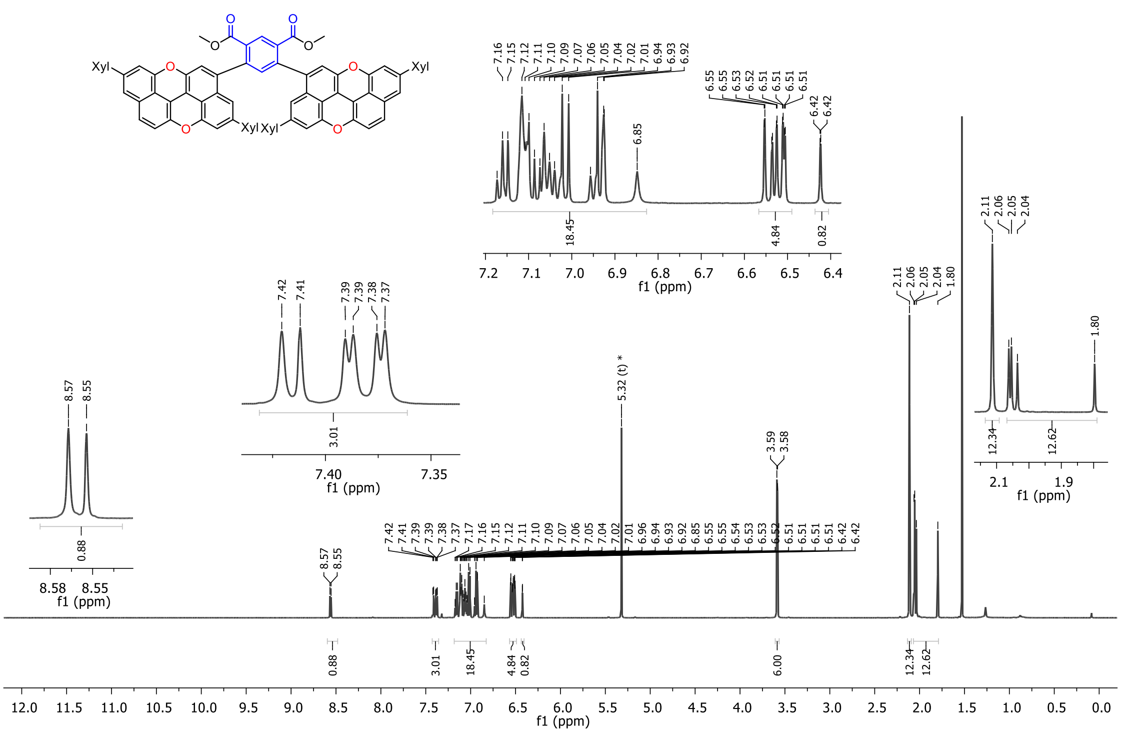

Figure S27. ^{1}H NMR (600 MHz, *CD_2Cl_2) spectrum of **19**.

dimethyl 4,6-bis(2,8-bis(2,6-dimethylphenyl)xantheno[2,1,9,8-klmna]xanthen-4-yl)isophthalate (19)

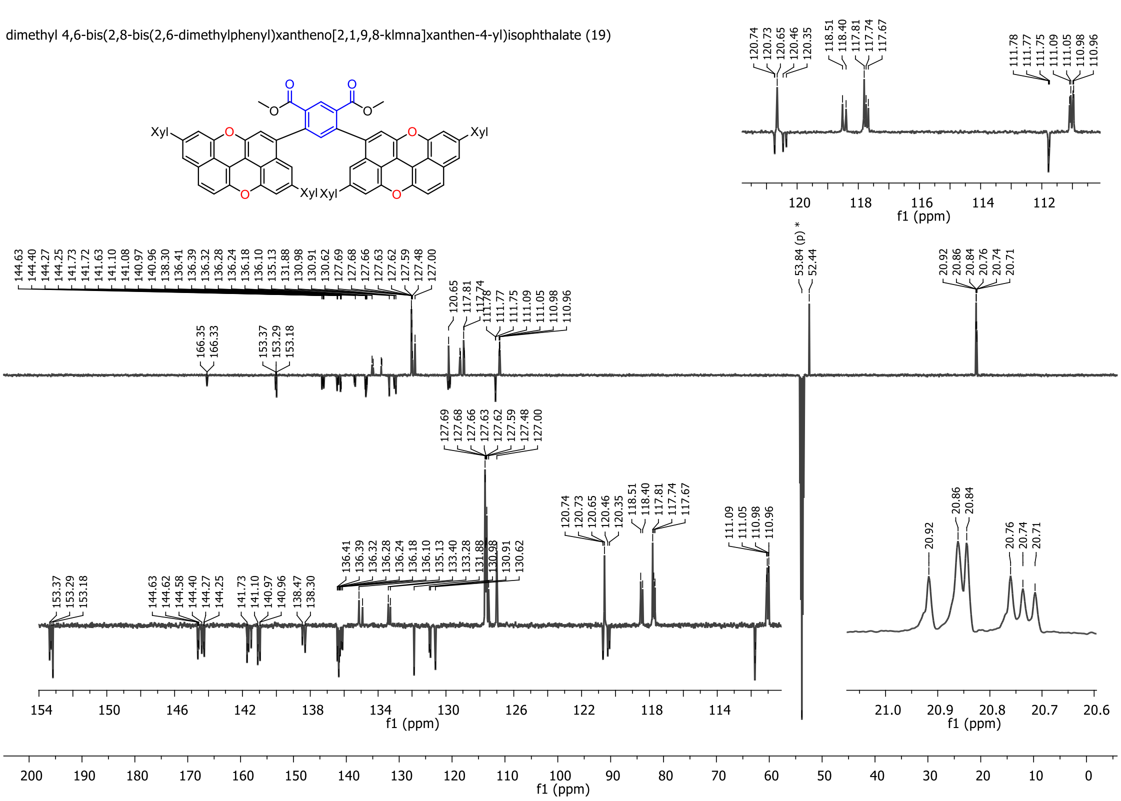

Figure S28. ^{13}C NMR (151 MHz, *CD_2Cl_2) spectrum of **19**.

dimethyl 2,5-bis(2,8-bis(2,6-dimethylphenyl)xantheno[2,1,9,8-klmna]xanthen-4-yl)terephthalate (20)

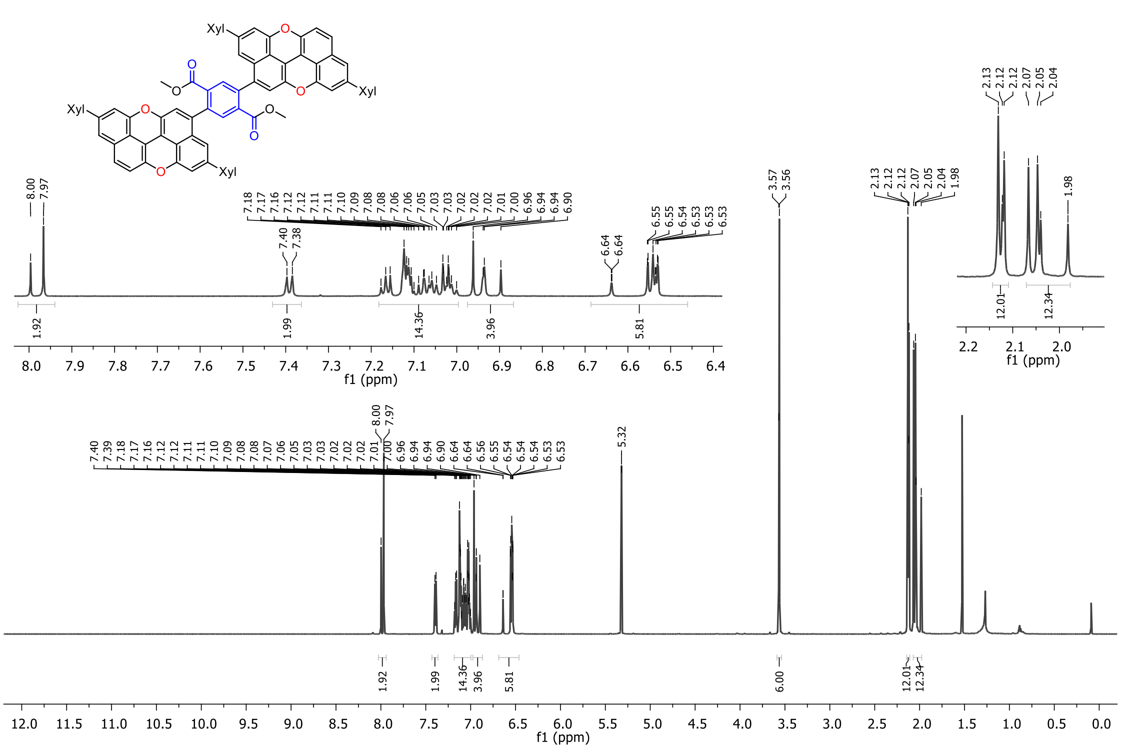

Figure S29. ^{1}H NMR (700 MHz, *CD_2Cl_2) spectrum of **20**.

dimethyl 2,5-bis(2,8-bis(2,6-dimethylphenyl)xantheno[2,1,9,8-klmna]xanthen-4-yl)terephthalate (20)

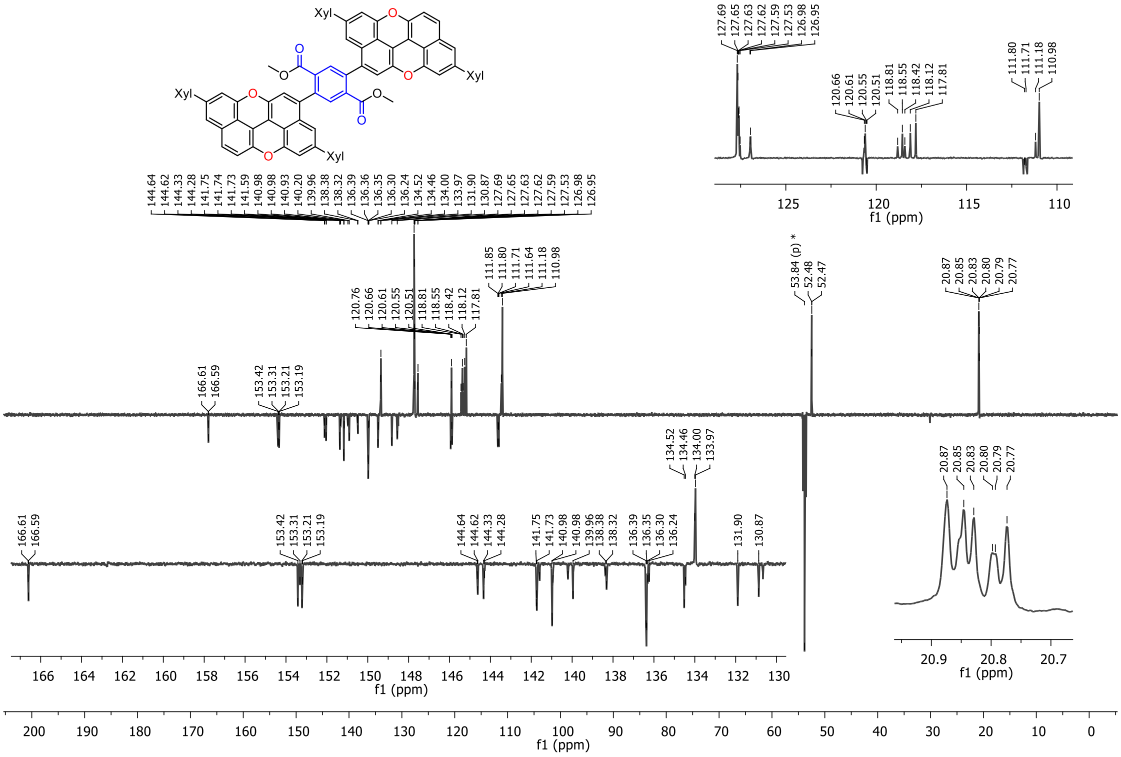

Figure S30. ^{13}C NMR (176 MHz, *CD_2Cl_2) spectrum of **20**.

tetramethyl 6,6'-(2,8-bis(2,6-dimethylphenyl)xantheno[2,1,9,8-klmna]xanthene-4,10-diyl)bis(4-methoxyisophthalate) (21)

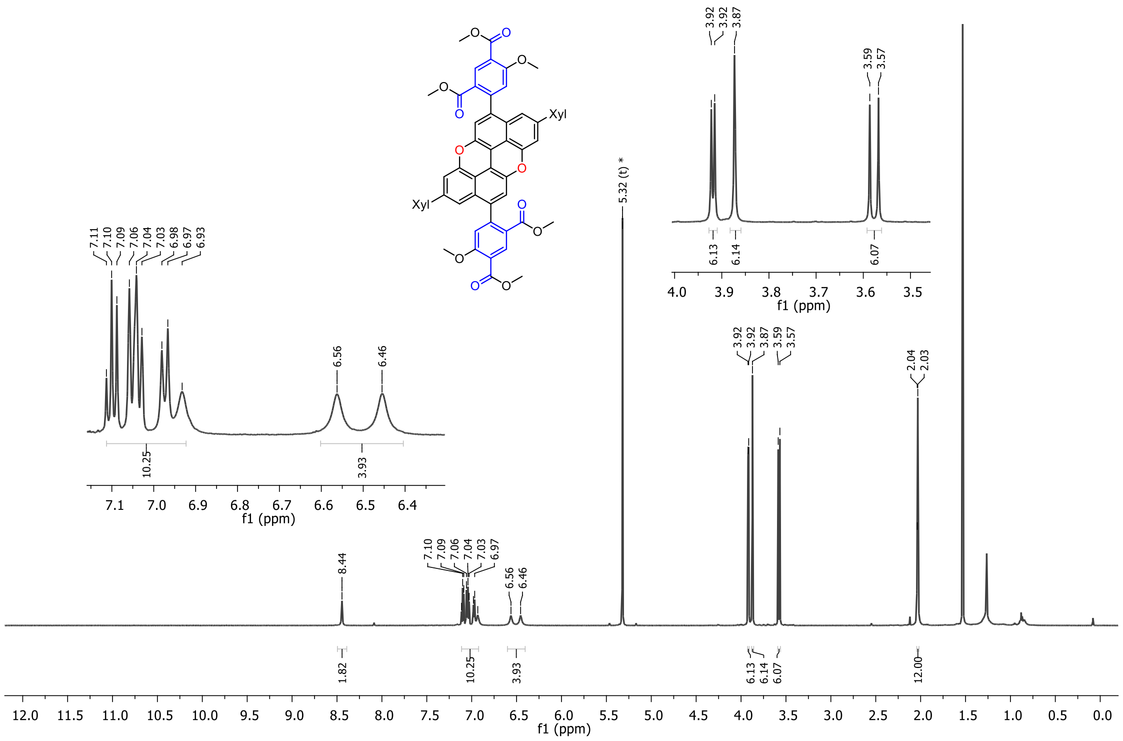

Figure S31. ^{1}H NMR (600 MHz, *CD_2Cl_2) spectrum of **21**.

6,15-bis(2,6-dimethylphenyl)anthra[9',1',2':7,8,1]isochromeno[5,4,3-cde]anthra[9,1,2-hij]isochromene-5,14-dione (2)

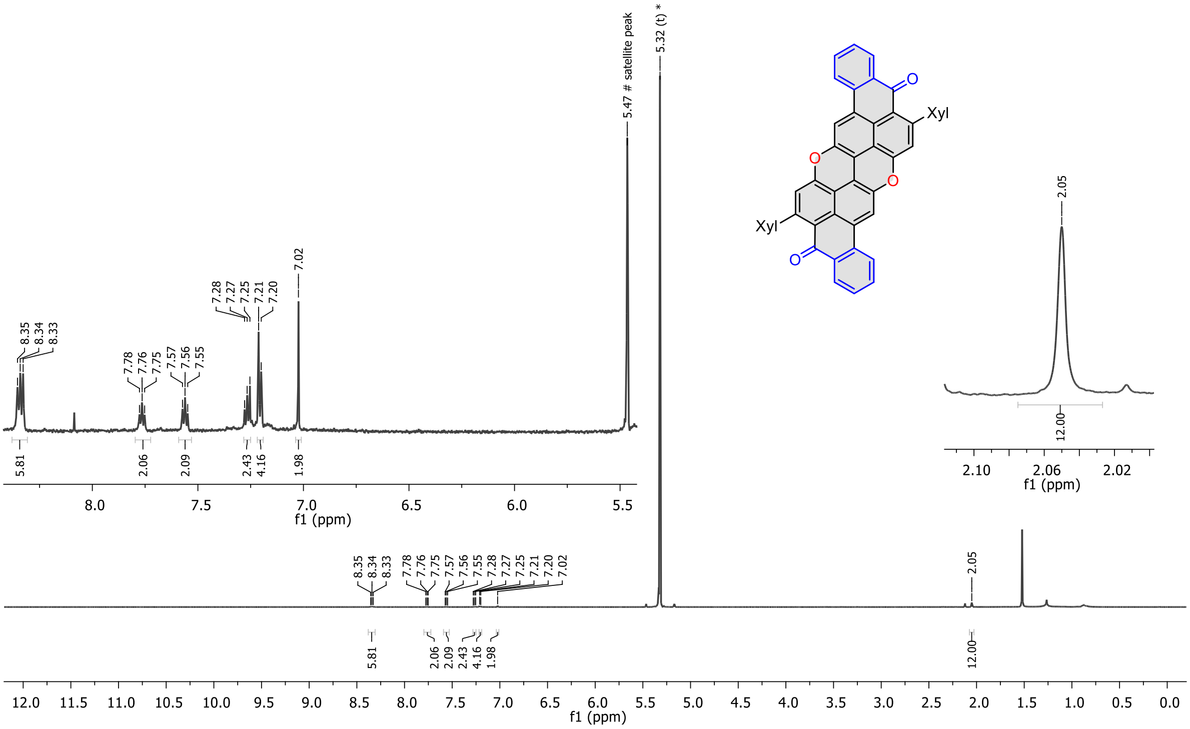

Figure S32. ^{1}H NMR (600 MHz, *CD_2Cl_2) spectrum of **2**.

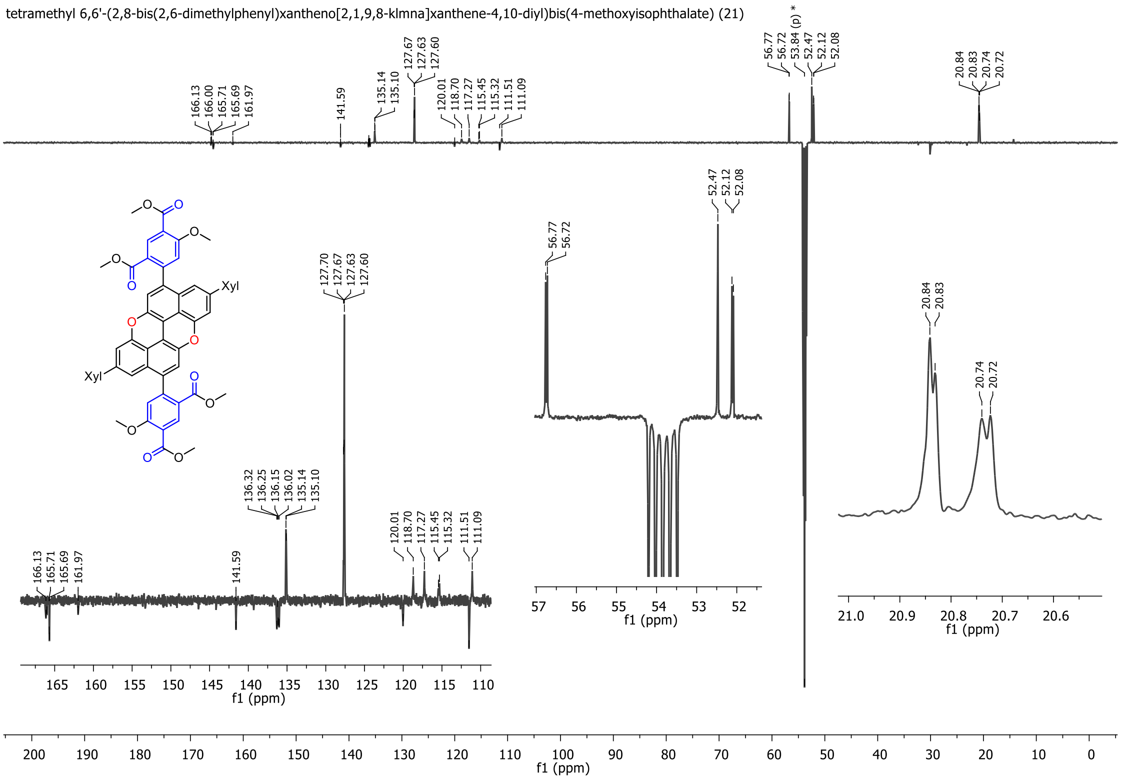

Figure S33. ^{13}C NMR (151 MHz, *CD_2Cl_2) spectrum of **21**.

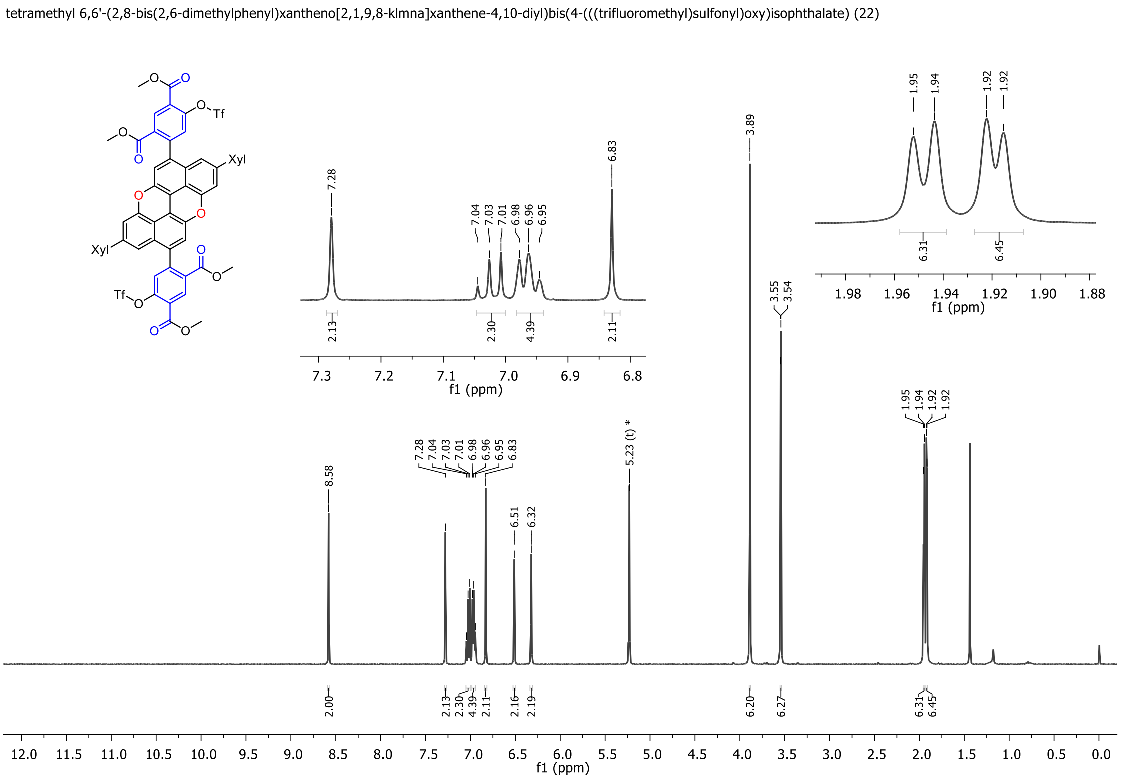

Figure S34. ^{1}H NMR (400 MHz, *CD_2Cl_2) spectrum of **22**.

tetramethyl 6,6'-(2,8-bis(2,6-dimethylphenyl)xantheno[2,1,9,8-klmna]xanthene-4,10-diyl)bis(4-(((trifluoromethyl)sulfonyl)oxy)isophthalate) (22)

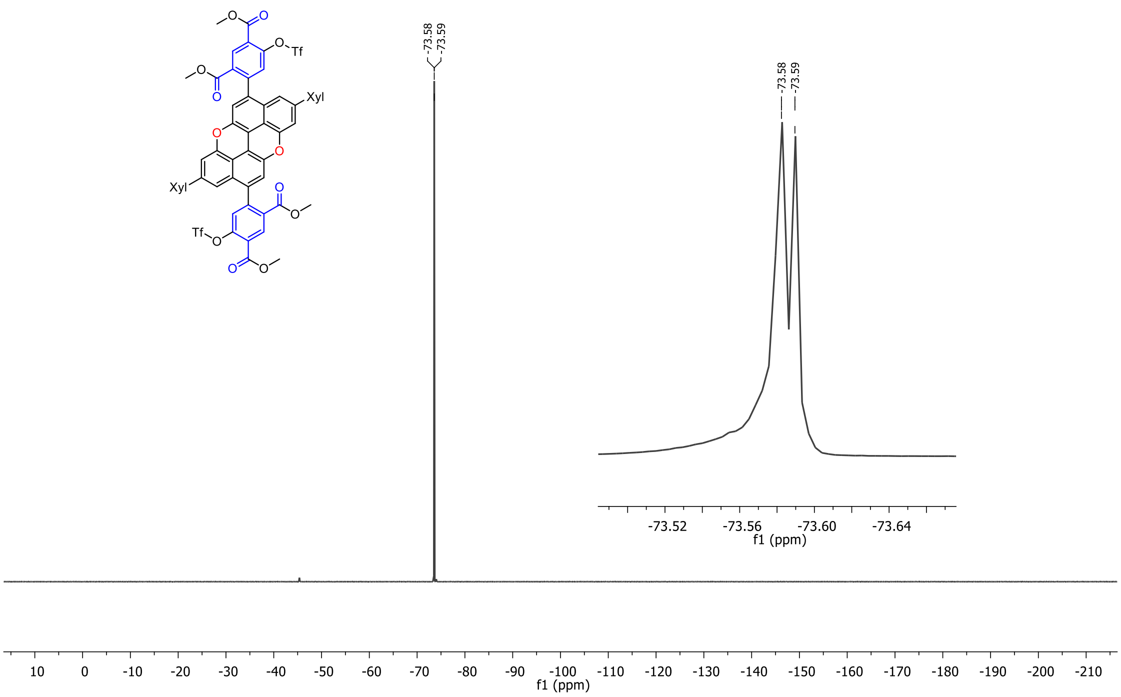

Figure S35. ^{19}F NMR (565 MHz, in CD_2Cl_2) spectrum of **22**.

tetramethyl 6,6'-(2,8-bis(2,6-dimethylphenyl)xantheno[2,1,9,8-klmna]xanthene-4,10-diyl)bis(4-(((trifluoromethyl)sulfonyl)oxy)isophthalate) (22)

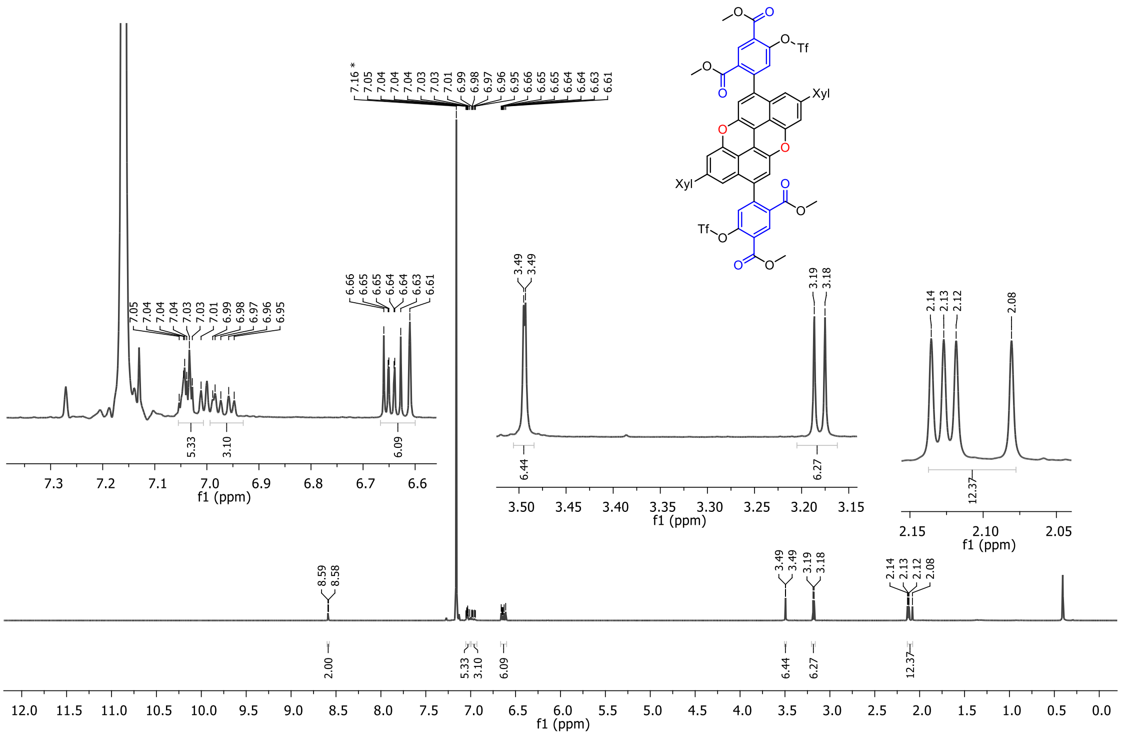

Figure S36. ^{1}H NMR (700 MHz, *C_6D_6) spectrum of **22**.

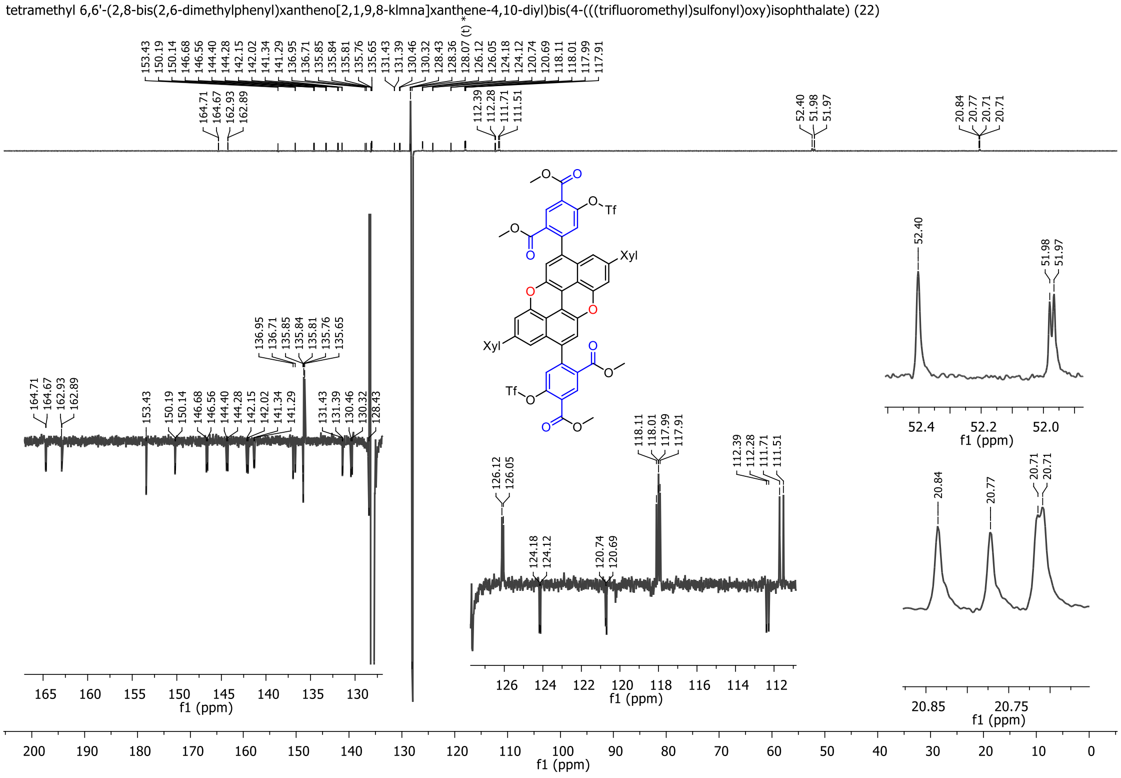

Figure S37. ^{13}C NMR (176 MHz, *C_6D_6) spectrum of **22**.

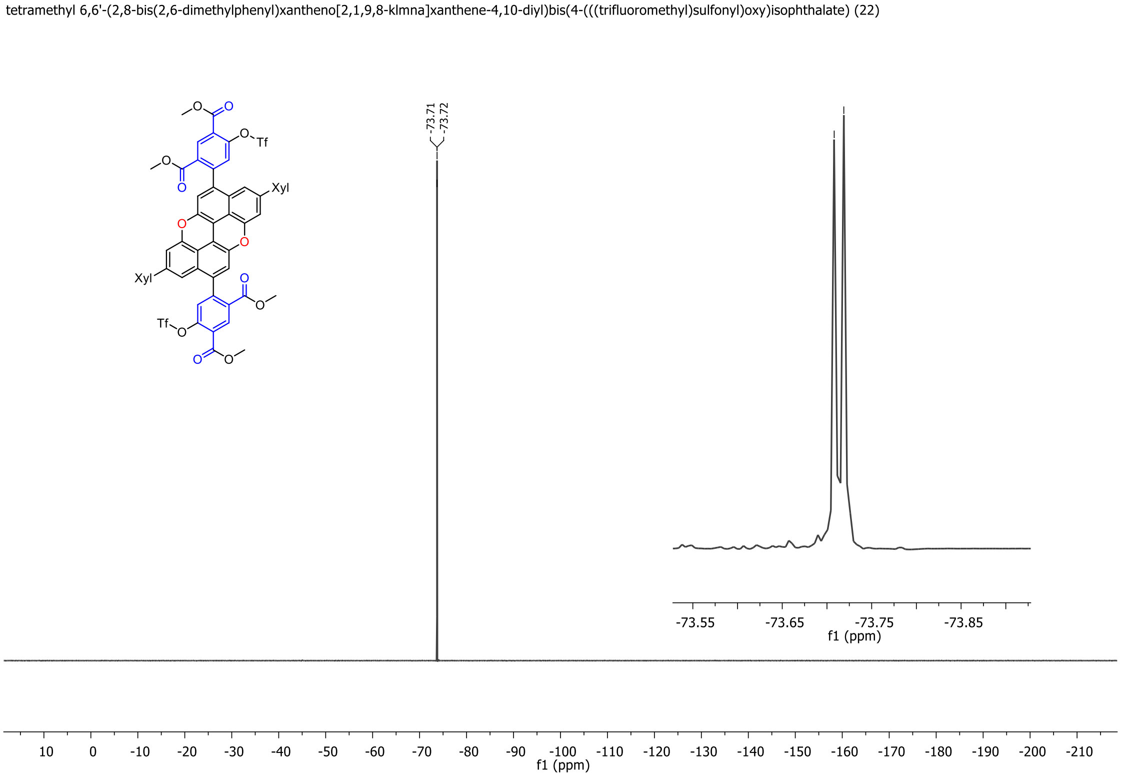

Figure S38. ^{19}F NMR (659 MHz, in C_6D_6) spectrum of **22**.

tetramethyl 6,6'-(2,8-bis(2,6-dimethylphenyl)xantheno[2,1,9,8-klmna]xanthene-4,10-diyl)bis(4-(2,8-bis(2,6-dimethylphenyl)xantheno[2,1,9,8-klmna]xanthen-4-yl)isophthalate) (23)

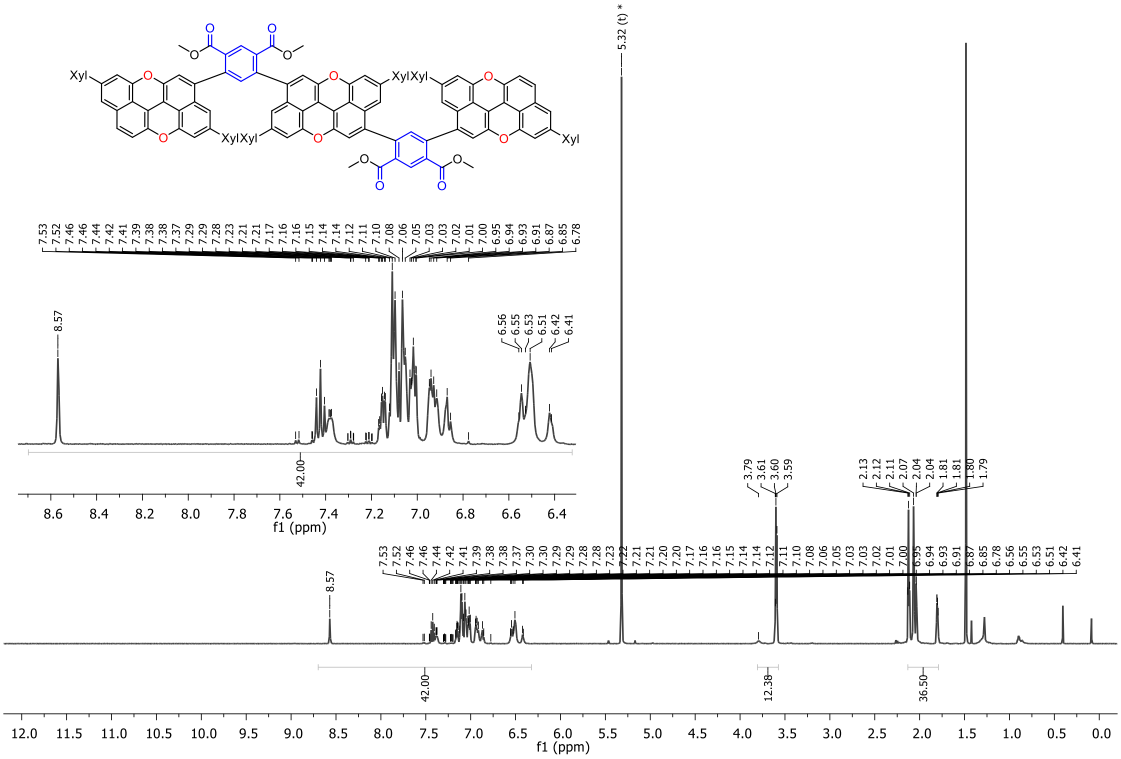

Figure S39. ^{1}H NMR (600 MHz, *CD_2Cl_2/CS_2 5:1) spectrum of **23**.

dimethyl 2-(2,8-bis(2,6-dimethylphenyl)xantheno[2,1,9,8-klmna]xanthen-4-yl)-5-methoxyterephthalate (24)

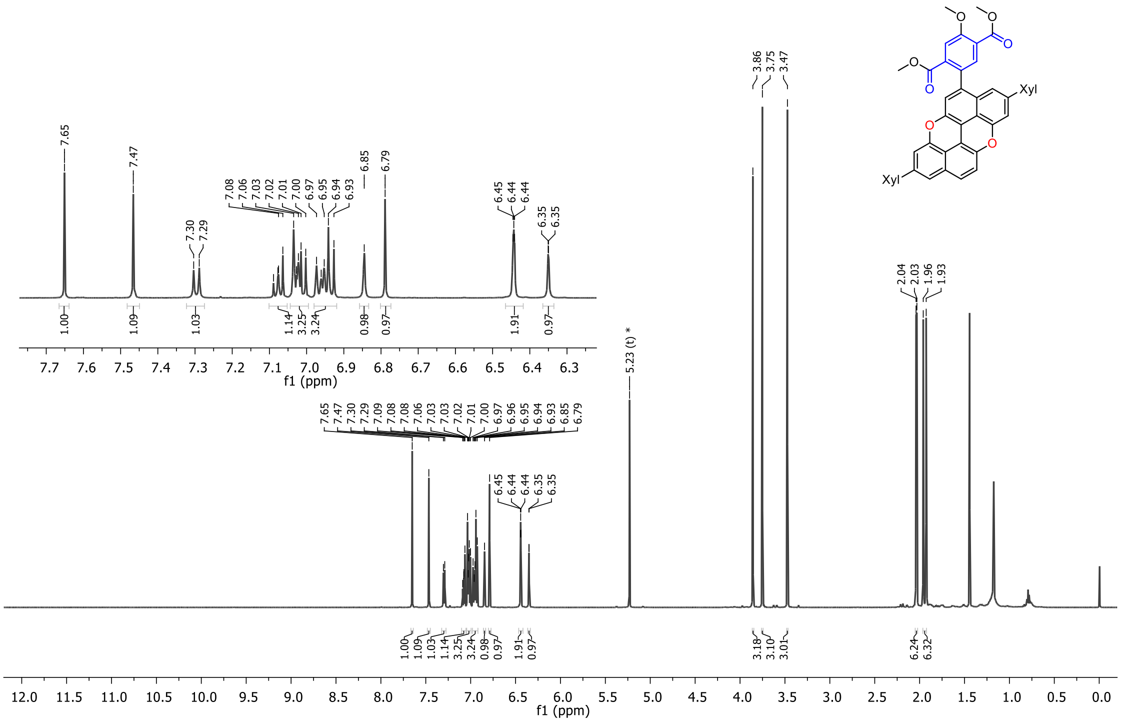

Figure S40. ^{1}H NMR (600 MHz, *CD_2Cl_2) spectrum of **24**.

dimethyl 2-(2,8-bis(2,6-dimethylphenyl)xantheno[2,1,9,8-klmna]xanthen-4-yl)-5-methoxyterephthalate (24)

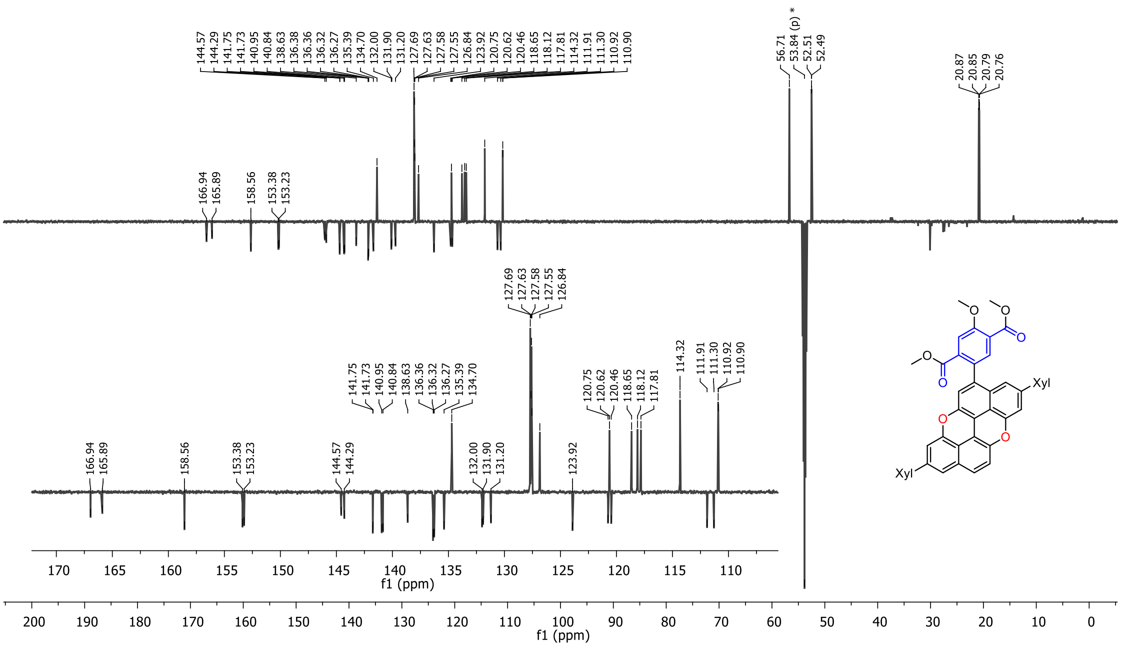

Figure S41. ^{13}C NMR (151 MHz, *CD_2Cl_2) spectrum of **24**.

dimethyl 2-(2,8-bis(2,6-dimethylphenyl)xantheno[2,1,9,8-klmna]xanthen-4-yl)-5-(((trifluoromethyl)sulfonyl)oxy)terephthalate (25)

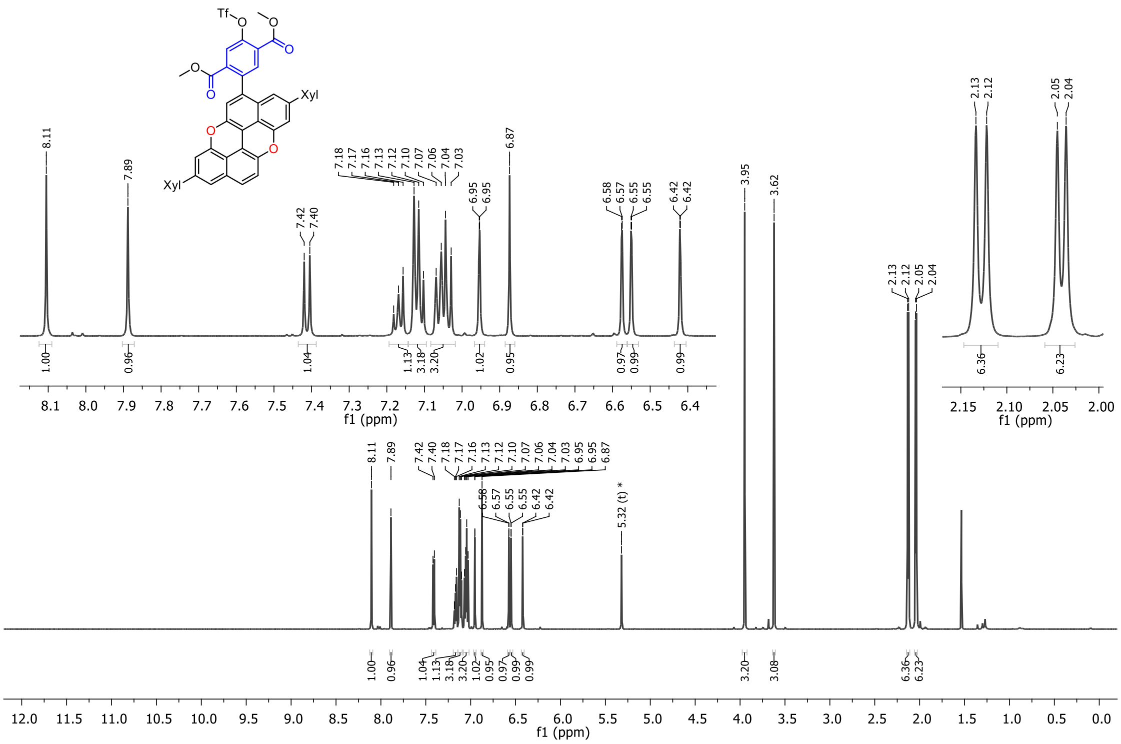

Figure S42. ^{1}H NMR (600 MHz, *CD_2Cl_2) spectrum of **25**.

dimethyl 2-(2,8-bis(2,6-dimethylphenyl)xantheno[2,1,9,8-klmna]xanthen-4-yl)-5-(((trifluoromethyl)sulfonyl)oxy)terephthalate (25)

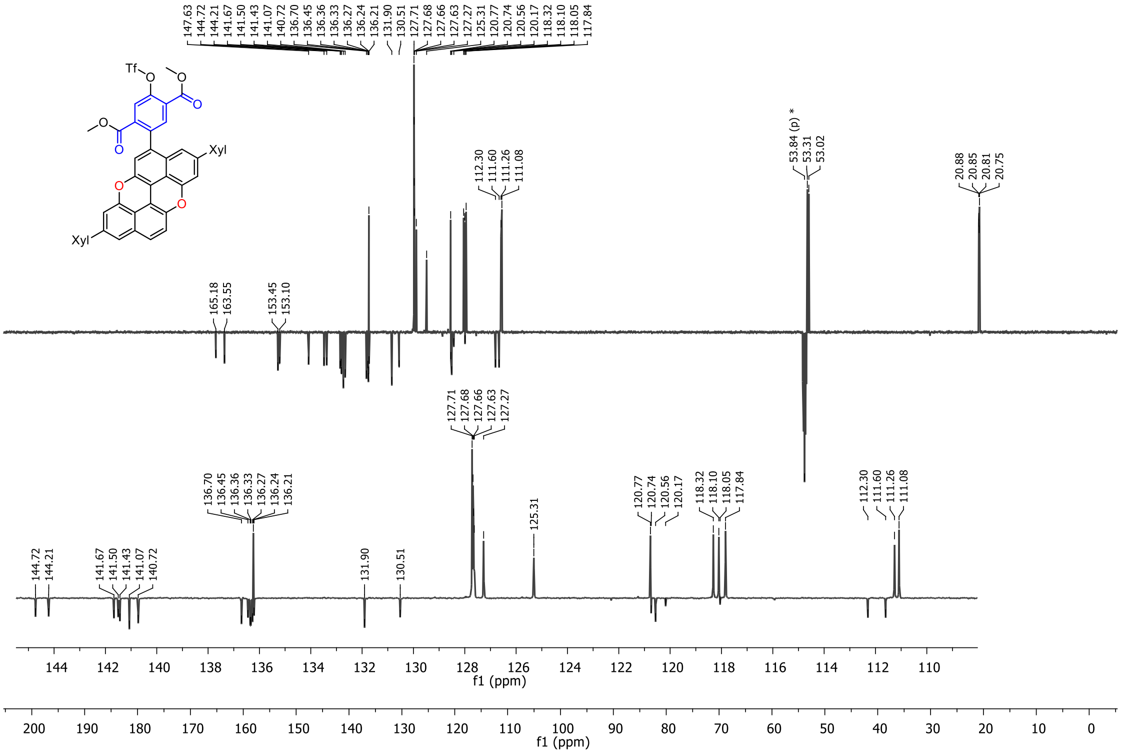

Figure S43. ^{13}C NMR (151 MHz, *CD_2Cl_2) spectrum of **25**.

dimethyl 2-(2,8-bis(2,6-dimethylphenyl)xantheno[2,1,9,8-klmna]xanthen-4-yl)-5-(((trifluoromethyl)sulfonyl)oxy)terephthalate (25)

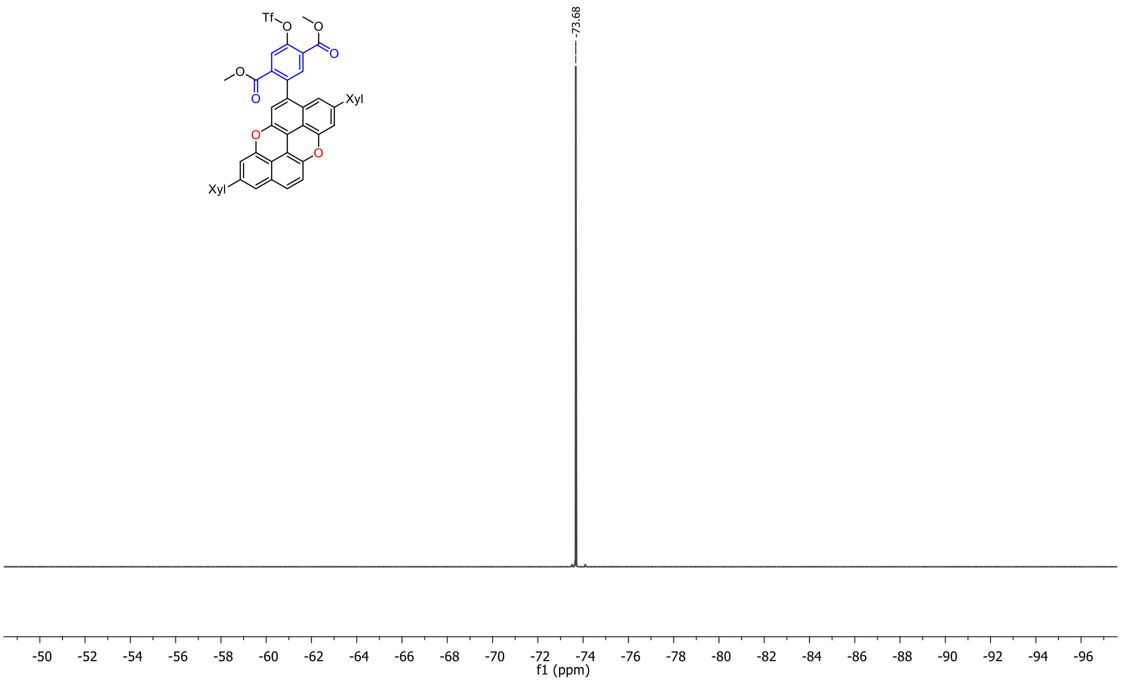

Figure S44. ^{19}F NMR (565 MHz, in CD_2Cl_2) spectrum of **25**.

tetramethyl 5,5'-(2,8-bis(2,6-dimethylphenyl)xantheno[2,1,9,8-klmna]xanthene-4,10-diyl)bis(2-(2,8-bis(2,6-dimethylphenyl)xantheno[2,1,9,8-klmna]xanthen-4-yl)terephthalate) (26)

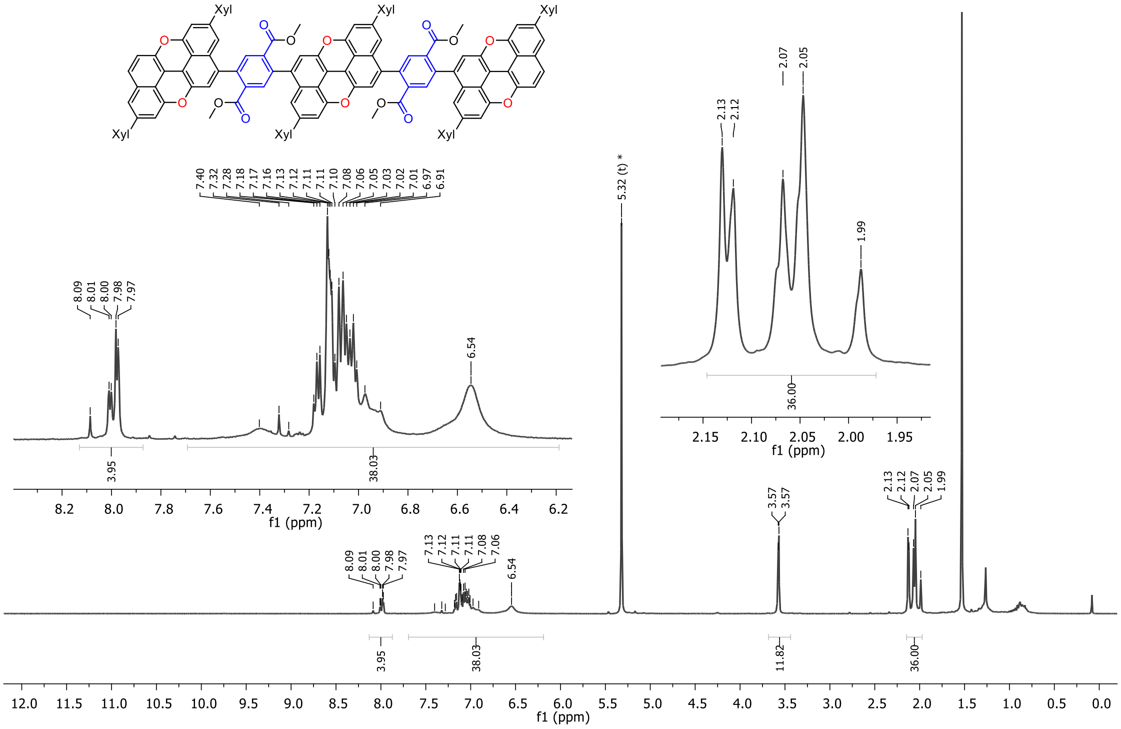

Figure S45. ^{1}H NMR (600 MHz, *CD_2Cl_2) spectrum of **26**.

4,13-bis(2,6-dimethylphenyl)-12H-anthra[9,1,2-hij]benzo[8,1]isochromeno[5,4,3-cde]isochromen-12-one (1)

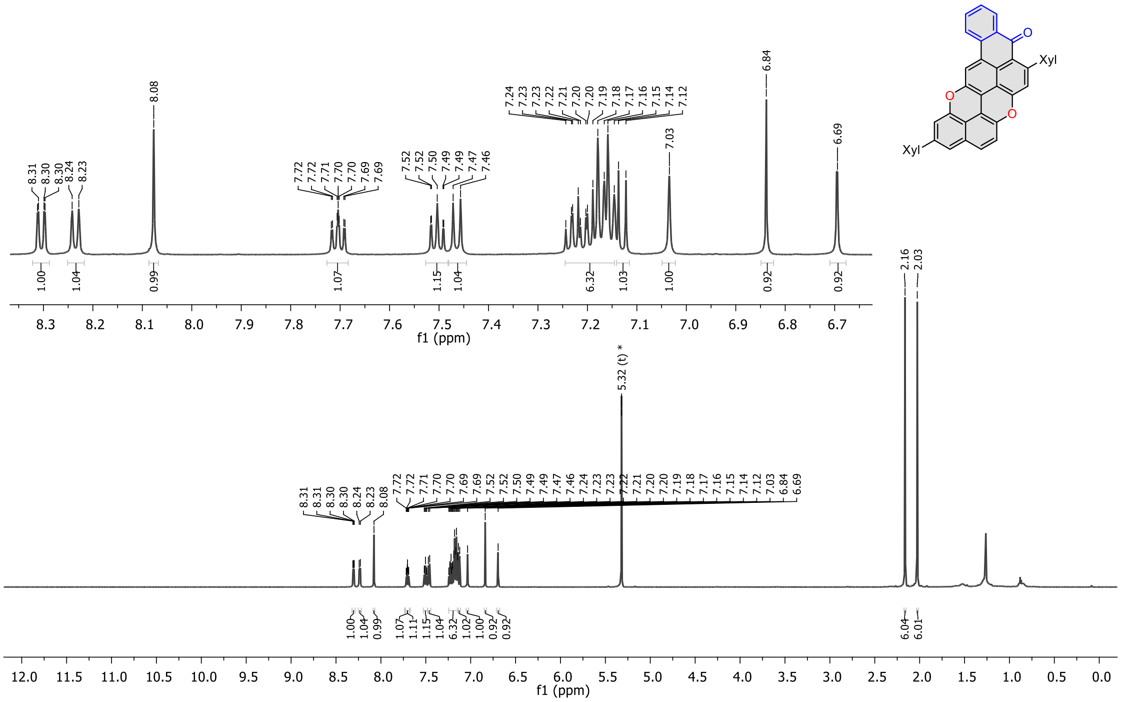

Figure S46. ^{1}H NMR (600 MHz, *CD_2Cl_2) spectrum of **1**.

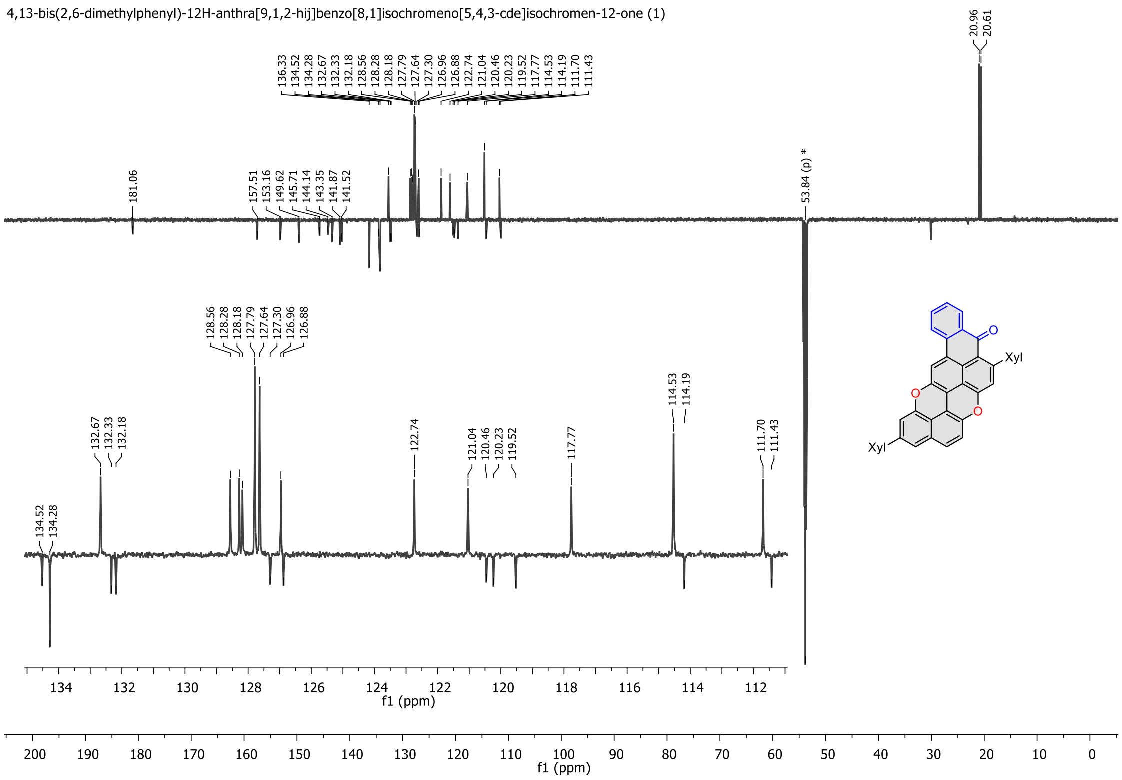

Figure S47. ^{13}C NMR (151 MHz, $^{*}CD_2Cl_2$) spectrum of **1**.

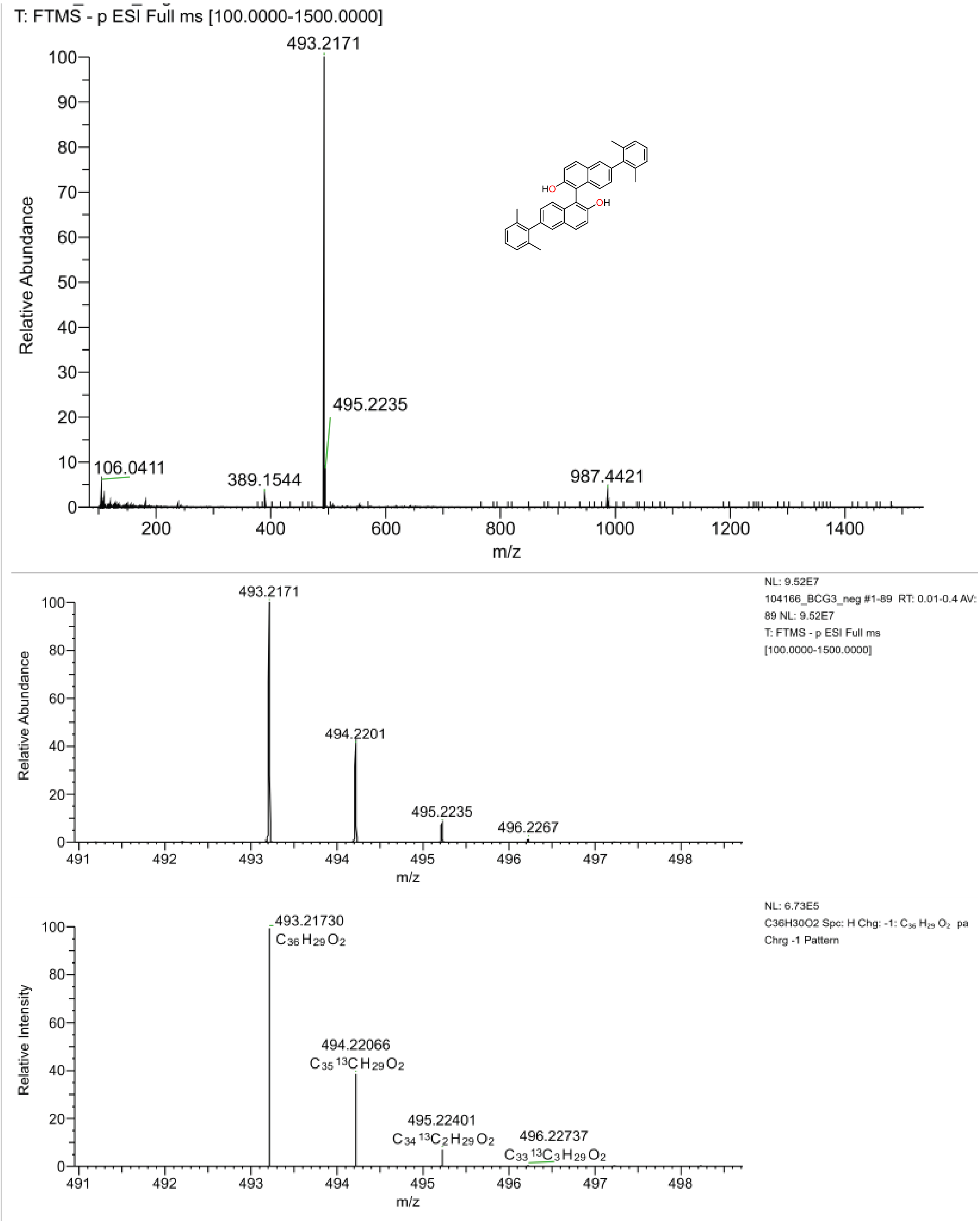

Figure S48. HRMS (ESI-orbitrap) of **7** (from top: full, zoom and simulated spectra).

Comment sample out of Toluene; 34% Laserpower

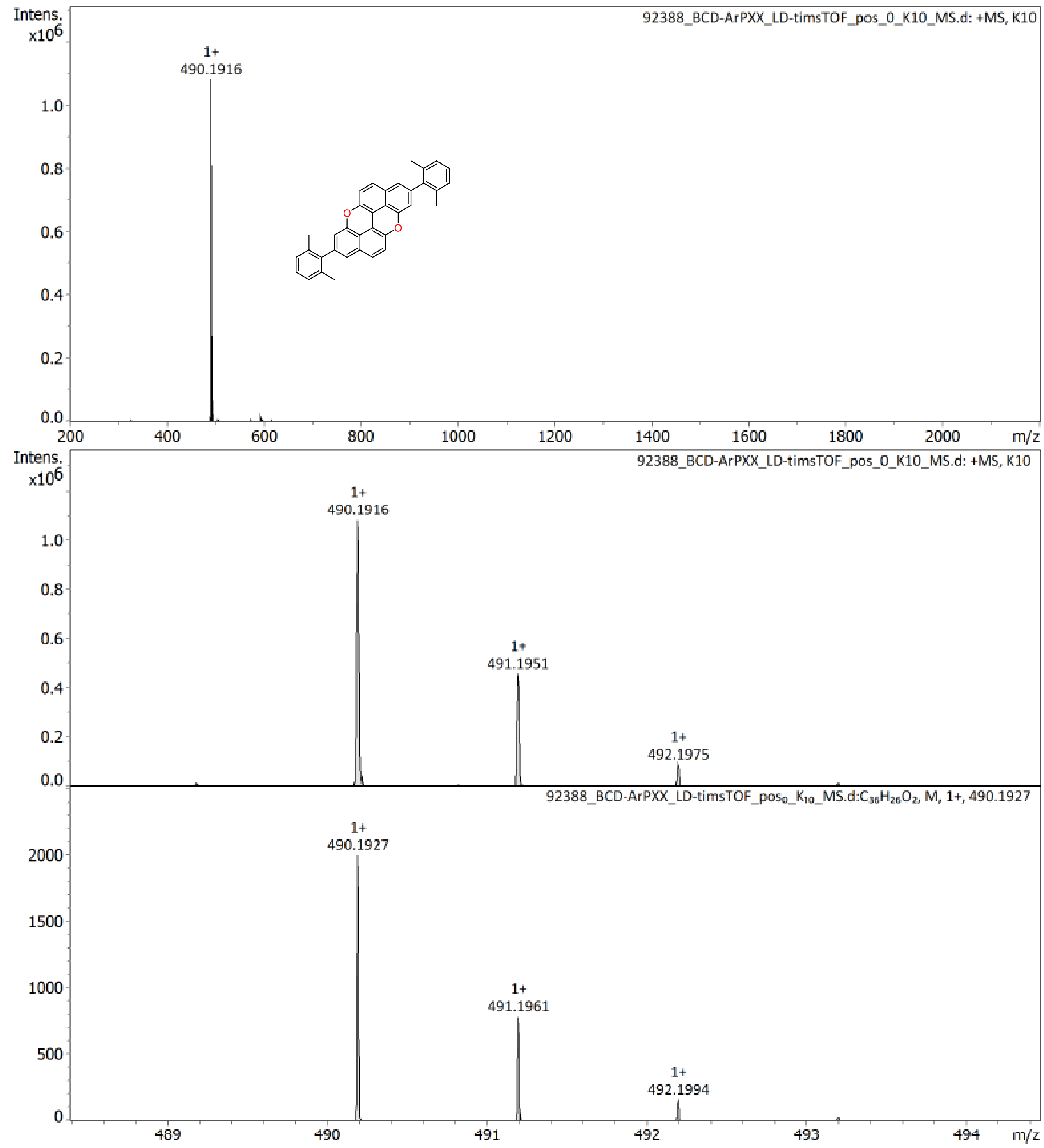

Figure S49. HRMS (MALDI-timsTOF) of **8** (from top: full, zoom and simulated spectra).

Comment sample out of Toluene; 48% Laserpower

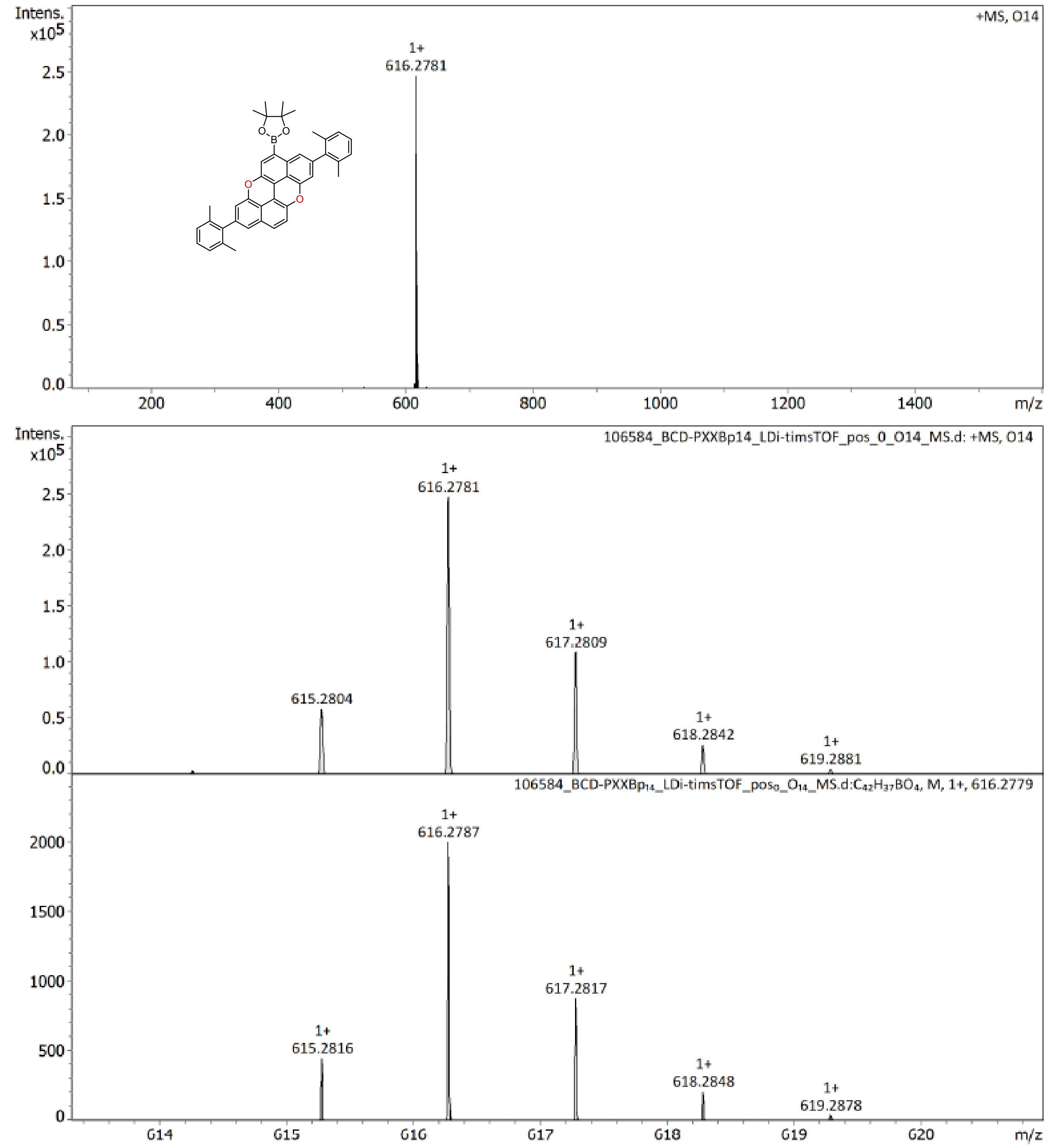

Figure S50. HRMS (LD-timsTOF) of **9** (from top: full, zoom and simulated spectra).

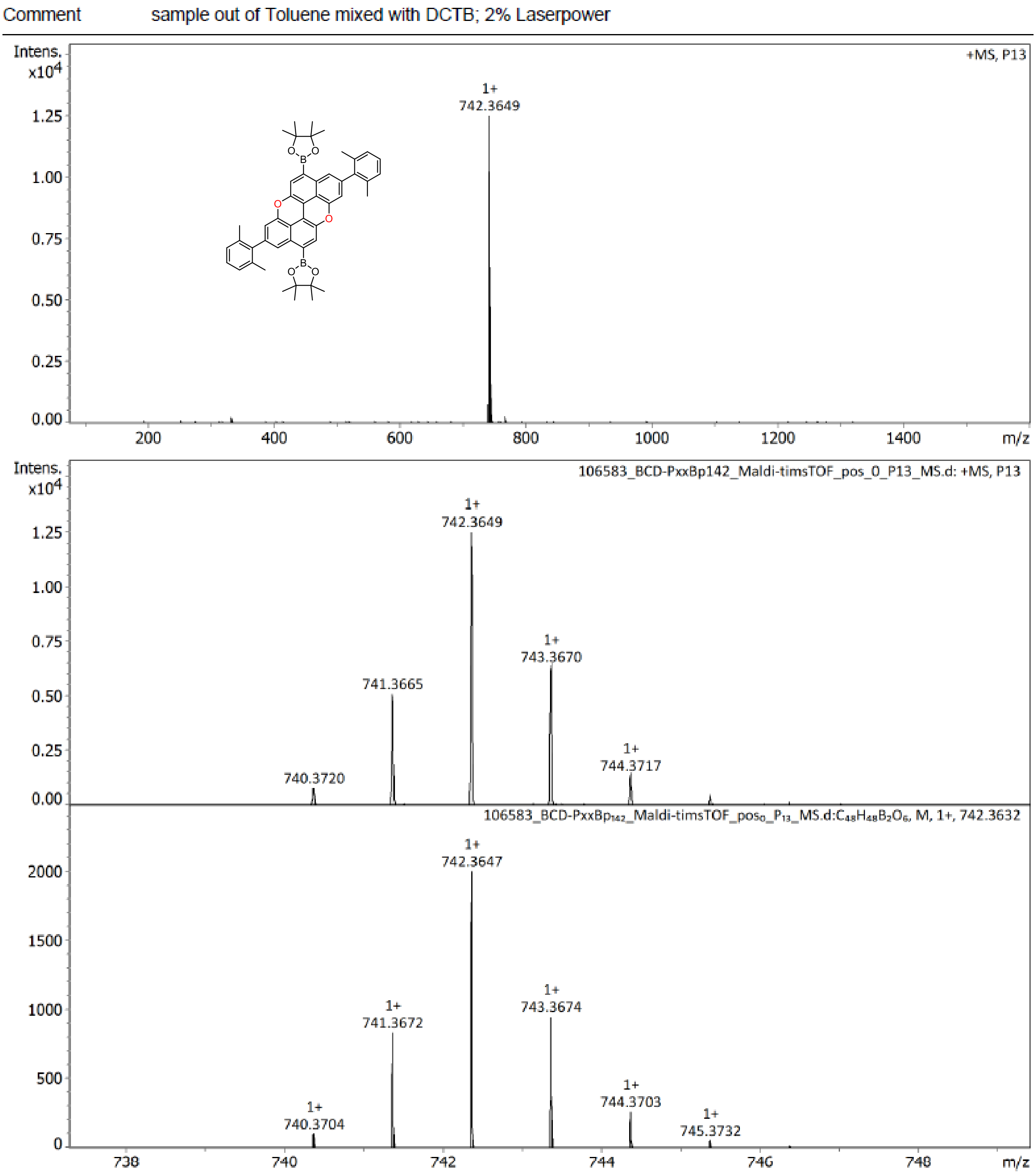

Figure S51. HRMS (MALDI-timsTOF) of **10** (from top: full, zoom and simulated spectra).

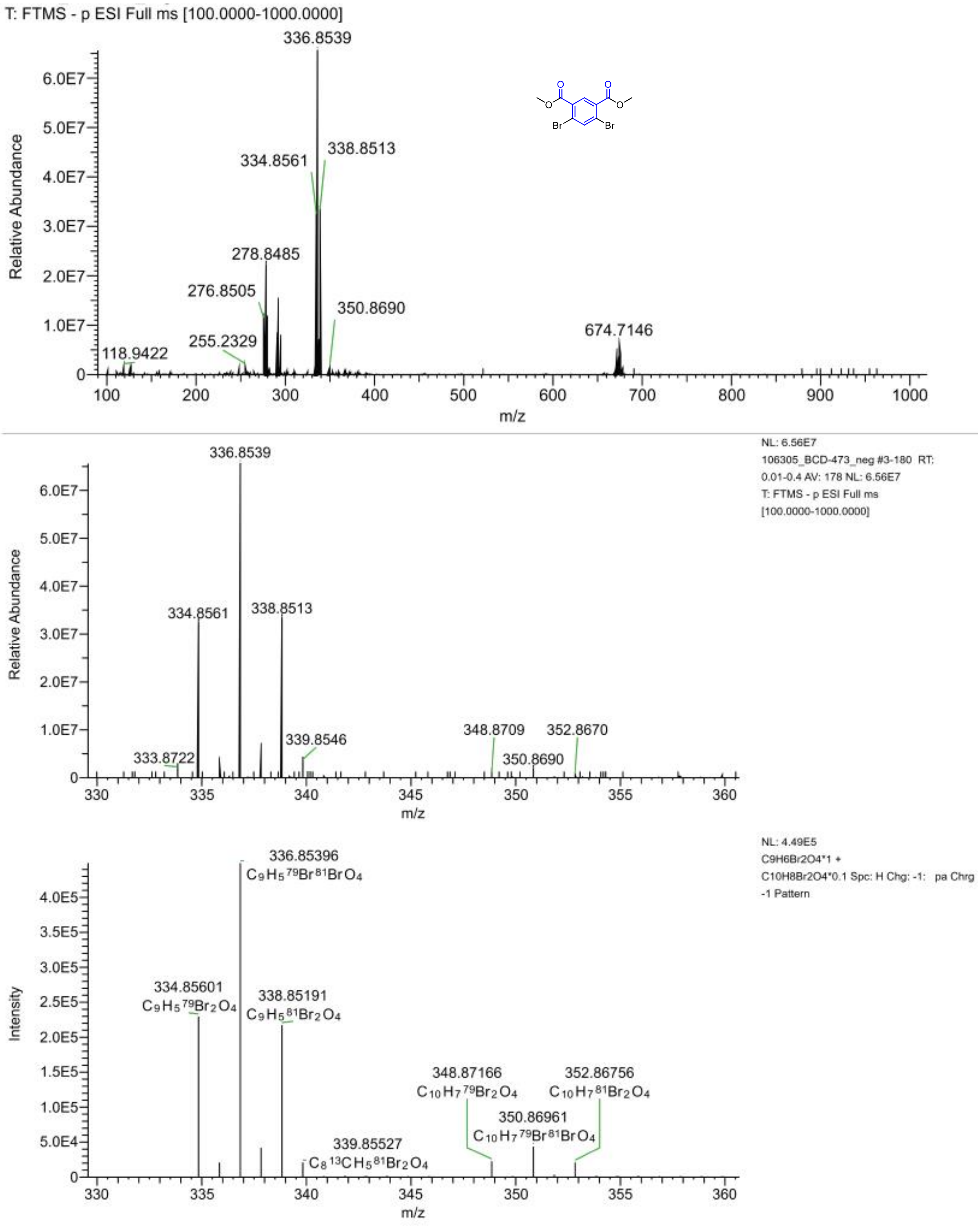

Figure S52. HRMS (ESI-orbitrap) of **11** (from top: full, zoom and simulated spectra).

Sample Chromatograms

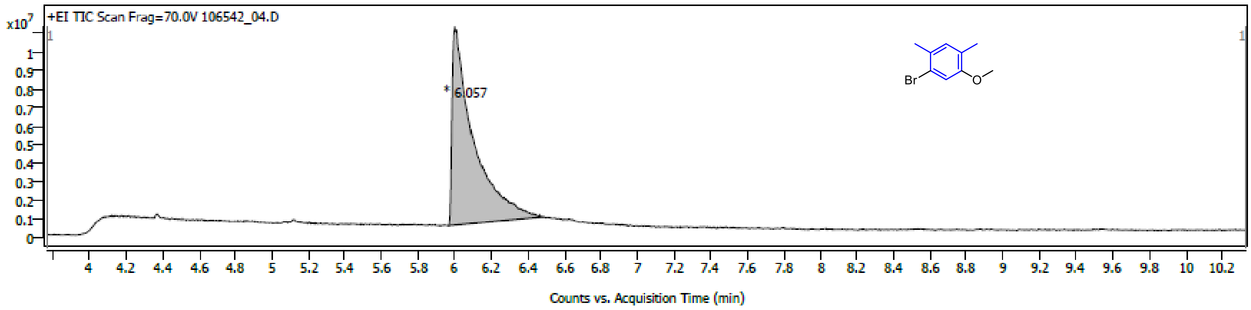

Chromatogram Peaks

Peak	Start	RT	End	Height	Area	Area %	SNR
1	5.963	6.057	6.483	6662915	87378016	100.00	

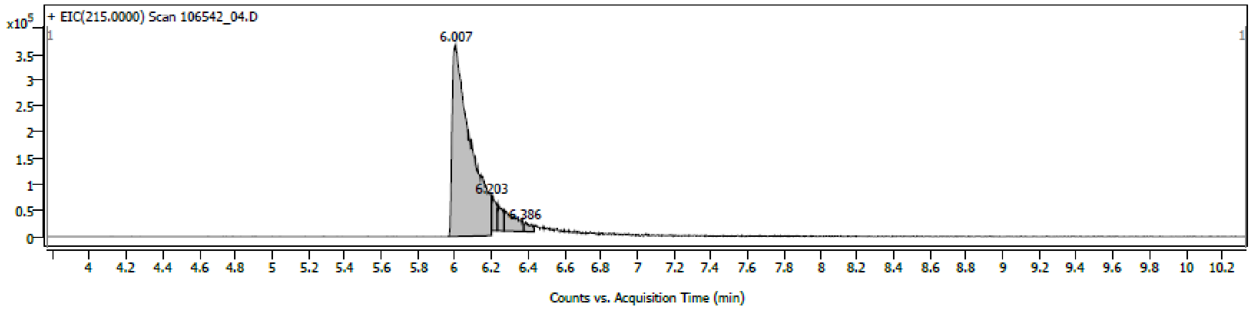

Chromatogram Peaks

Peak	Start	RT	End	Height	Area	Area %	SNR
1	5.967	6.007	6.200	366573	2570863	100.00	
2	6.200	6.203	6.233	65276	114325	4.45	
3	6.233	6.236	6.270	51104	93189	3.62	
4	6.270	6.273	6.376	38951	171097	6.66	
5	6.376	6.386	6.433	18157	45735	1.78	

Sample Spectra

+ Scan (rt: 5.947-6.343 min) Sub

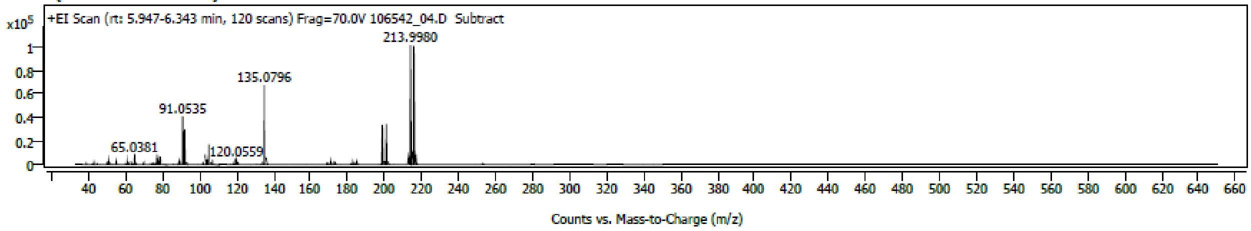

Figure S53. HRMS (GC-QTOF) of **12** (from top: chromatogram, mass spectrum).

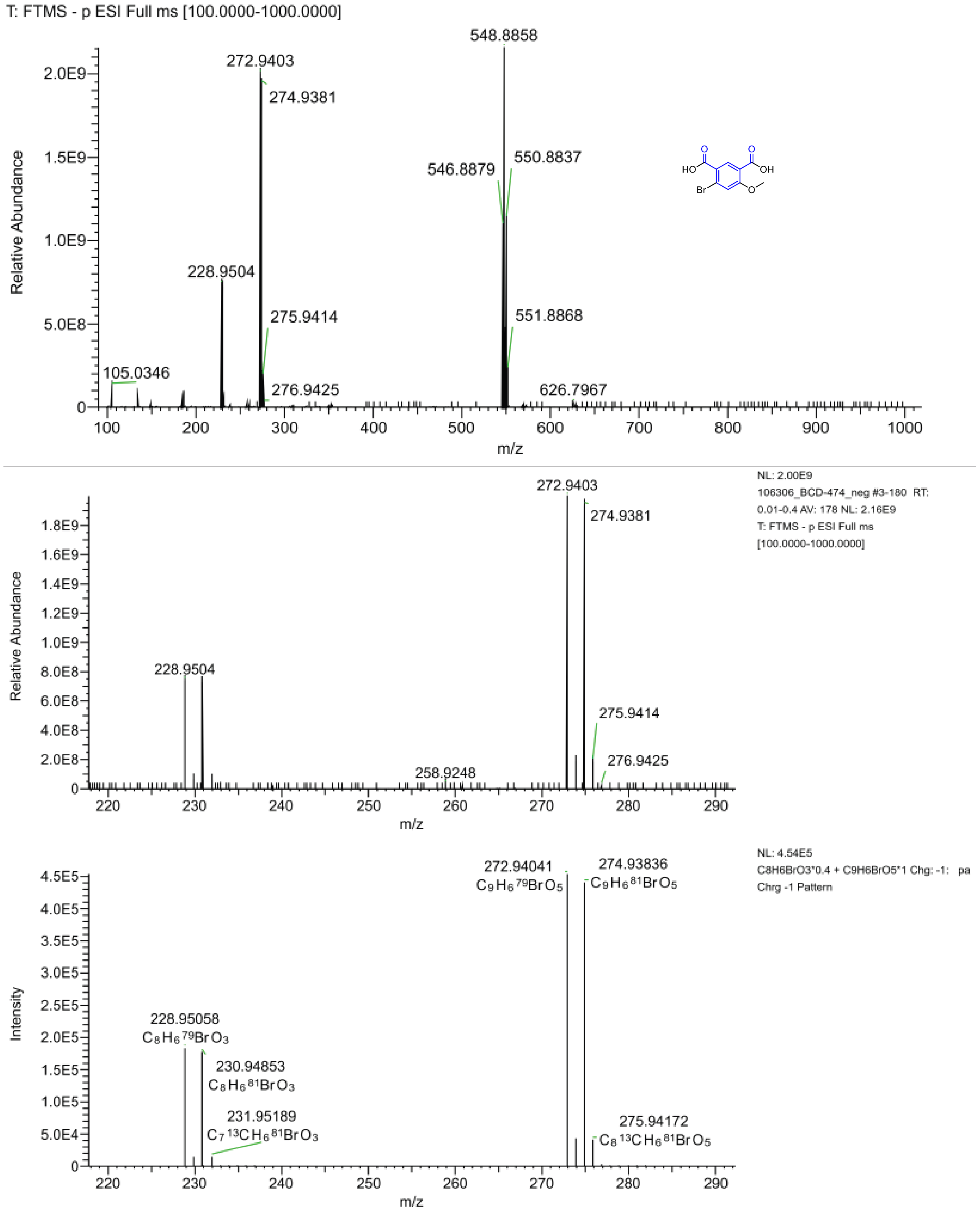

Figure S54. HRMS (ESI-orbitrap) of **13** (from top: full, zoom and simulated spectra).

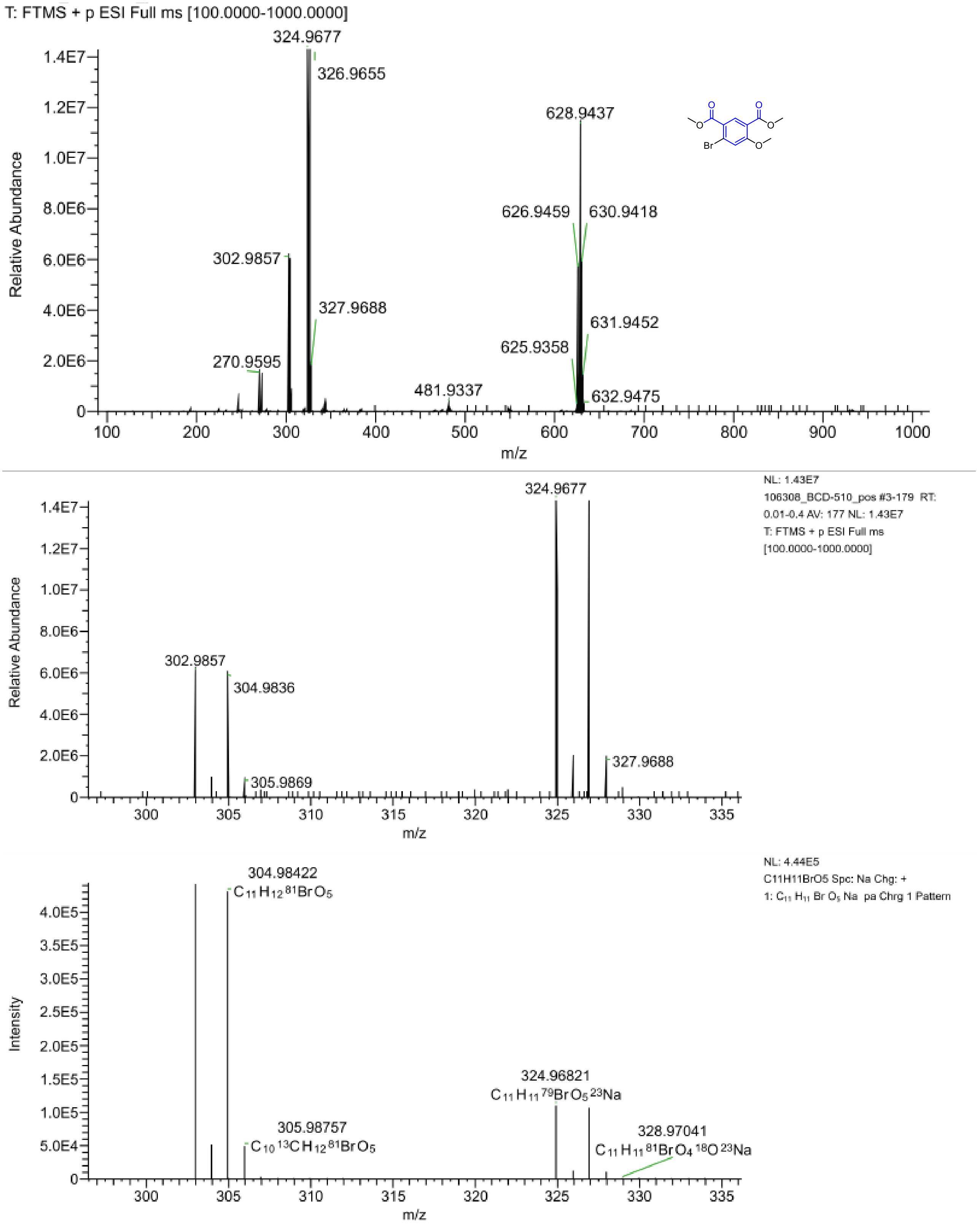

Figure S55. HRMS (ESI-orbitrap) of **14** (from top: full, zoom and simulated spectra).

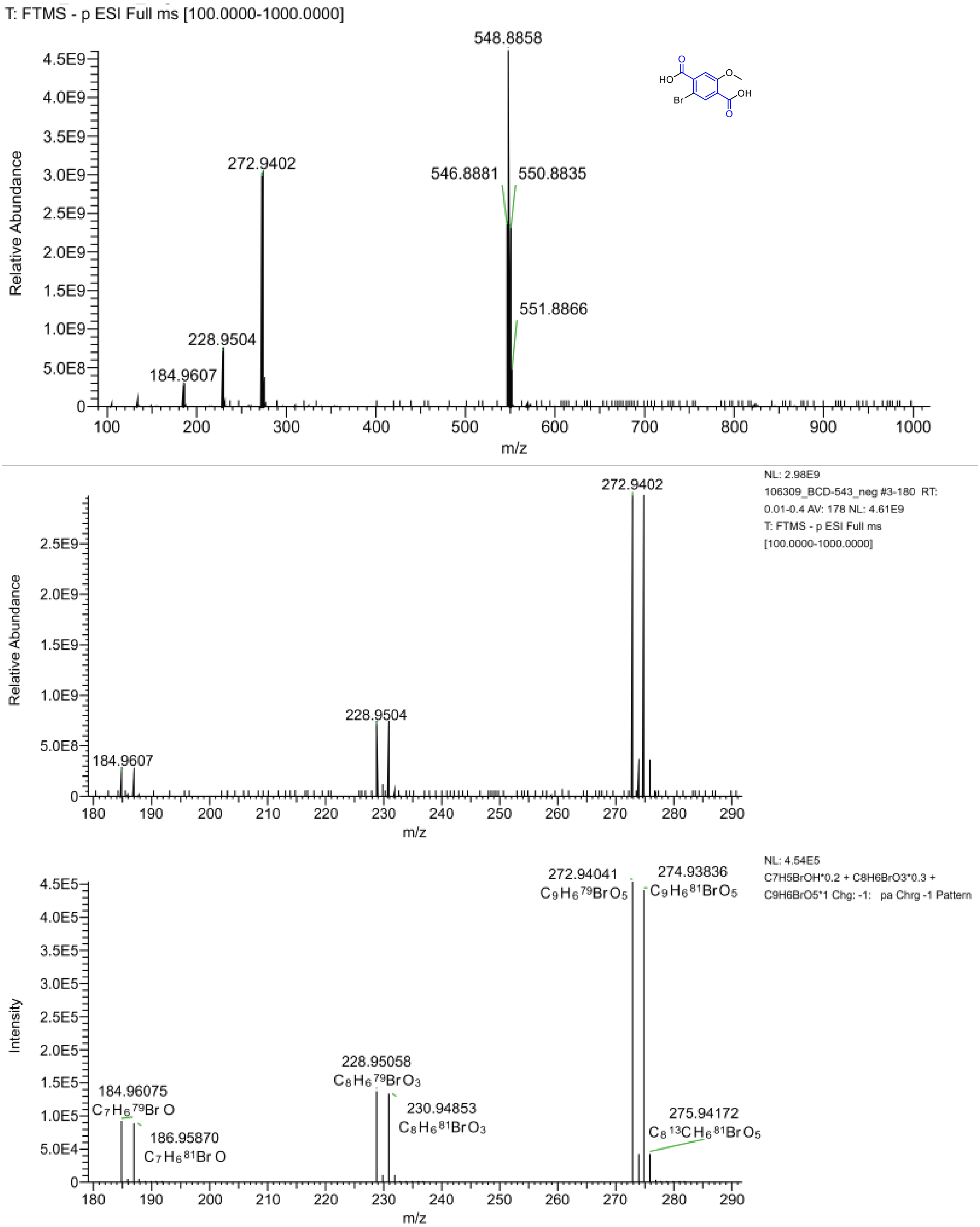

Figure S56. HRMS (ESI-orbitrap) of **15** (from top: full, zoom and simulated spectra).

Comment De Luca / Bonifazi
Ergebnis: +/- 5 ppm
ACN / MeOH + 1% H2O

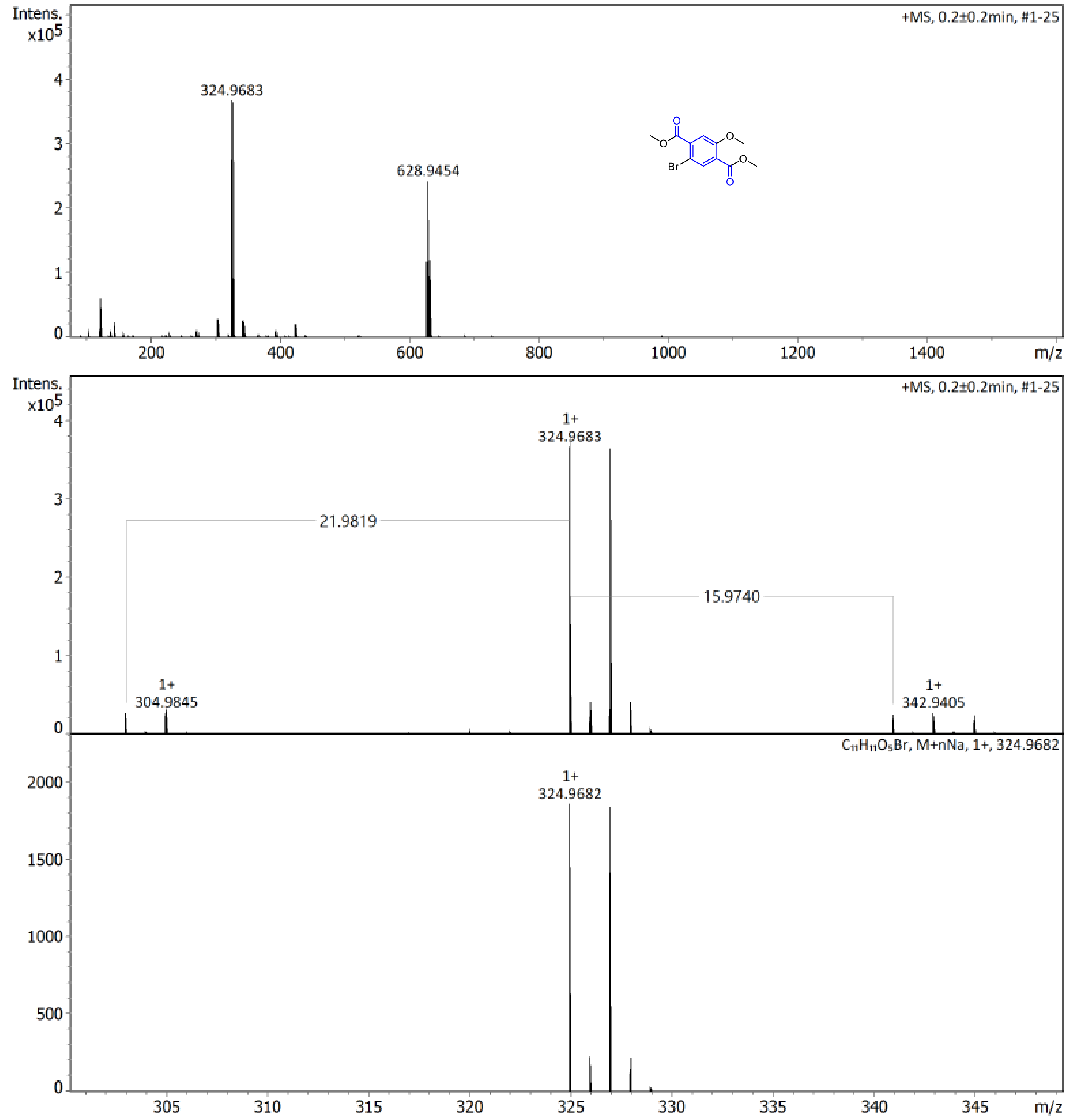

Figure S57. HRMS (ESI-timsTOF) of **16** (from top: full, zoom and simulated spectra).

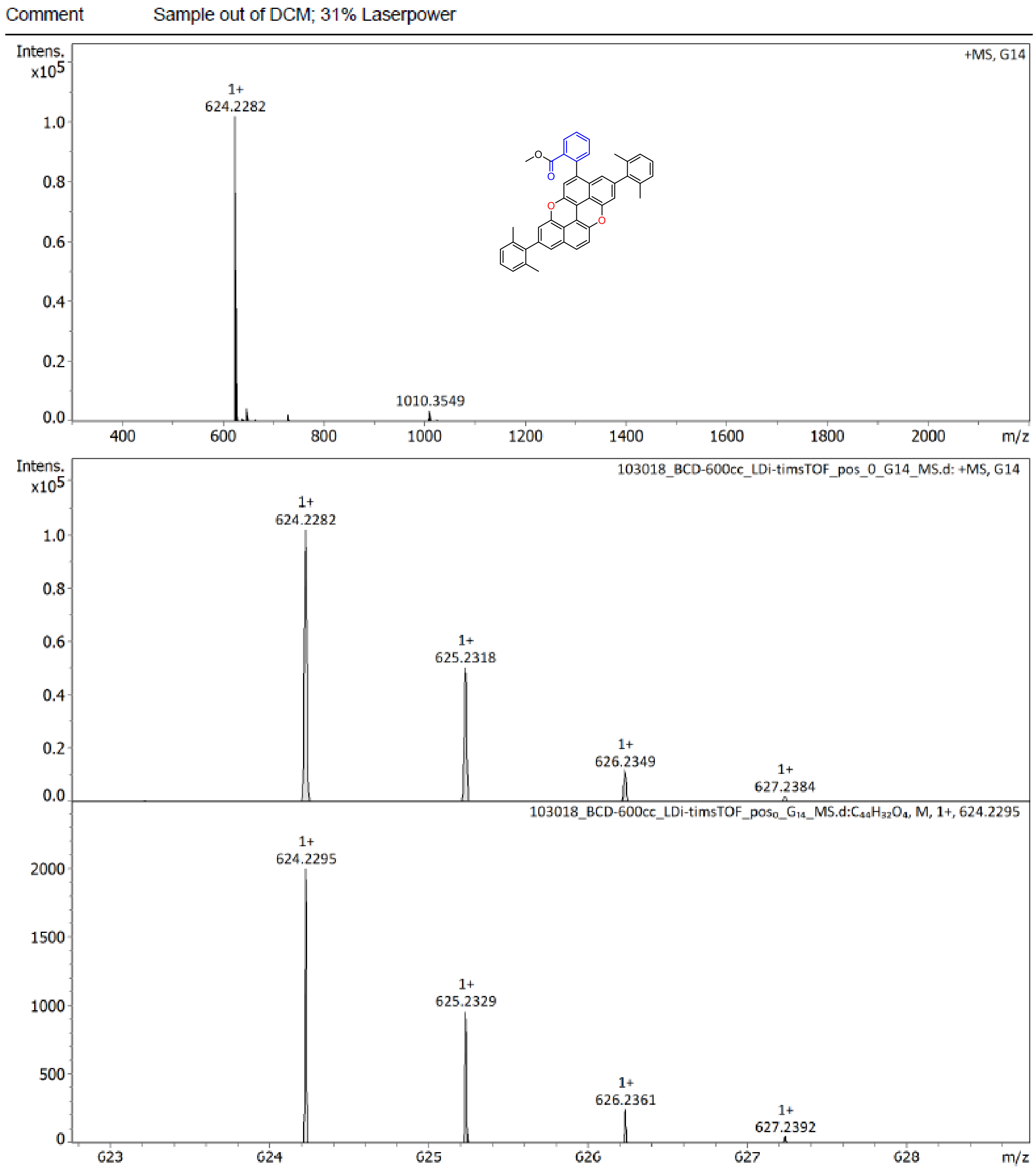

Figure S58. HRMS (LD-timsTOF) of **17** (from top: full, zoom and simulated spectra).

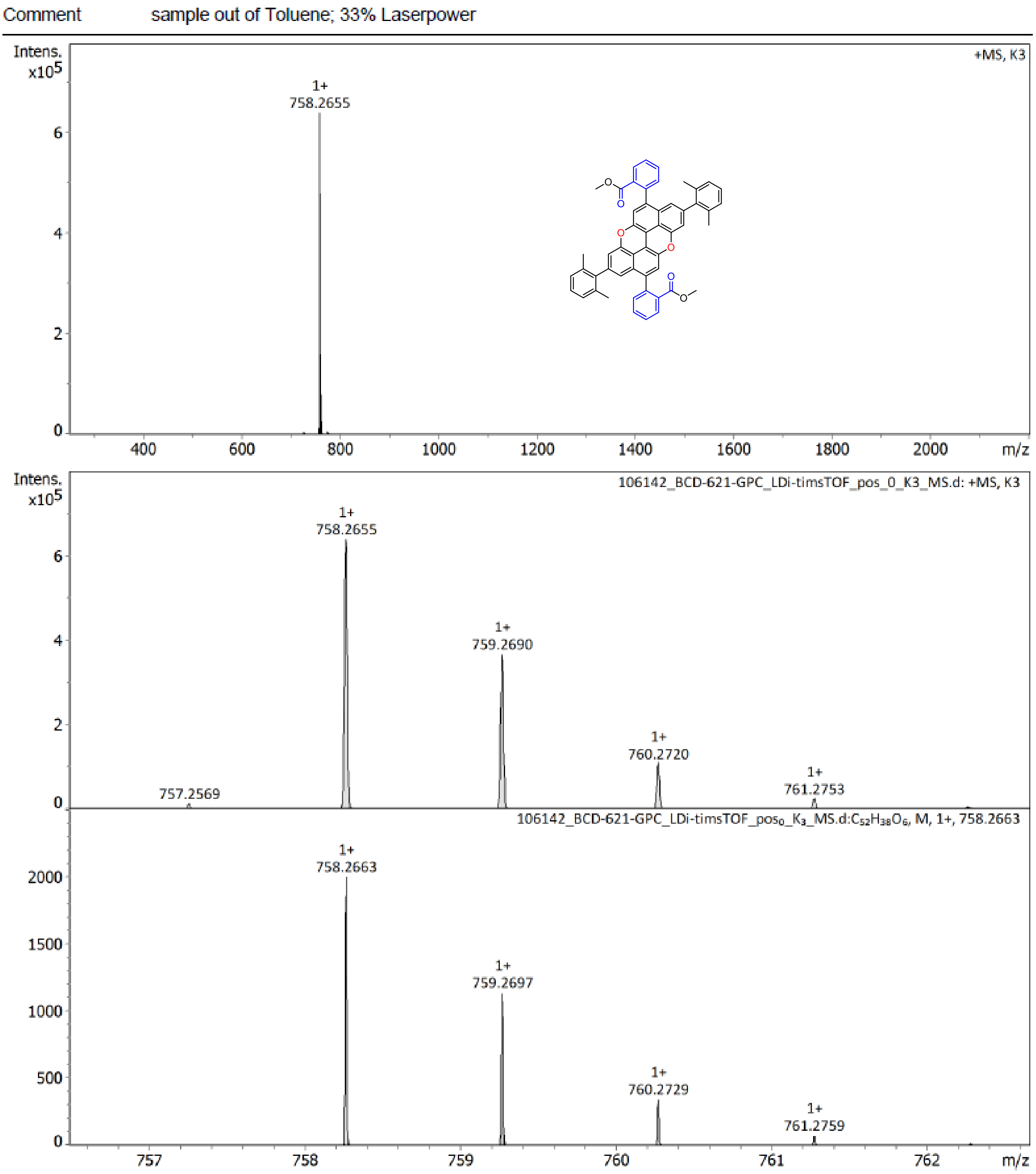

Figure S59. HRMS (LD-timsTOF) of **18** (from top: full, zoom and simulated spectra).

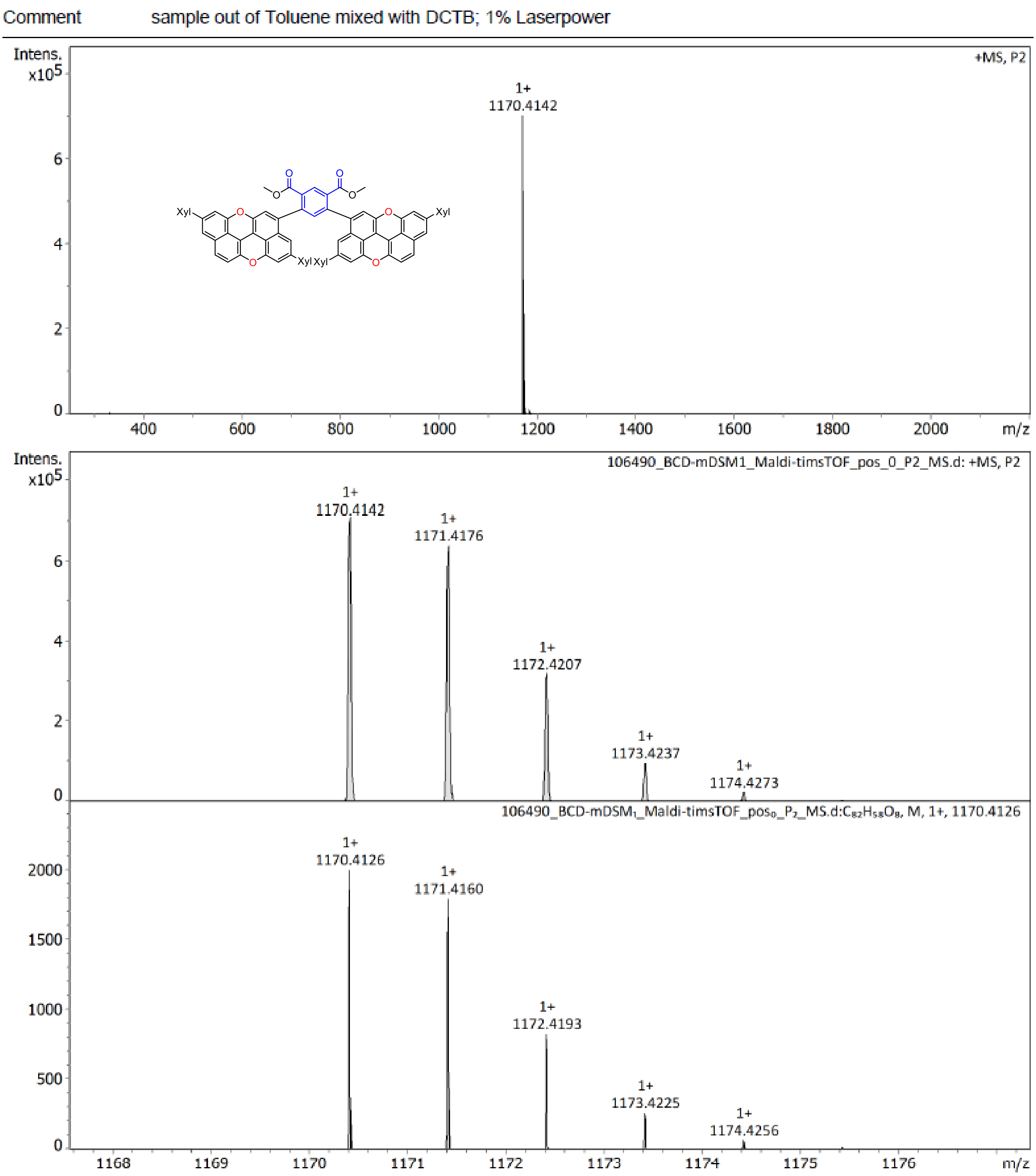

Figure S60. HRMS (MALDI-timsTOF) of **19** (from top: full, zoom and simulated spectra).

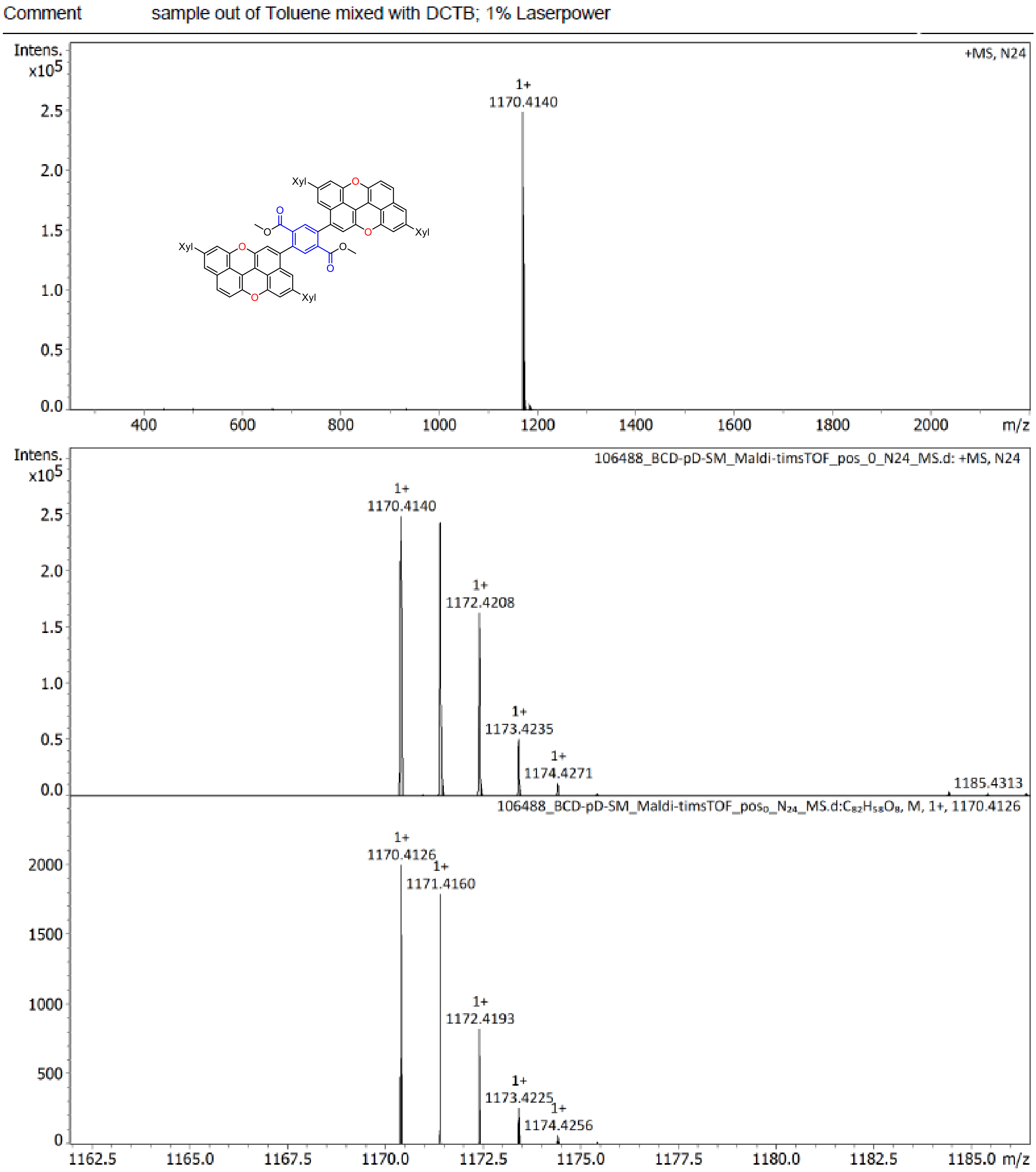

Figure S61. HRMS (MALDI-timsTOF) of **20** (from top: full, zoom and simulated spectra).

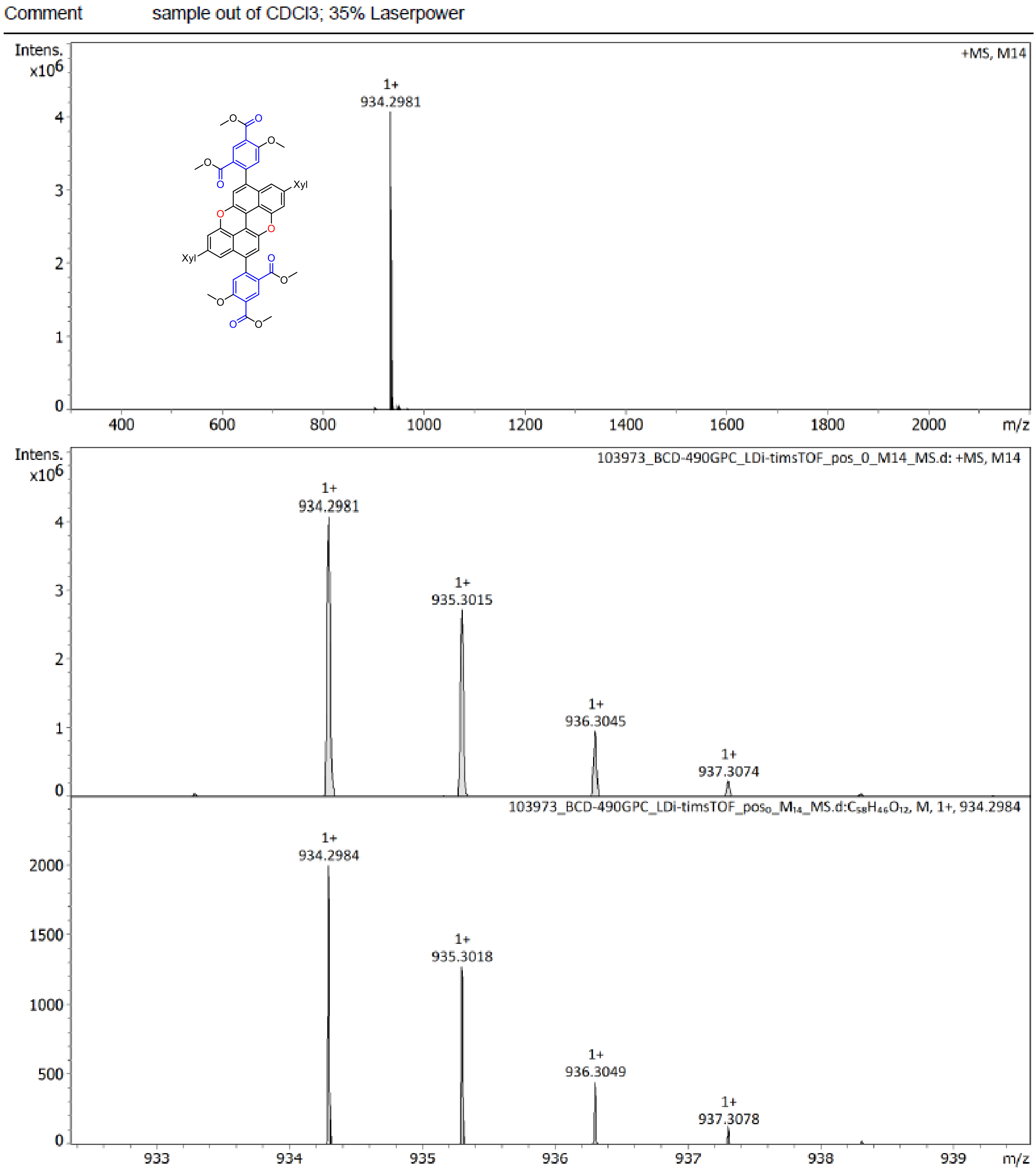

Figure S62. HRMS (LD-timsTOF) of **21** (from top: full, zoom and simulated spectra).

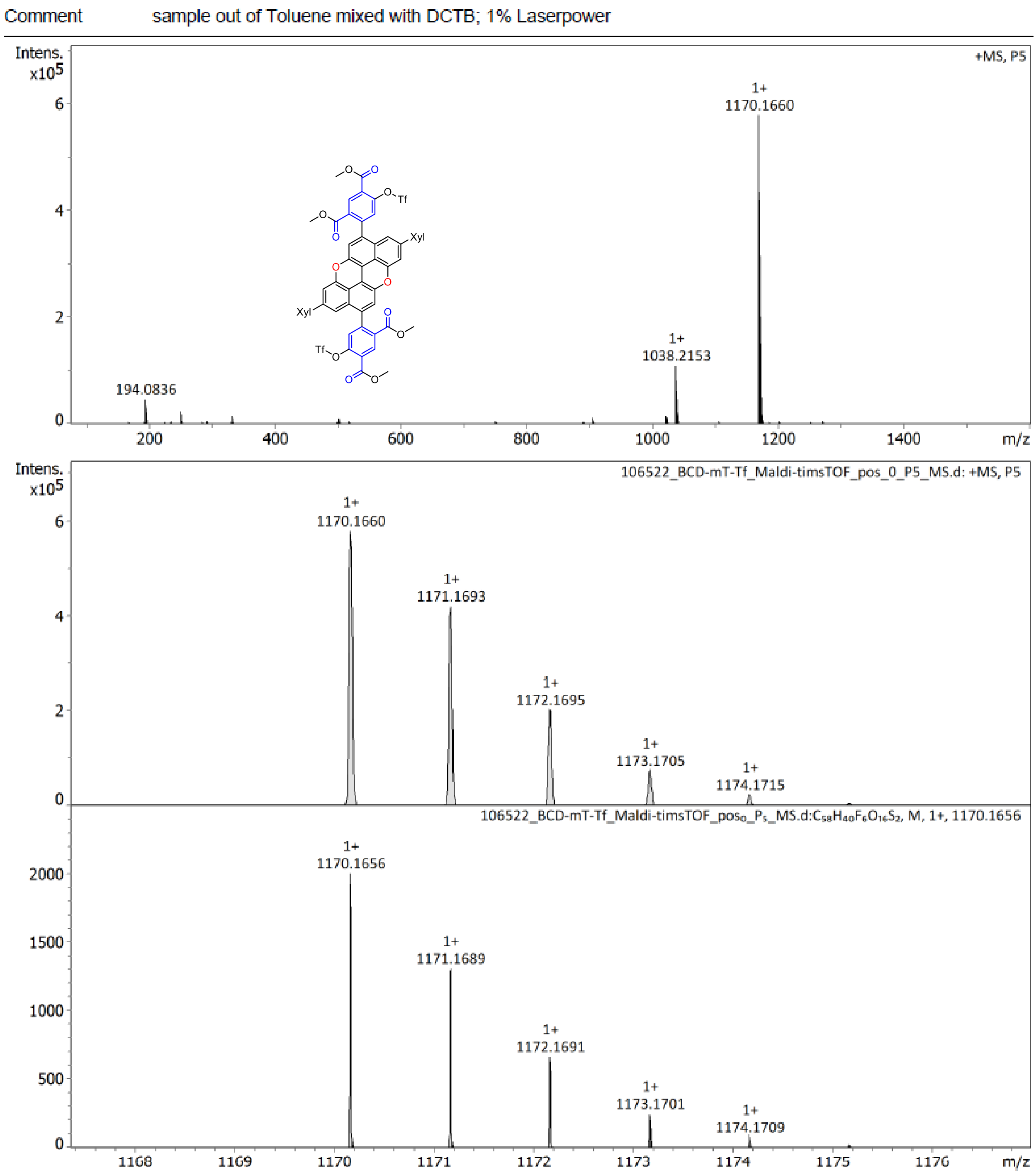

Figure S63. HRMS (MALDI-timsTOF) of **22** (from top: full, zoom and simulated spectra).

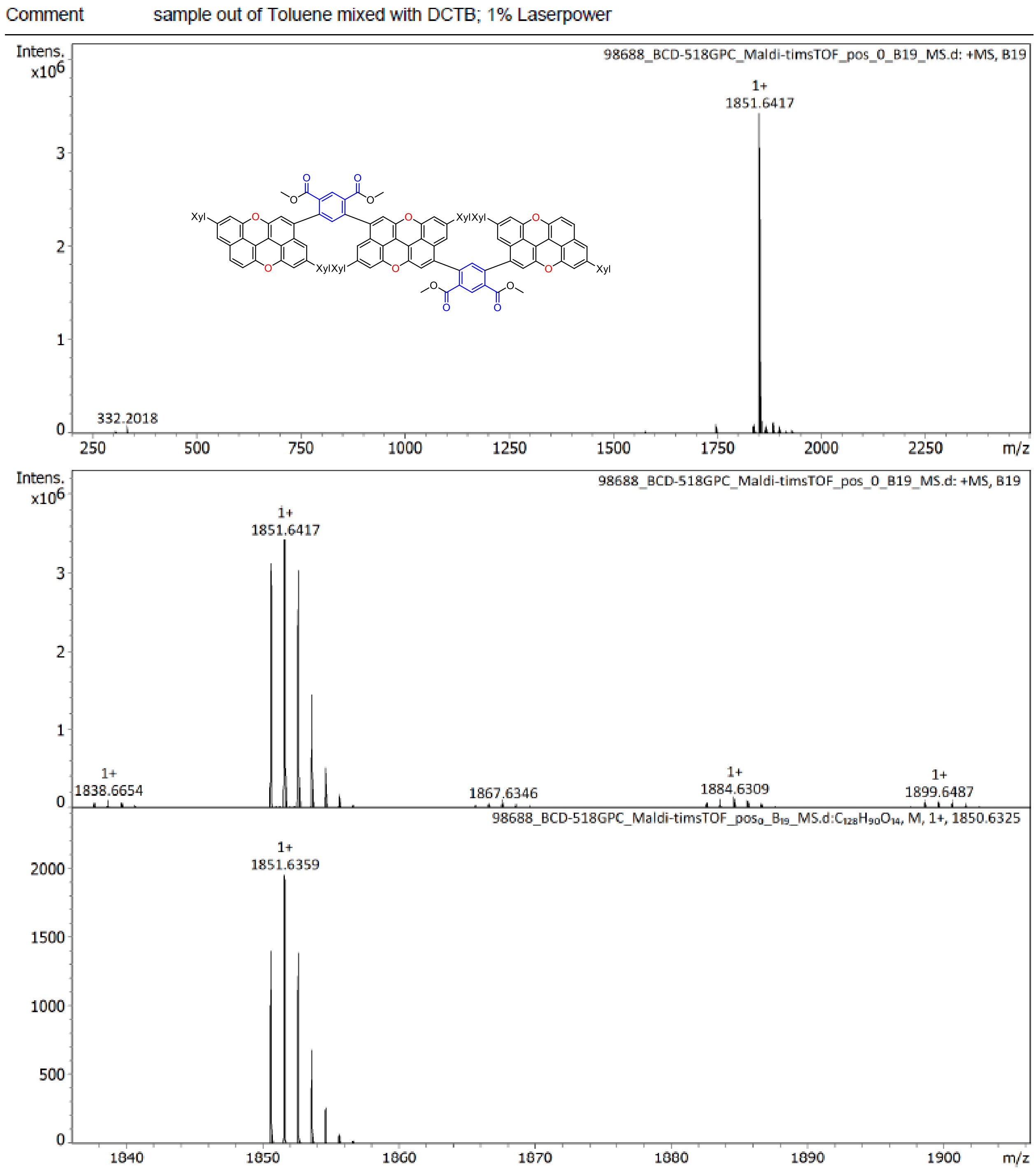

Figure S64. HRMS (MALDI-timsTOF) of **23** (from top: full, zoom and simulated spectra).

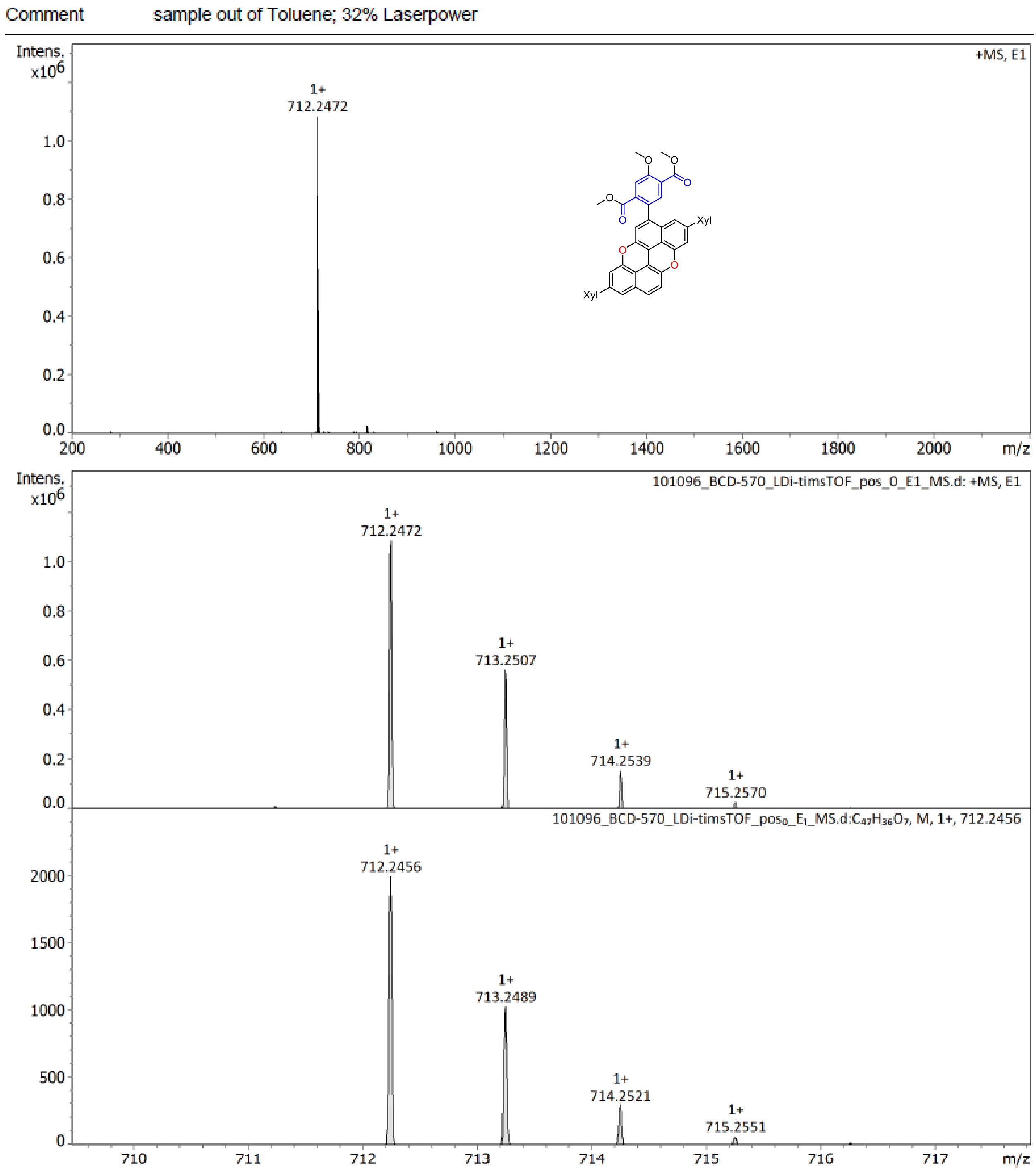

Figure S65. HRMS (LD-timsTOF) of **24** (from top: full, zoom and simulated spectra).

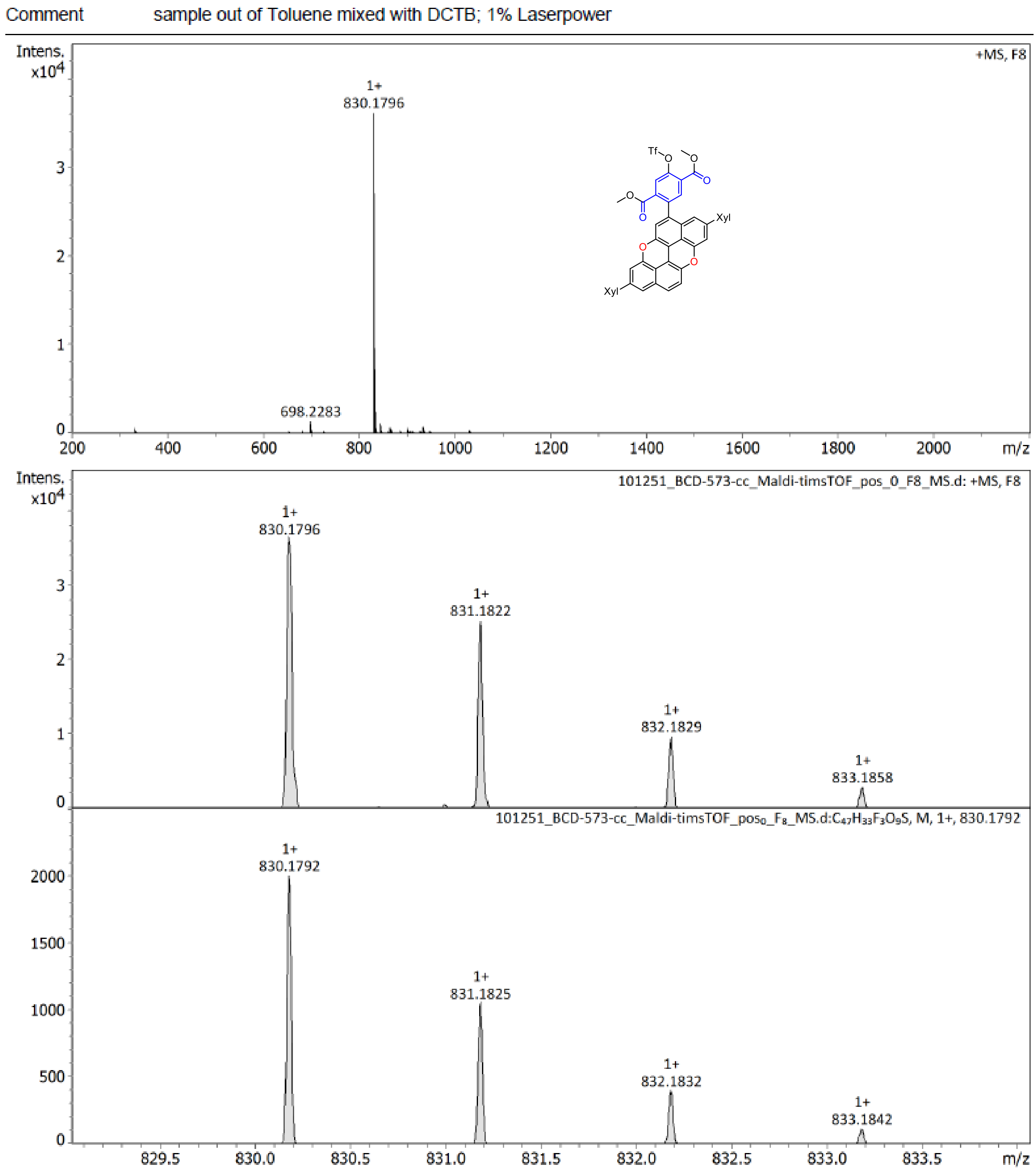

Figure S66. HRMS (MALDI-timsTOF) of **25** (from top: full, zoom and simulated spectra).

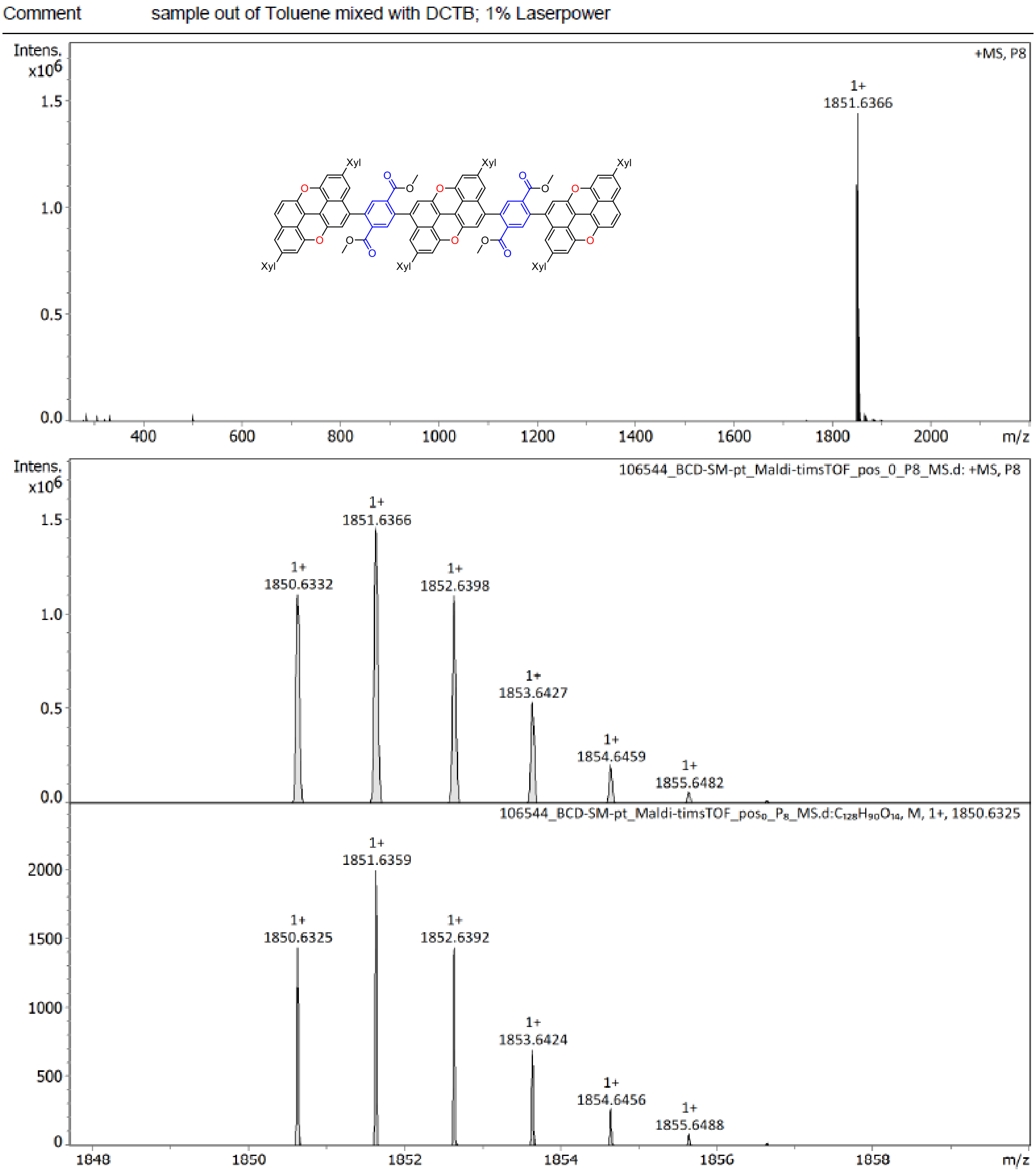

Figure S67. HRMS (MALDI-timsTOF) of **26** (from top: full, zoom and simulated spectra).

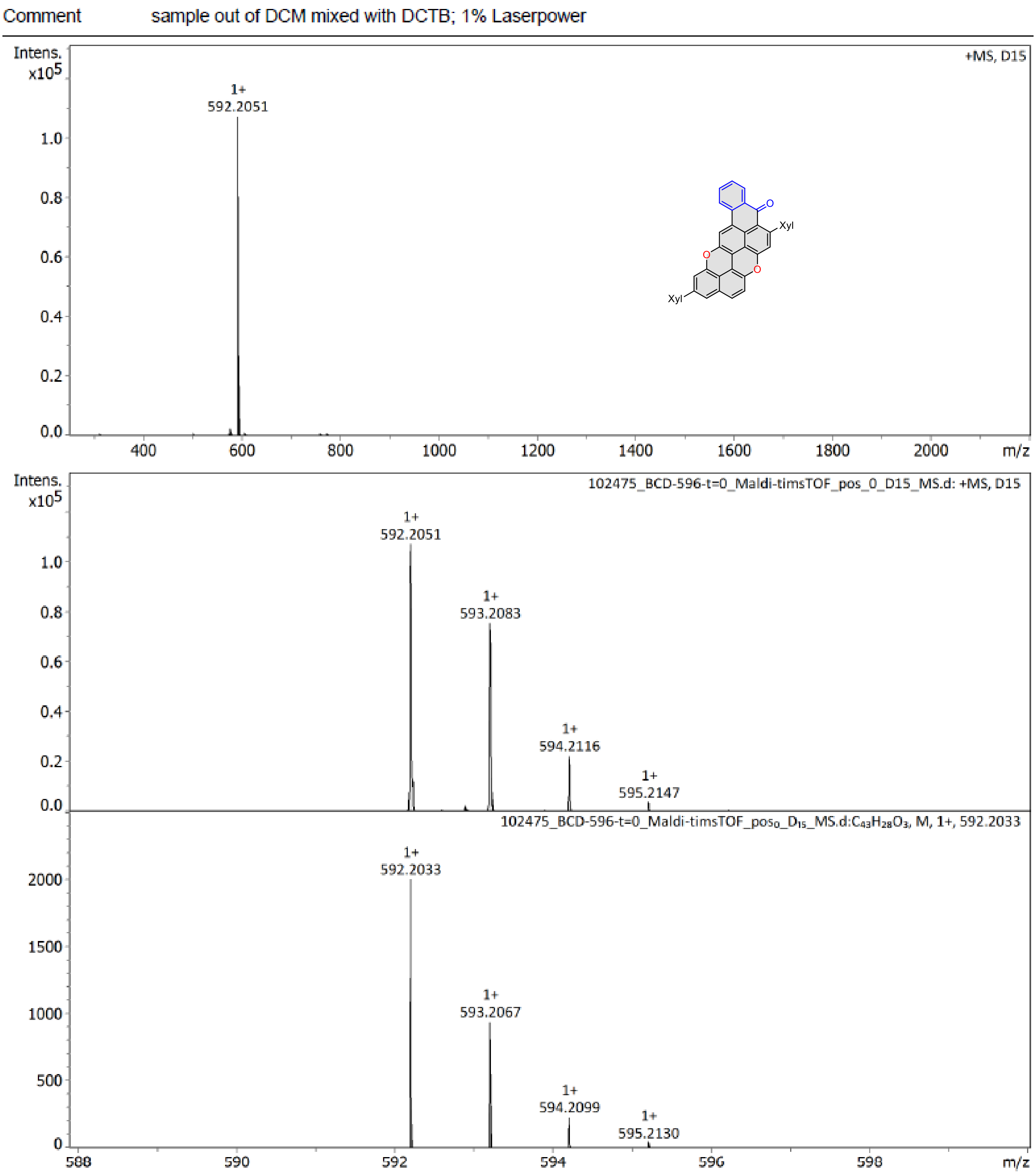

Figure S68. HRMS (MALDI-timsTOF) of **1** (from top: full, zoom and simulated spectra).

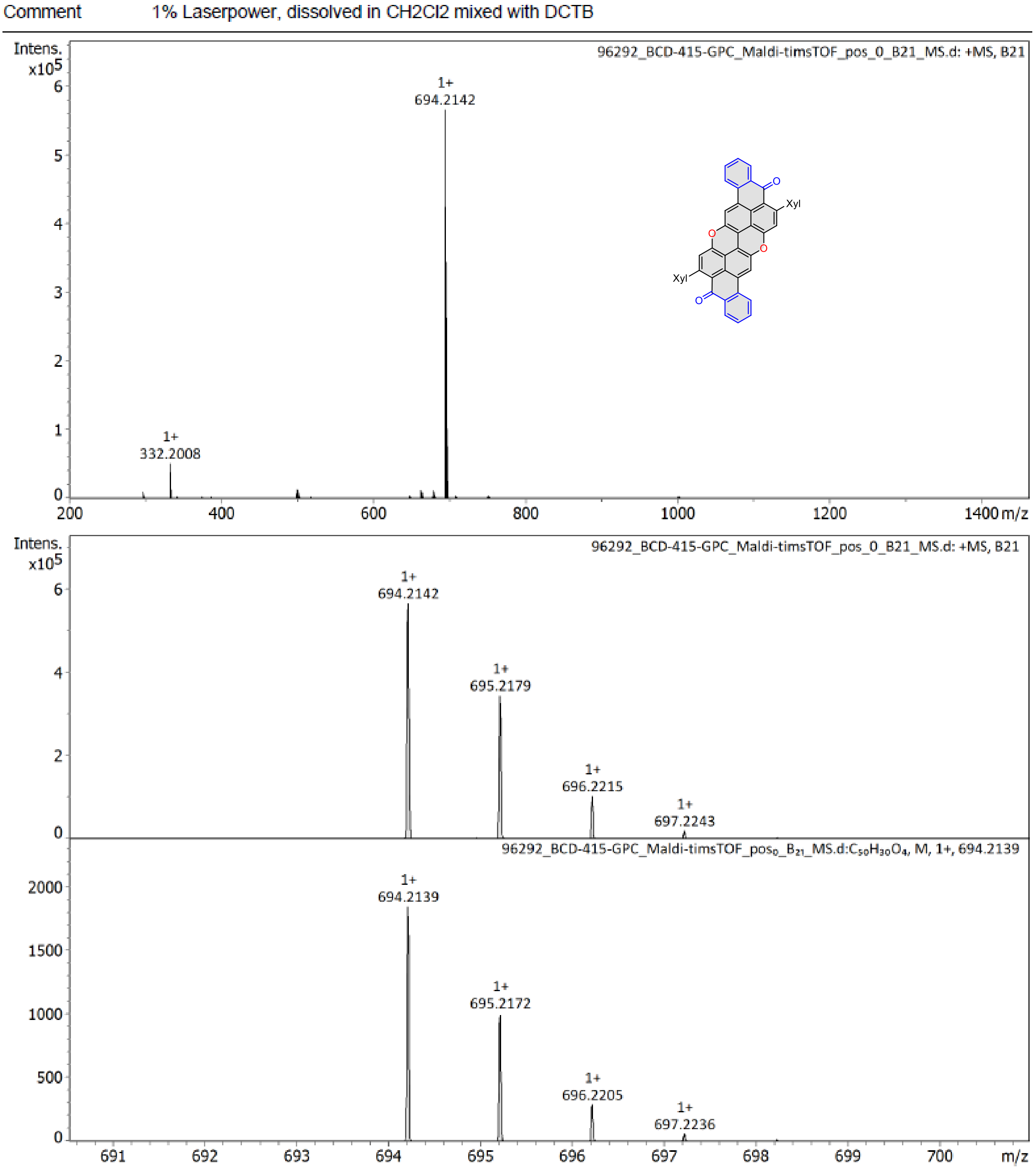

Figure S69. HRMS (MALDI-timsTOF) of **2** (from top: full, zoom and simulated spectra); 332.20 = matrix.

Comment sample out of Toluene mixed with DCTB; 1% Laserpower

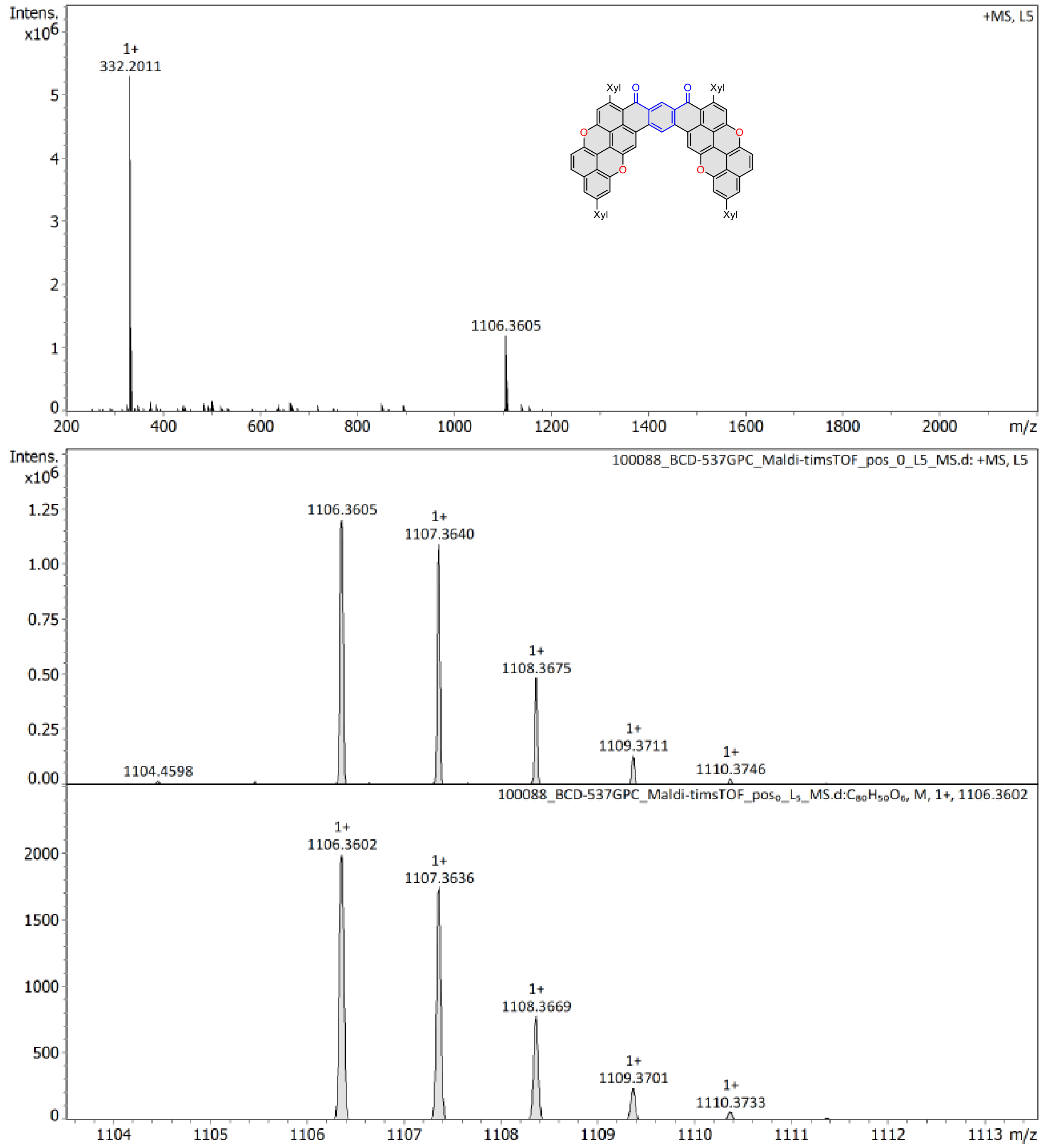

Figure S70. HRMS (MALDI-timsTOF) of **3** (from top: full, zoom and simulated spectra); 332.20 = matrix.

Comment sample out of DCM mixed with DCTB; 1% Laserpower

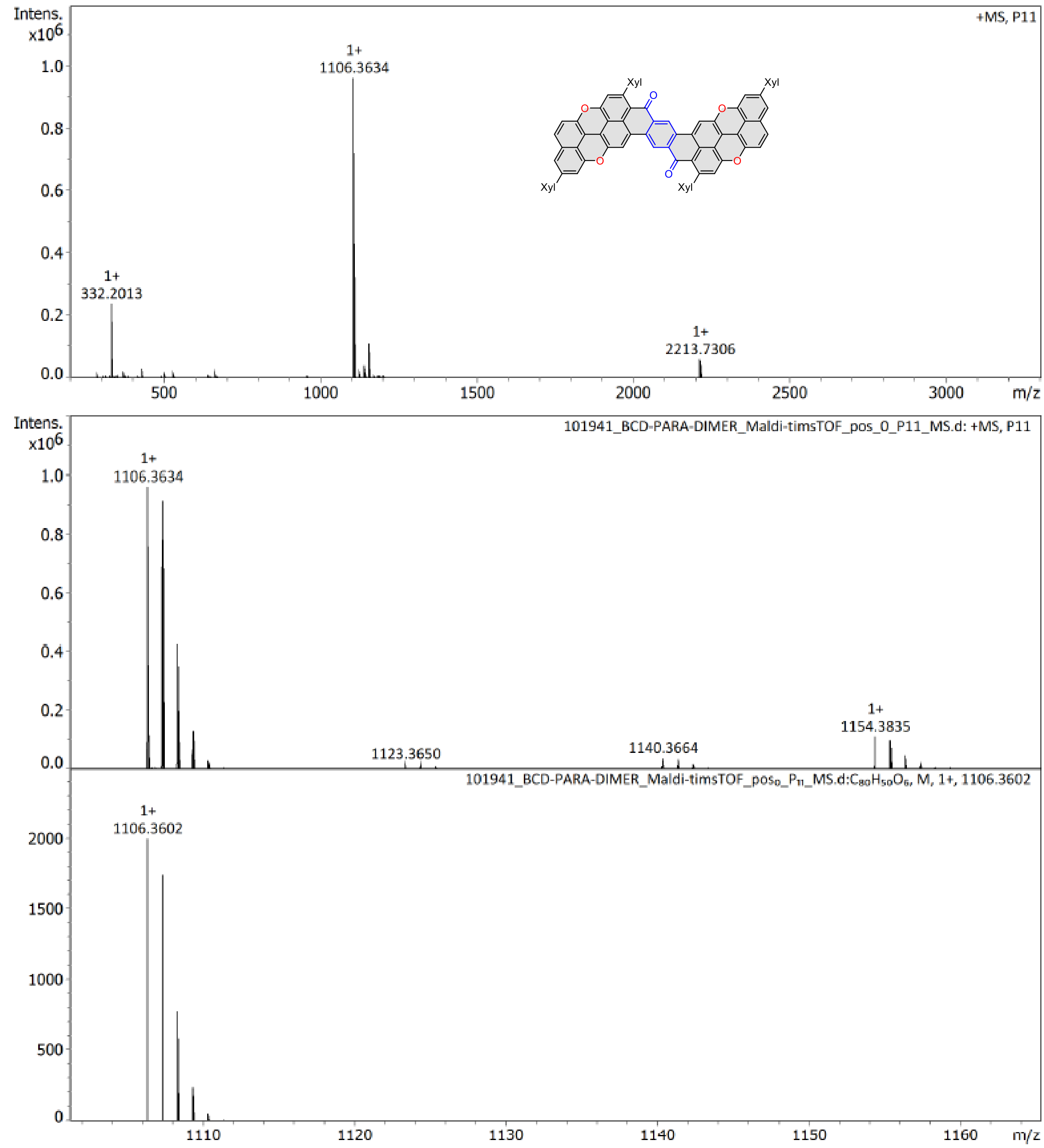

Figure S71. HRMS (MALDI-timsTOF) of **4** (from top: full, zoom and simulated spectra); 332.20 = matrix.

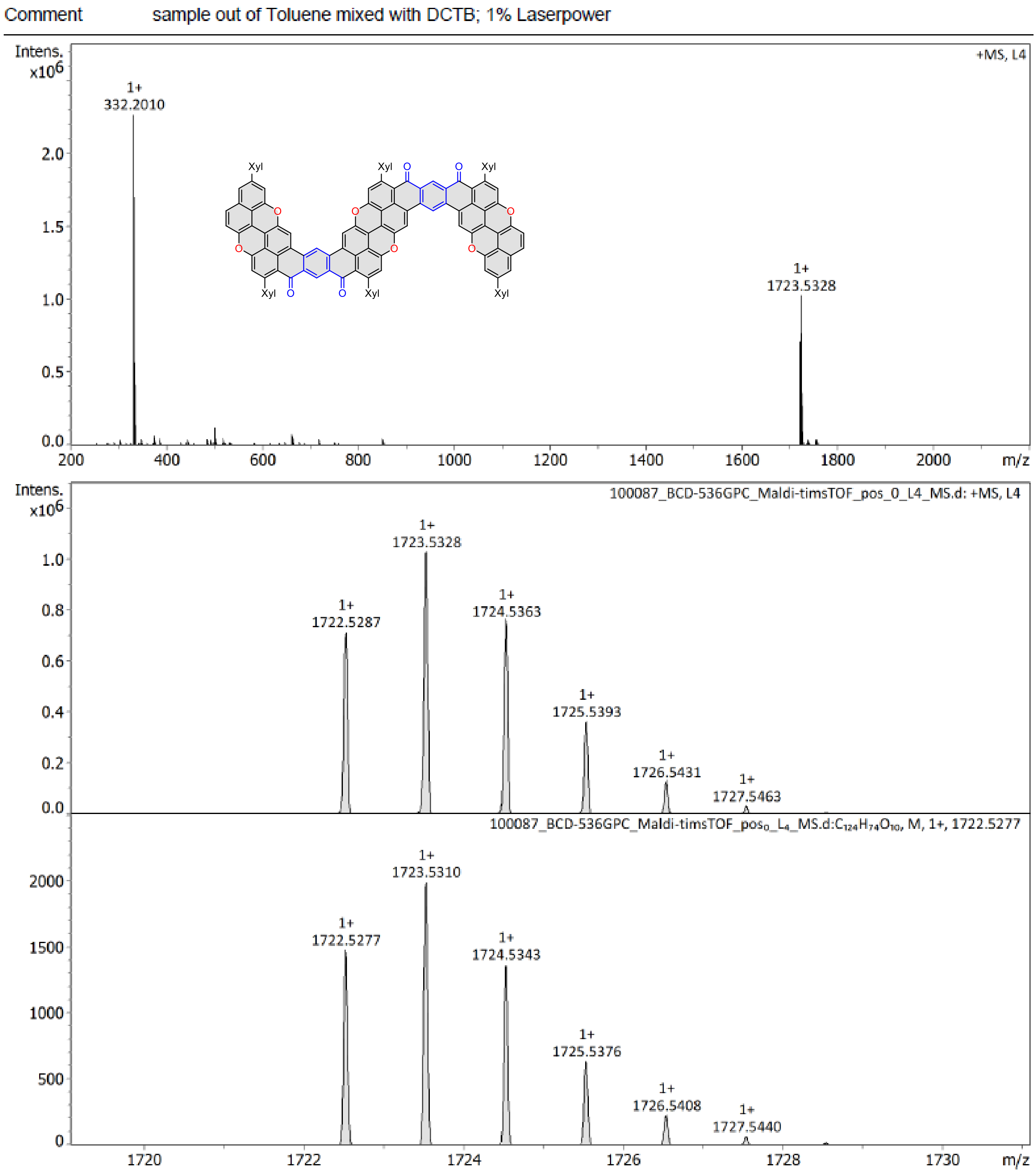

Figure S72. HRMS (MALDI-timsTOF) of **5** (from top: full, zoom and simulated spectra); 332.20 = matrix.

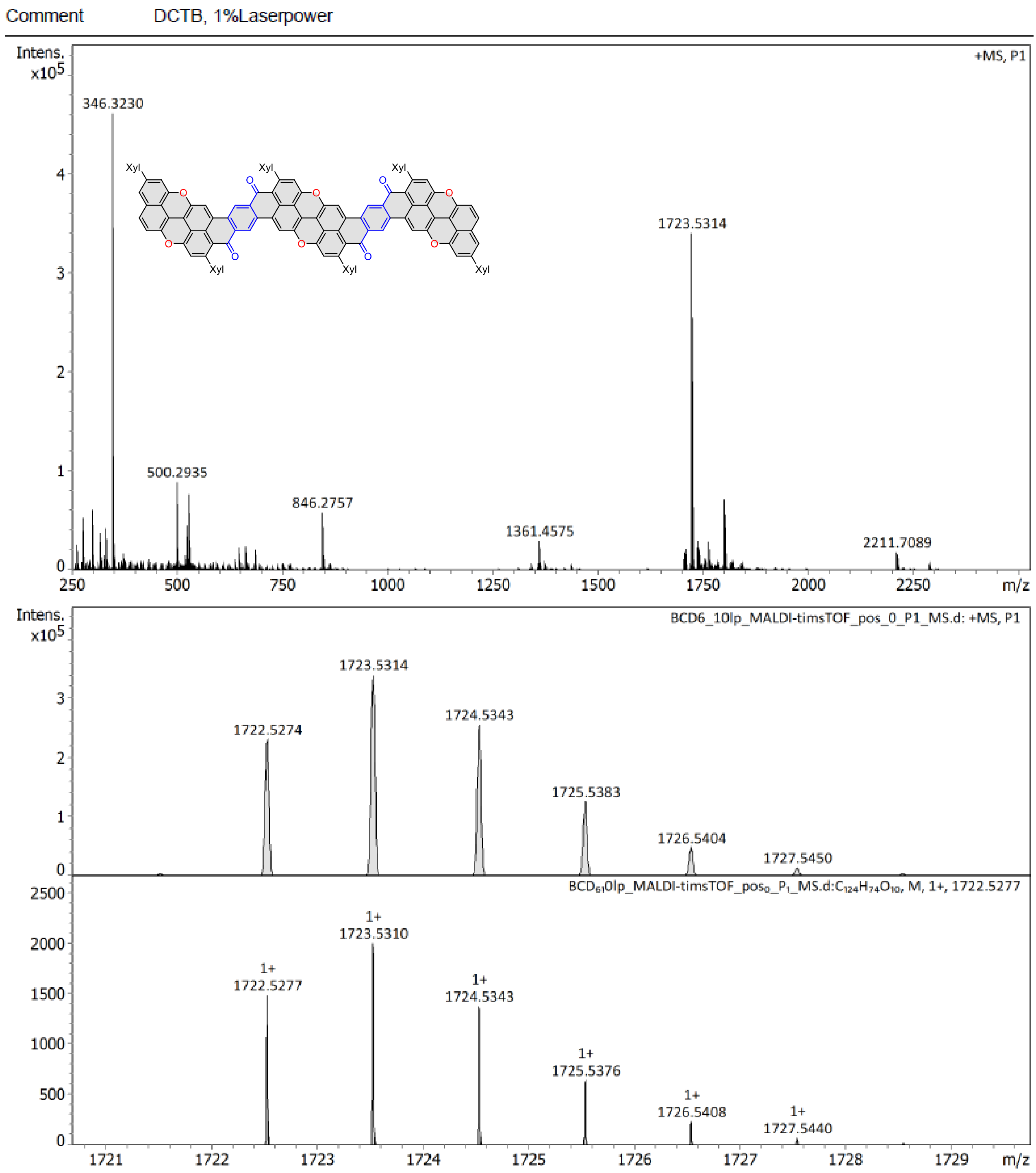

Figure S73. HRMS (MALDI-timsTOF) of **6** (from top: full, zoom and simulated spectra); 346.32 = matrix.

3. Optoelectronic characterization

3.1. Photophysical properties

Photophysical characterization of nanoribbon **1-6** and reference **8**

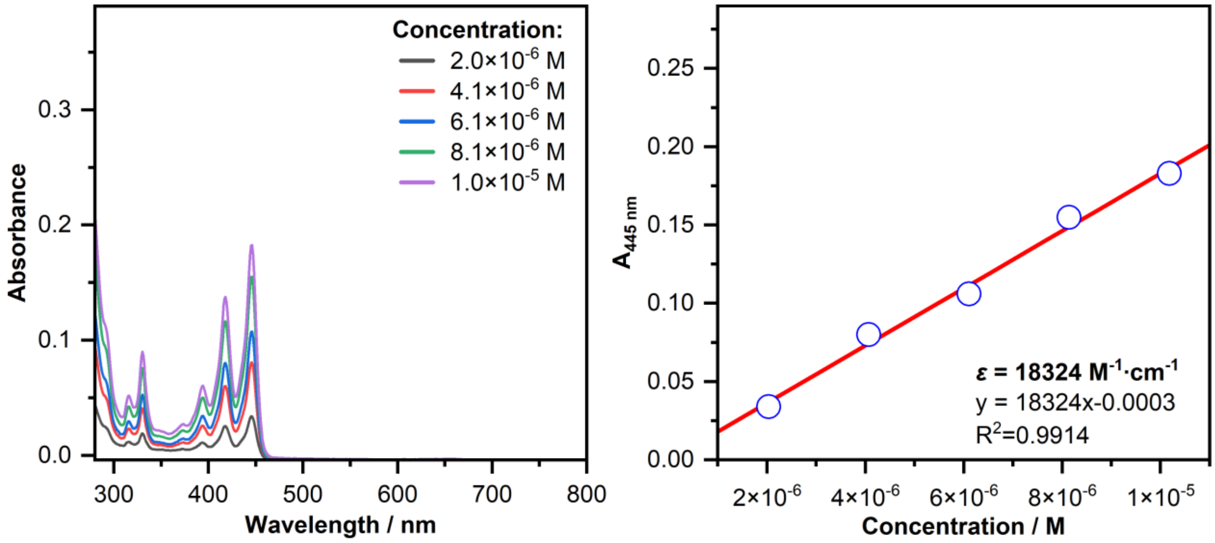

Figure S74. Determination of the molar absorption coefficient (ε) of **8** in CH_2Cl_2.

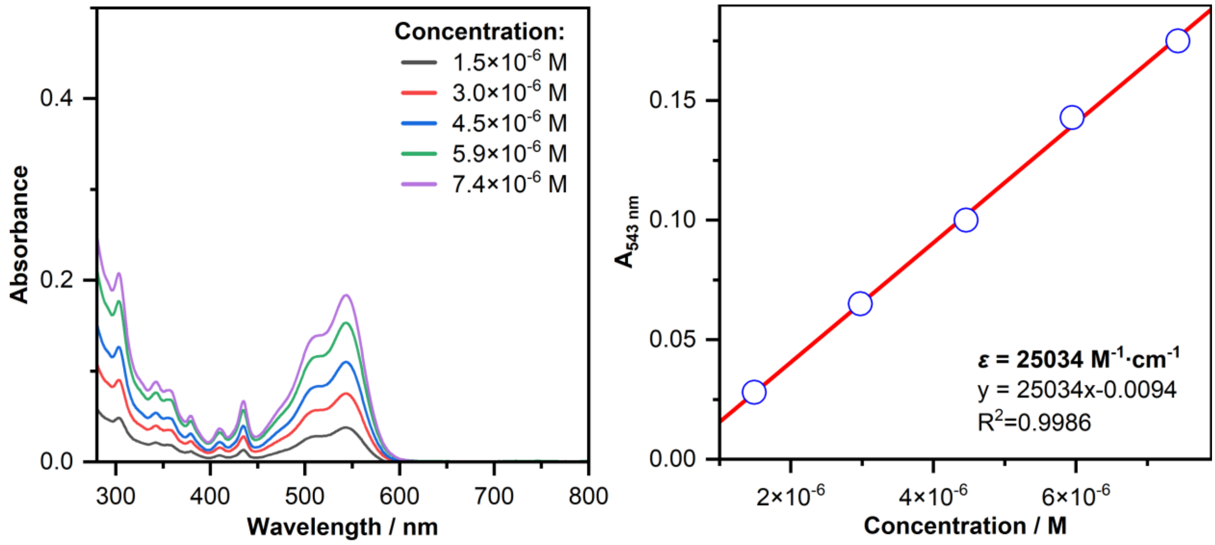

Figure S75. Determination of the molar absorption coefficient (ε) of **1** in CH_2Cl_2.

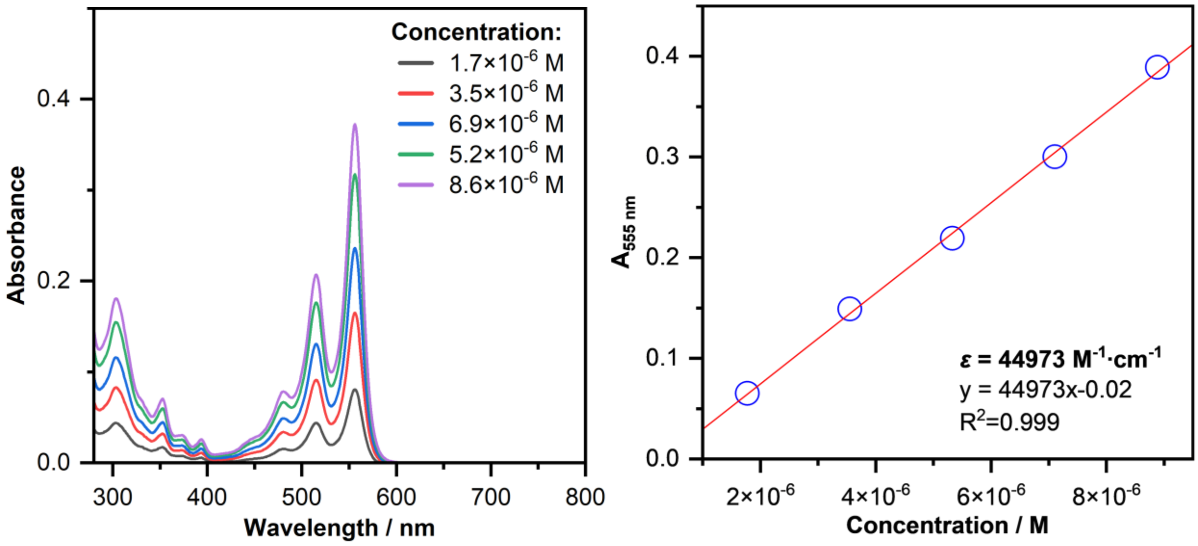

Figure S76. Determination of the molar absorption coefficient (ε) of **2** in CH_2Cl_2.

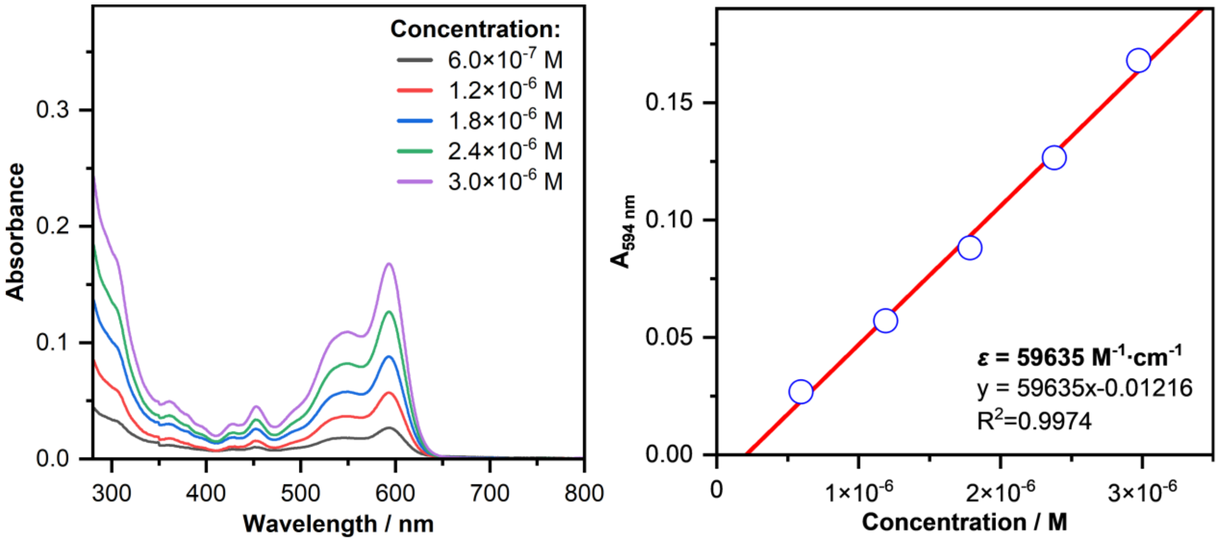

Figure S77. Determination of the molar absorption coefficient (ε) of **3** in CH_2Cl_2.

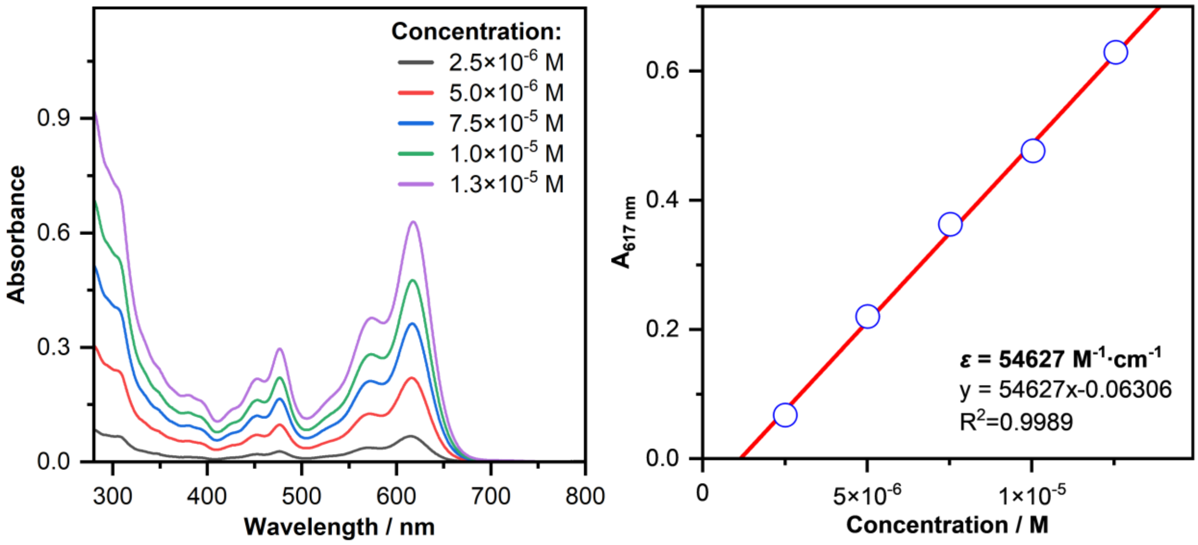

Figure S78. Determination of the molar absorption coefficient (ε) of **4** in CH_2Cl_2.

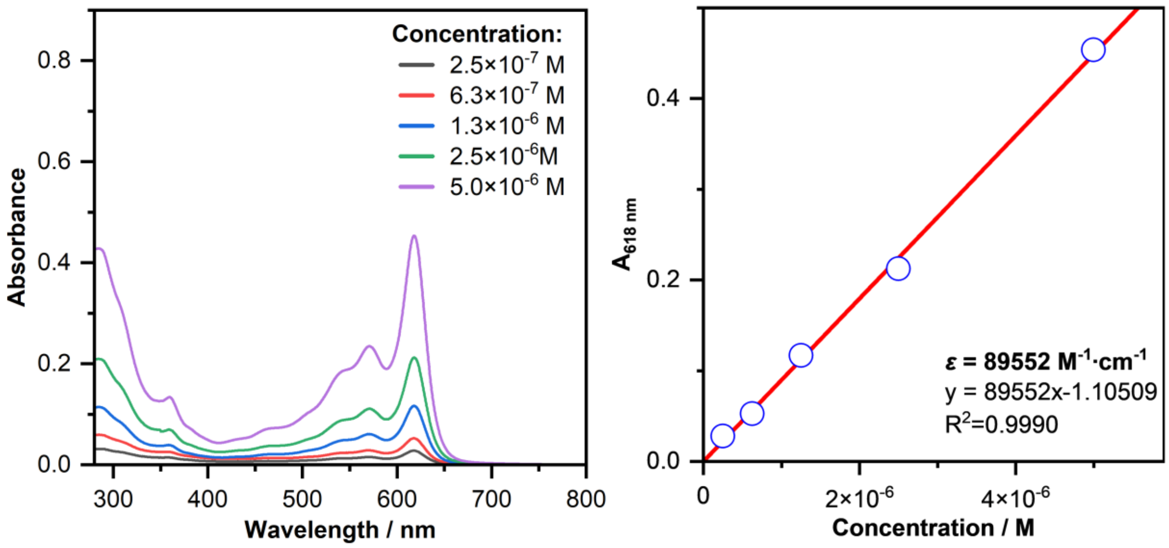

Figure S79. Determination of the molar absorption coefficient (ε) of **5** in CH_2Cl_2.

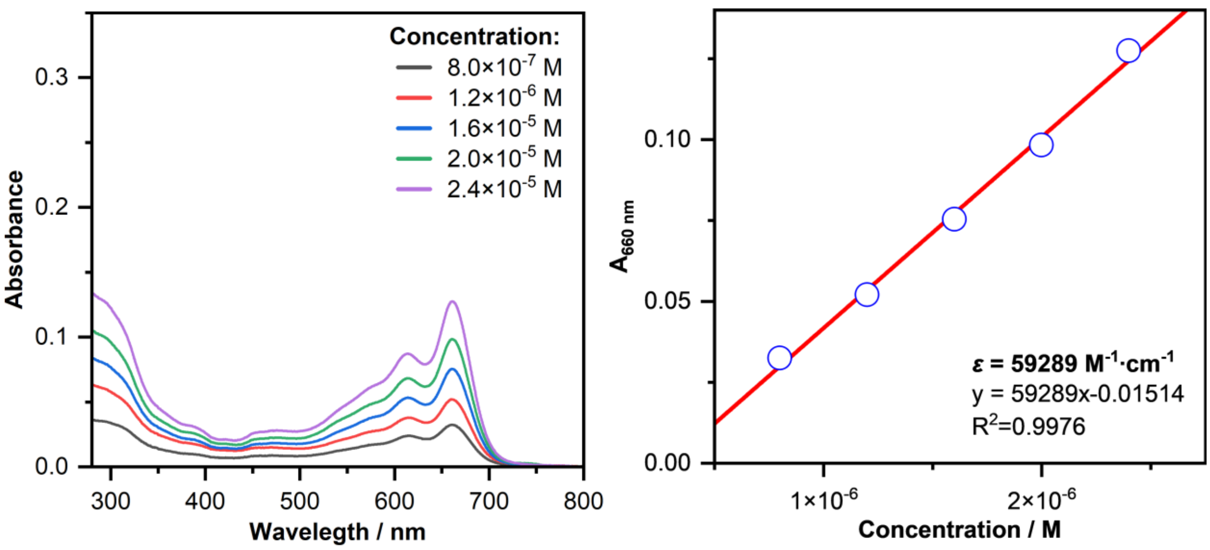

Figure S80. Determination of the molar absorption coefficient (ε) of **6** in CH_2Cl_2.

Fluorescence quantum yield of nanoribbon **1-6** and reference **8**.

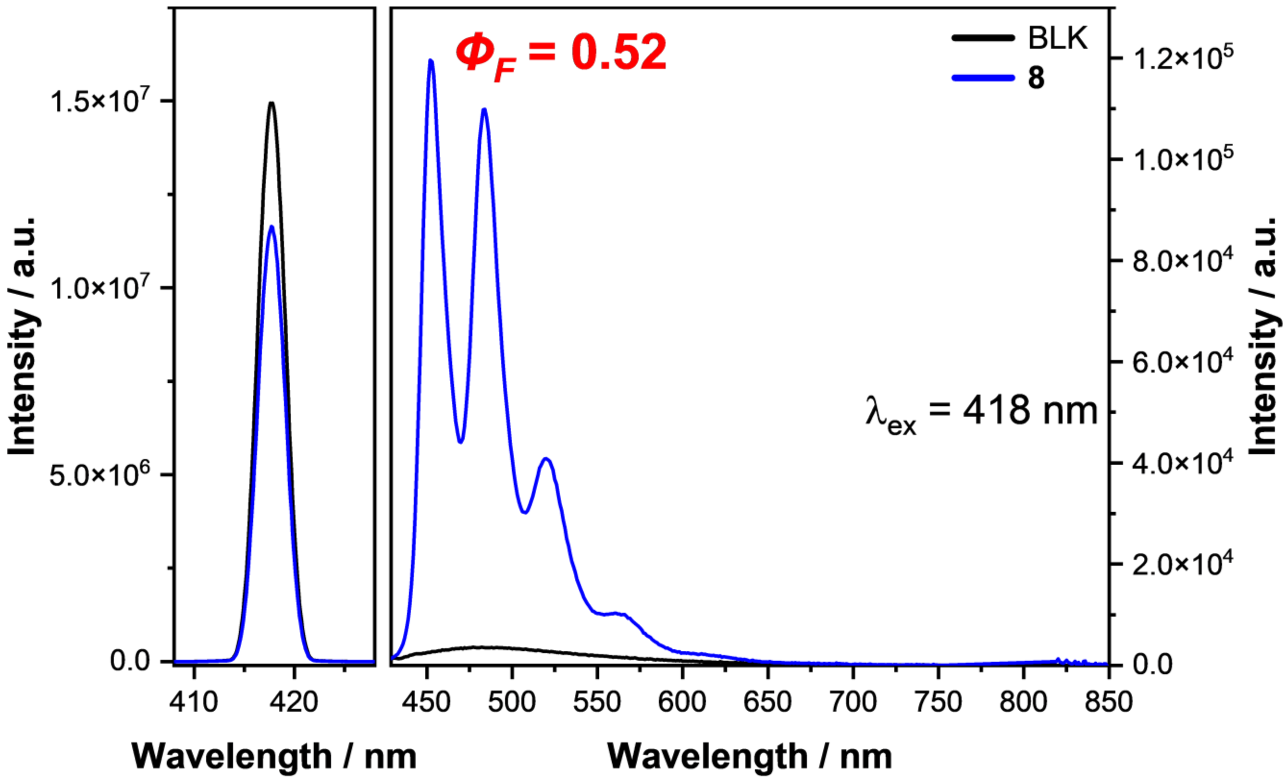

Figure S81. Excitation scatter region (left) and emission spectra (right) of 1.8 × 10^{-6} M **8** in CH_2Cl_2 used for the absolute quantum yield calculation.

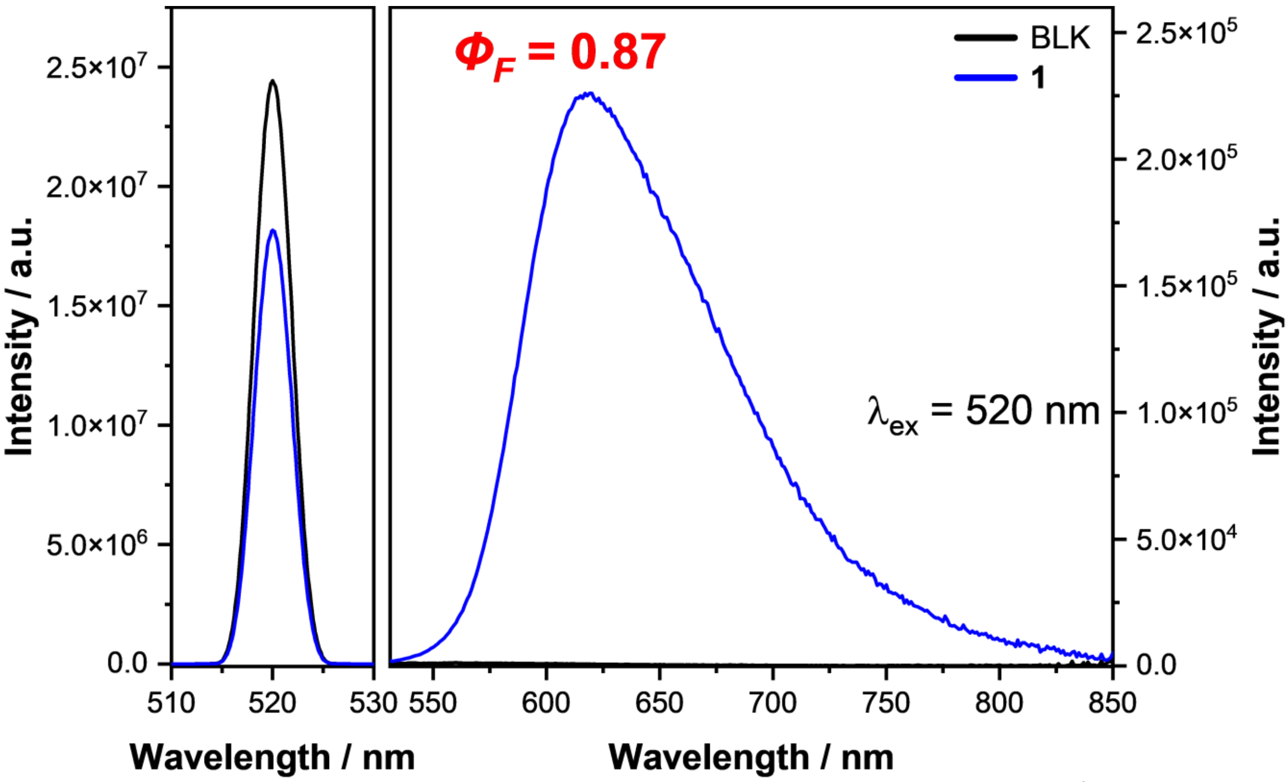

Figure S82. Excitation scatter region (left) and emission spectra (right) of 4.8 × 10^{-6} M **1** in CH_2Cl_2 used for the absolute quantum yield calculation.

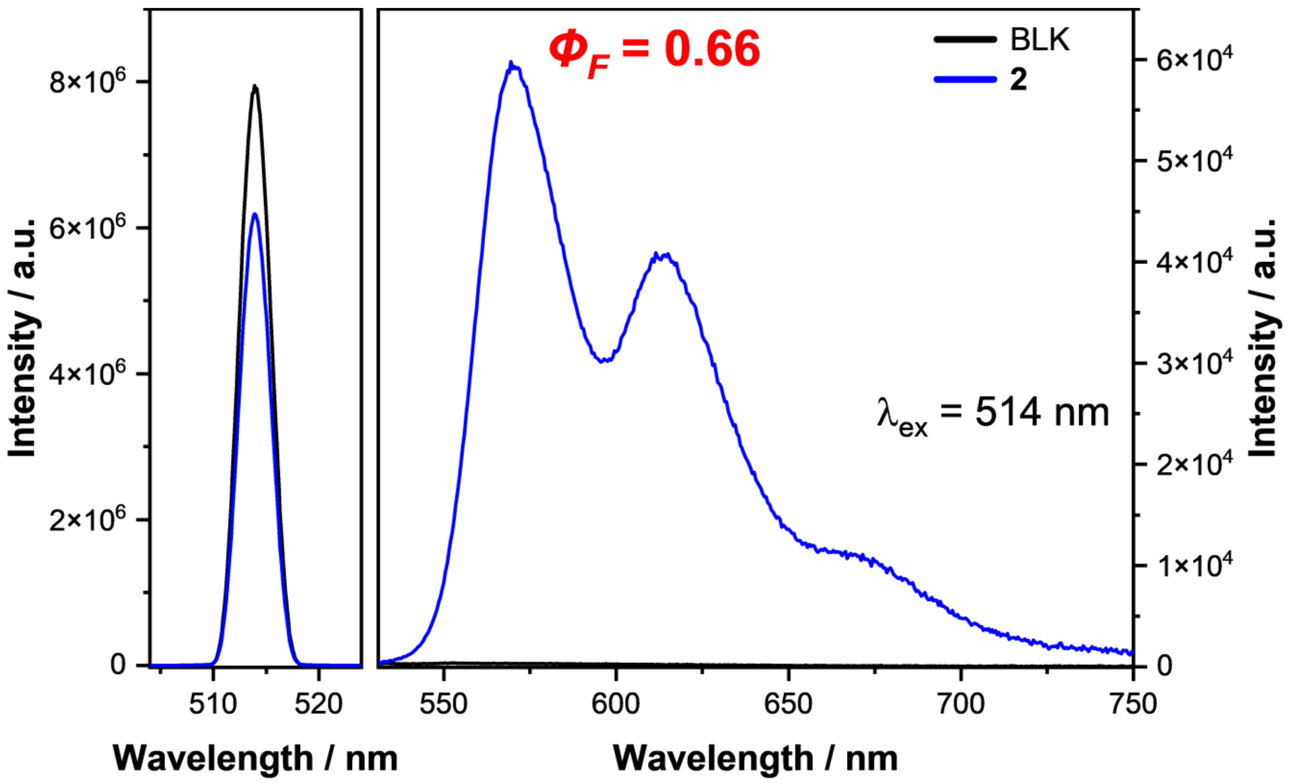

Figure S83. Excitation scatter region (left) and emission spectra (right) of 1.1 × 10^{-6} M **2** in CH_2Cl_2 used for the absolute quantum yield calculation.

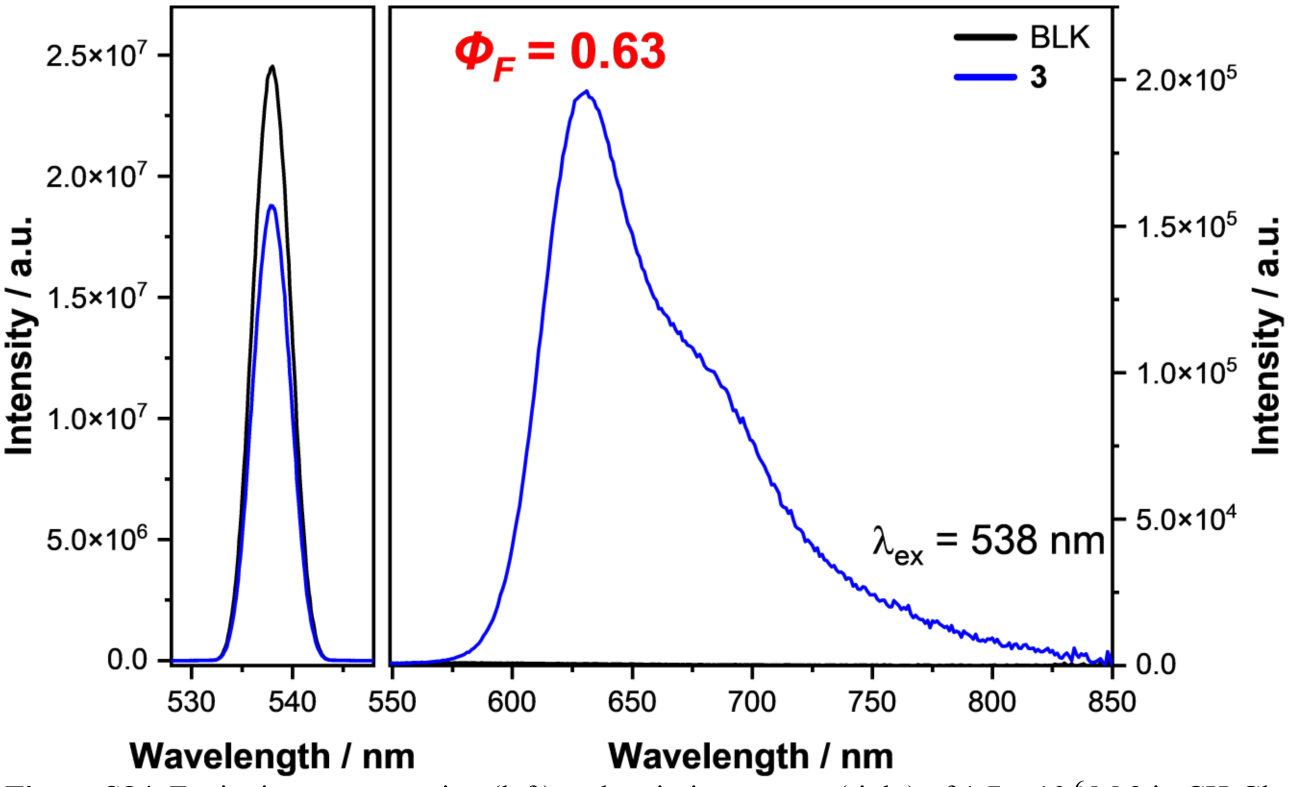

Figure S84. Excitation scatter region (left) and emission spectra (right) of 1.7 × 10^{-6} M **3** in CH_2Cl_2 used for the absolute quantum yield calculation.

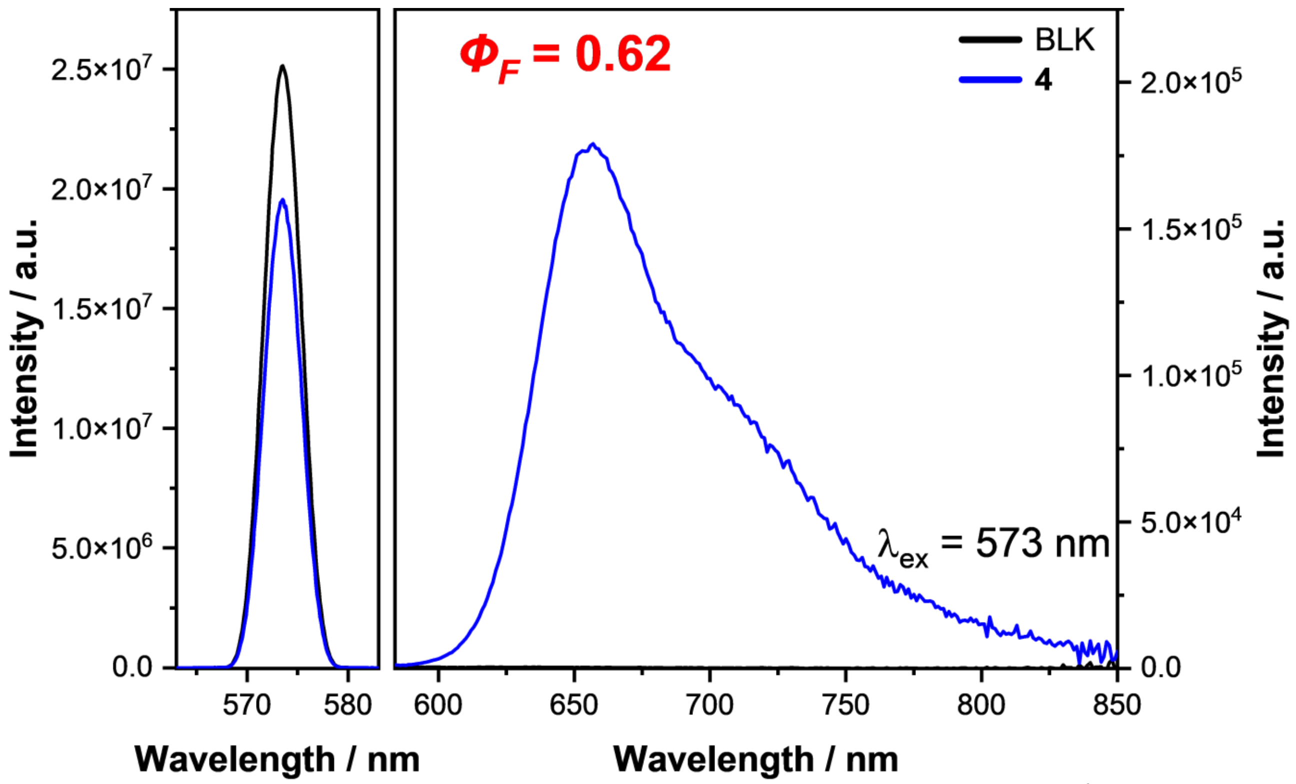

Figure S85. Excitation scatter region (left) and emission spectra (right) of 1.8×10^{-6} M **4** in CH_2Cl_2 used for the absolute quantum yield calculation.

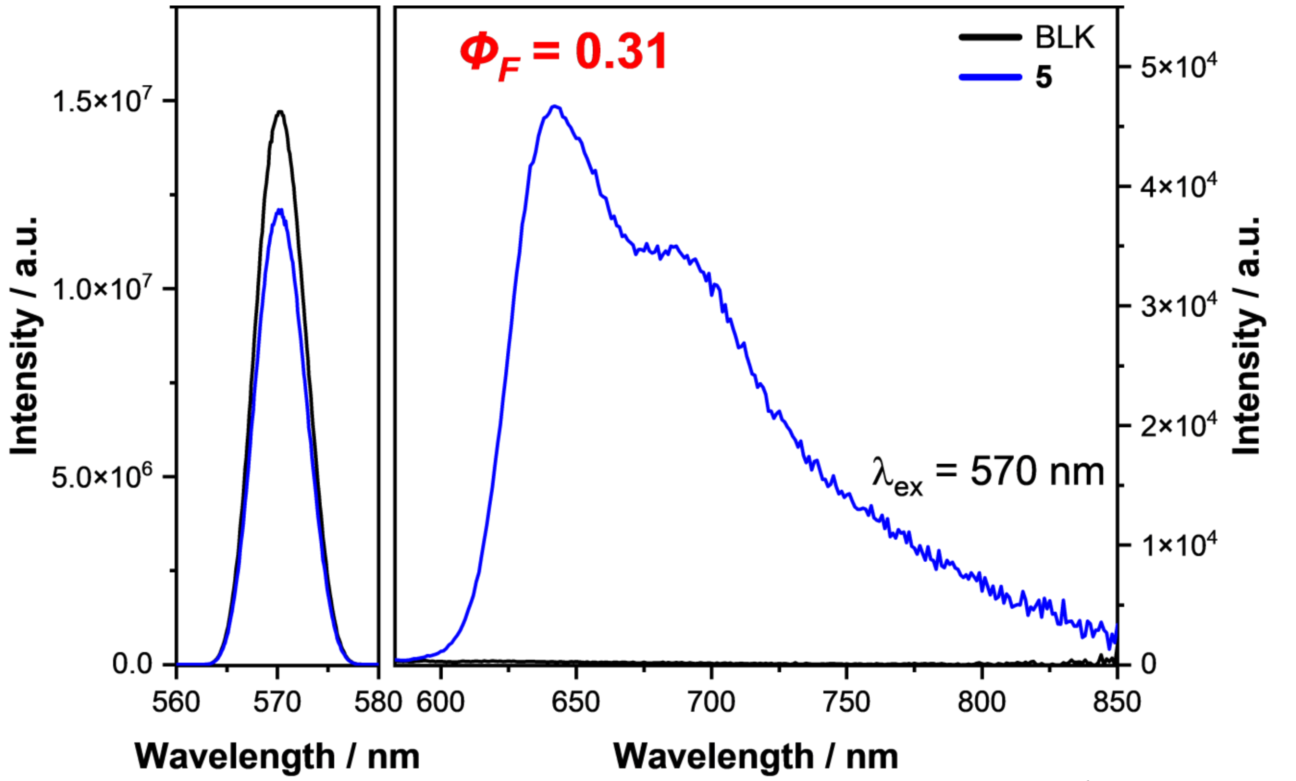

Figure S86. Excitation scatter region (left) and emission spectra (right) of 1.1×10^{-6} M **5** in CH_2Cl_2 used for the absolute quantum yield calculation.

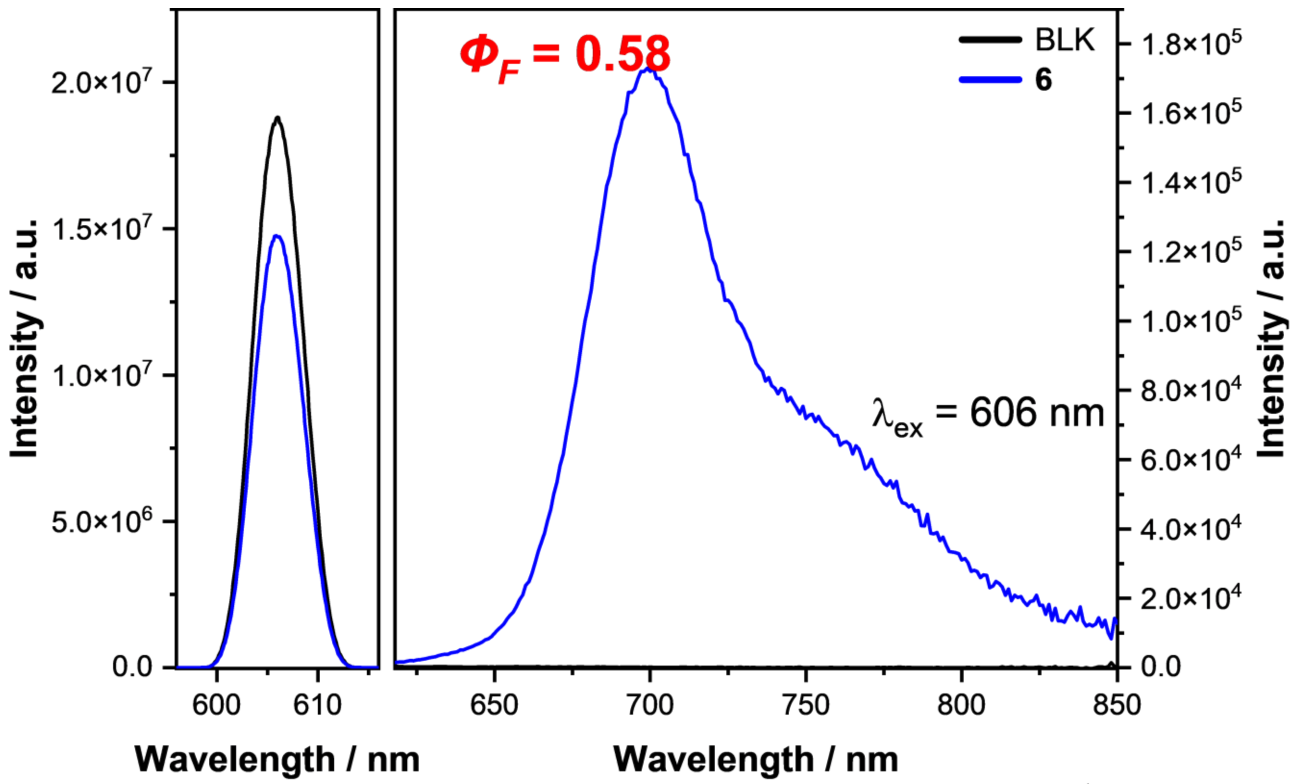

Figure S87. Excitation scatter region (left) and emission spectra (right) of 1.7 × 10^{-6} M **6** in CH_2Cl_2 used for the absolute quantum yield calculation.

Fluorescence lifetime of nanoribbon **1-6** and reference **8**

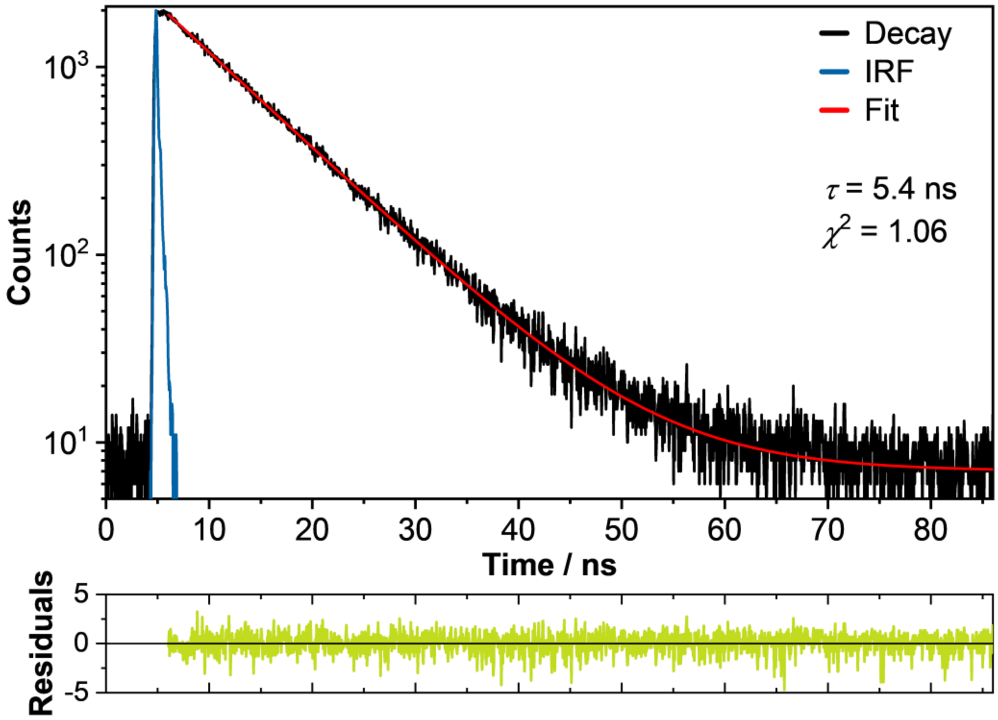

Figure S88. Time-resolved emission decay (λ_{ex} = 374.2 nm, λ_{em} = 483 nm) of 1.8 × 10^{-6} M **8** at RT in CH_2Cl_2.

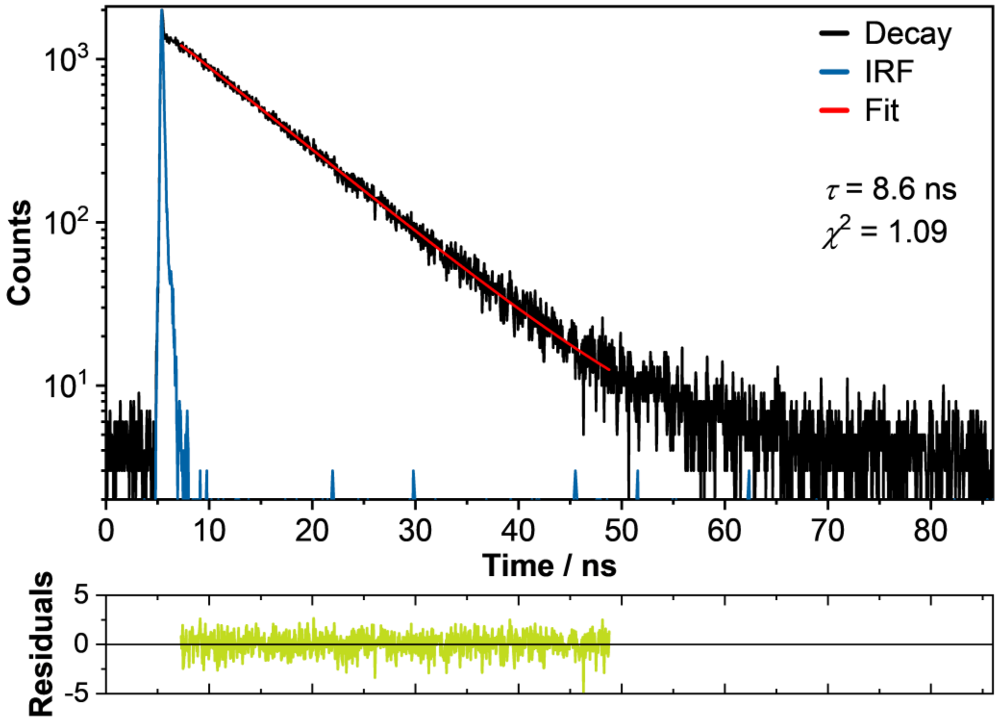

Figure S89. Time-resolved emission decay (λ_{ex} = 505 nm, λ_{em} = 631 nm) of 4.8 × 10^{-6} M **1** at RT in CH_2Cl_2.

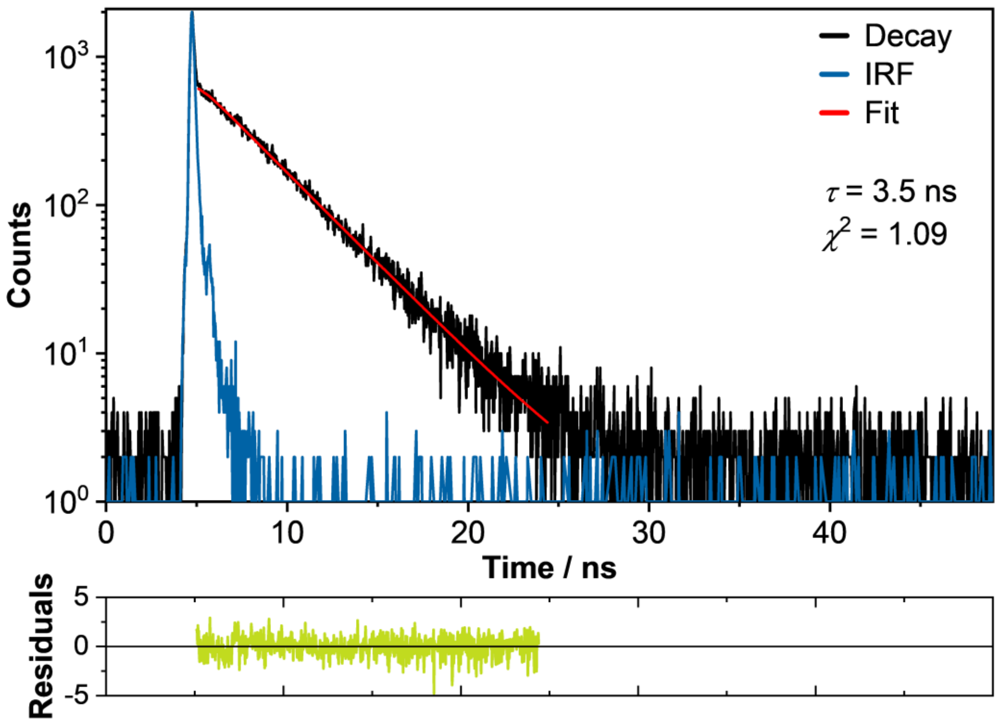

Figure S90. Time-resolved emission decay (λ_{ex} = 505 nm, λ_{em} = 612 nm) of 1.1 × 10^{-6} M **2** at RT in CH_2Cl_2.

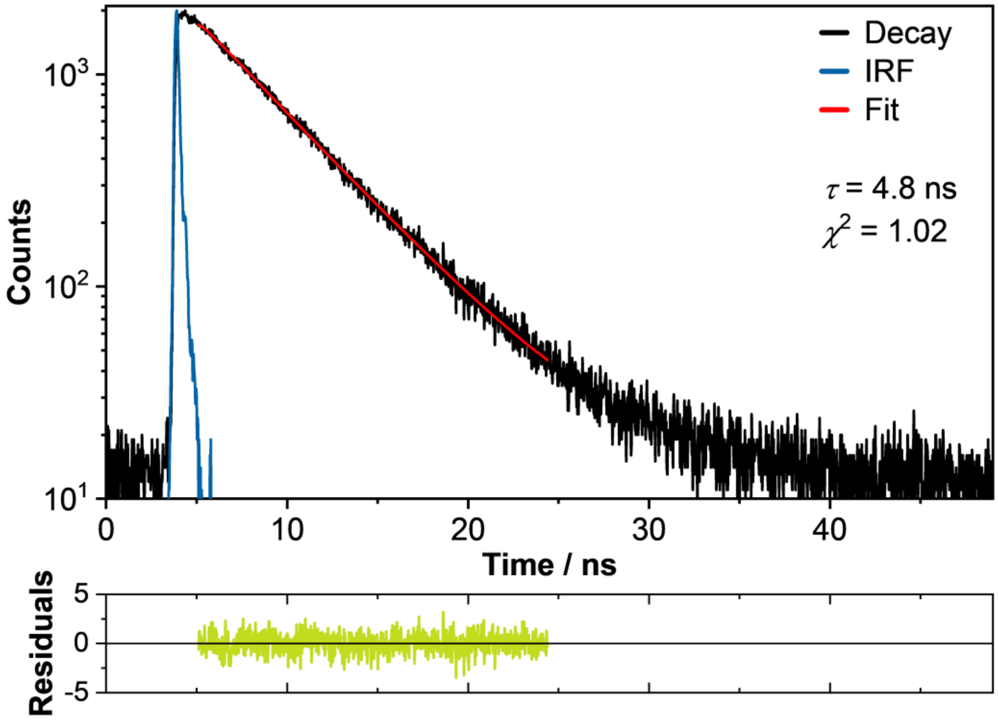

Figure S91. Time-resolved emission decay (λ_{ex} = 374.2 nm, λ_{em} = 628 nm) of 1.7 × 10^{-6} M **3** at RT in CH_2Cl_2.

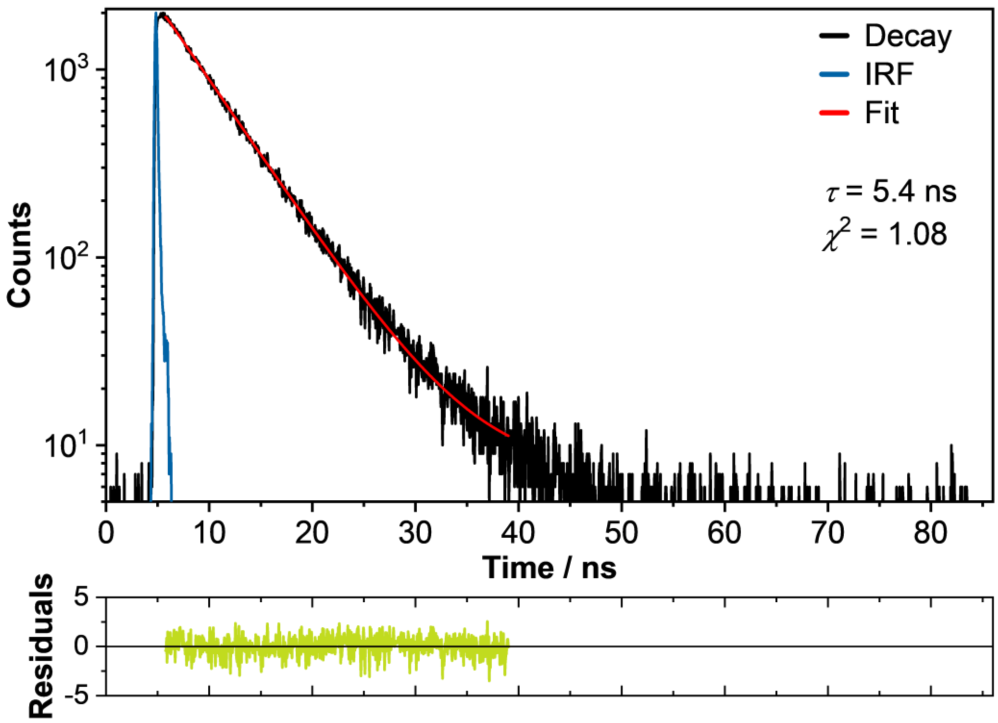

Figure S92. Time-resolved emission decay (λ_{ex} = 374.2 nm, λ_{em} = 714 nm) of 1.8 × 10^{-6} M **4** at RT in CH_2Cl_2.

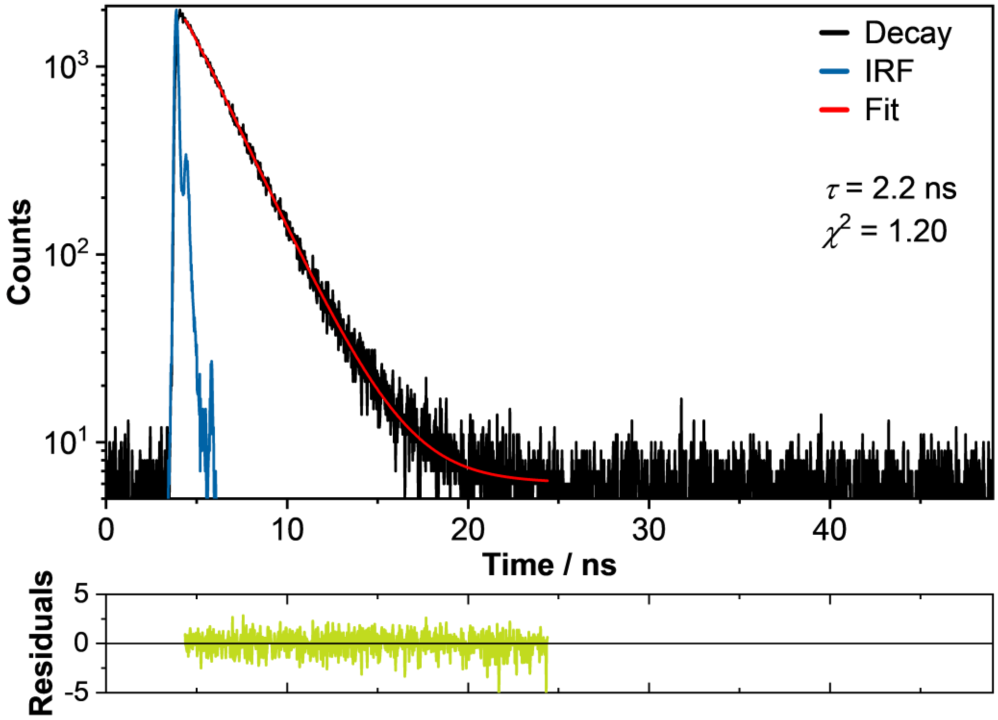

Figure S93. Time-resolved emission decay (λ_{ex} = 374.2 nm, λ_{em} = 636 nm) of 1.1 × 10^{-6} M **5** at RT in CH_2Cl_2.

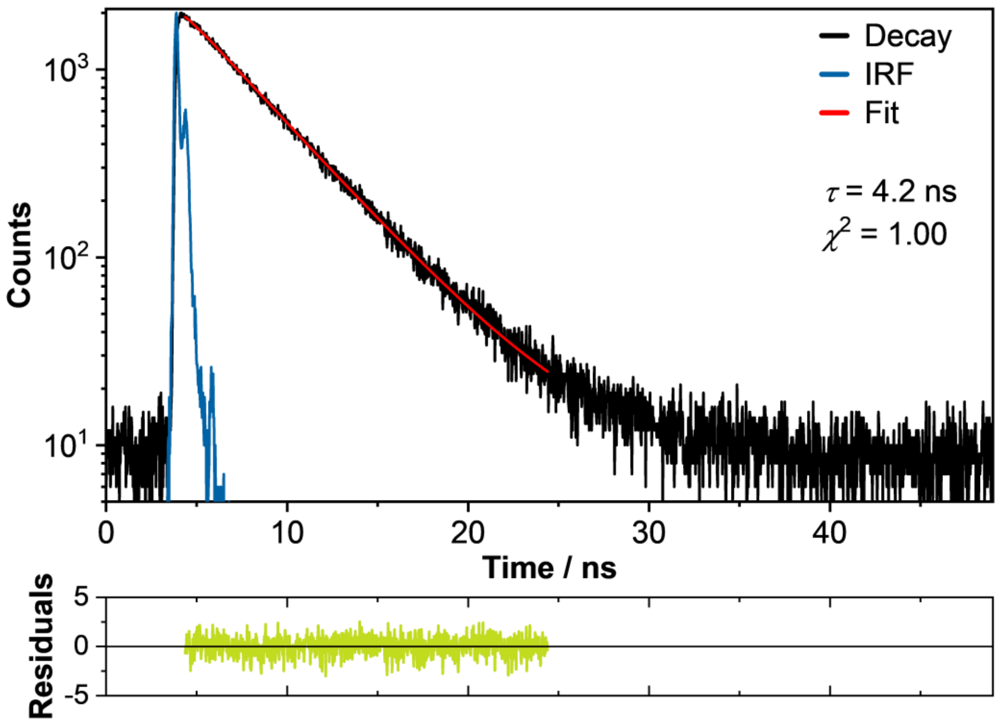

Figure S94. Time-resolved emission decay (λ_{ex} = 374.2 nm, λ_{em} = 696 nm) of 1.7 × 10^{-6} M **6** at RT in CH_2Cl_2.

Singlet oxygen sensitization quantum yield of nanoribbon **1-6** and reference **8**

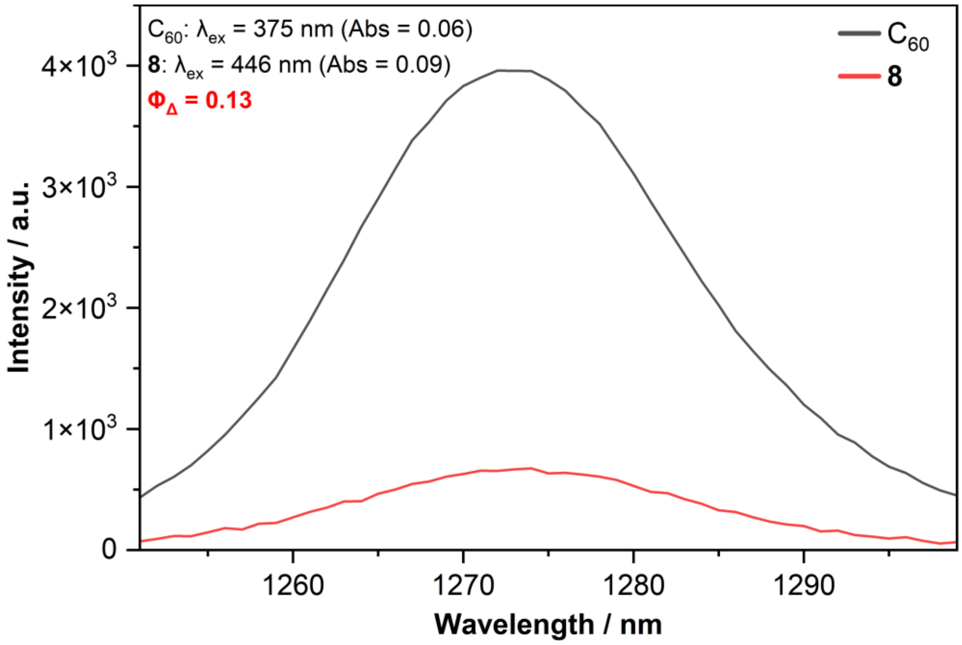

Figure S95. Emission of singlet oxygen sensitized by **8** and the reference C_{60} at RT in $CHCl_3$.

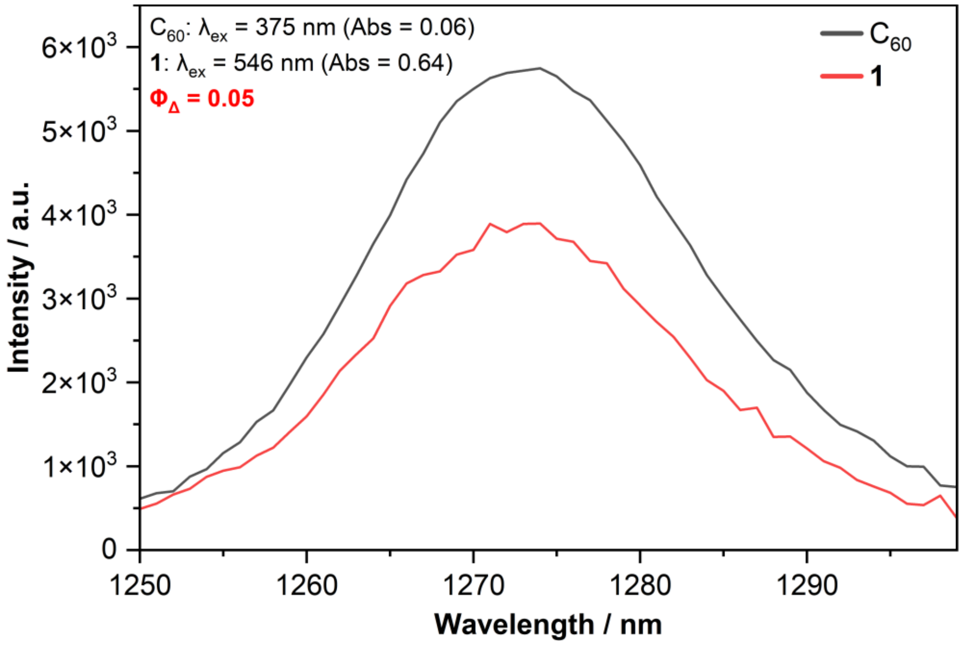

Figure S96. Emission of singlet oxygen sensitized by **1** and the reference C_{60} at RT in $CHCl_3$.

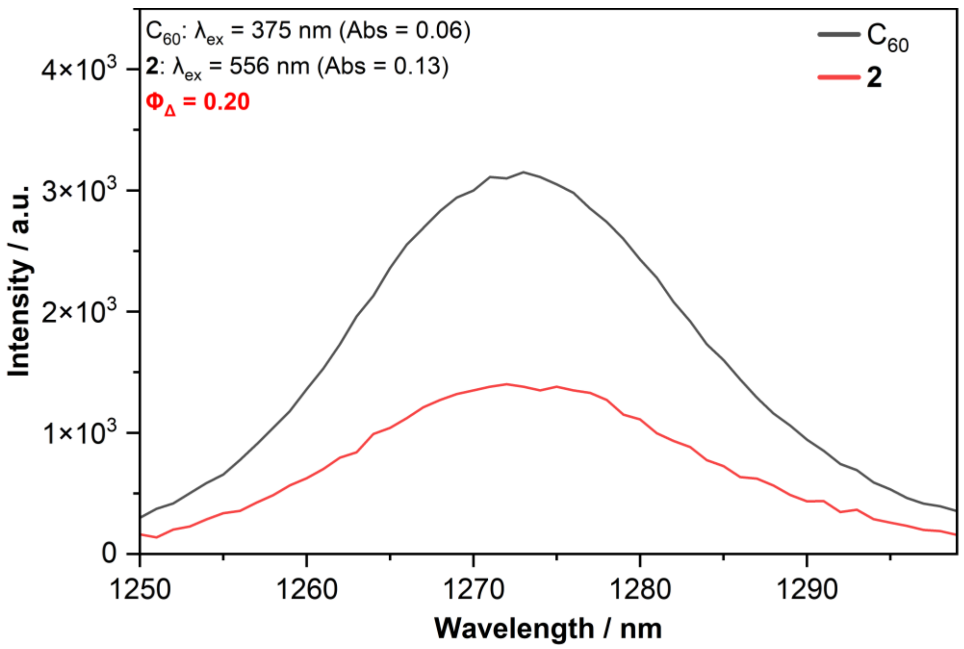

Figure S97. Emission of singlet oxygen sensitized by **2** and the reference C_{60} at RT in $CHCl_3$.

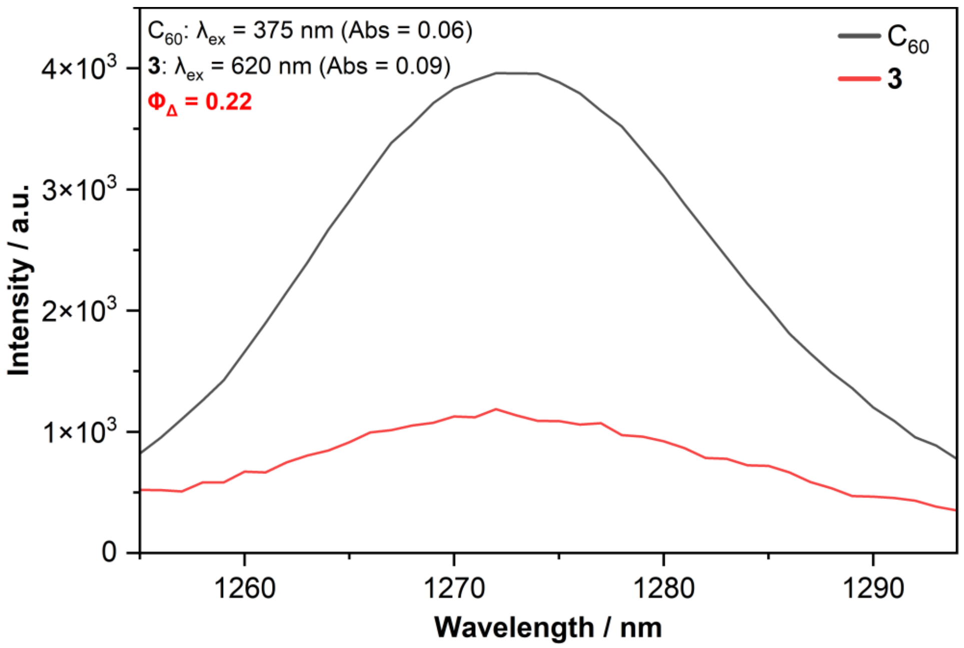

Figure S98. Emission of singlet oxygen sensitized by **3** and the reference C_{60} at RT in $CHCl_3$.

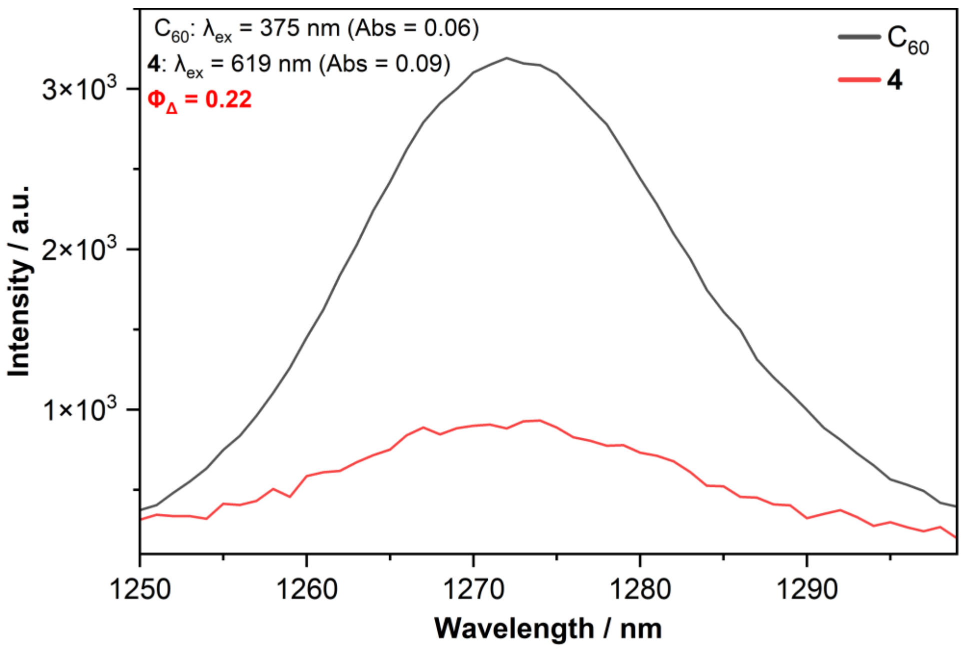

Figure S99. Emission of singlet oxygen sensitized by **4** and the reference C_{60} at RT in $CHCl_3$.

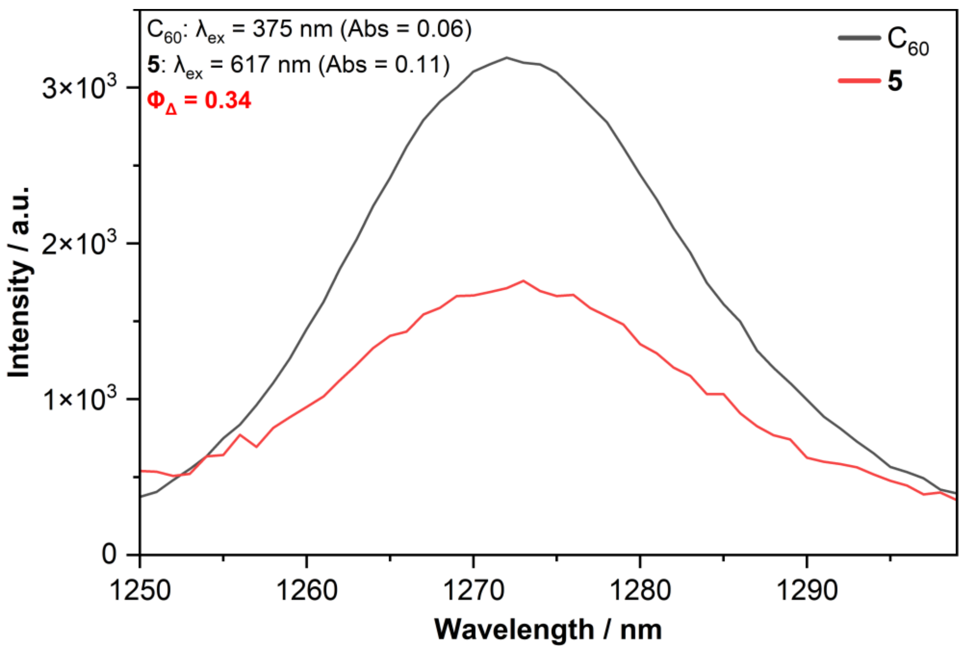

Figure S100. Emission of singlet oxygen sensitized by **5** and the reference C_{60} at RT in $CHCl_3$.

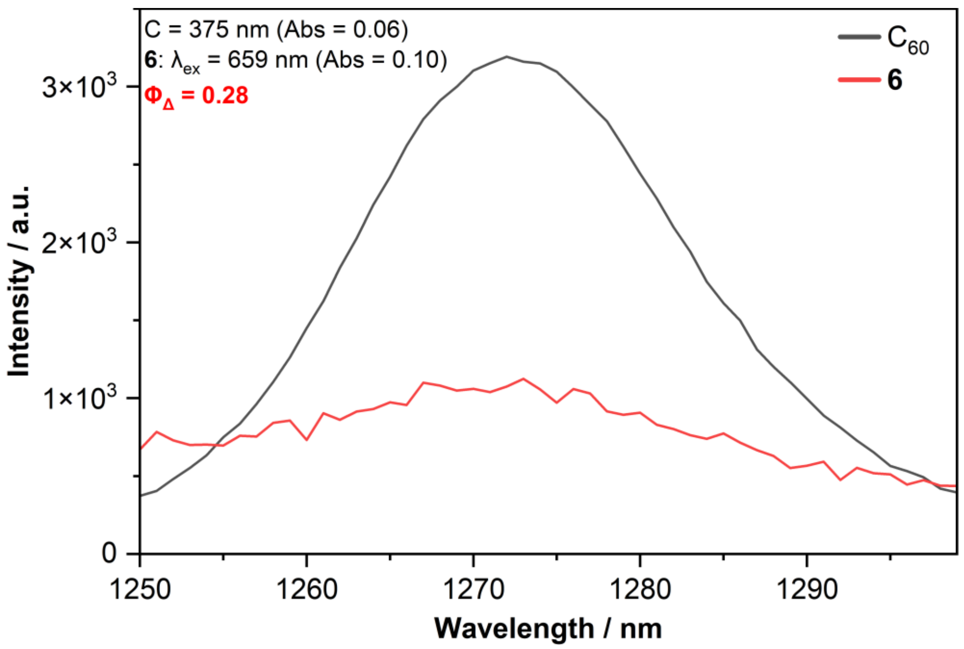

Figure S101. Emission of singlet oxygen sensitized by **6** and the reference C_{60} at RT in $CHCl_3$.

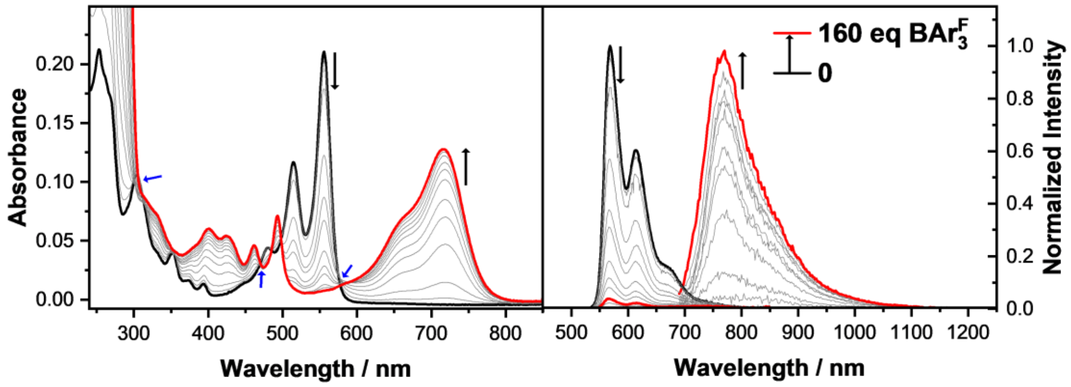

Figure S102. Complexation reaction of **2** and **BAr3F** and the absorption (left) and normalized emission (right) of the titration of **1** (4.5×10^{-6} M) with an excess of **BAr3F** (up to 160 eq). Blue arrows = isosbestic points.

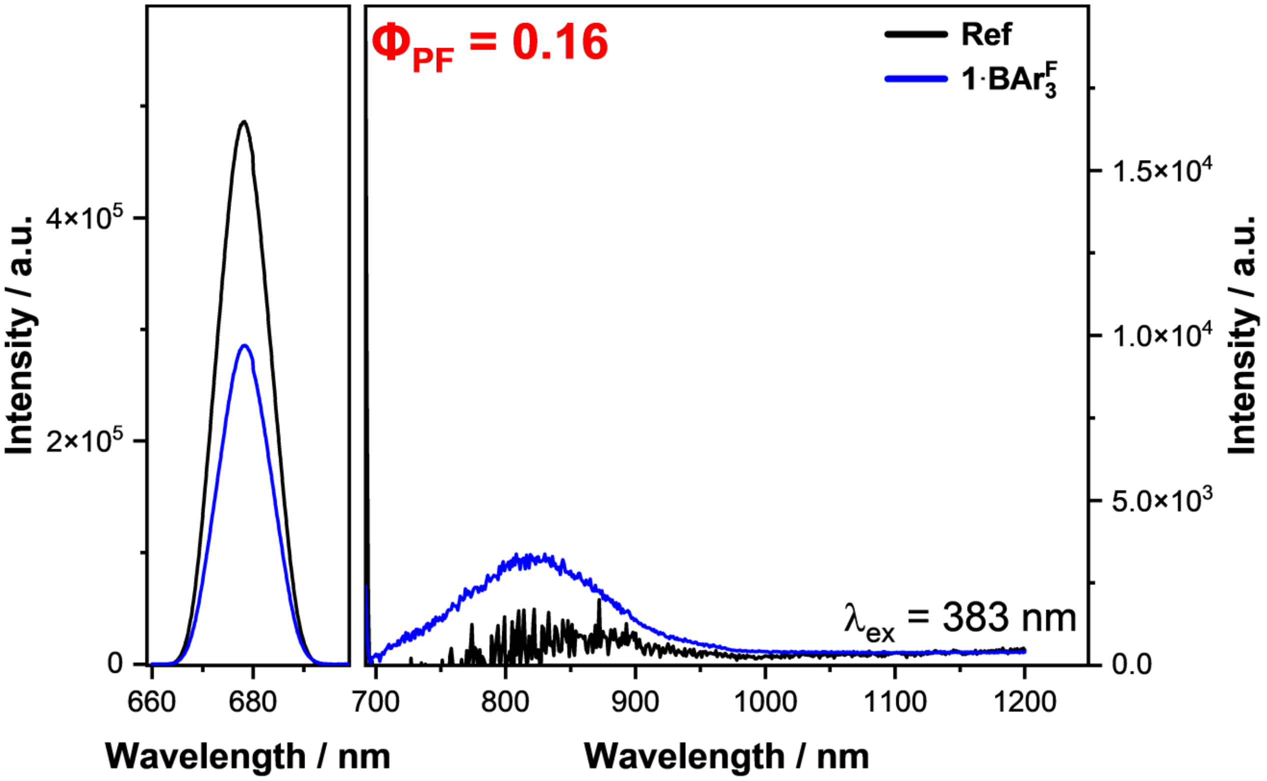

Figure S103. Excitation scatter region (left) and emission spectra (right) of **1·BAr$_3$^{F}** in CH_2Cl_2 used for the absolute quantum yield calculation.

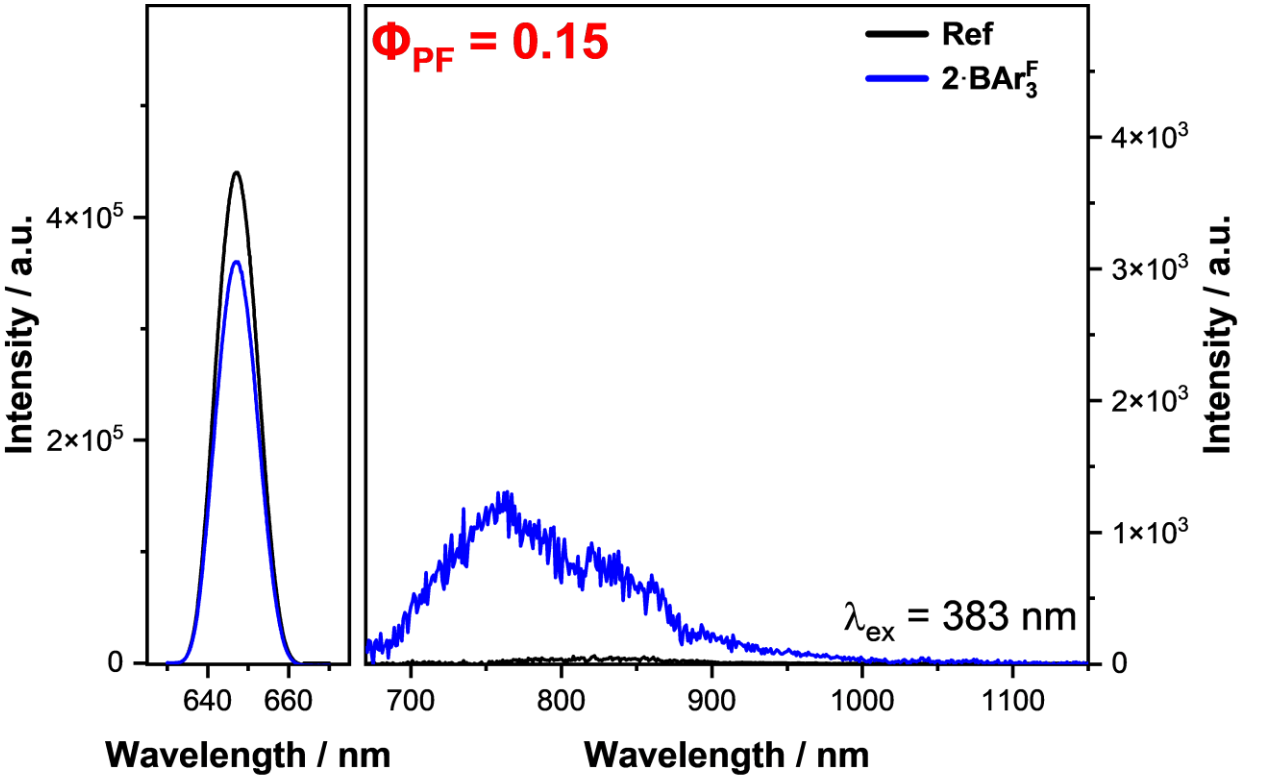

Figure S104. Excitation scatter region (left) and emission spectra (right) of **2·BAr$_3$^{F}** in CH_2Cl_2 used for the absolute quantum yield calculation.

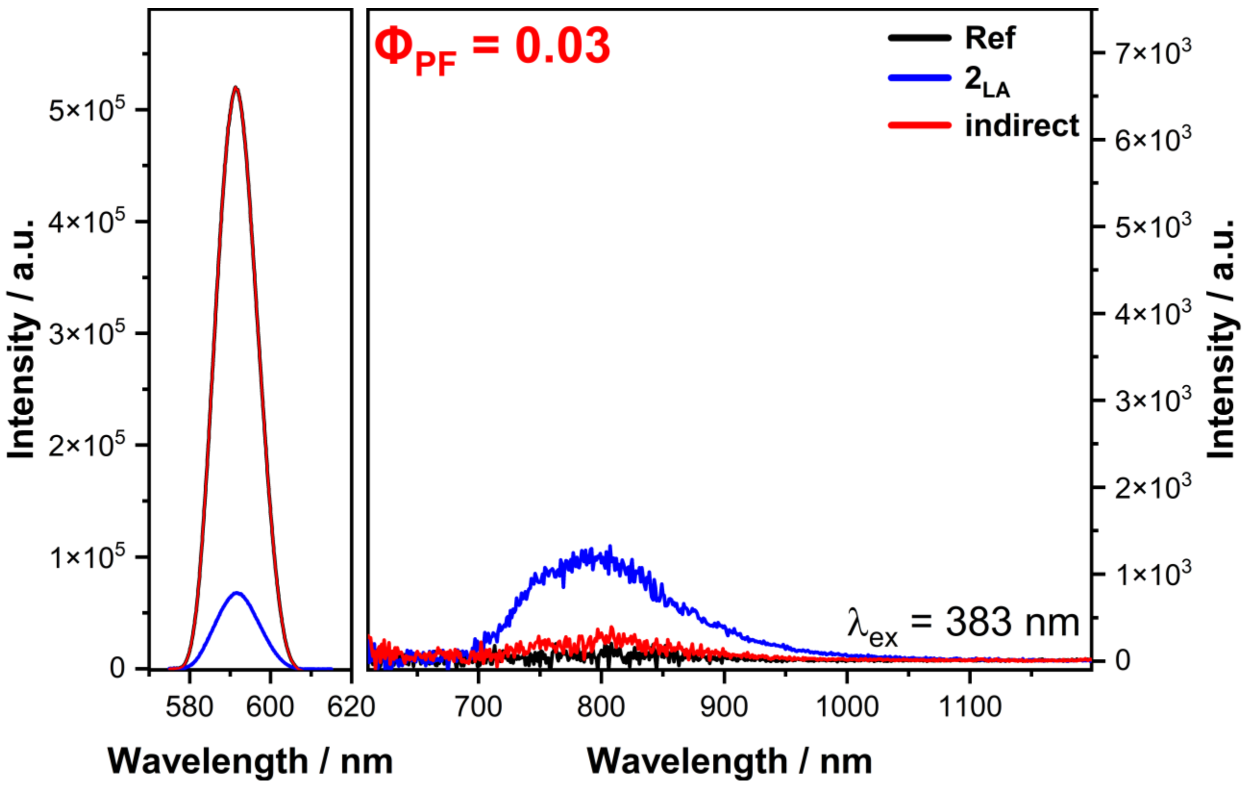

Figure S105. Excitation scatter region (left) and emission spectra (right) of solid **2·BAr$_3^F$** used for the absolute quantum yield calculation (via direct and indirect excitation method).

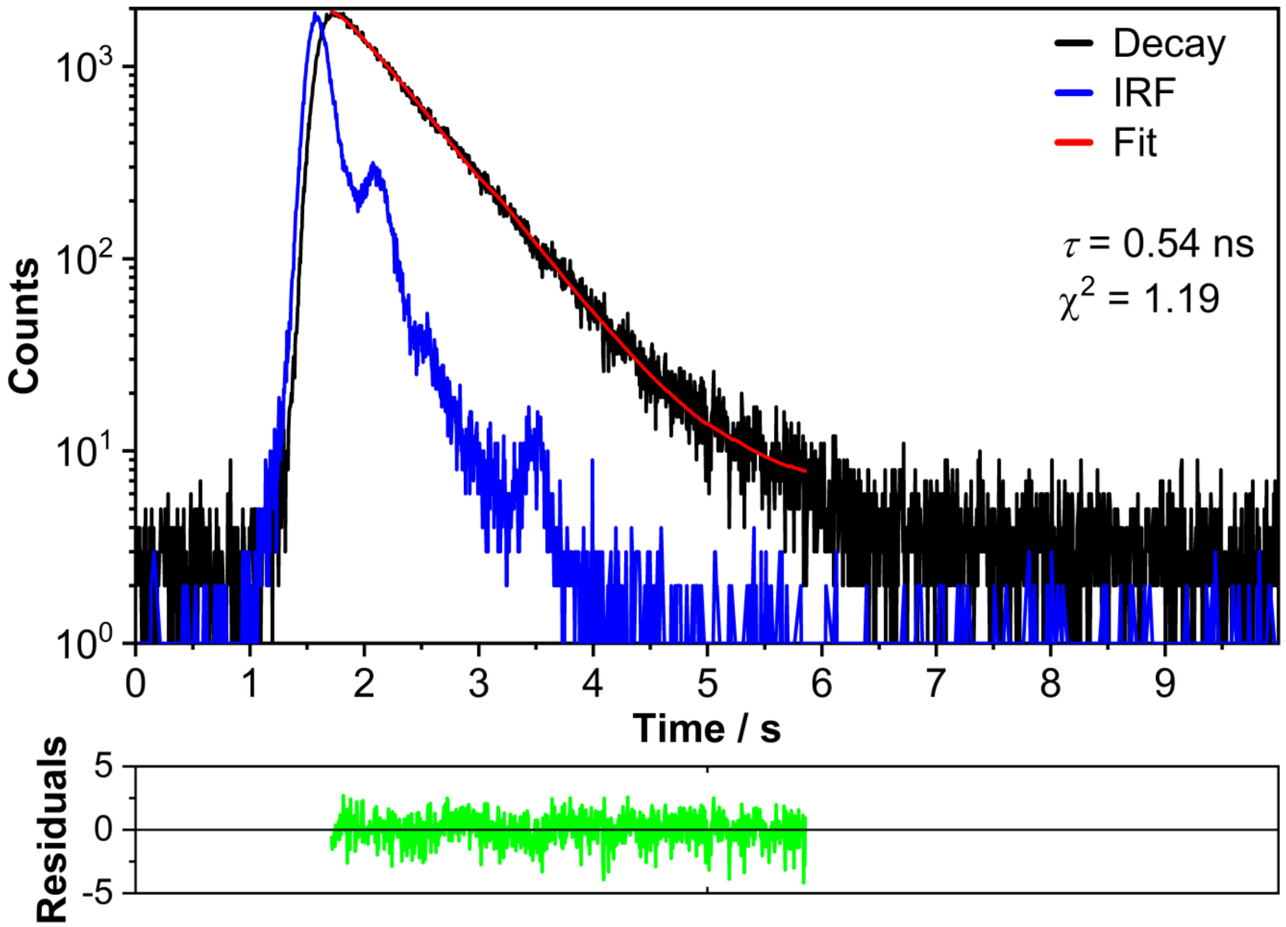

Figure S106. Time-resolved emission decay (λ_{ex} = 374.2 nm, λ_{em} = 790 nm) of **1·BAr$_3^F$** at RT in CH_2Cl_2.

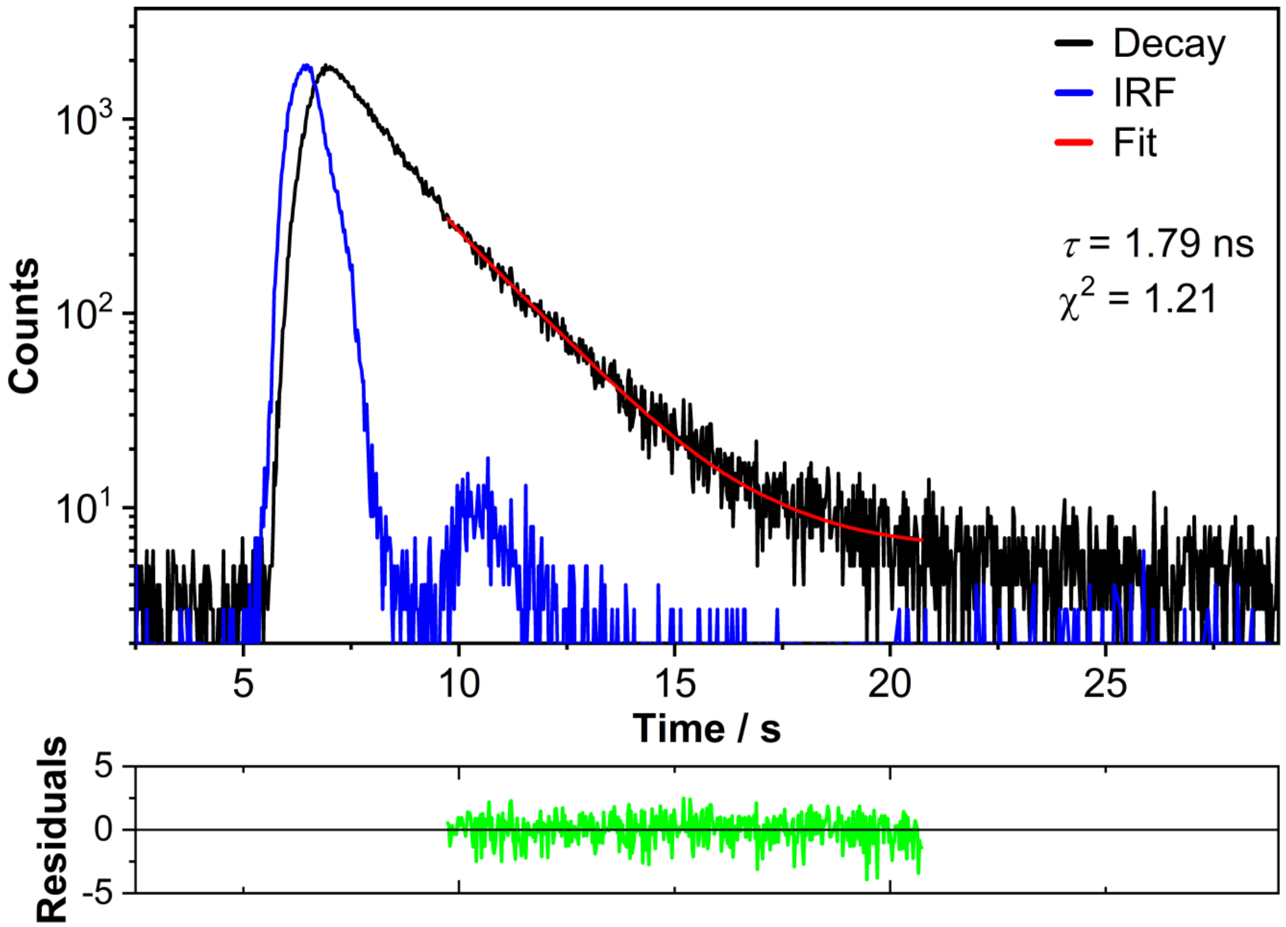

Figure S107. Time-resolved emission decay (λ_{ex} = 374.2 nm, λ_{em} = 764 nm) of **2·BAr$_3^F$** at RT in CH_2Cl_2.

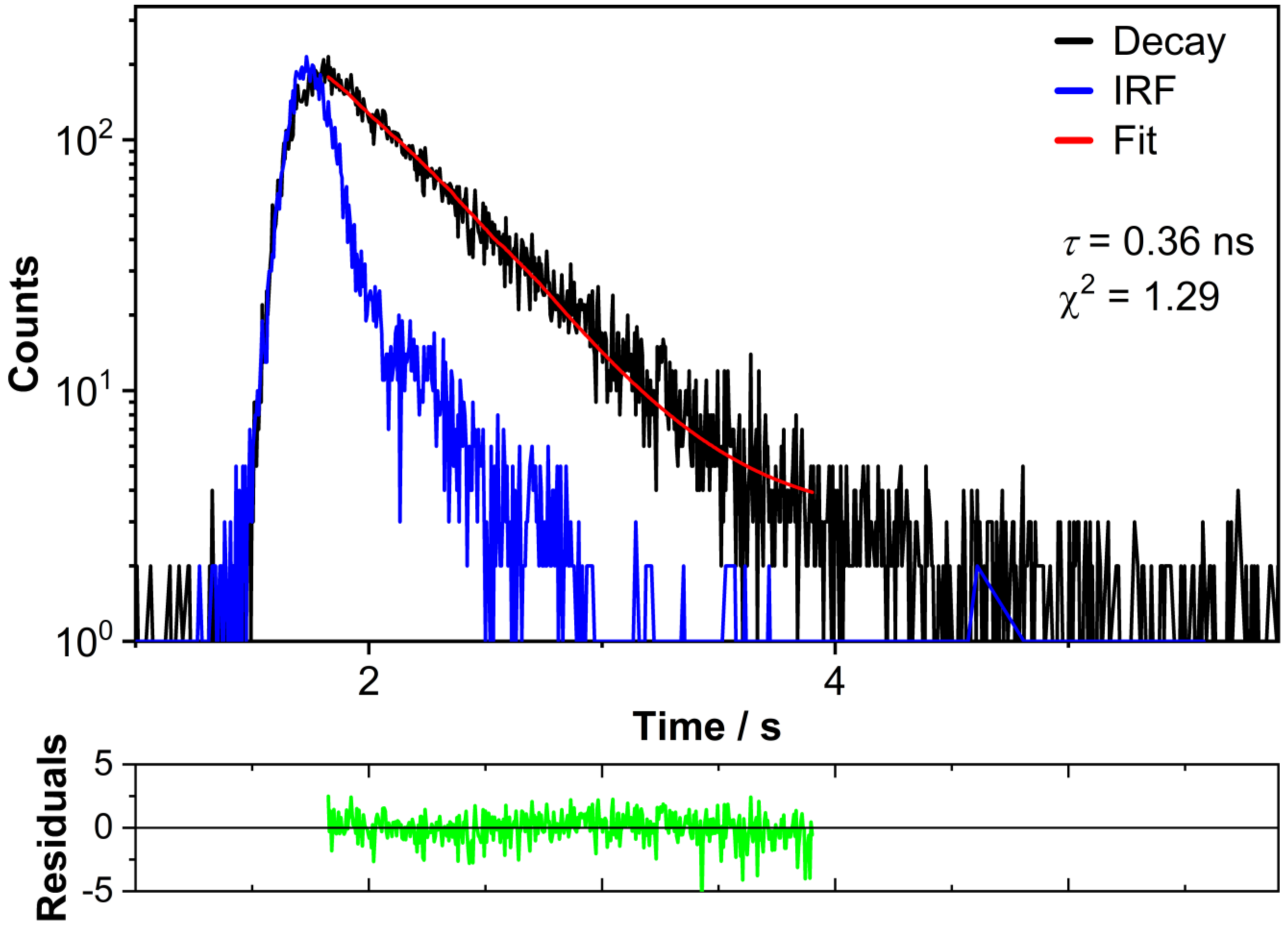

Figure S108. Time-resolved emission decay (λ_{ex} = 374.2 nm, λ_{em} = 850 nm) of **5·BAr$_3$^{F}** at RT in CH_2Cl_2.

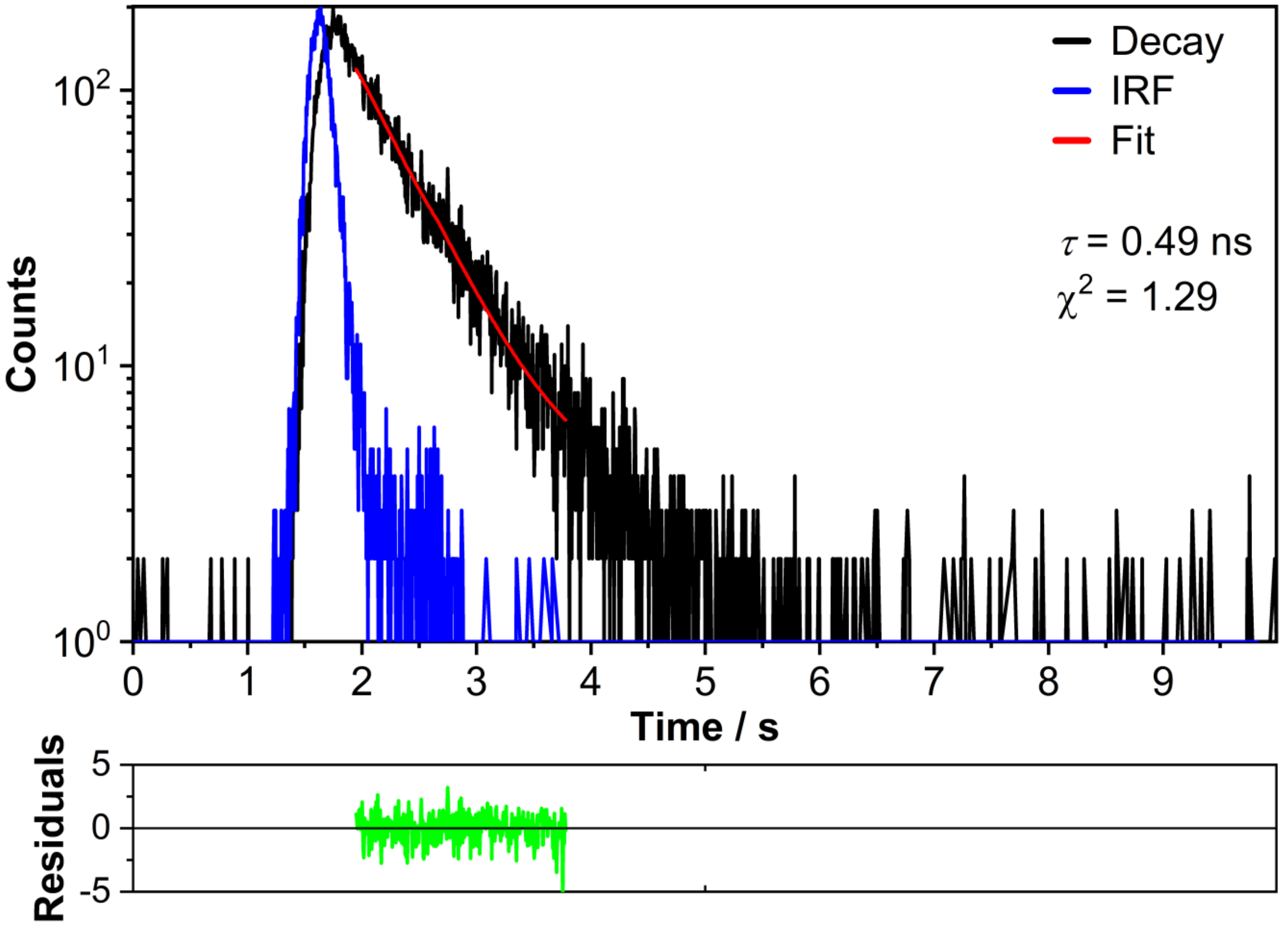

Figure S109. Time-resolved emission decay (λ_{ex} = 374.2 nm, λ_{em} = 840 nm) of solid **1·BAr$_3$^{F}** at RT.

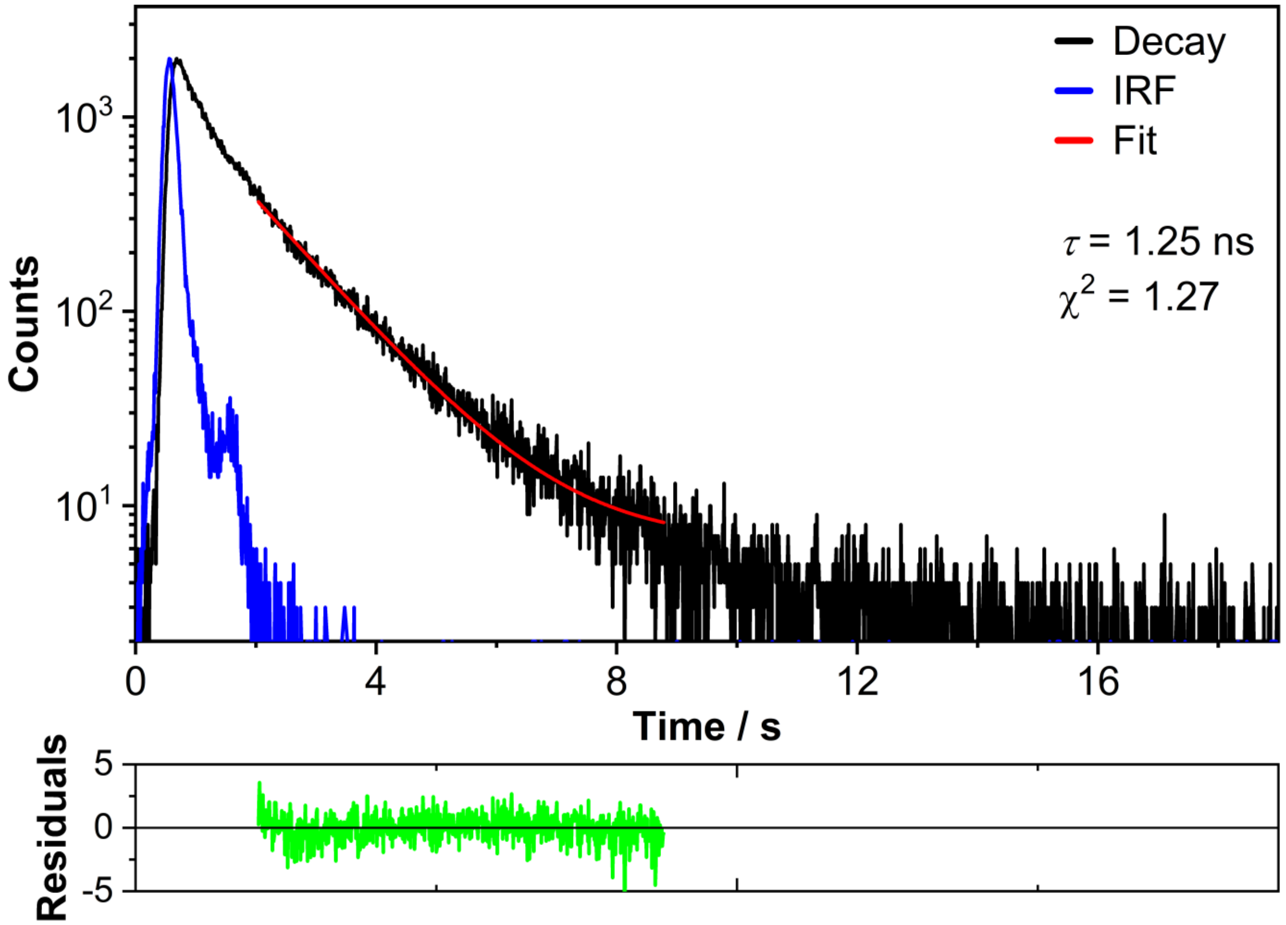

Figure S110. Time-resolved emission decay (λ_{ex} = 374.2 nm, λ_{em} = 722 nm) of solid **2·BAr$_3$^{F}** at RT.

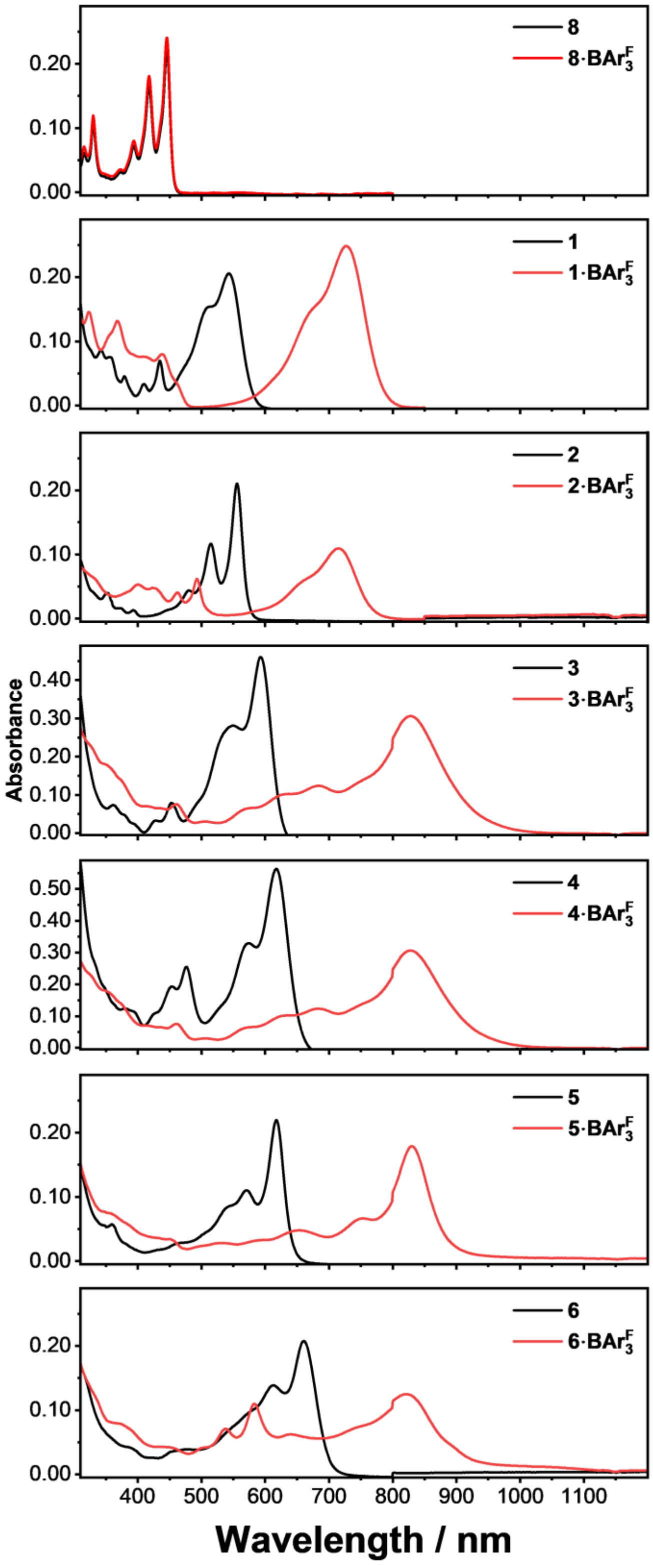

Figure S111. Absorbance spectra of the PXX derivatives (black) and their complexes with **BAr$_3^F$** (red) in CH_2Cl_2.

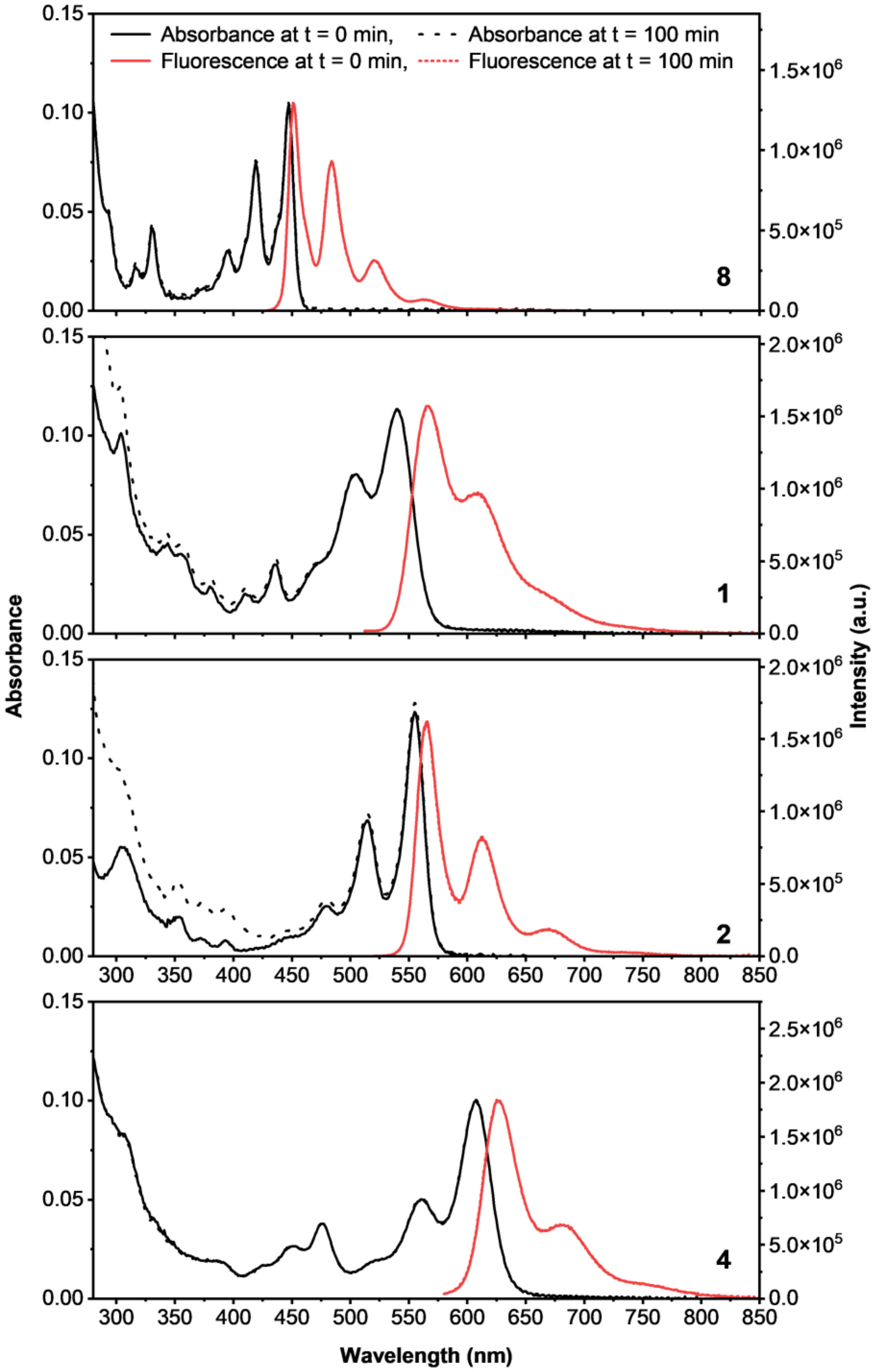

Figure S112. Absorbance and emission spectra of **8**–**4** before and after irradiation for 100 min in air equilibrated C_6H_6 at RT.

3.2. Electrochemical properties

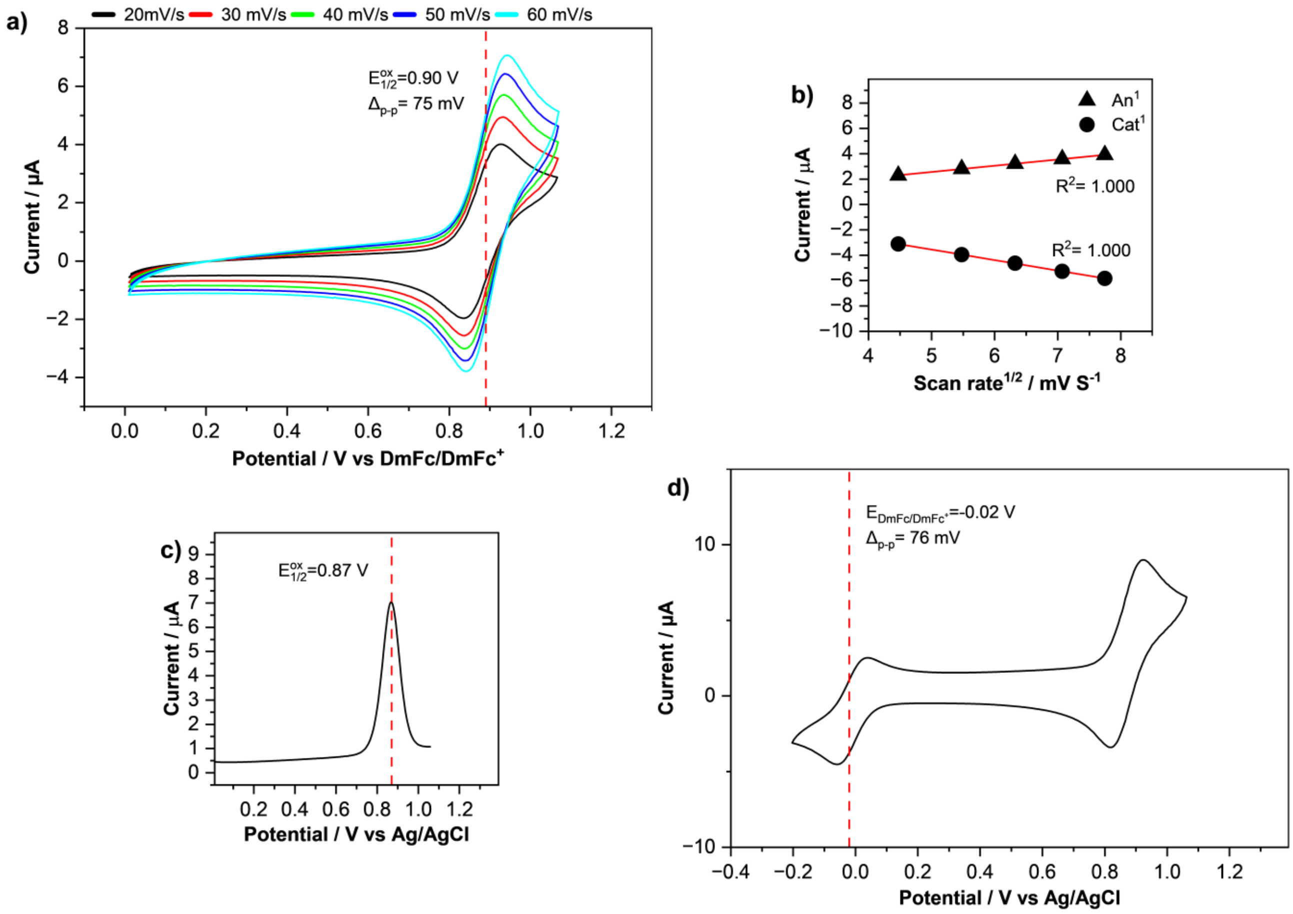

Figure S113. Cyclic voltammetry of 0.2 mM **8** in CH_2Cl_2: a) CV at different scan rates (20 – 60 mV/s); b) Linear dependence between cathodic peak current and scan rate$^{1/2}$; c) Differential pulse voltammetry of **8**; d) CV of **8** mixed with Decamethylferrocene as an internal reference (scan rate of 60 mV/s).

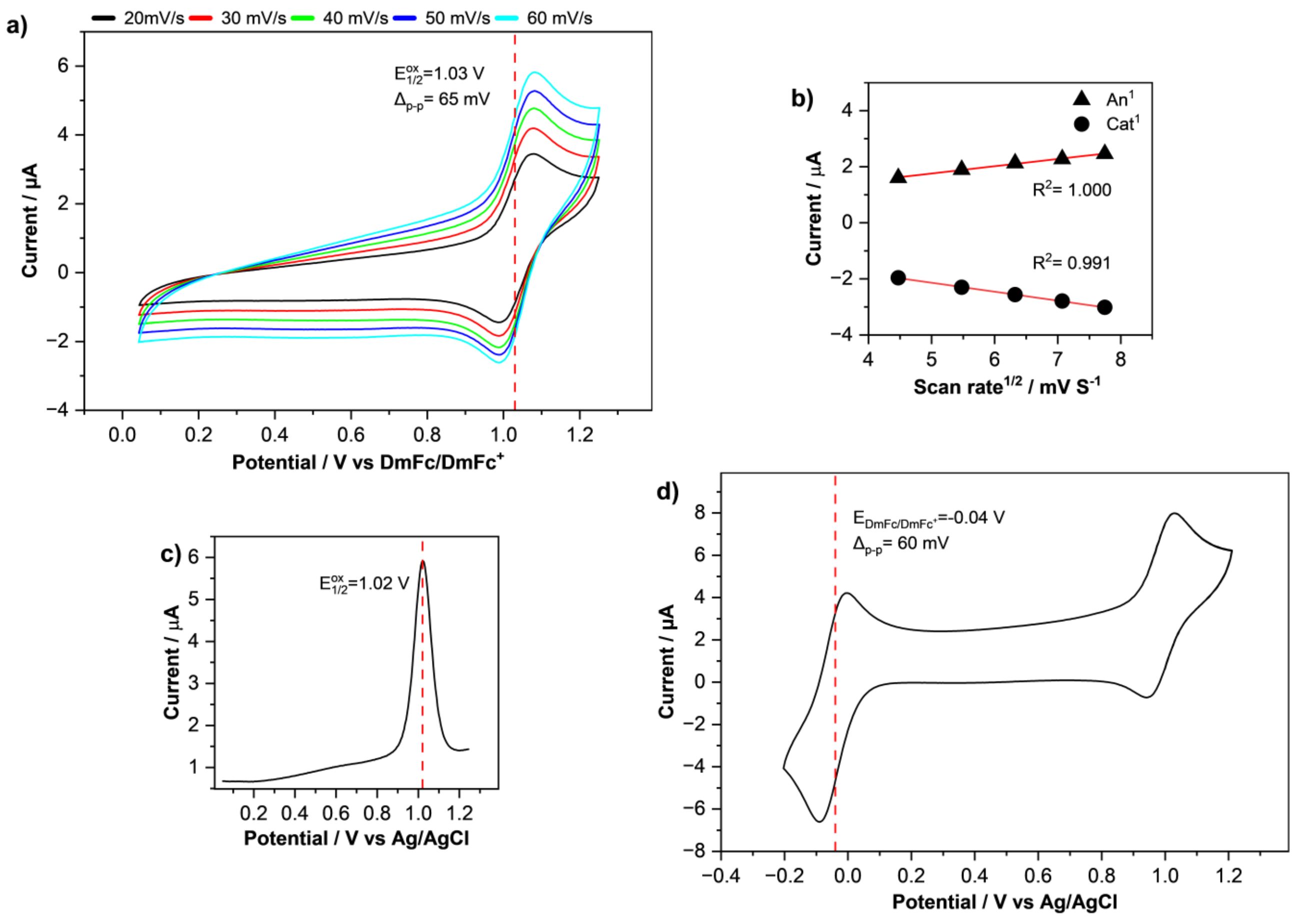

Figure S114. Cyclic voltammetry of 0.2 mM **1** in CH_2Cl_2: a) CV at different scan rates (20 – 60 mV/s); b) Linear dependence between cathodic peak current and scan rate$^{1/2}$; c) Differential pulse voltammetry of **1**; d) CV of **1** mixed with Decamethylferrocene as an internal reference (scan rate of 60 mV/s).

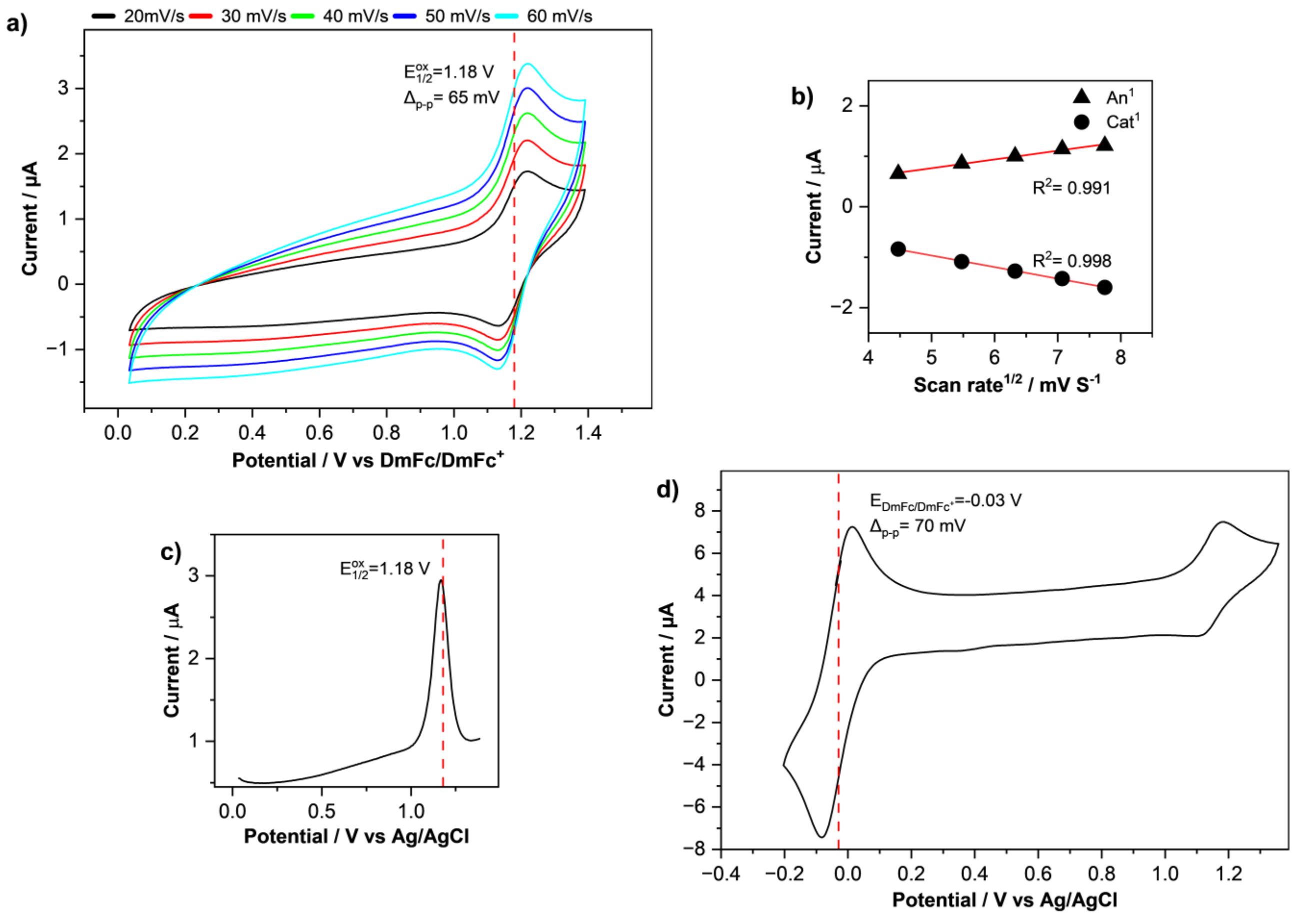

Figure S115. Cyclic voltammetry of 0.2 mM **2** in CH_2Cl_2: a) CV at different scan rates (20 – 60 mV/s); b) Linear dependence between cathodic peak current and scan rate$^{1/2}$; c) Differential pulse voltammetry of **2**; d) CV of **2** mixed with Decamethylferrocene as an internal reference (scan rate of 60 mV/s).

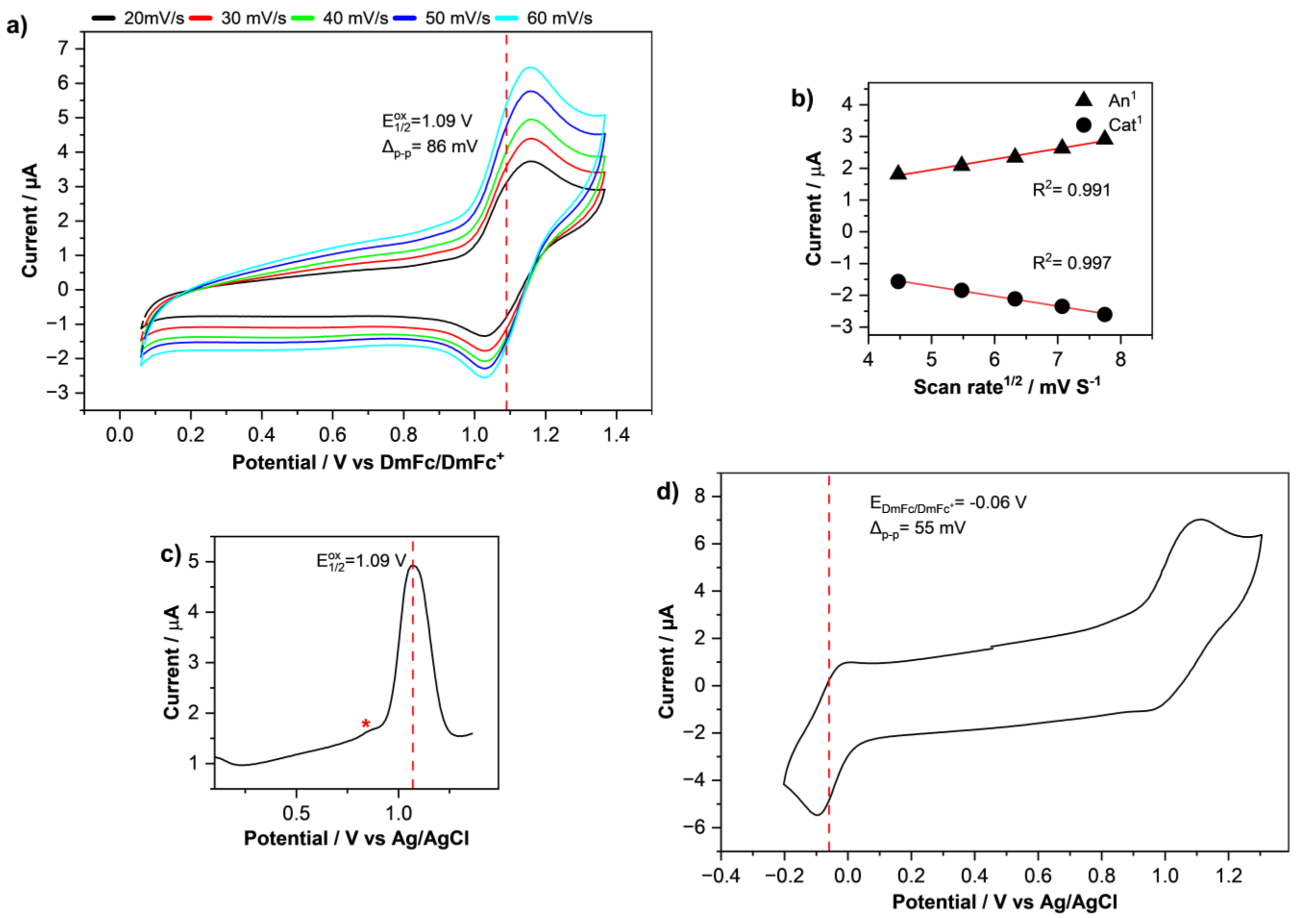

Figure S116. Cyclic voltammetry of 0.2 mM **3** in CH_2Cl_2: a) CV at different scan rates (20 – 60 mV/s); b) Linear dependence between cathodic peak current and scan rate$^{1/2}$; c) Differential pulse voltammetry of **3**; d) CV of **3** mixed with Decamethylferrocene as an internal reference (scan rate of 60 mV/s). *Appeared after applying higher potential to show the first irreversible oxidation.

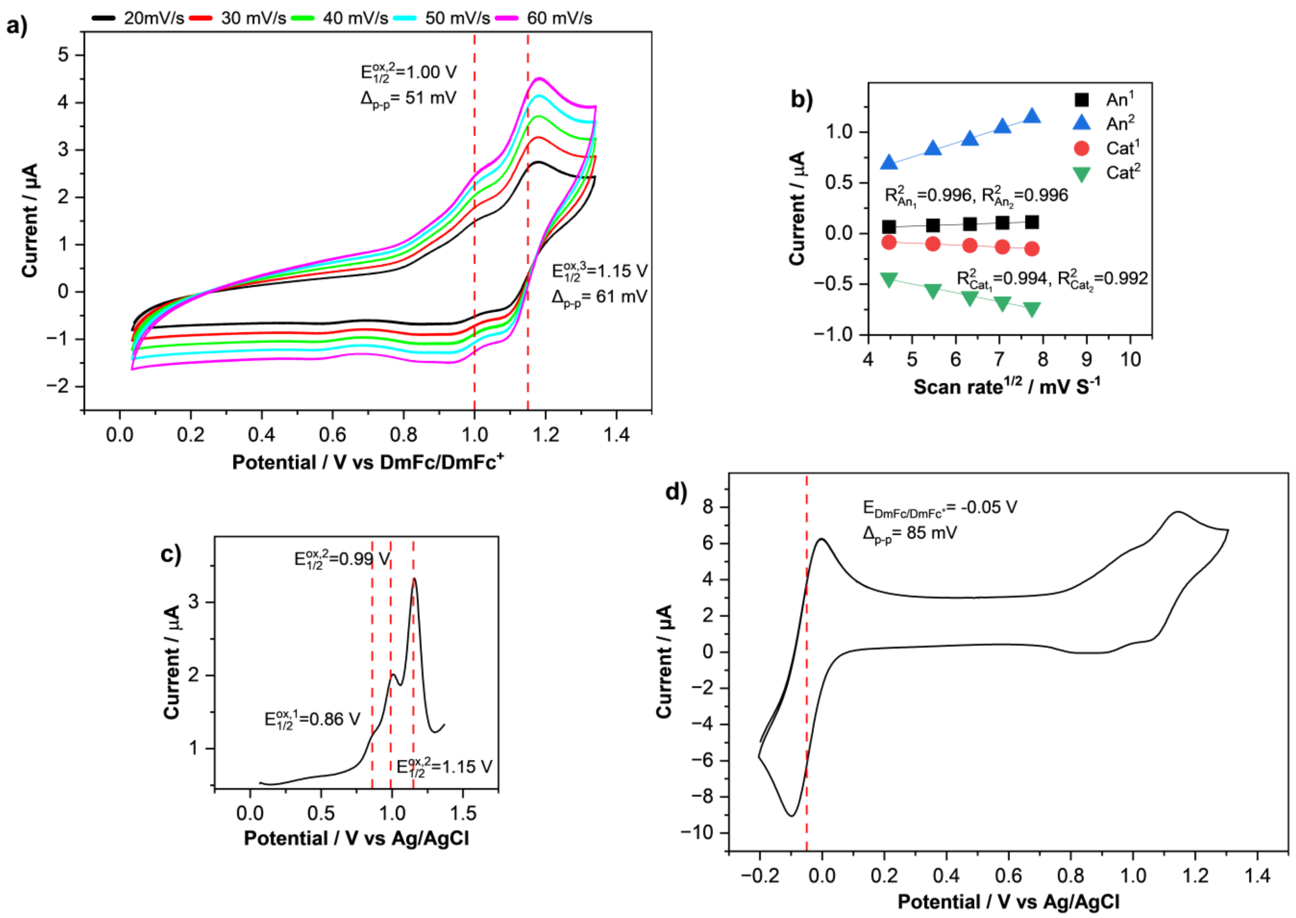

Figure S117. Cyclic voltammetry of 0.2 mM **4** in CH_2Cl_2: a) CV at different scan rates (20 – 60 mV/s); b) Linear dependence between cathodic peak current and scan rate$^{1/2}$; c) Differential pulse voltammetry of **4**; d) CV of **4** mixed with Decamethylferrocene as an internal reference (scan rate of 60 mV/s).

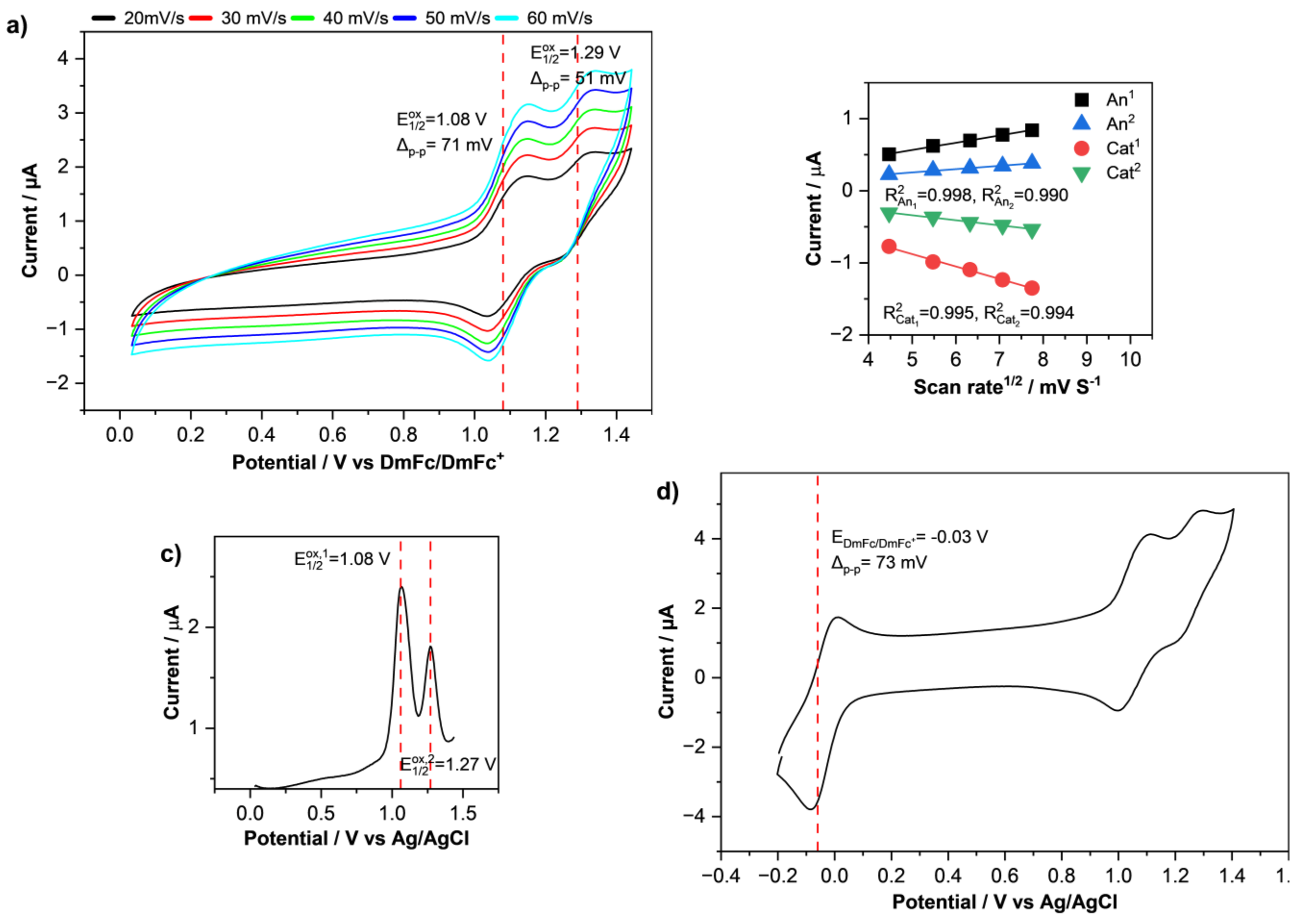

Figure S118. Cyclic voltammetry of 0.2 mM **5** in CH_2Cl_2: a) CV at different scan rates (20 – 60 mV/s); b) Linear dependence between cathodic peak current and scan rate$^{1/2}$; c) Differential pulse voltammetry of **5**; d) CV of **5** mixed with Decamethylferrocene as an internal reference (scan rate of 60 mV/s).

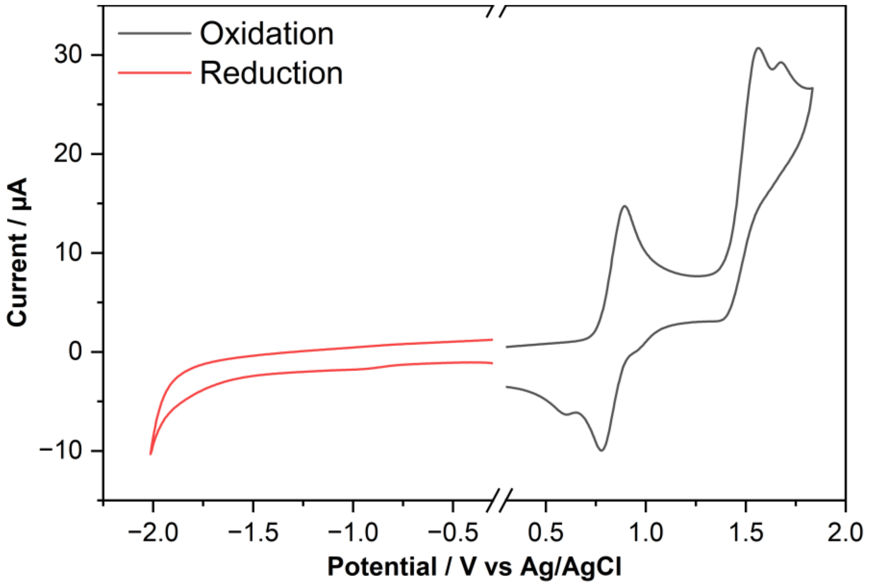

Figure S119. Cyclic voltammogram of 0.2 mM **8** from -2.0 to 1.8 V (scan rate = 60 mV/s). This demonstrates that there are no reversible oxidation events (beyond 1.25 V) and reversible reduction events.

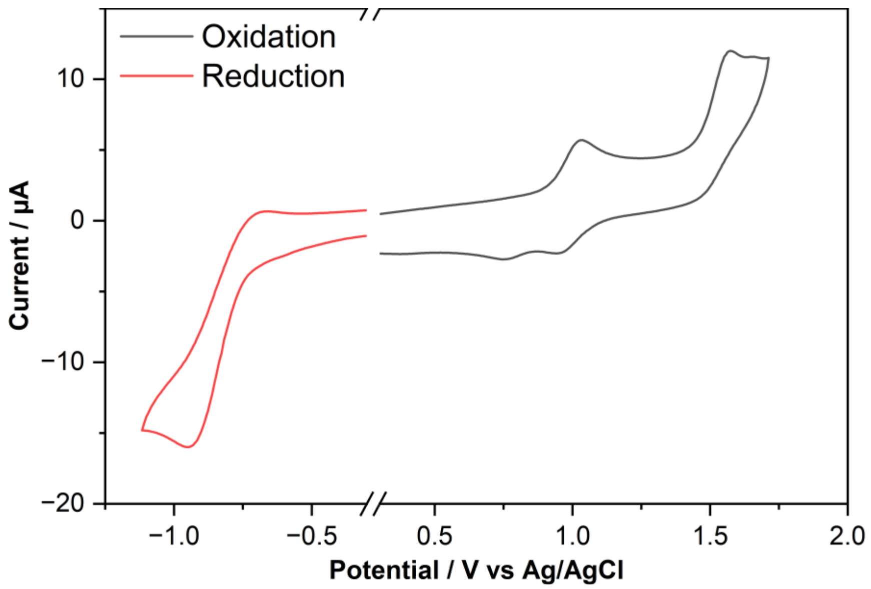

Figure S120. Cyclic voltammogram of 0.2 mM **1** from -1.2 to 1.8 V (scan rate = 60 mV/s). This demonstrates that there are no reversible oxidation events (beyond 1.25 V) and reversible reduction events.

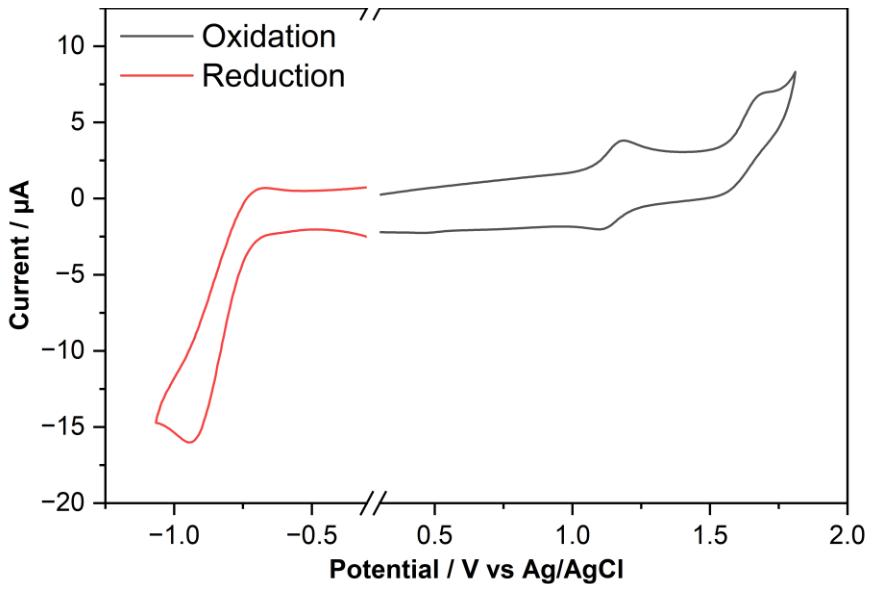

Figure S121. Cyclic voltammogram of 0.2 mM **2** from -1.2 to 1.8 V (scan rate = 60 mV/s),demonstrating that there are no reversible oxidation events beyond 1.40 V and reversible reduction events.

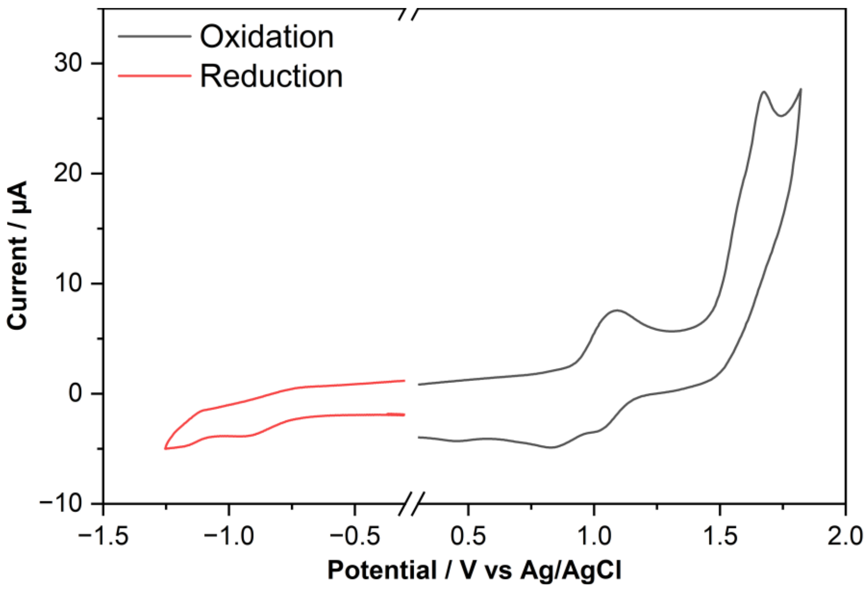

Figure S122. Cyclic voltammogram of 0.2 mM **3** from -1.25 to 1.8 V (scan rate = 60 mV/s). This demonstrates that there are no reversible oxidation events (beyond 1.4 V) and reversible reduction events.

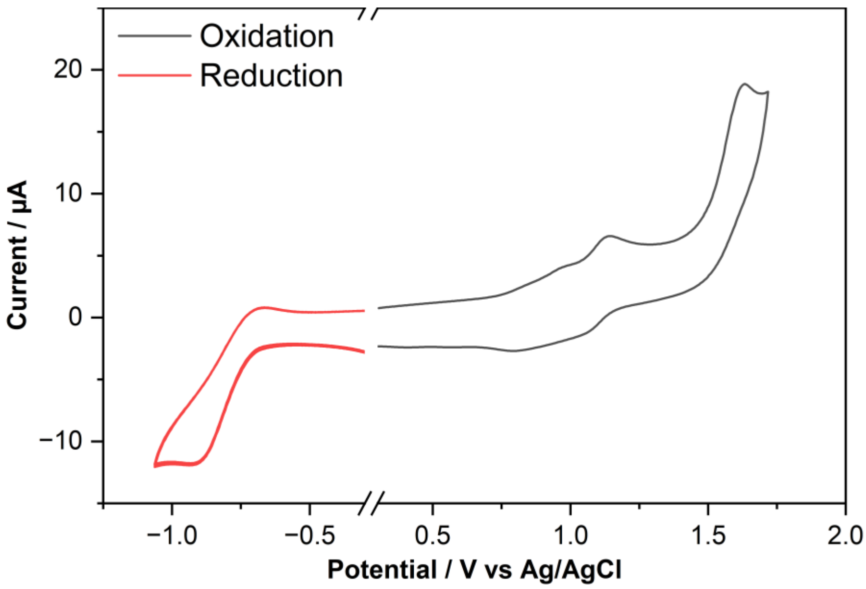

Figure S123. Cyclic voltammogram of 0.2 mM **4** from -1.1 to 1.8 V (scan rate = 60 mV/s). This demonstrates that there are no reversible oxidation events (beyond 1.4 V) and reversible reduction events.

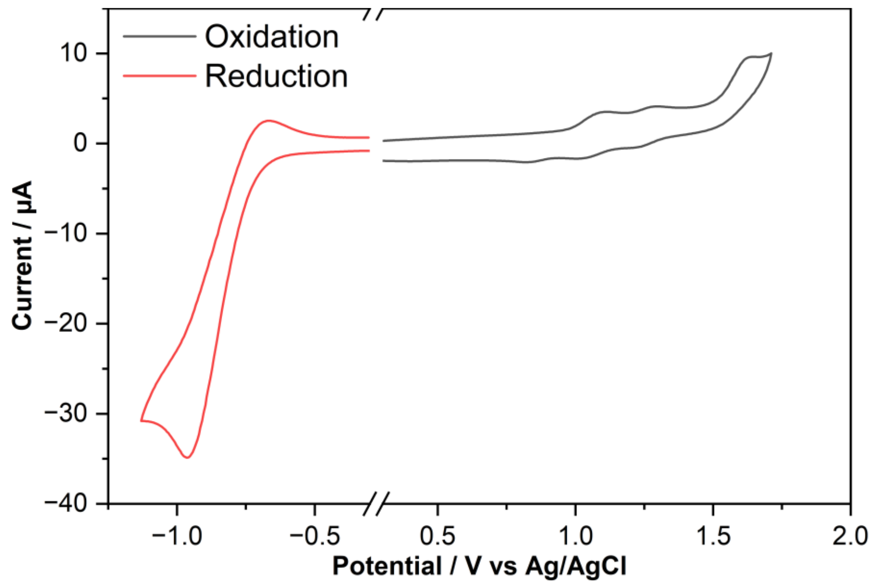

Figure S124. Cyclic voltammogram of 0.2 mM **5** from -1.2 to 1.75 V (scan rate = 60 mV/s). This demonstrates that there are no reversible oxidation events (beyond 1.4 V) and reversible reduction events.

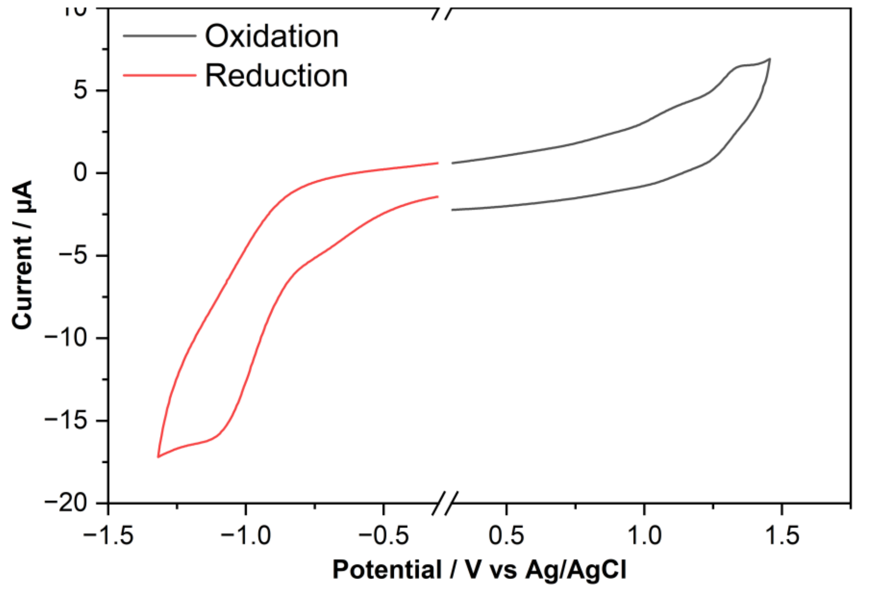

Figure S125. Cyclic voltammogram of 0.2 mM **6** from -1.3 to 1.45 V (scan rate = 60 mV/s). This demonstrates that there are no confirmed reversible oxidation and reversible reduction events.

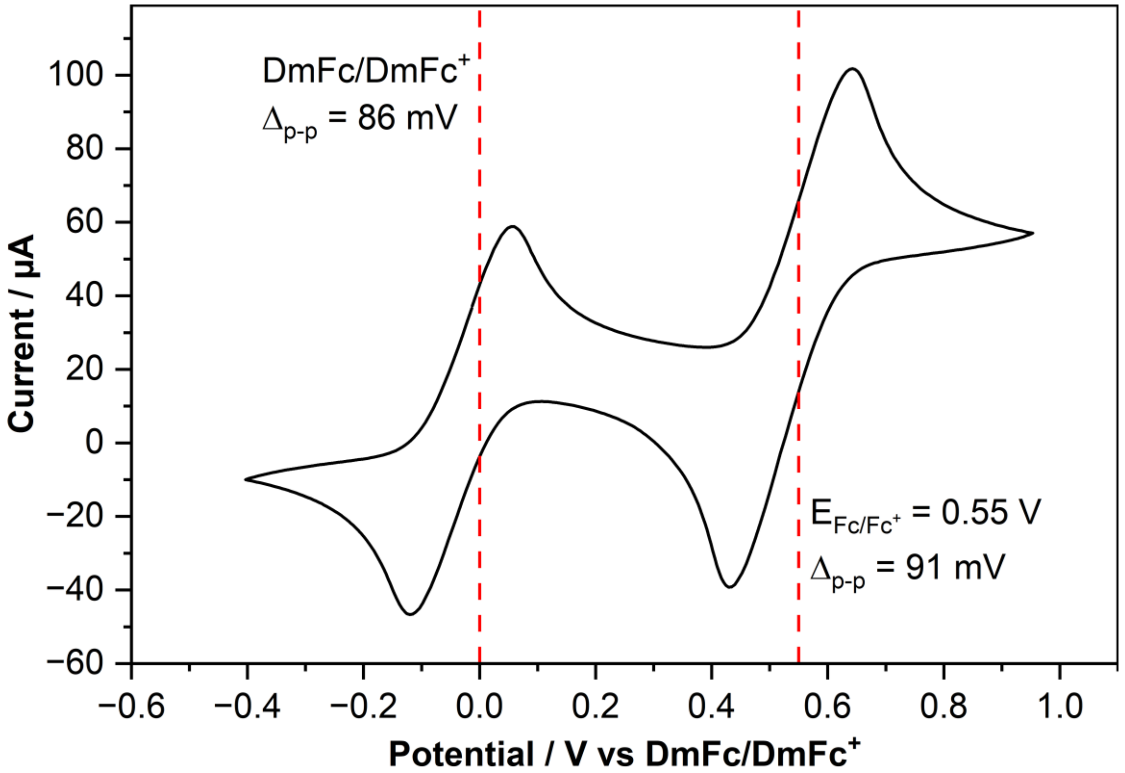

Figure S126. Cyclic voltammetry of Ferrocene in CH_2Cl_2. Scan rate: 60 mV/s. The potential of the Fc/Fc^+ was determined against $DmFc/DmFc^+$ to calculate the frontier orbital energies.

4. Preparation and characterization of the light-harvesting systems (LHS)

To prepare the light-harvesting system, all the components were dissolved in 2 mL of CH_2Cl_2 and then heated at 60 °C to evaporate CH_2Cl_2. The resulting samples were heated to 60 °C and cooled down to room temperature, this process was repeated three times.

4.1 Photophysical characterization of nanoribbons in 5CB

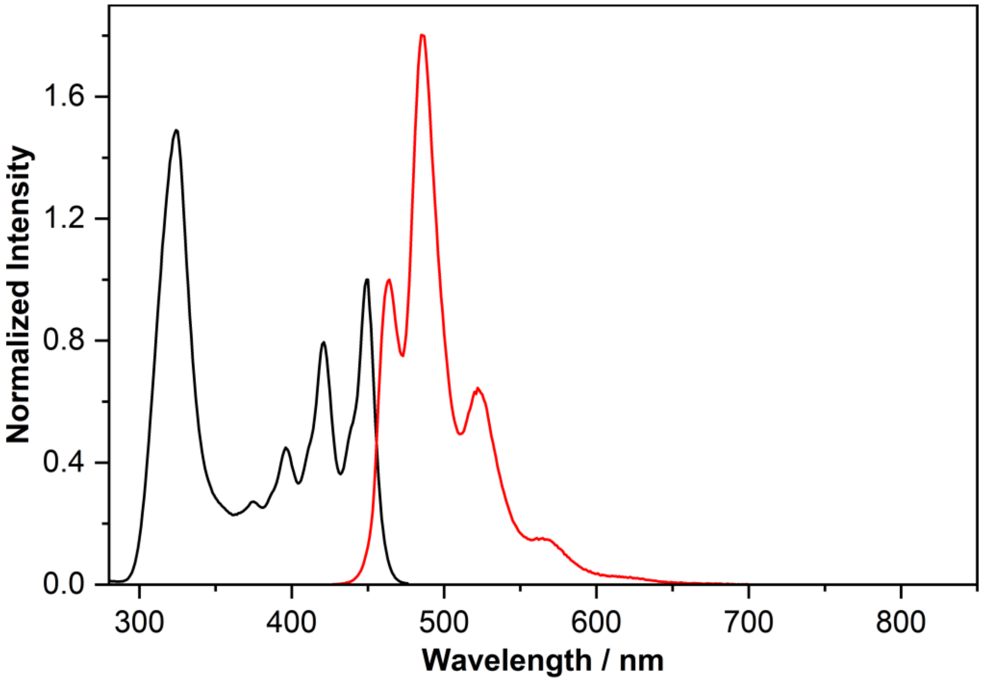

Figure S127. Normalized steady-state excitation (black, λ_{em} = 487 nm) and emission spectra (red, λ_{ex} = 417 nm) of **8^{1000}@5CB** at RT.

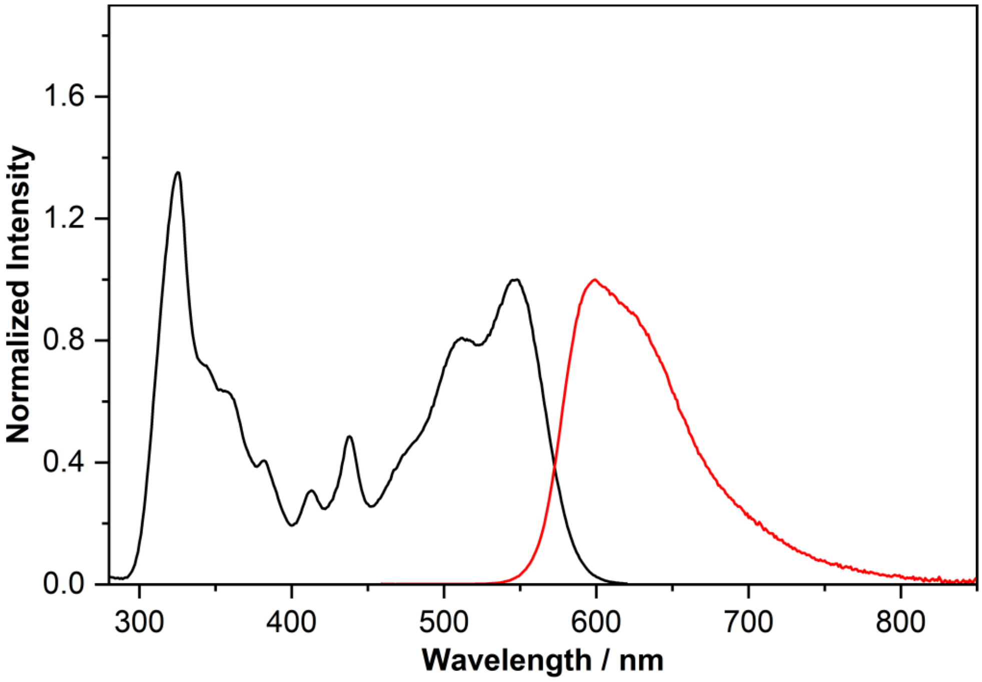

Figure S128. Normalized steady-state excitation (black, λ_{em} = 630 nm) and emission spectra (red, λ_{ex} = 449 nm) of **1^{500}@5CB** at RT.

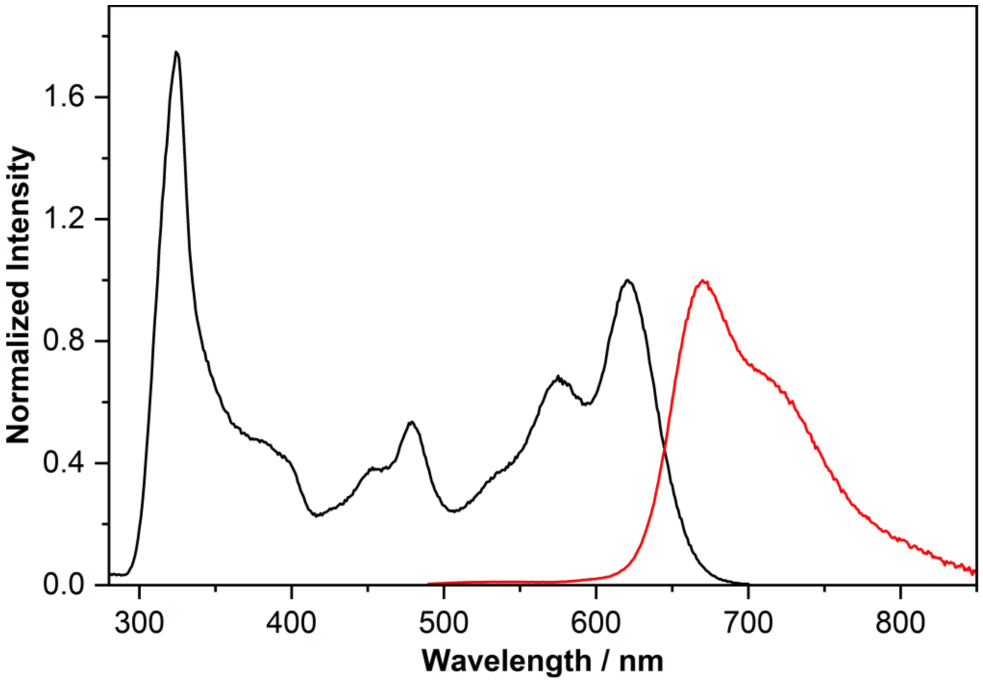

Figure S129. Normalized steady-state excitation (black, λ_{em} = 630 nm) and emission spectra (red, λ_{ex} = 449 nm) of **4^{250}@5CB** at RT.

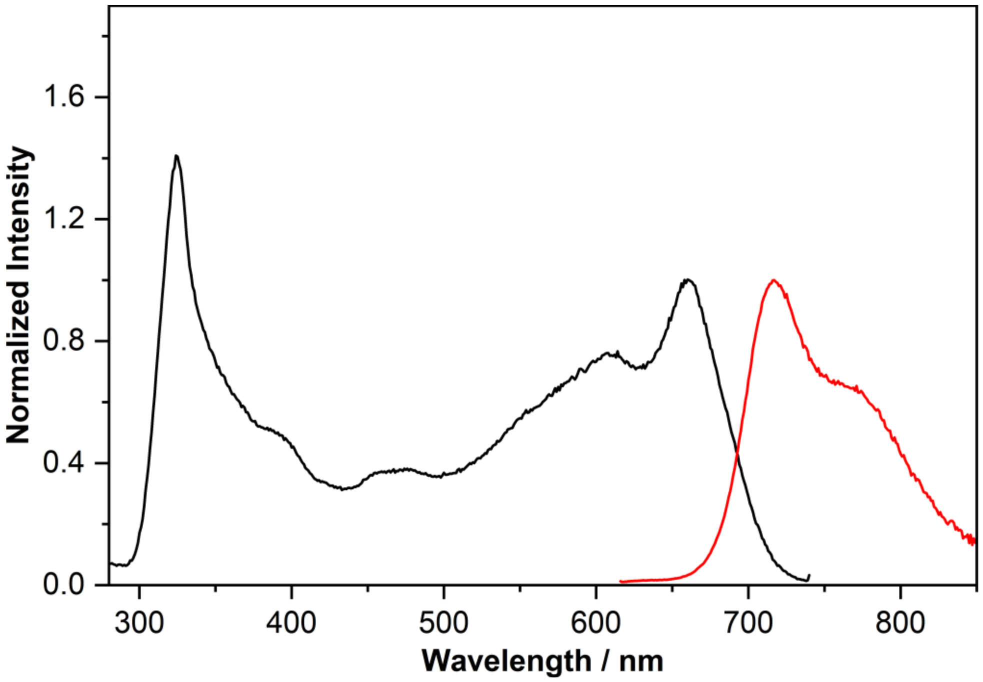

Figure S130. Normalized steady-state excitation (black, λ_{em} = 740 nm) and emission spectra (red, λ_{ex} = 605 nm) of **6^{250}@5CB** at RT.

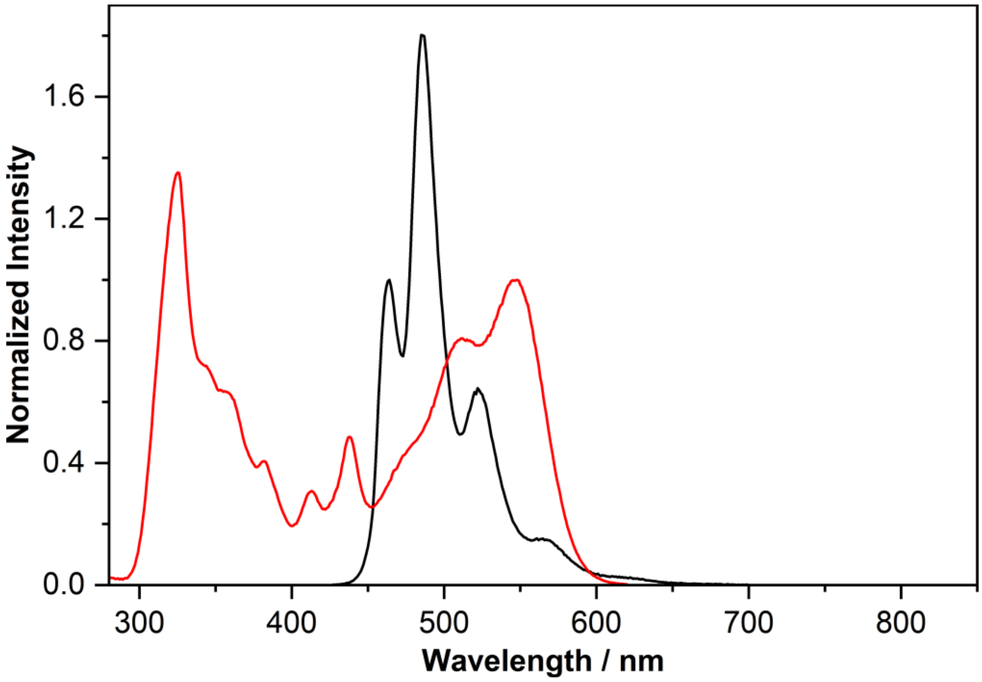

Figure S131. Spectral overlap of the normalized steady-state excitation spectrum (red; λ_{em} = 630 nm) **1** and emission spectrum (black; λ_{ex} = 417 nm) of **8** in **5CB** at RT.

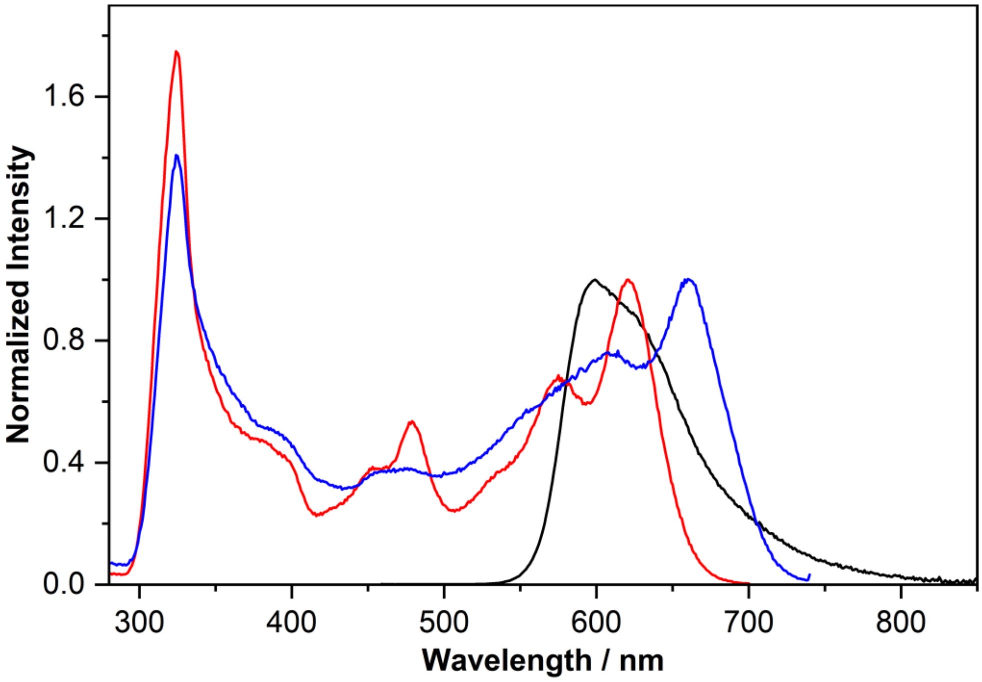

Figure S132. Spectral overlap of the normalized steady-state excitation spectrum **4** (red; λ_{em} = 710 nm) and **6** (blue; λ_{em} = 750 nm) and emission spectrum (black; λ_{ex} = 449 nm) of **1** in **5CB** at RT.

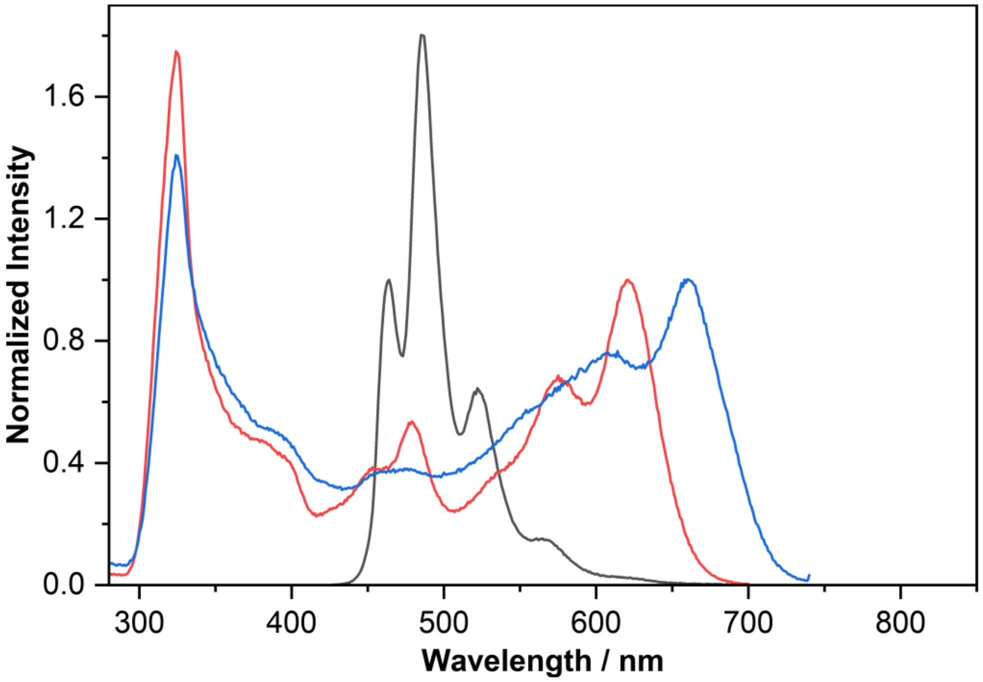

Figure S133. Spectral overlap of the normalized steady-state excitation spectrum **4** (red; λ_{em} = 710 nm) and **6** (blue; λ_{em} = 750 nm) and emission spectrum (black λ_{ex} = 417 nm) of **8** in **5CB** at RT.

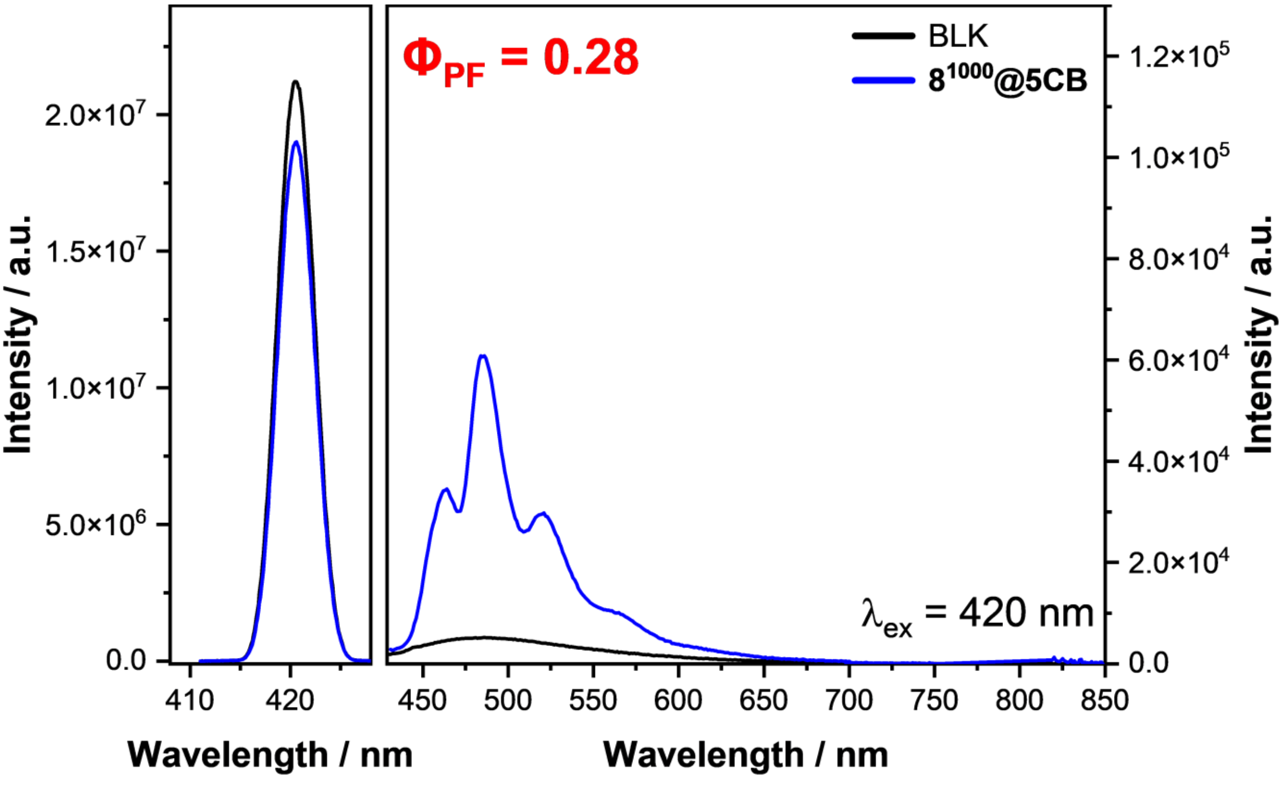

Figure S134. Excitation scatter region (left) and emission spectra (right) of **8**^{1000}**@5CB** at RT.

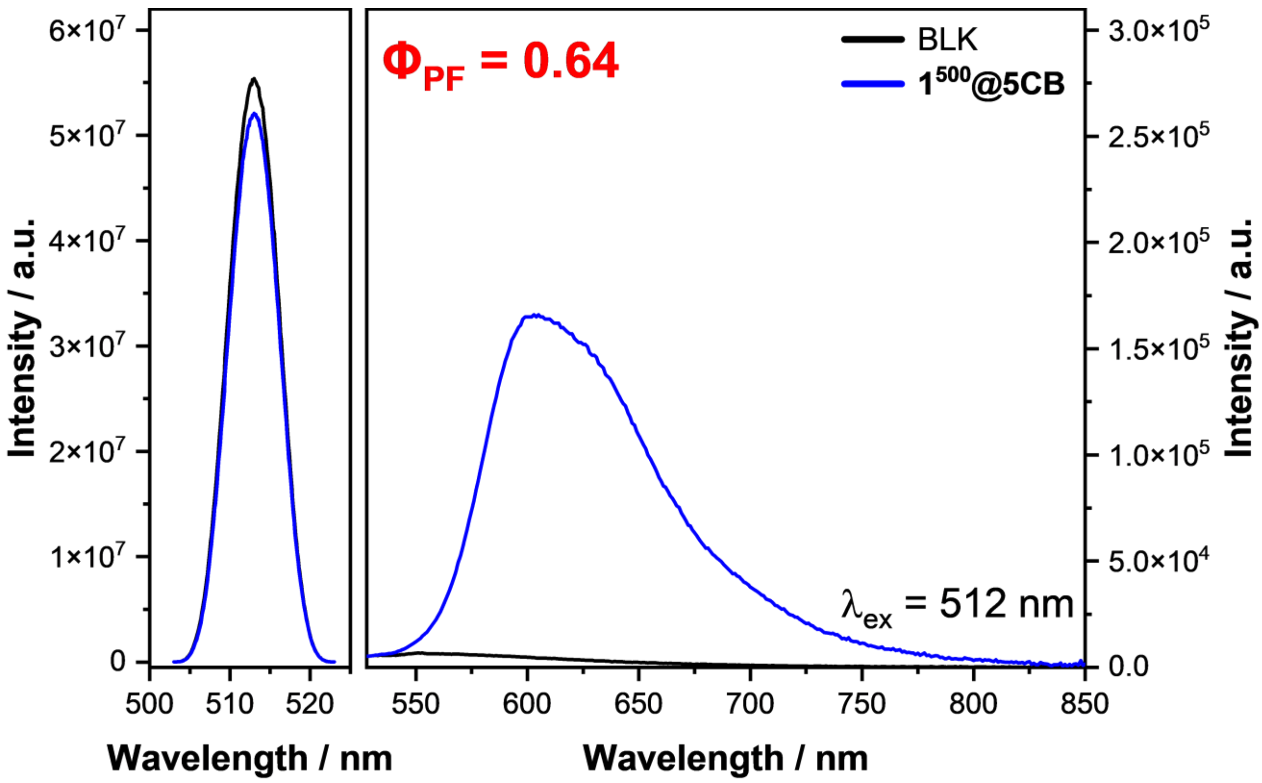

Figure S135. Excitation scatter region (left) and emission spectra (right) of **1^{500}@5CB** at RT.

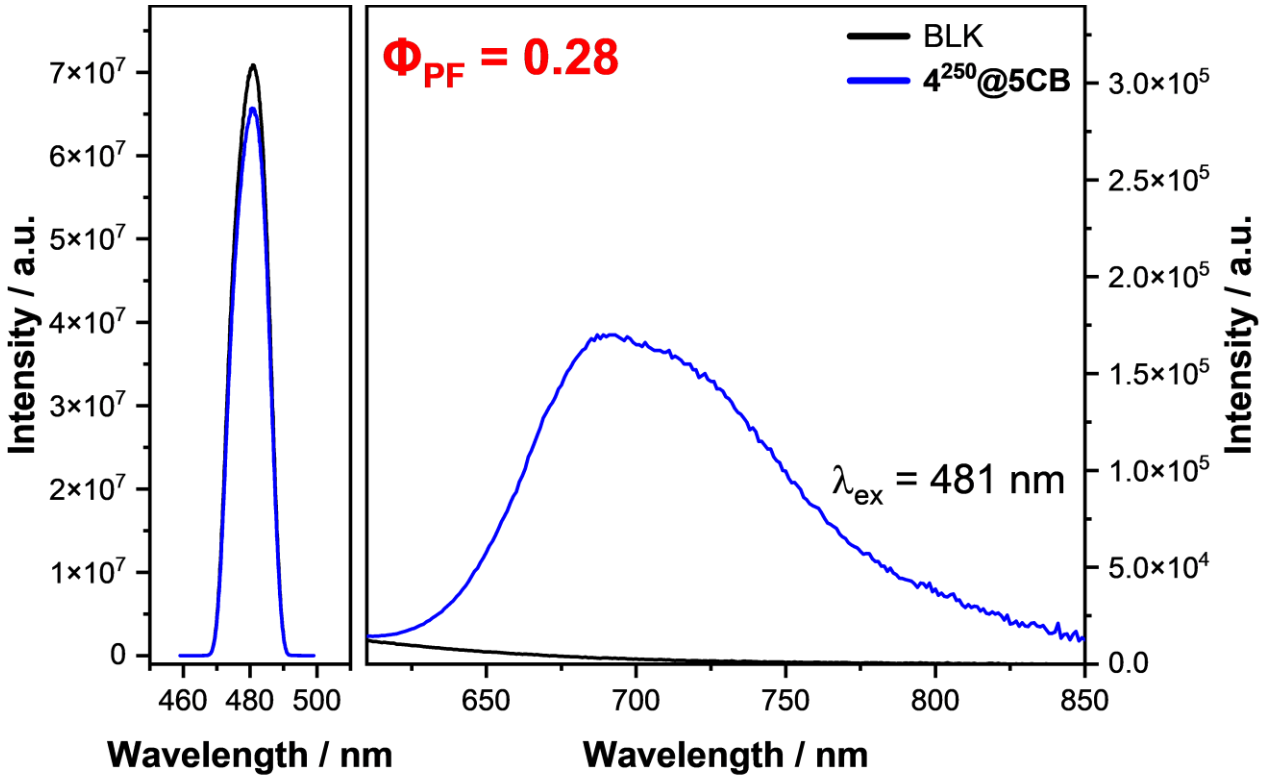

Figure S136. Excitation scatter region (left) and emission spectra (right) of **4^{250}@5CB** at RT.

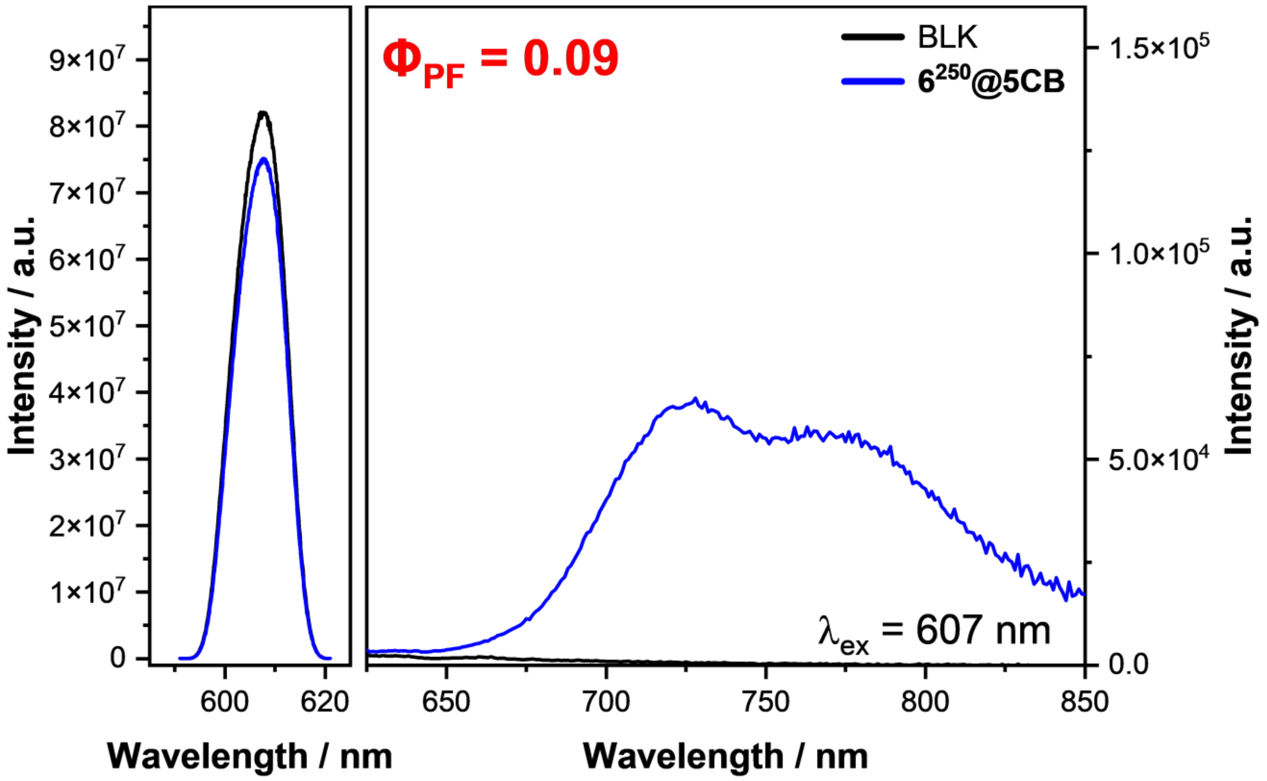

Figure S137. Excitation scatter region (left) and emission spectra (right) of **6^{250}@5CB** at RT.

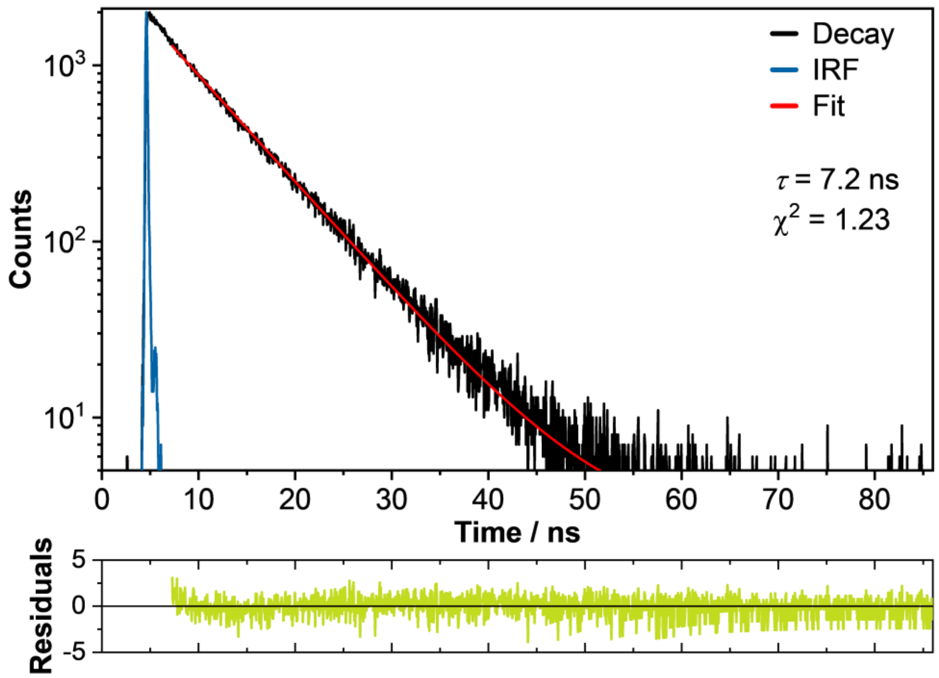

Figure S138. Time-resolved emission decay (λ_{ex} = 374.2 nm, λ_{em} = 484 nm) of **8^{1000}@5CB** at RT.

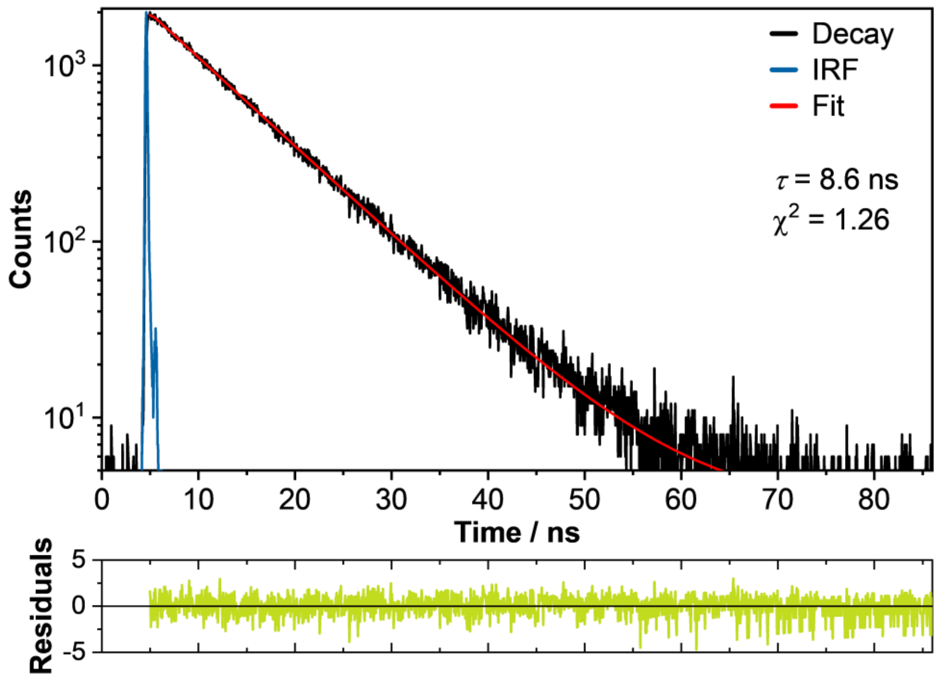

Figure S139. Time-resolved emission decay (λ_{ex} = 374.2 nm, λ_{em} = 600 nm) of **1^{500}@5CB** at RT.

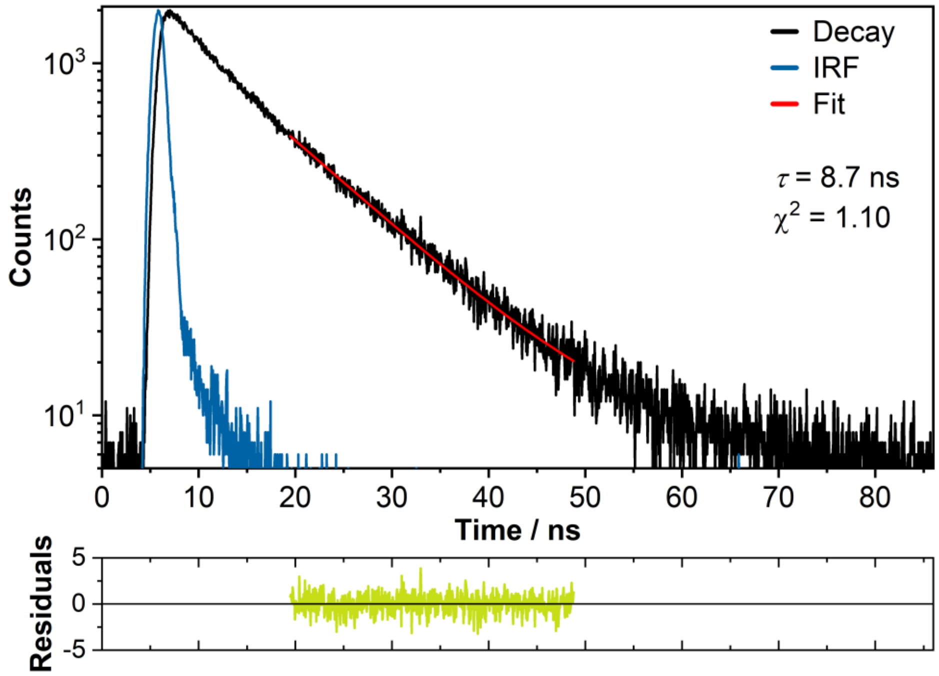

Figure S140. Time-resolved emission decay (λ_{ex} = 612.6 nm, λ_{em} = 670 nm) of **4^{250}@5CB** at RT.

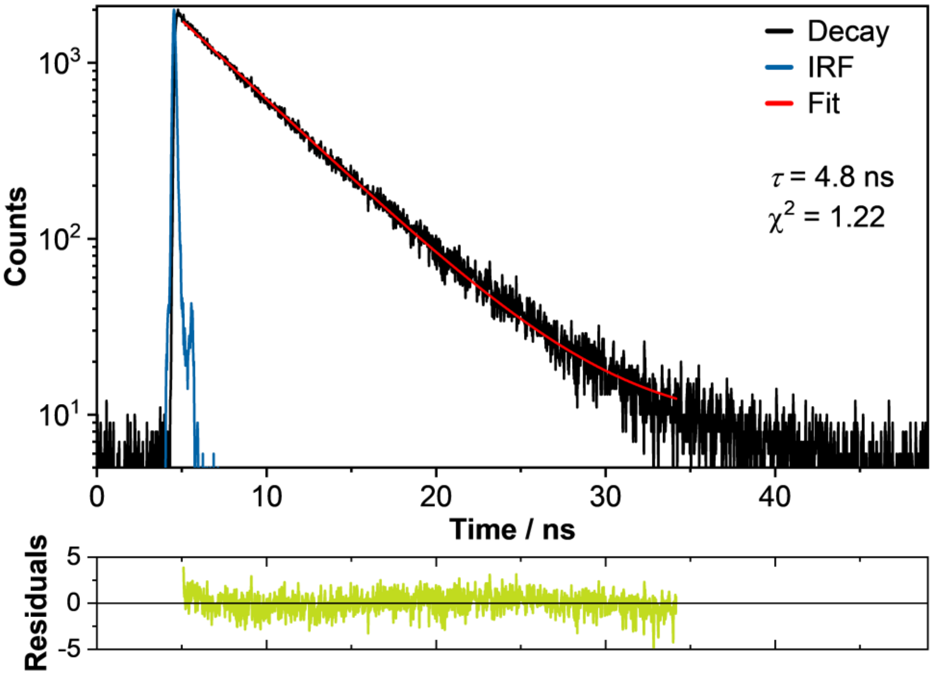

Figure S141. Time-resolved emission decay (λ_{ex} = 505 nm, λ_{em} = 717 nm) of **6^{250}@5CB** at RT.

4.2 Photophysical characterization of light-harvesting systems

The FRET efficiency is defined as the fraction of the absorbed light that is transferred from the donor to the acceptor.[14] It was calculated based on the fluorescence intensities of the donor alone (I_D) and with the acceptor (I_{DA}).

$$\Phi_{FRET}=1-\frac{I_{DA}}{I_D}$$

where I_{DA} and I_D are the fluorescence intensities ($\boldsymbol{\Phi_{FRET,8\ or\ 2}}$) or integrated area ($\boldsymbol{\Phi_{FRET,8}}$) of the donor alone and donor with the acceptor, respectively.

The antenna effect was calculated based on the fluorescence intensities of the acceptor when excited at the maximum absorption of the donor and the maximum absorption of the acceptor.

$$\text{Antenna effect} = \frac{I_D - I_{D,cor}}{I_A - I_{A,cor}}$$

where I_D = emission intensity of the acceptor upon excitation at the λ_{max} of donor, $I_{D,cor}$ = correction for the emission intensity of the acceptor due to the excitation of the acceptor at the λ_{max} of the donor, I_A = emission intensity of the acceptor upon excitation at the λ_{max} of acceptor, and $I_{A,cor}$ = correction for the tail emission intensity of the donor.

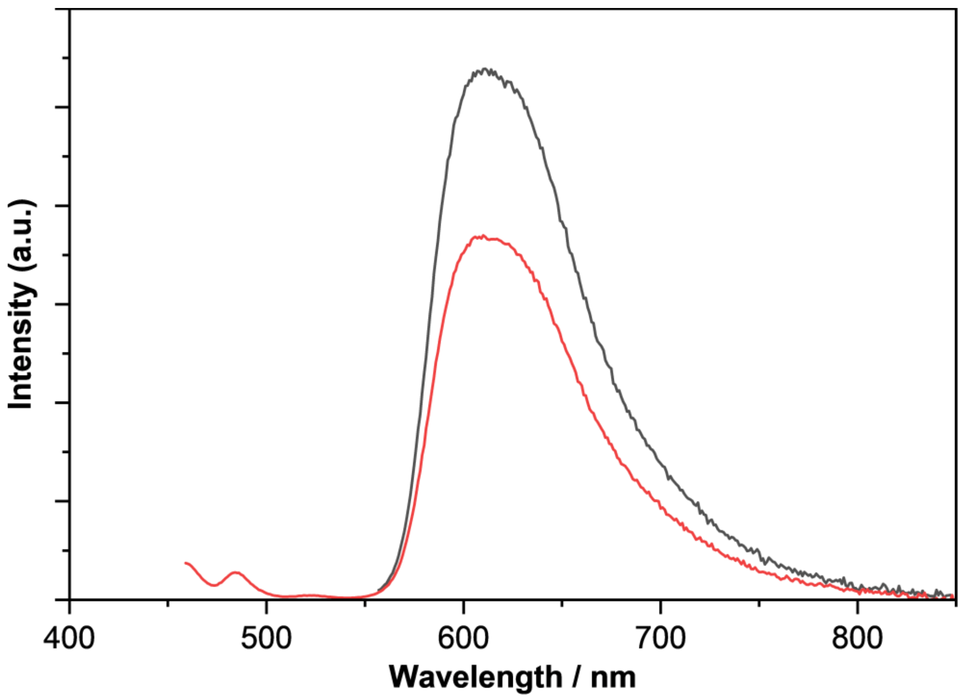

Figure S142. Emission spectra upon excitation at the λ_{max} of donor **8** (red, 449 nm) and acceptor **1** (black, 547 nm) in **8^{1000}/1^{1000}@5CB** at RT.

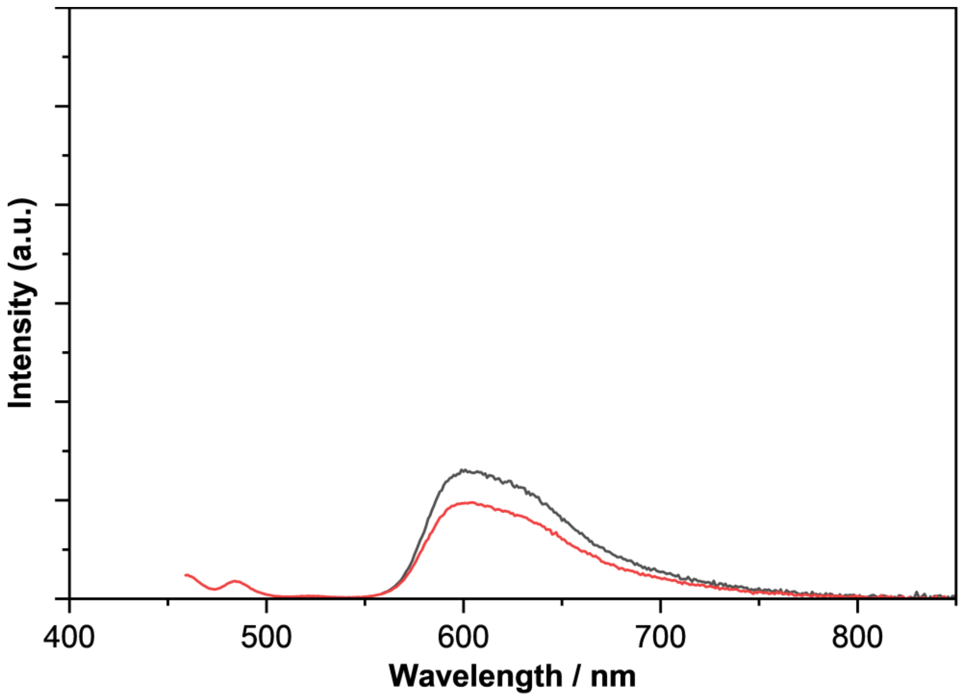

Figure S143. Emission spectra upon excitation at the λ_{max} of donor **8** (red, 449 nm) and acceptor **1** (black, 547 nm) in **8^{1000}/1^{500}@5CB** at RT.

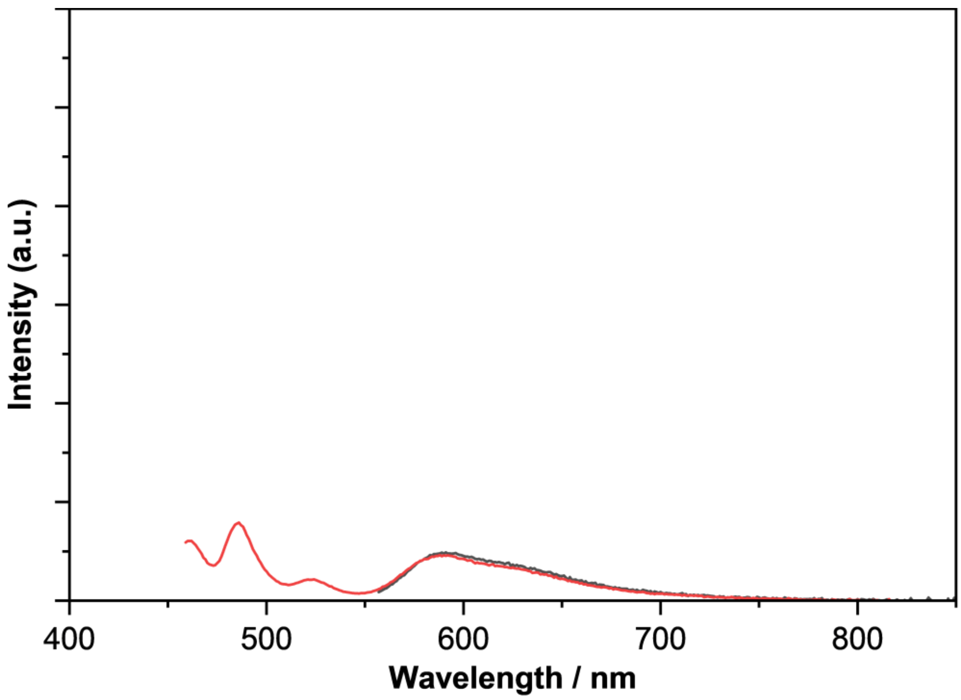

Figure S144. Emission spectra upon excitation at the λ_{max} of donor **8** (red, 449 nm) and acceptor **1** (black, 547 nm) in **8^{1000}/1^{200}@5CB** at RT.

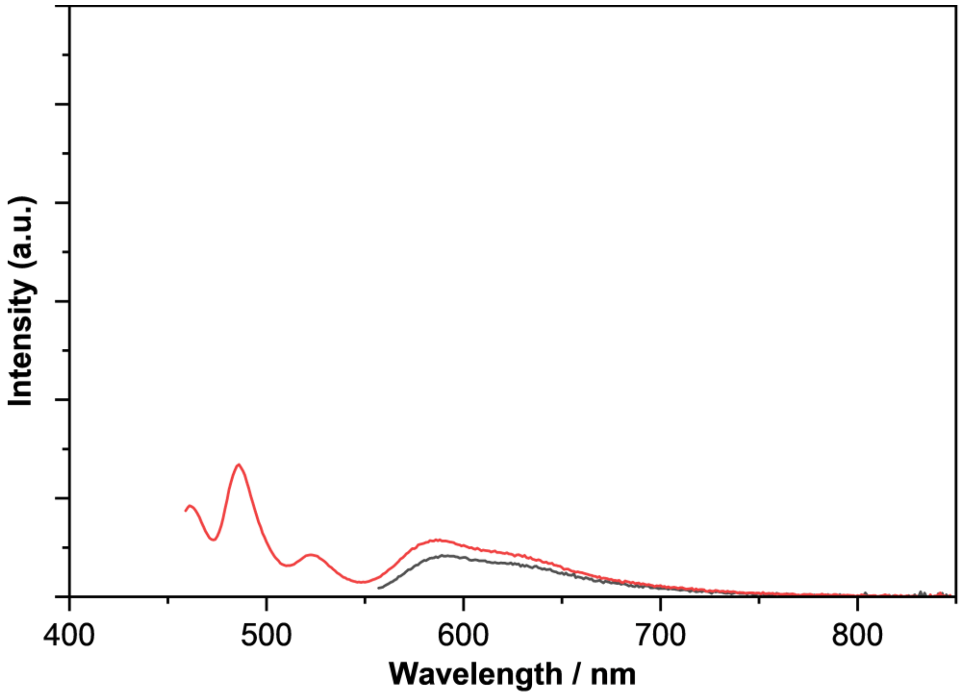

Figure S145. Emission spectra upon excitation at the λ_{max} of donor **8** (red, 449 nm) and acceptor **1** (black, 547 nm) in **8^{1000}/1^{100}@5CB** at RT.

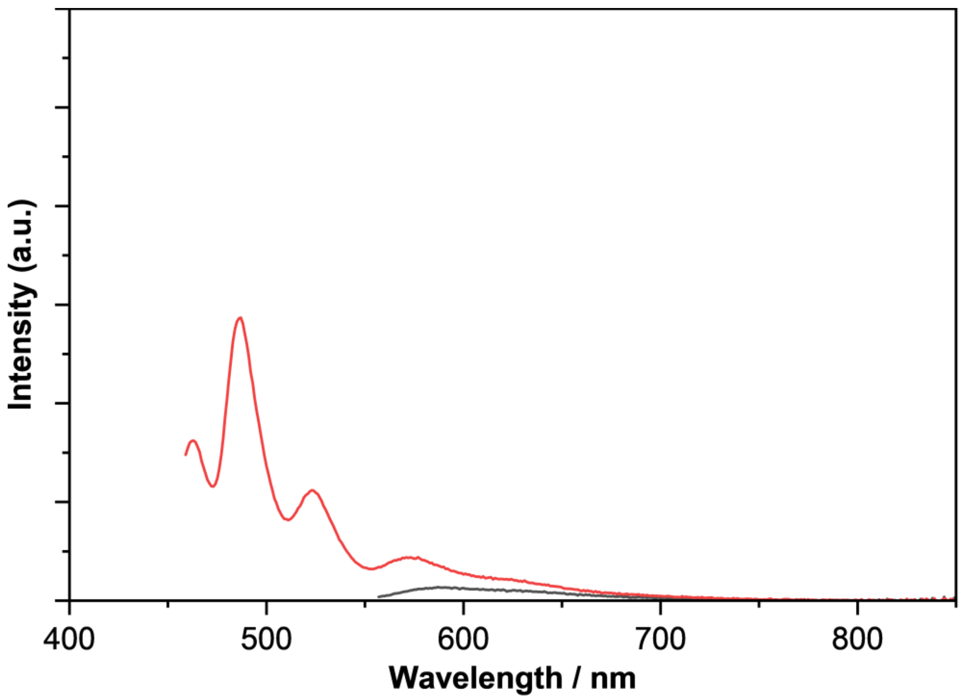

Figure S146. Emission spectra upon excitation at the λ_{max} of donor **8** (red, 449 nm) and acceptor **1** (black, 547 nm) in $\mathbf{8^{1000}/1^{20}@5CB}$ at RT.

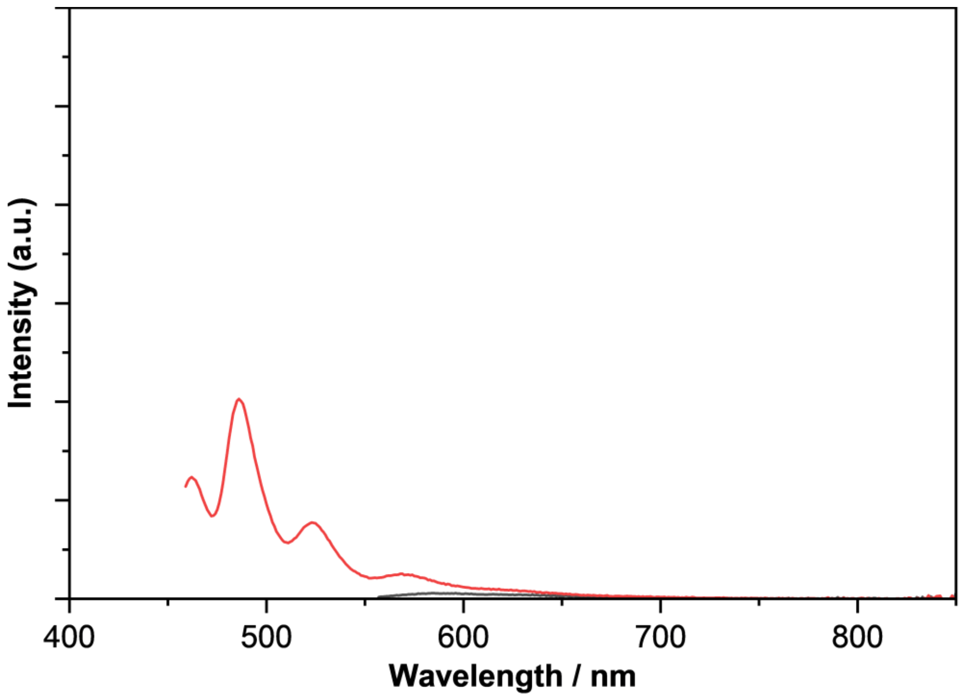

Figure S147. Emission spectra upon excitation at the λ_{max} of donor **8** (red, 449 nm) and acceptor **1** (black, 547 nm) in $\mathbf{8^{1000}/1^{10}@5CB}$ at RT.

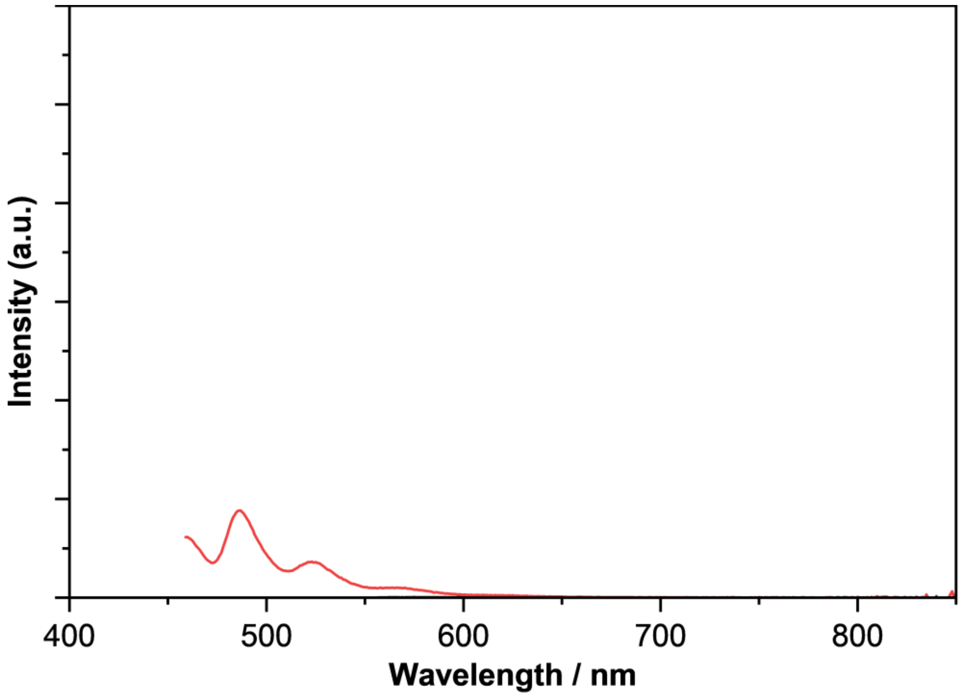

Figure S148. Emission spectra upon excitation at the λ_{max} of donor **8** (red, 449 nm) and acceptor **1** (black, 547 nm) in $\mathbf{8^{1000}/1^{5}@5CB}$ at RT.

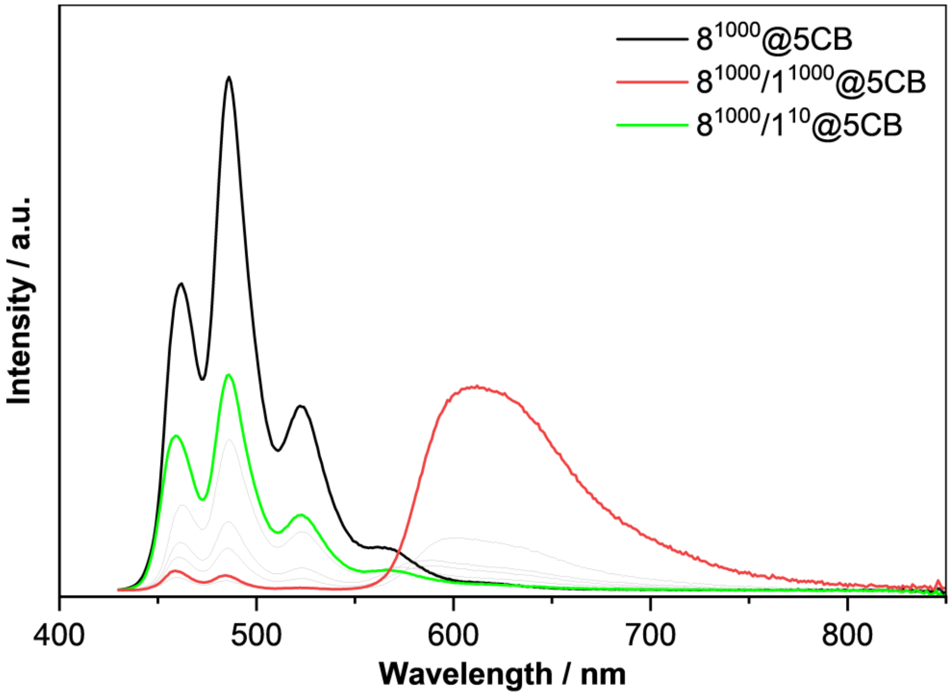

Figure S149. Emission spectra upon excitation of donor **8** at 420 nm in $\mathbf{8^{1000}/1^{m}@5CB}$ at RT.

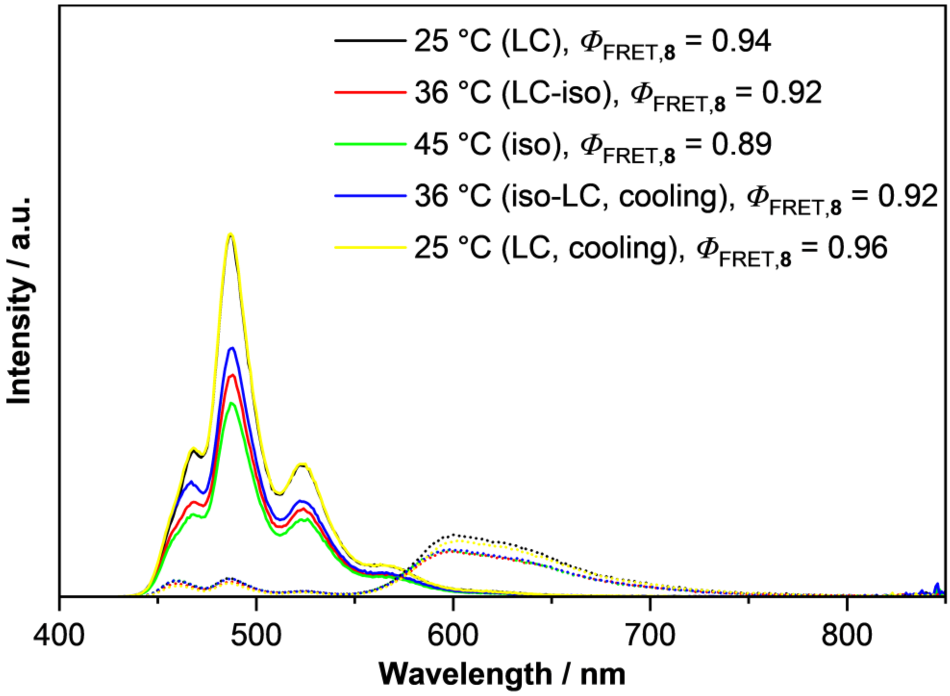

Figure S150. Emission spectra upon excitation of donor **8** at 420 nm in **$8^{1000}/1^{500}$@5CB** (after 1 year) at various temperatures.

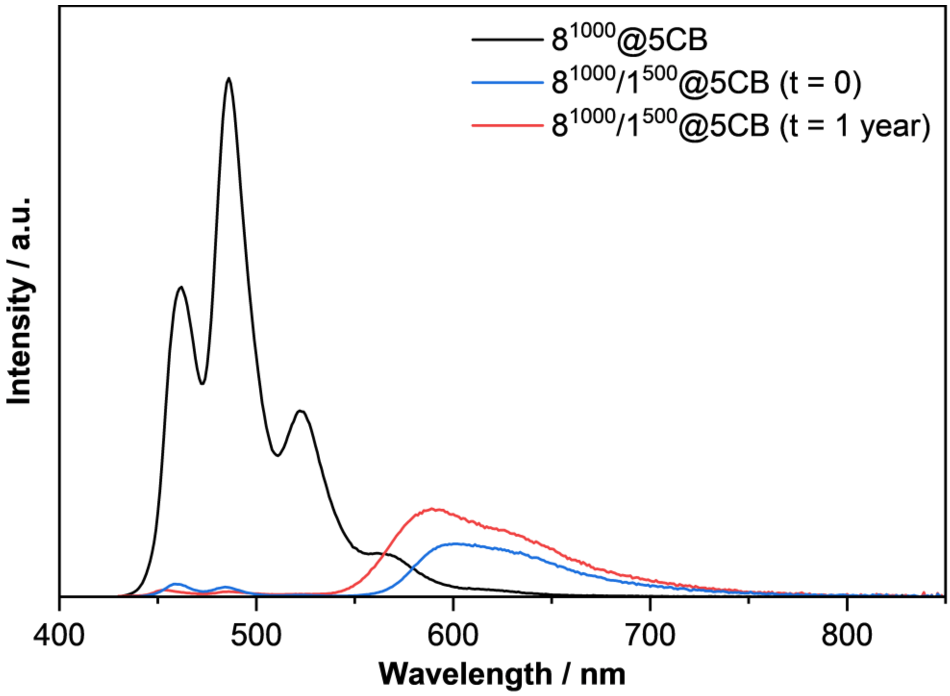

Figure S151. Emission spectra upon excitation of donor **8** at 420 nm in **$8^{1000}/1^{500}$@5CB** at RT.

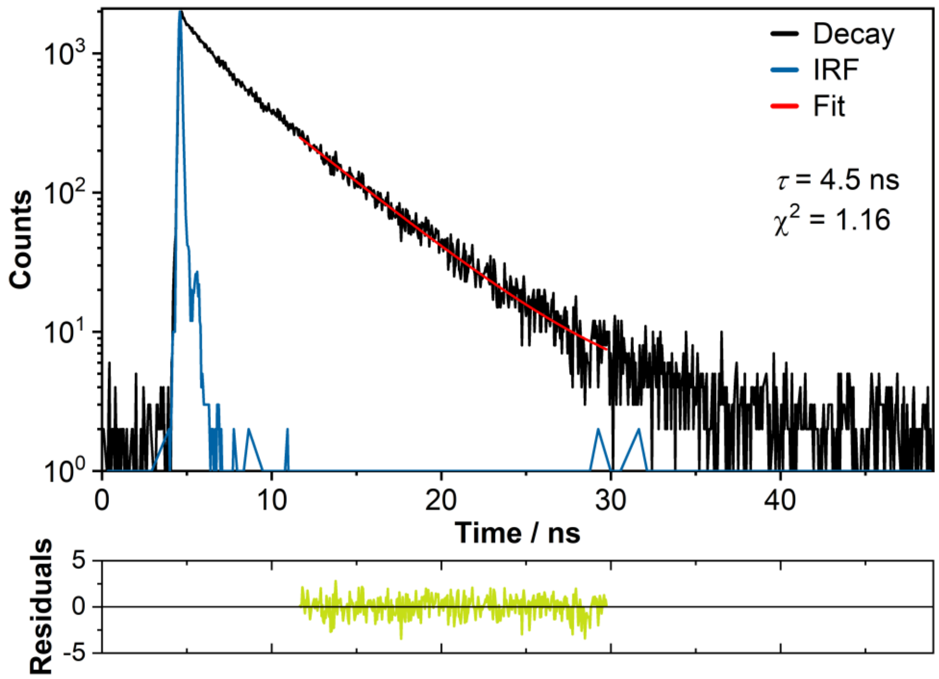

Figure S152. Time-resolved emission decay (λ_{ex} = 374.2 nm, λ_{em} = 459 nm) of donor **8** in $\mathbf{8^{1000}/1^{1000}@5CB}$ at RT.

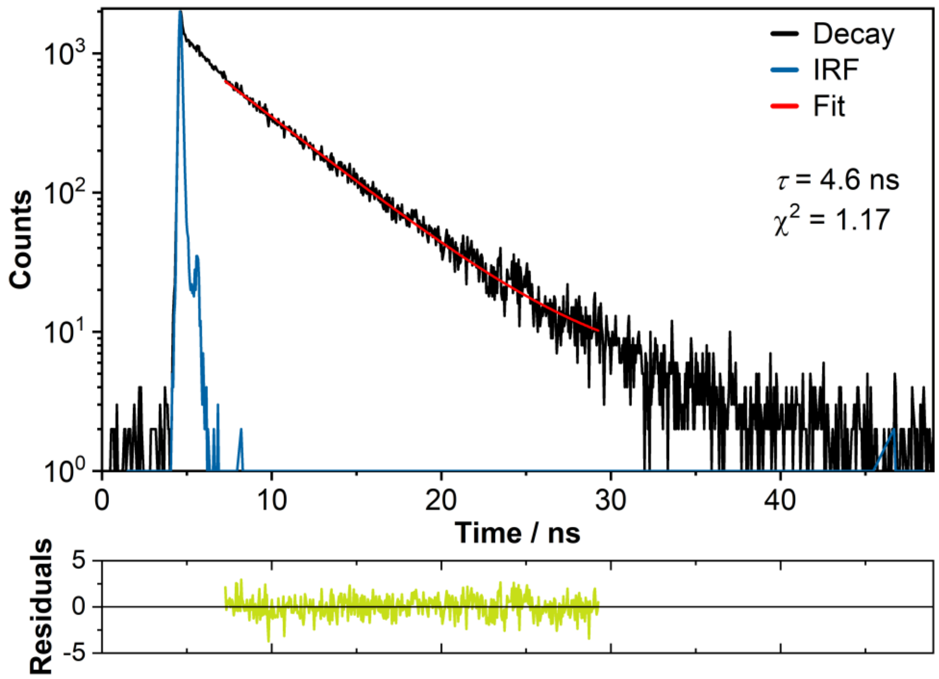

Figure S153. Time-resolved emission decay (λ_{ex} = 374.2 nm, λ_{em} = 459 nm) of donor **8** in $\mathbf{8^{1000}/1^{500}@5CB}$ at RT.

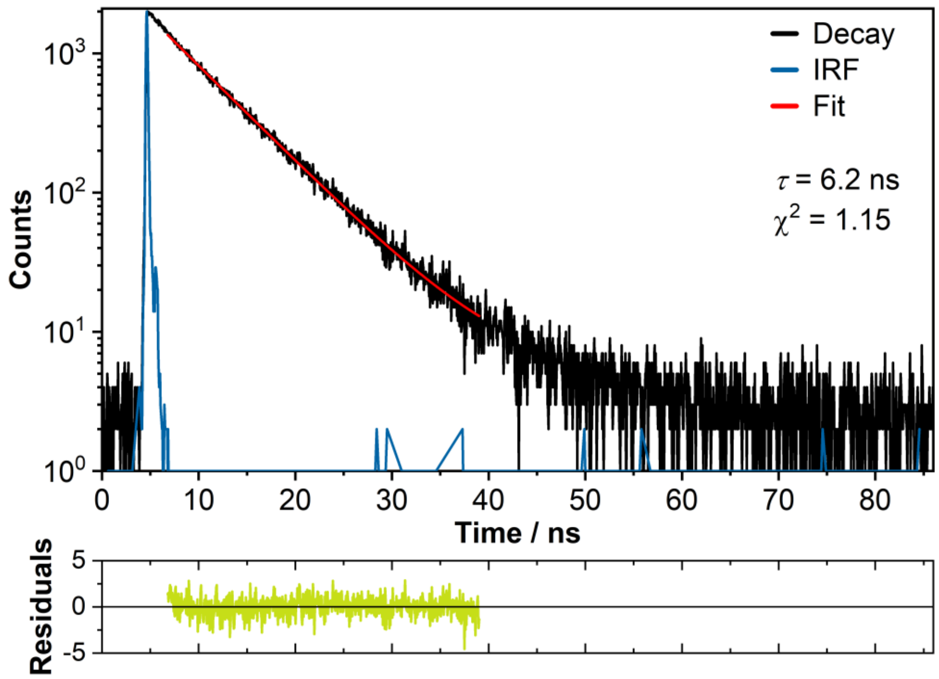

Figure S154. Time-resolved emission decay (λ_{ex} = 374.2 nm, λ_{em} = 459 nm) of donor **8** in **8^{1000}/1^{200}@5CB** at RT.

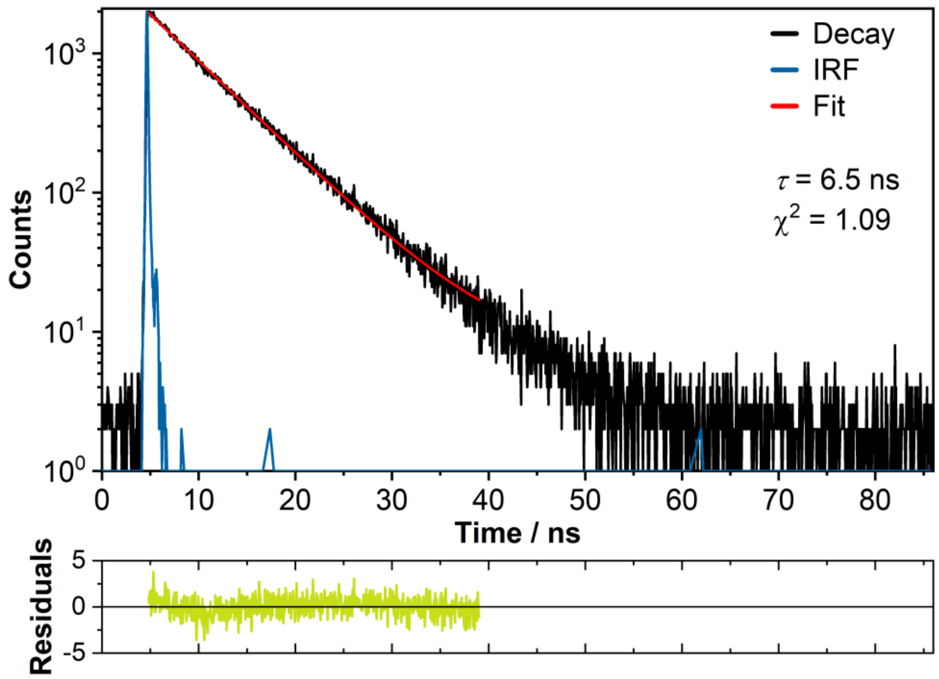

Figure S155. Time-resolved emission decay (λ_{ex} = 374.2 nm, λ_{em} = 459 nm) of donor **8** in **8^{1000}/1^{100}@5CB** at RT.

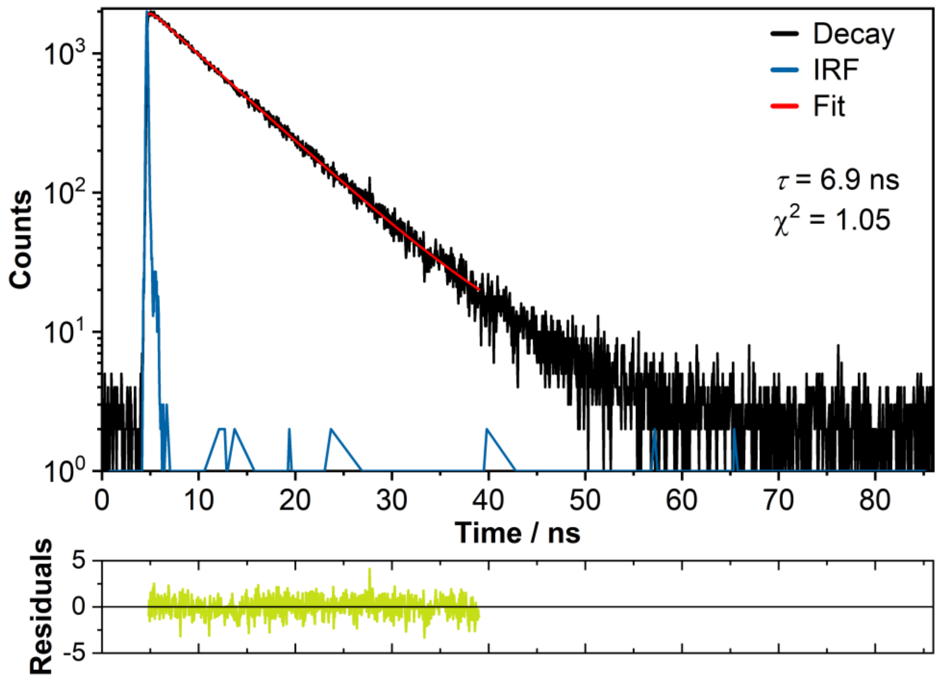

Figure S156. Time-resolved emission decay (λ_{ex} = 374.2 nm, λ_{em} = 459 nm) of donor **8** in **8^{1000}/1^{20}@5CB** at RT.

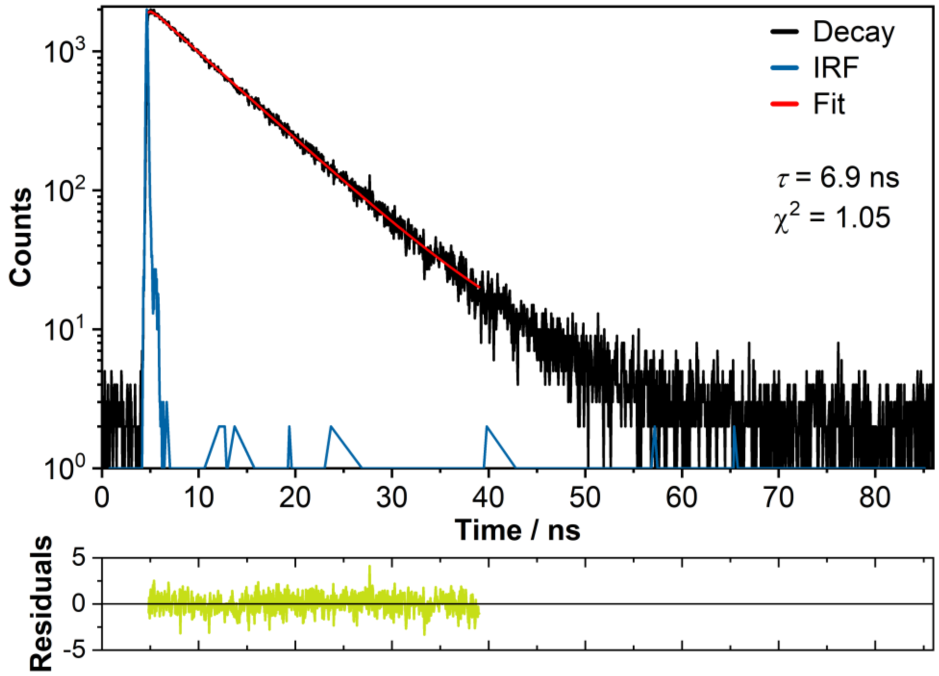

Figure S157. Time-resolved emission decay (λ_{ex} = 374.2 nm, λ_{em} = 459 nm) of donor **8** in **8^{1000}/1^{10}@5CB** at RT.

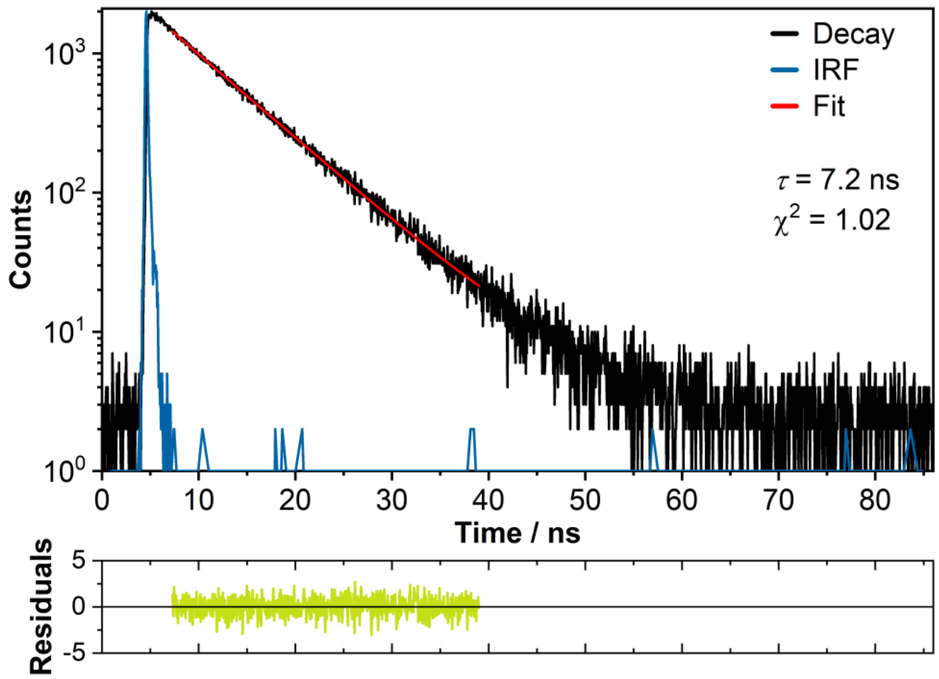

Figure S158. Time-resolved emission decay (λ_{ex} = 374.2 nm, λ_{em} = 459 nm) of donor **8** at in **8^{1000}/1^{5}@5CB** at RT.

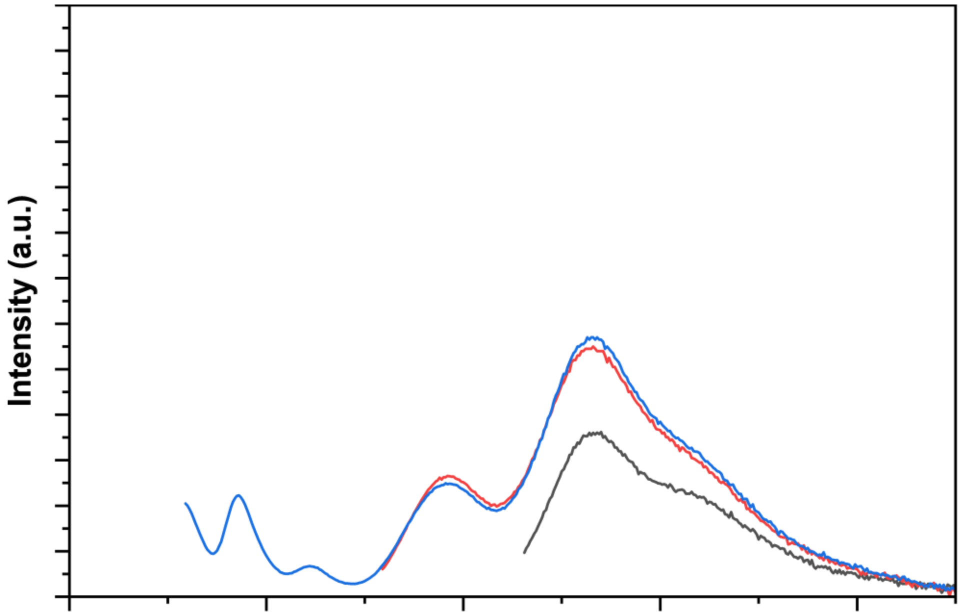

Figure S159. Emission spectra upon excitation at the λ_{max} of primary donor **8** (blue, 449 nm), relay acceptor **1** (red, 547 nm) and final acceptor **4** (black, 621 nm) in **8^{1000}/1^{500}/4^{250}@5CB** at RT.

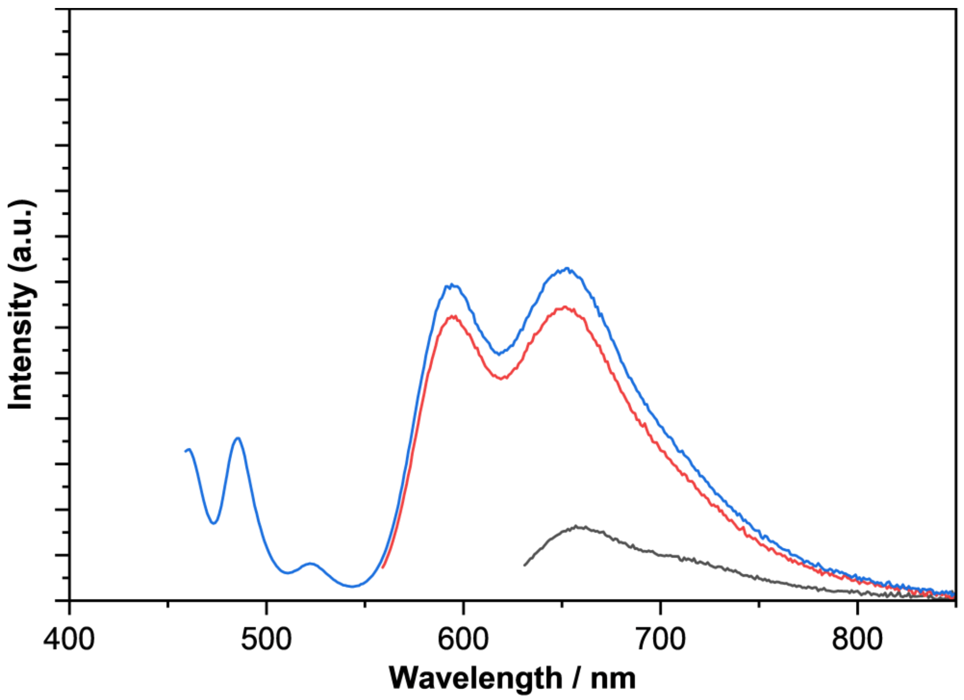

Figure S160. Emission spectra upon excitation at the λ_{max} of primary donor **8** (blue, 449 nm), relay acceptor **1** (red, 547 nm) and final acceptor **4** (black, 621 nm) in $\mathbf{8^{1000}/1^{500}/4^{50}}$**@5CB** at RT.

.

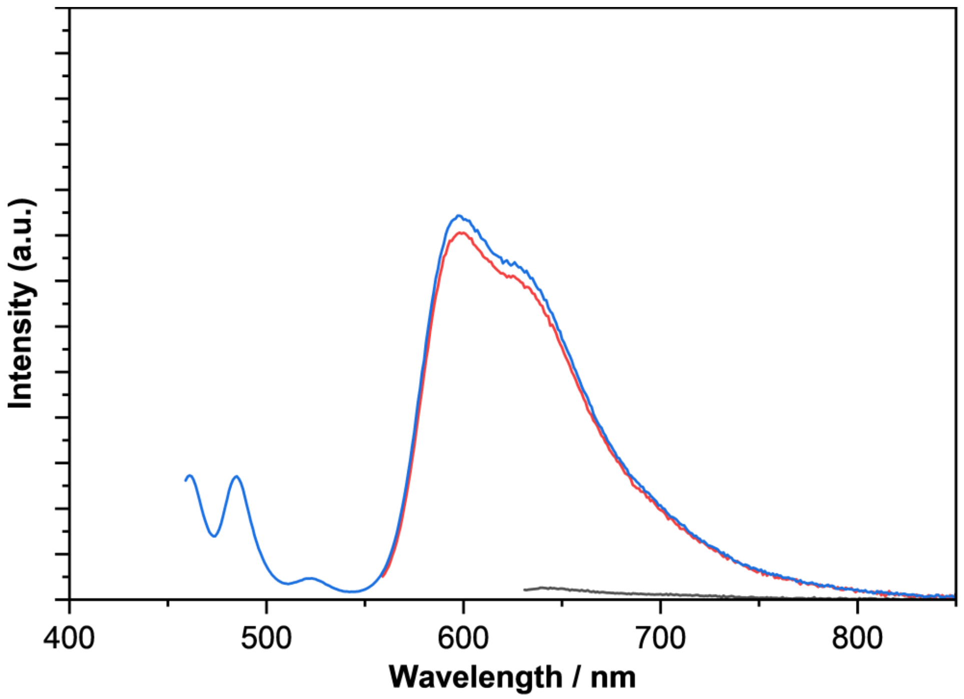

Figure S161. Emission spectra upon excitation at the λ_{max} of primary donor **8** (blue, 449 nm), relay acceptor **1** (red, 547 nm) and final acceptor **4** (black, 621 nm) in $\mathbf{8^{1000}/1^{500}/4^{5}}$**@5CB** at RT.

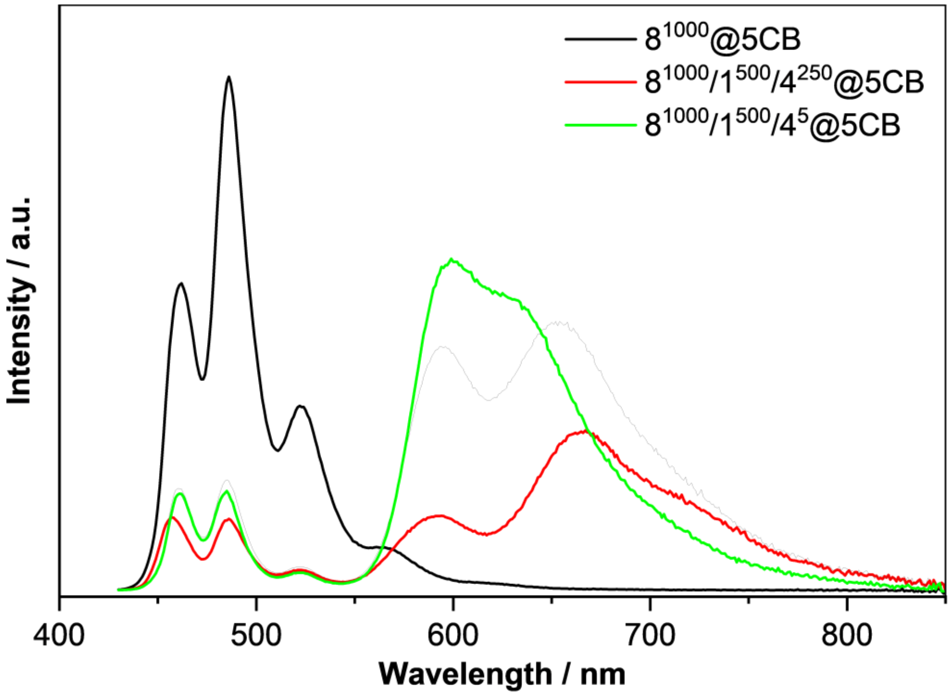

Figure S162. Emission spectra upon excitation of donor **8** at 420 nm in **$8^{1000}/1^{n}/4^{p}$@5CB** at RT.

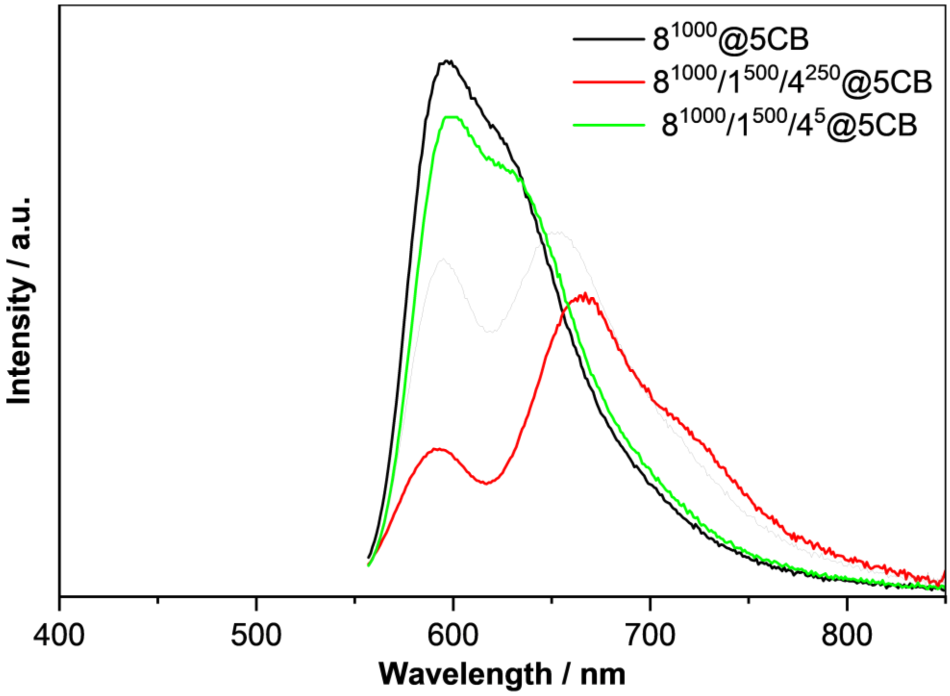

Figure S163. Emission spectra upon excitation of relay acceptor **1** at 547 nm in **$8^{1000}/1^{n}/4^{p}$@5CB** at RT.

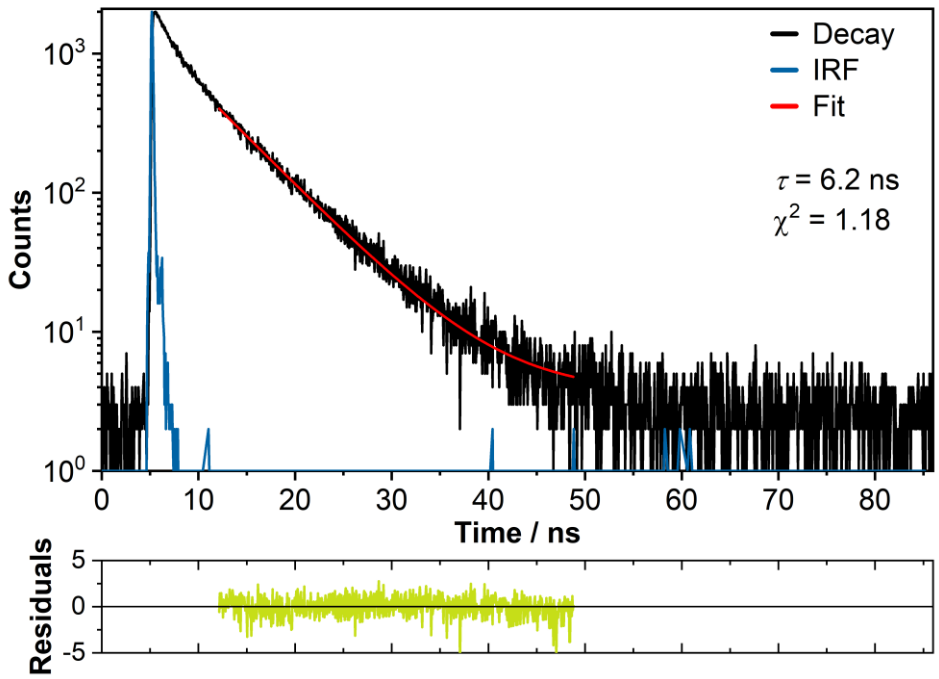

Figure S164. Time-resolved emission decay (λ_{ex} = 505 nm, λ_{em} = 575 nm) of relay acceptor **1** in $\mathbf{8^{1000}/1^{500}/4^{250}}$**@5CB** at RT.

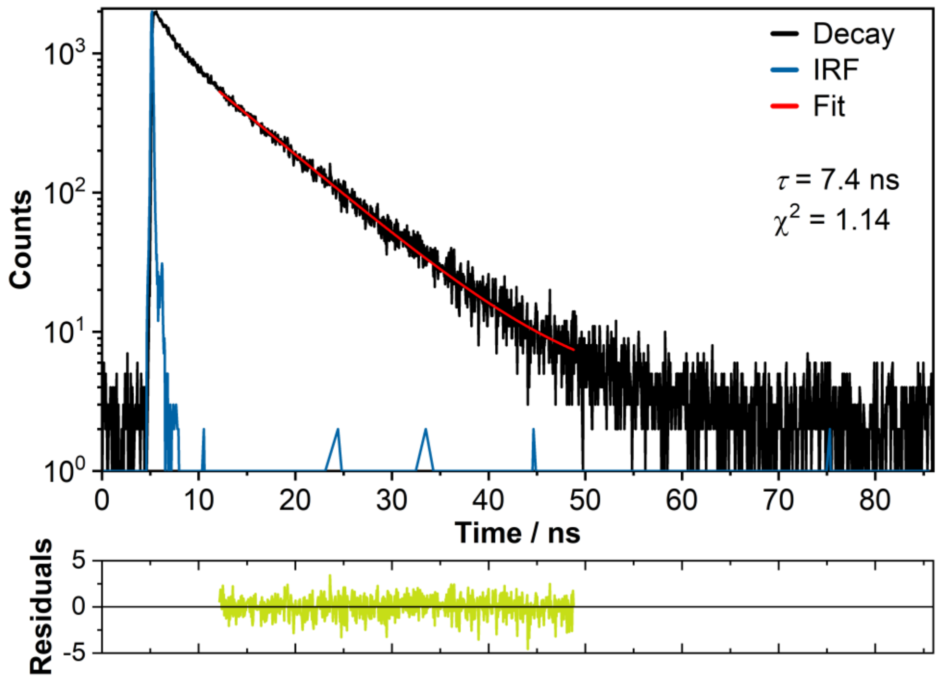

Figure S165. Time-resolved emission decay (λ_{ex} = 505 nm, λ_{em} = 575 nm) of relay acceptor **1** in $\mathbf{8^{1000}/1^{500}/4^{50}}$**@5CB** at RT.

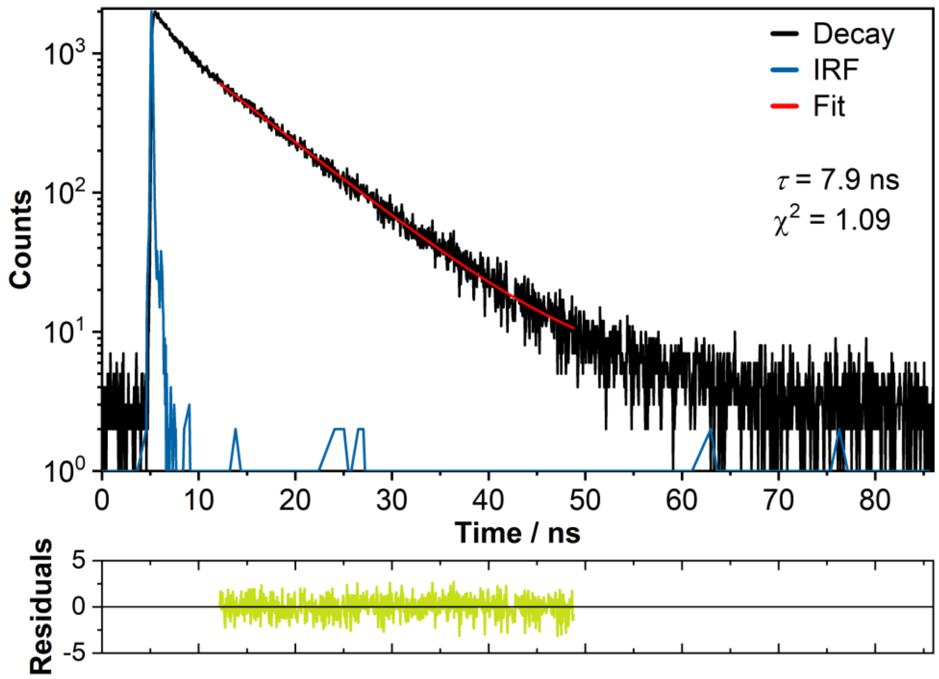

Figure S166. Time-resolved emission decay (λ_{ex} = 505 nm, λ_{em} = 575 nm) of relay acceptor **1** in **8^{1000}/1^{500}/4^{5}@5CB** at RT.

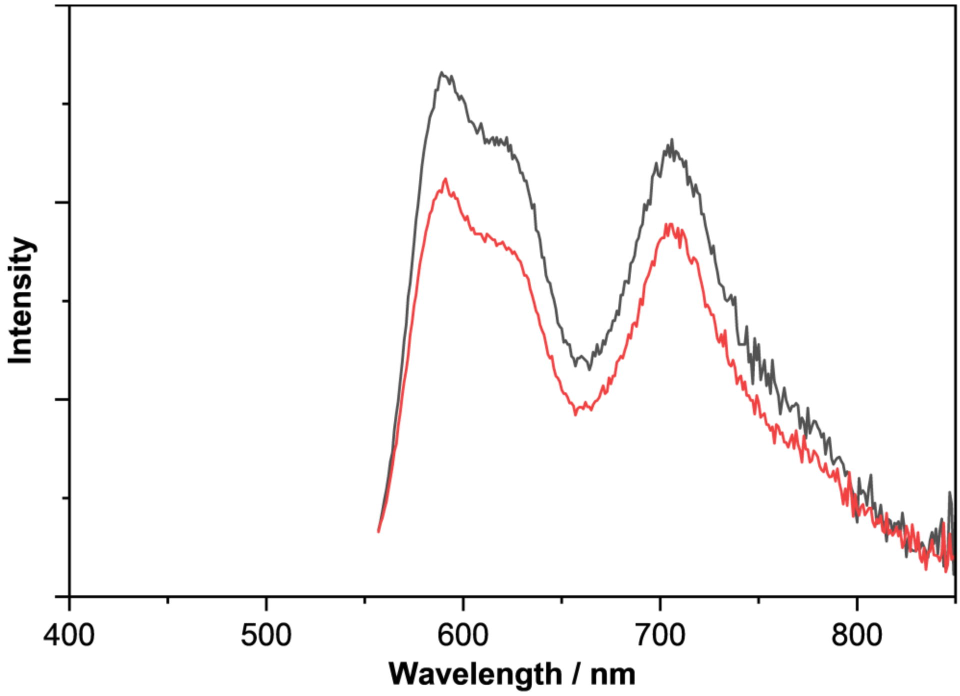

Figure S167. Emission spectra upon excitation at the λ_{max} of primary donor **8** (red, 449 nm) and relay acceptor **1** (black, 547 nm) in **8^{1000}/1^{500}/6^{250}@5CB** at RT.

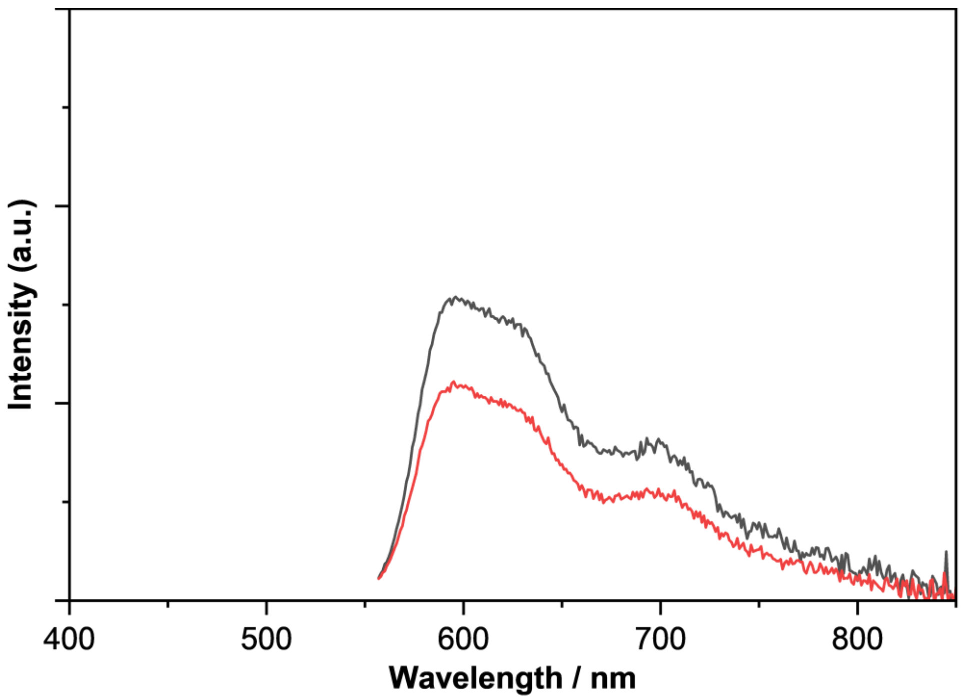

Figure S168. Emission spectra upon excitation at the λ_{max} of primary donor **8** (red, 449 nm) and relay acceptor **1** (black, 547 nm) in $\mathbf{8^{1000}/1^{500}/6^{50}@5CB}$ at RT.

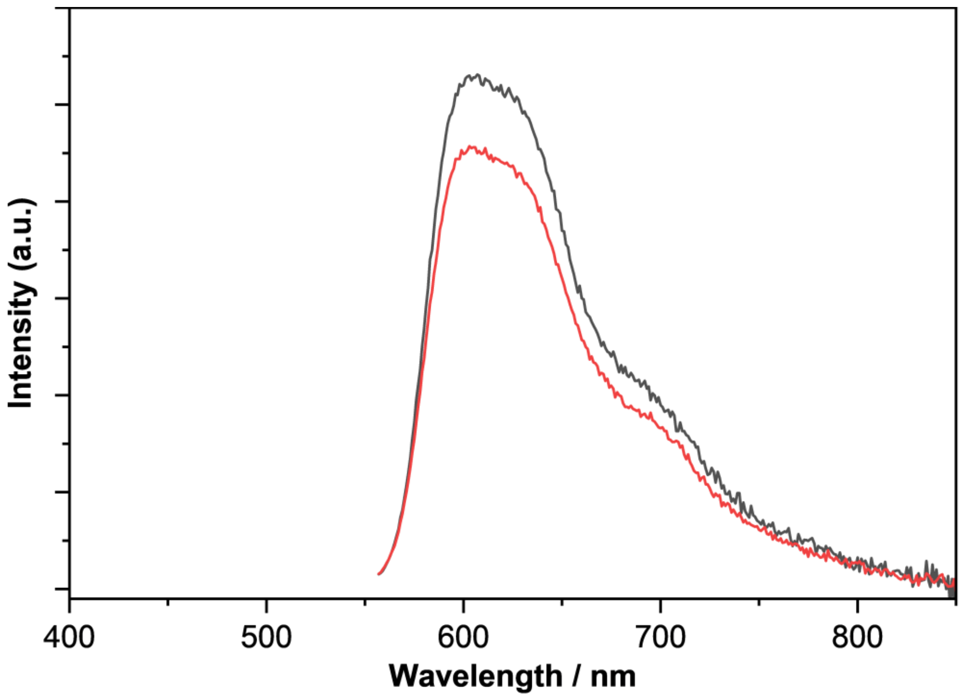

Figure S169. Emission spectra upon excitation at the λ_{max} of primary donor **8** (red, 449 nm) and relay acceptor **1** (black, 547 nm) in $\mathbf{8^{1000}/1^{500}/6^{5}@5CB}$ at RT.

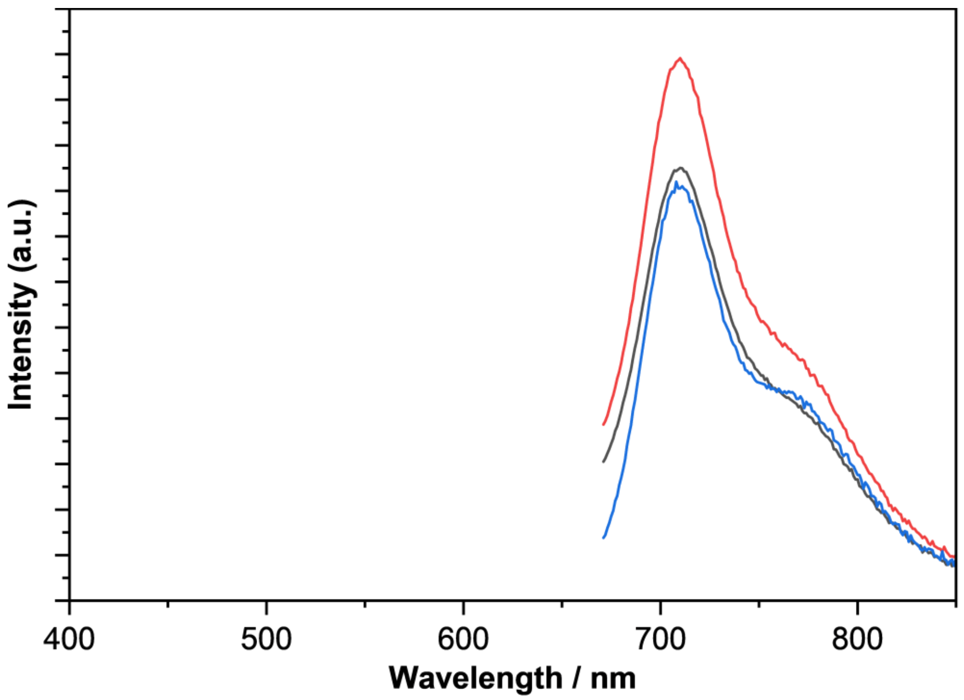

Figure S170. Emission spectra upon excitation at the λ_{max} of primary donor **8** (black, 449 nm), relay acceptor **1** (red, 547 nm) and final acceptor **6** (blue, 661 nm) in $\mathbf{8^{1000}/1^{500}/6^{250}@5CB}$ at RT.

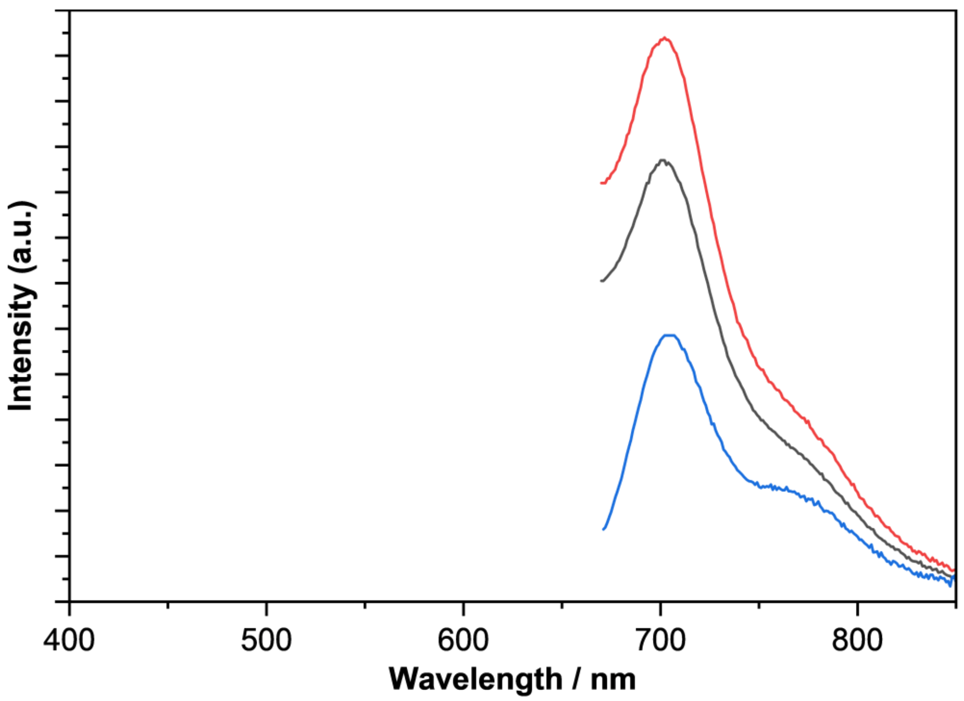

Figure S171. Emission spectra upon excitation at the λ_{max} of primary donor **8** (black, 449 nm), relay acceptor **1** (red, 547 nm) and final acceptor **6** (blue, 661 nm) in $\mathbf{8^{1000}/1^{500}/6^{50}@5CB}$ at RT.

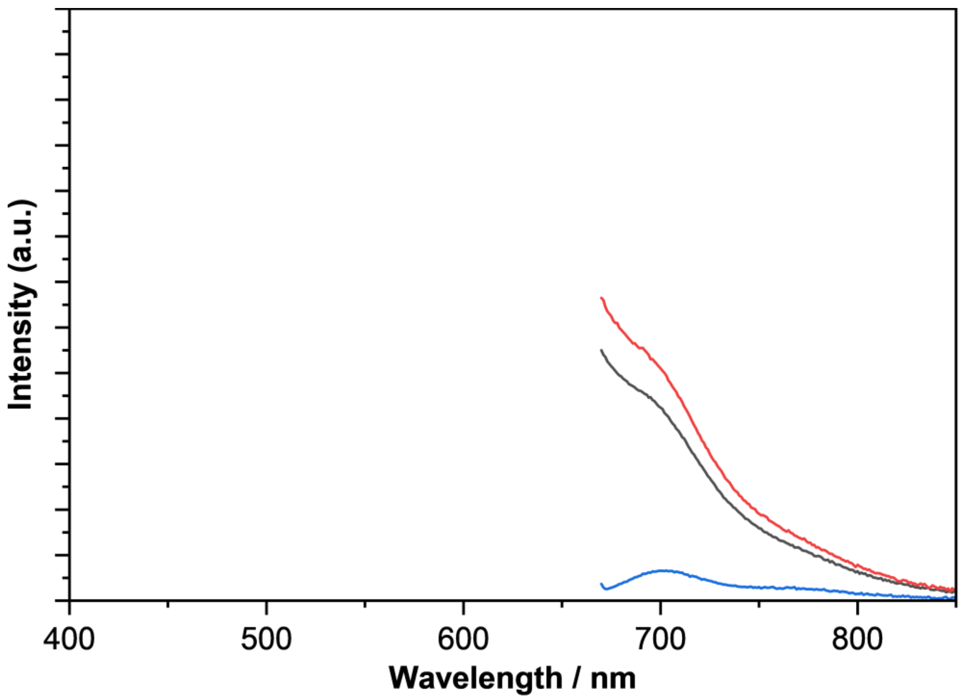

Figure S172. Emission spectra upon excitation at the λ_{max} of primary donor **8** (black, 449 nm), relay acceptor **1** (red, 547 nm) and final acceptor **6** (blue, 661 nm) in **$8^{1000}/1^{500}/6^{5}$@5CB** at RT.

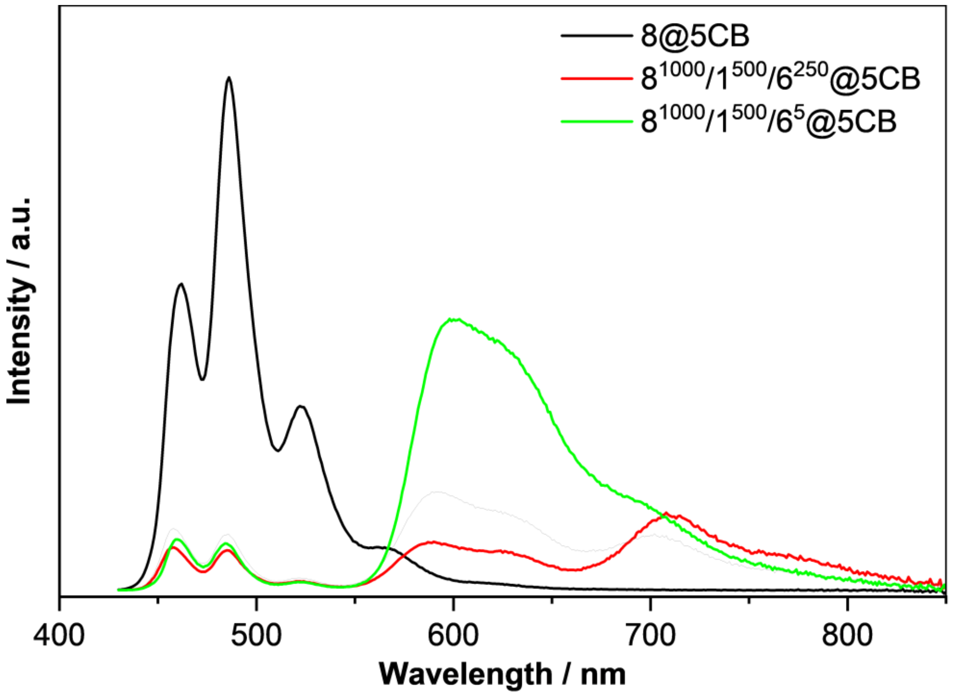

Figure S173. Emission spectra upon excitation of donor **8** at 420 nm in **$8^{1000}/1^{500}/6^{p}$@5CB** at RT.

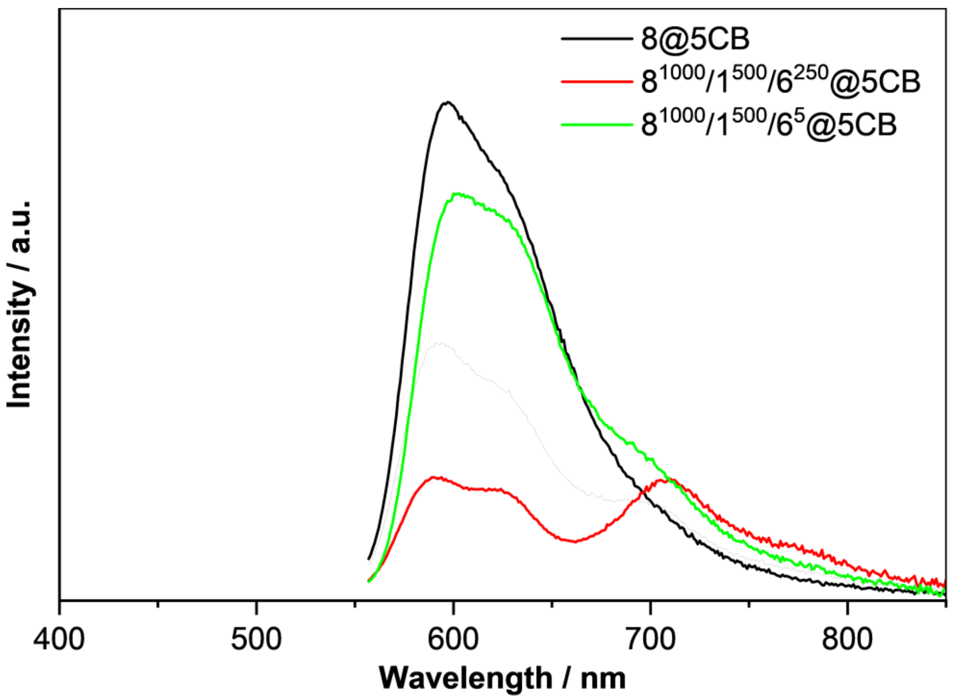

Figure S174. Emission spectra upon excitation of relay acceptor **1** at 547 nm in **8^{1000}/1^{500}/6^{p}@5CB** at RT.

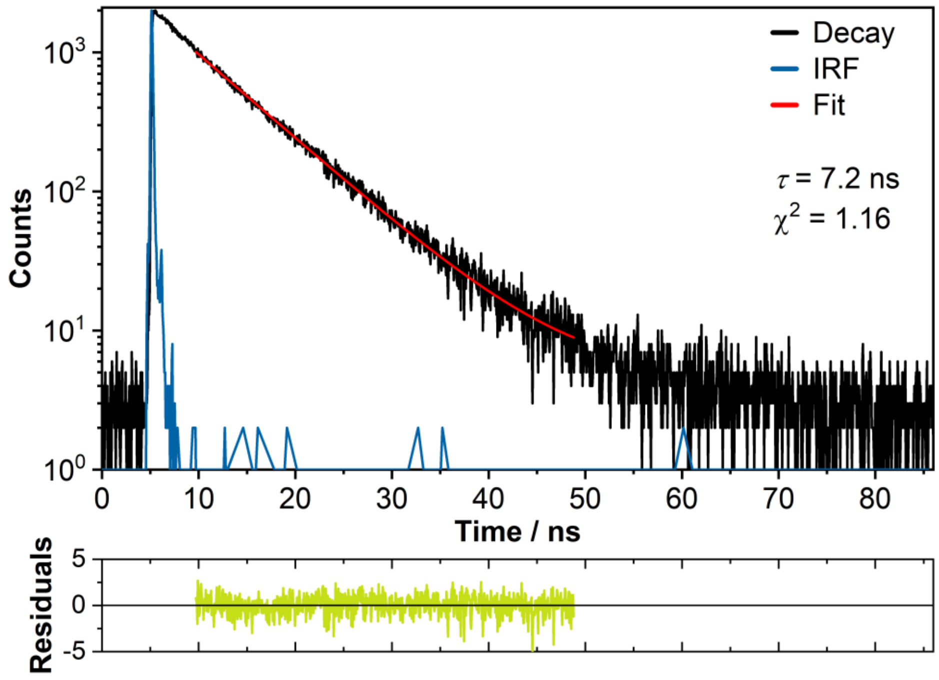

Figure S175. Time-resolved emission decay (λ_{ex} = 505 nm, λ_{em} = 575 nm) of relay acceptor **1** in **8^{1000}/1^{500}/6^{250}@5CB** at RT.

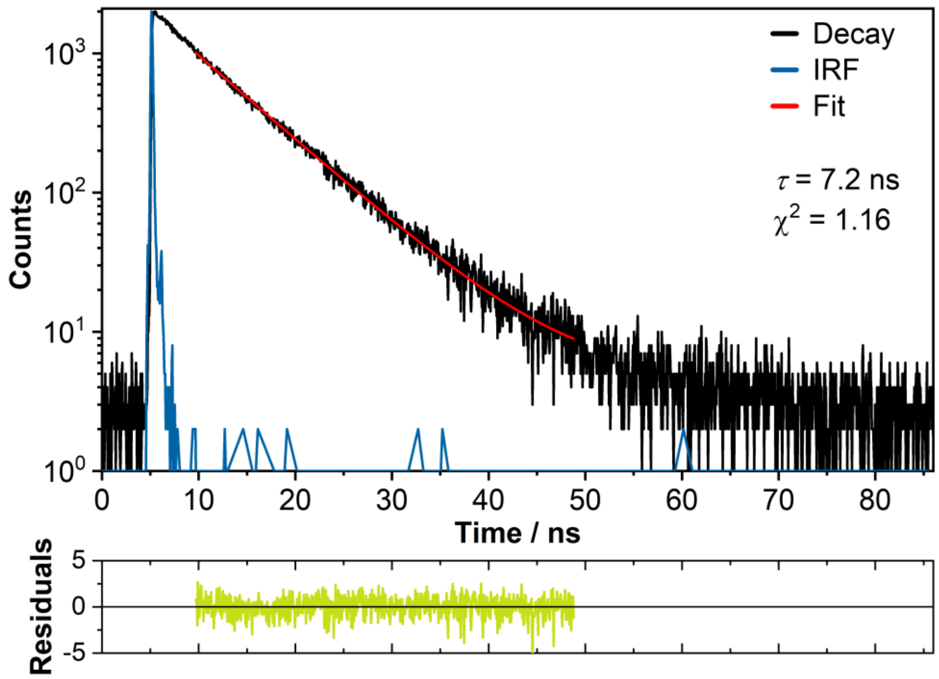

Figure S176. Time-resolved emission decay (λ_{ex} = 505 nm, λ_{em} = 575 nm) of relay acceptor **1** in $\mathbf{8^{1000}/1^{500}/6^{50}}$**@5CB** at RT.

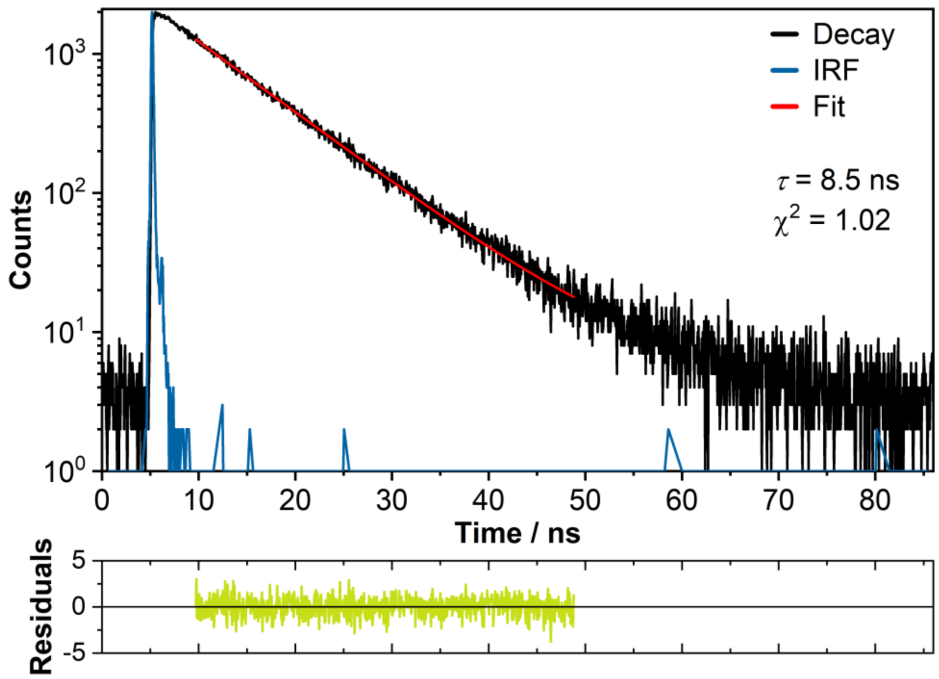

Figure S177. Time-resolved emission decay (λ_{ex} = 505 nm, λ_{em} = 575 nm) of relay acceptor **1** in $\mathbf{8^{1000}/1^{500}/6^{5}}$**@5CB** at RT.

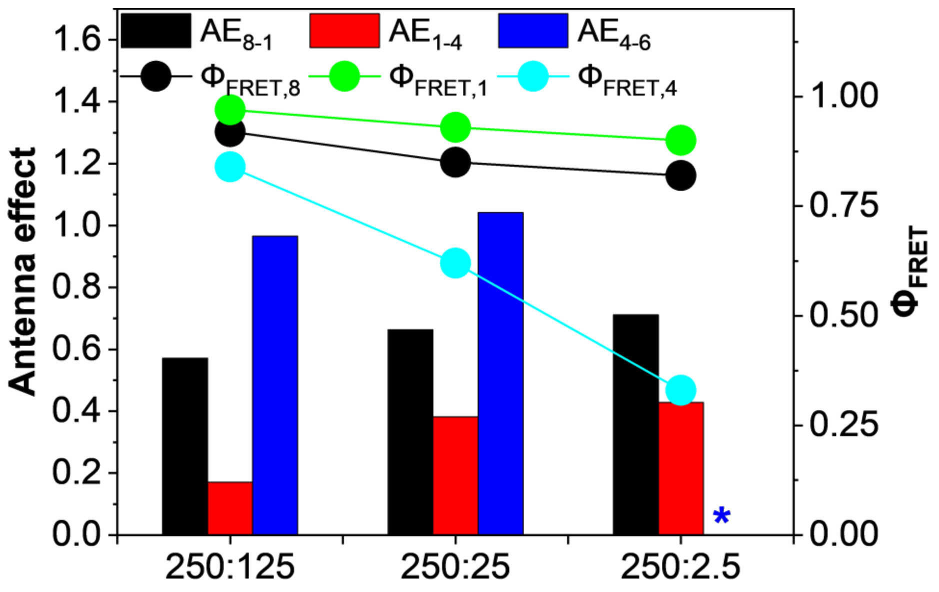

Figure S178. Φ_{FRET} and antenna effect data of **$8^{1000}/1^{500}/4^{250}/6^{q}$@5CB** at RT. *Cannot be determined.

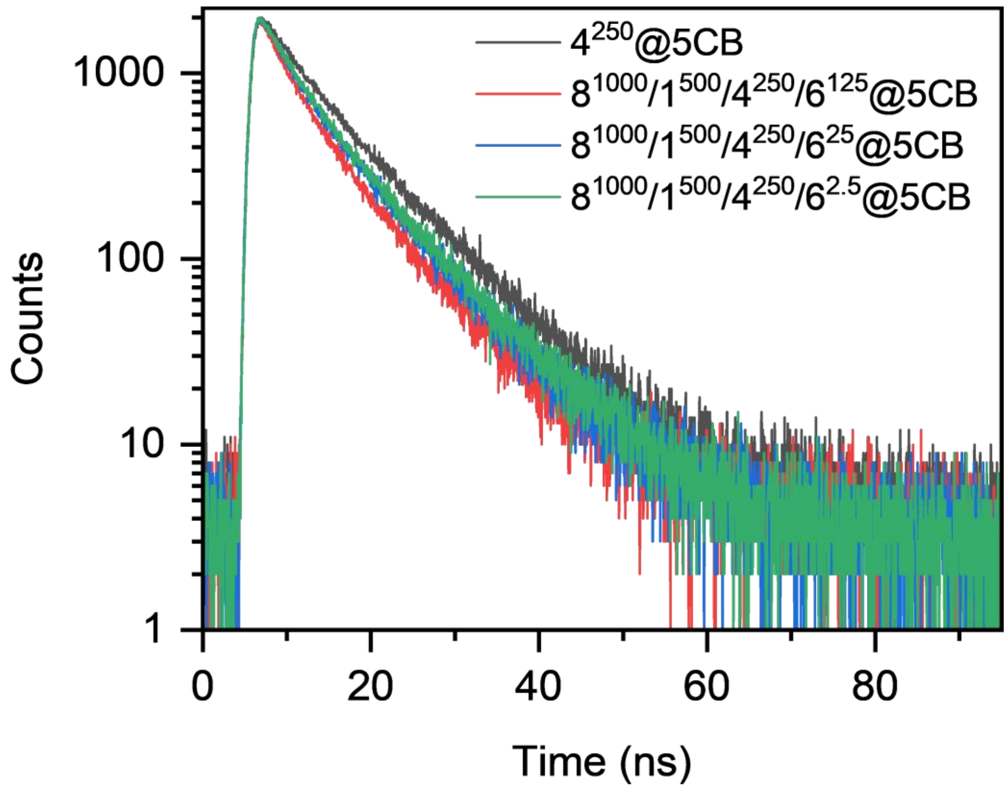

Figure S179. Time-resolved emission decay (λ_{ex} = 612.6 nm, λ_{em} = 650 nm) of relay acceptor **4** in **$8^{1000}/1^{500}/4^{250}/6^{q}$@5CB** at RT.

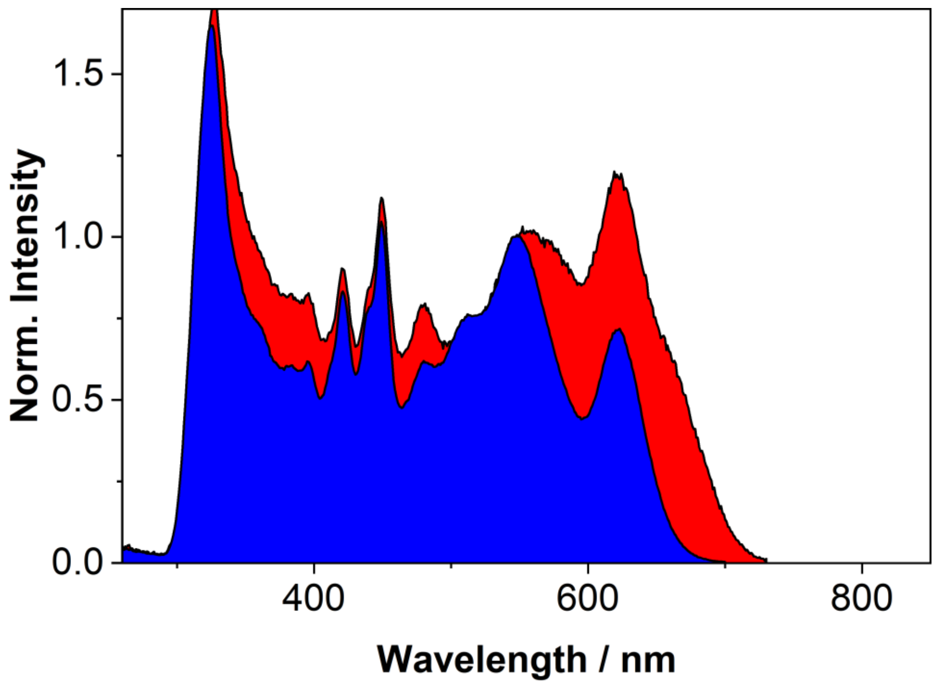

Figure S180. Normalized excitation spectra of $\mathbf{8^{1000}/1^{500}/4^{250}/6^{125}@5CB}$ (red, λ_{em} = 740 nm) and $\mathbf{8^{1000}/1^{500}/4^{250}@5CB}$ (blue, λ_{em} = 710 nm) at RT.

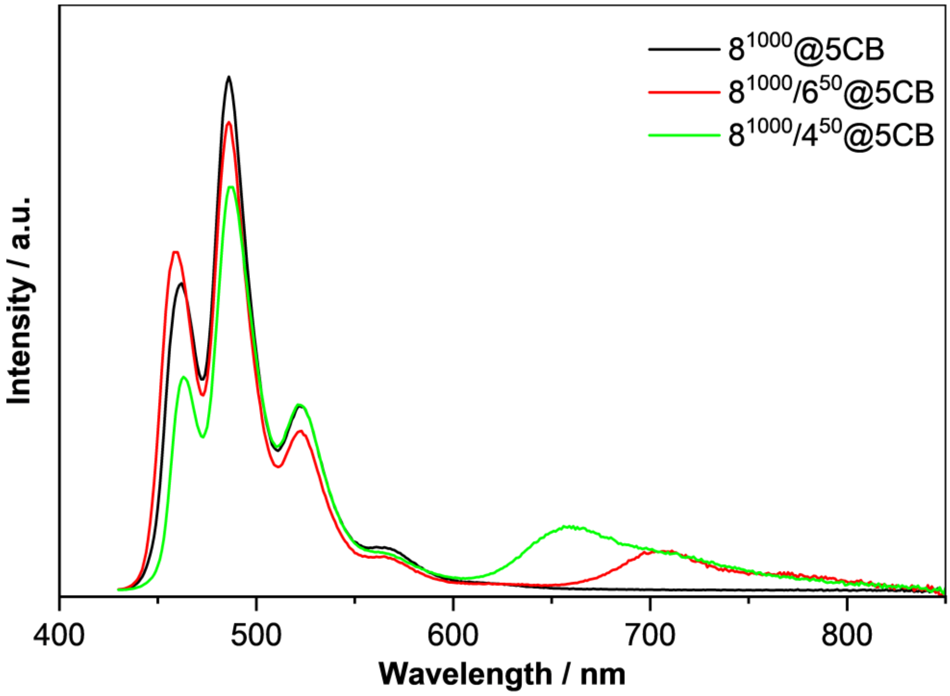

Figure S181. Emission spectra upon excitation of donor **8** at 420 nm of $\mathbf{8^{1000}/4^{50}@5CB}$ and $\mathbf{8^{1000}/6^{50}@5CB}$ at RT.

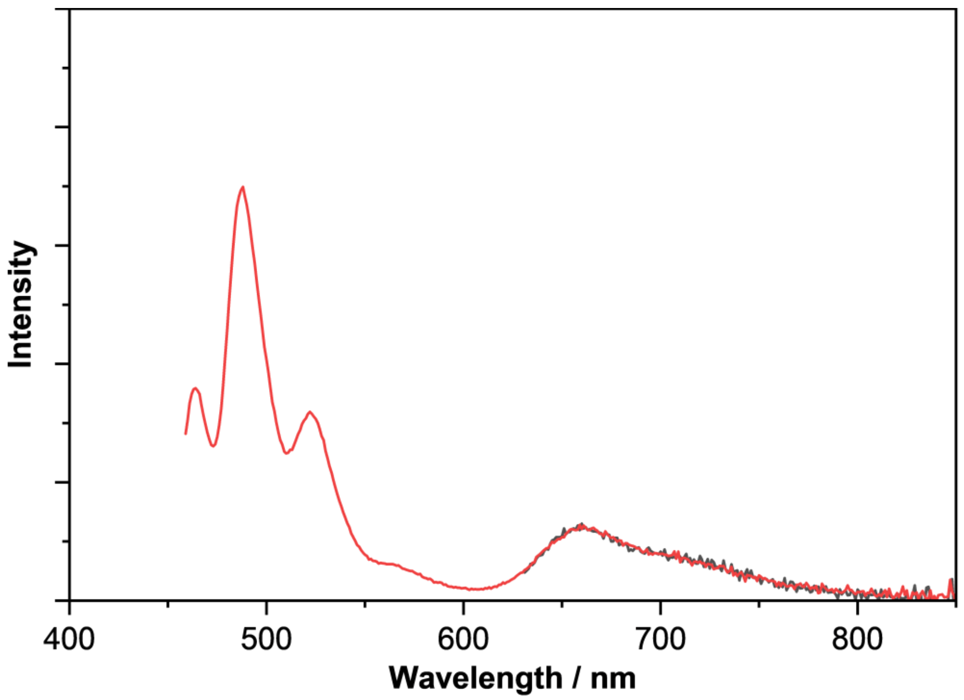

Figure S182. Emission spectra upon excitation at the λ_{max} of donor **8** (red, 449 nm) and final acceptor **4** (black, 621 nm) in $\mathbf{8^{1000}/4^{50}@5CB}$ at RT.

.

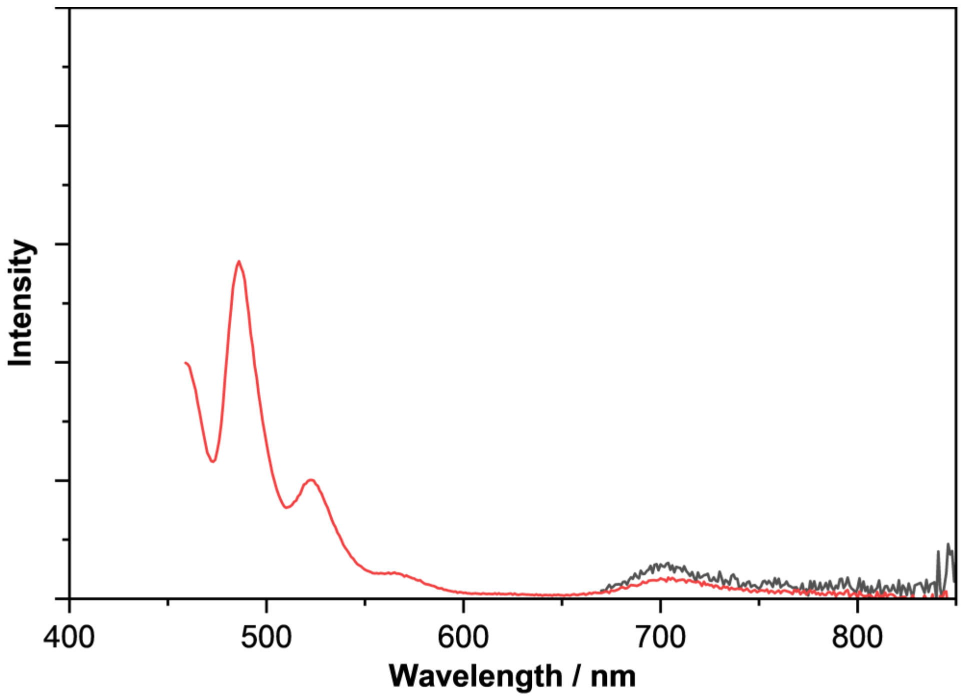

Figure S183. Emission spectra upon excitation at the λ_{max} of donor **8** (red, 449 nm) and final acceptor **6** (black, 661 nm) in $\mathbf{8^{1000}/6^{50}@5CB}$ at RT.

4.3 Structure and thermal properties of the light-harvesting systems

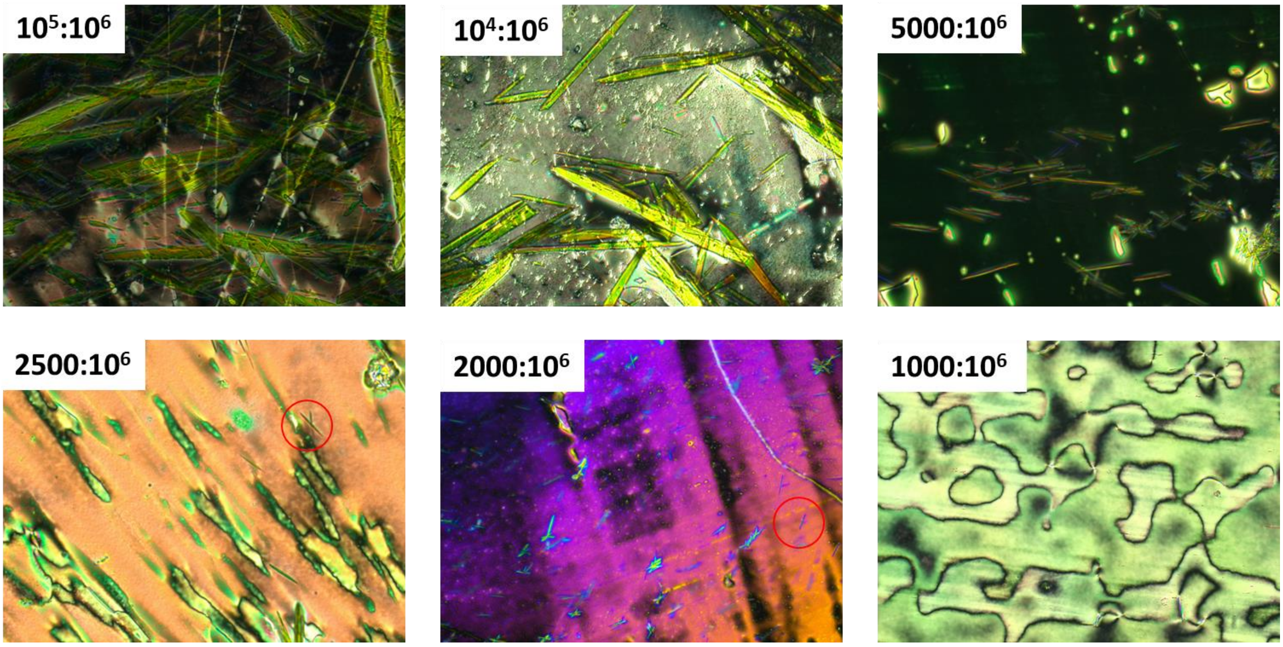

Figure S184. POM images of **5CB** doped with **8** at various **8**/**5CB** ratio at 20× magnification.

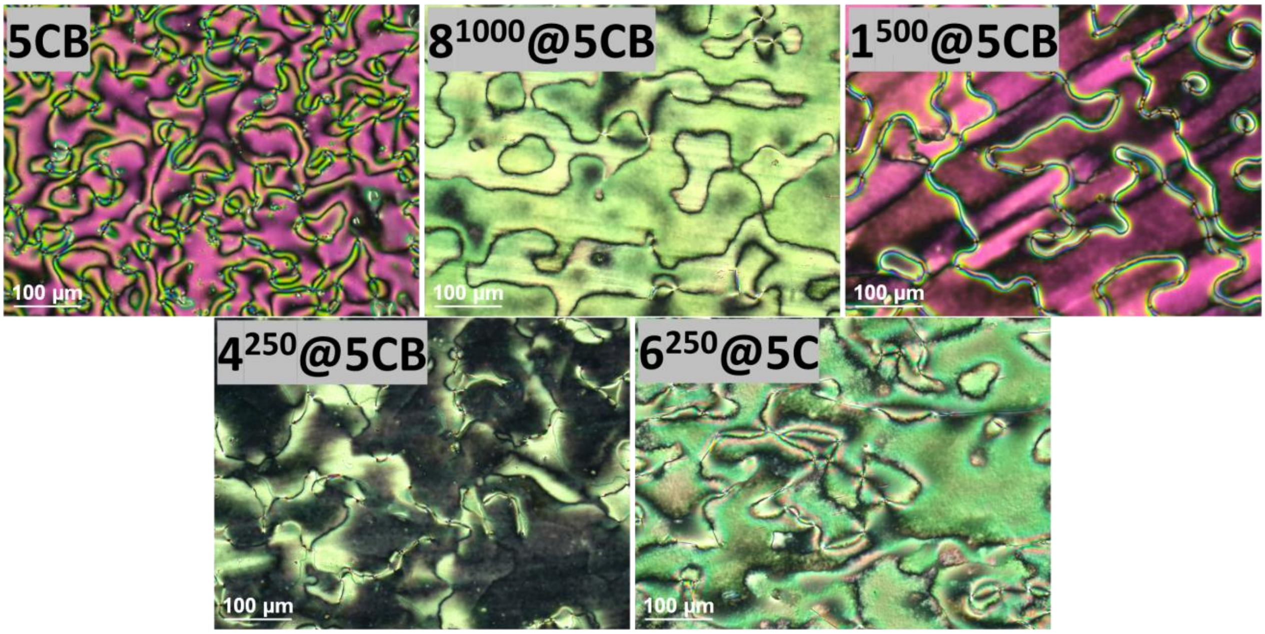

Figure S185. POM images of **5CB** and nanoribbons in **5CB** at 20× magnification.

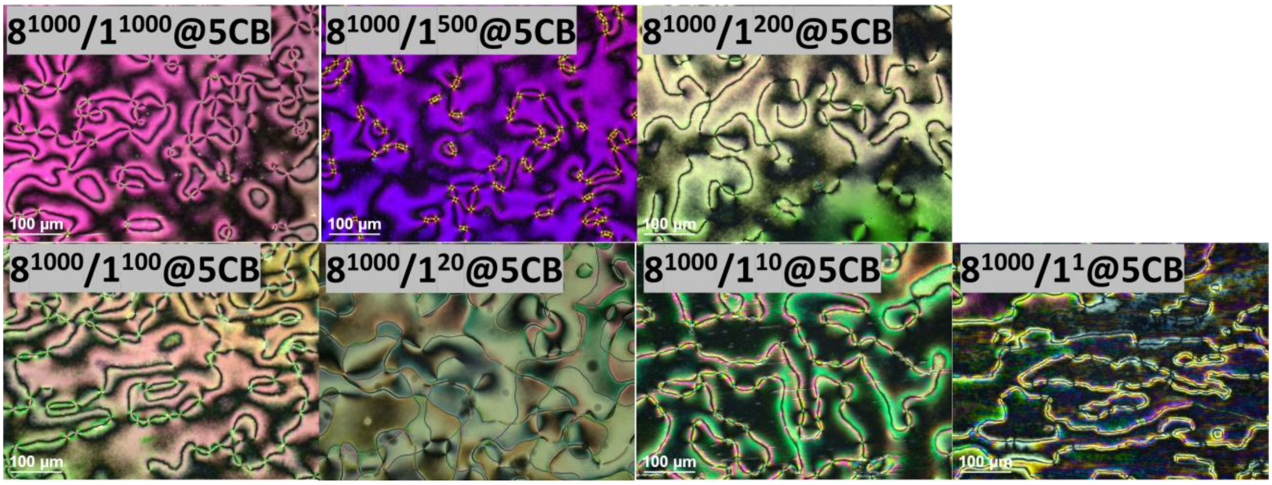

Figure S186. POM images of **$8^{1000}/1^{m}$@5CB** at 20× magnification.

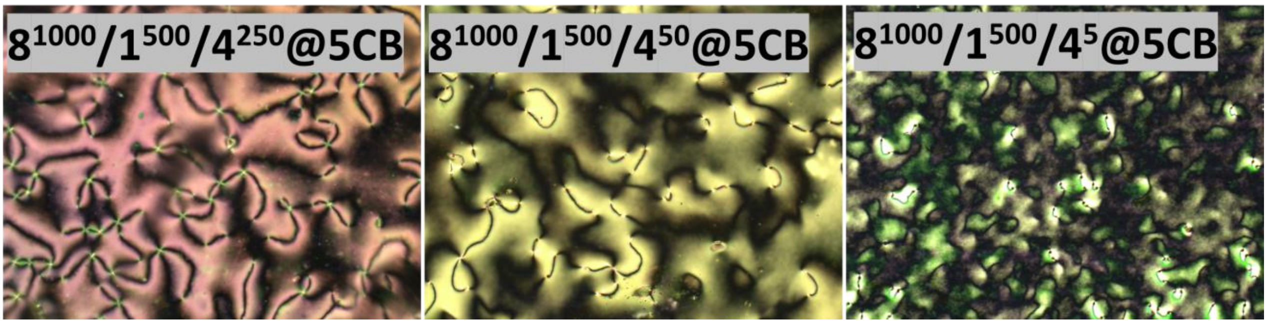

Figure S187. POM images of **$8^{1000}/1^{250}/4^{p}$@5CB** at 20× magnification.

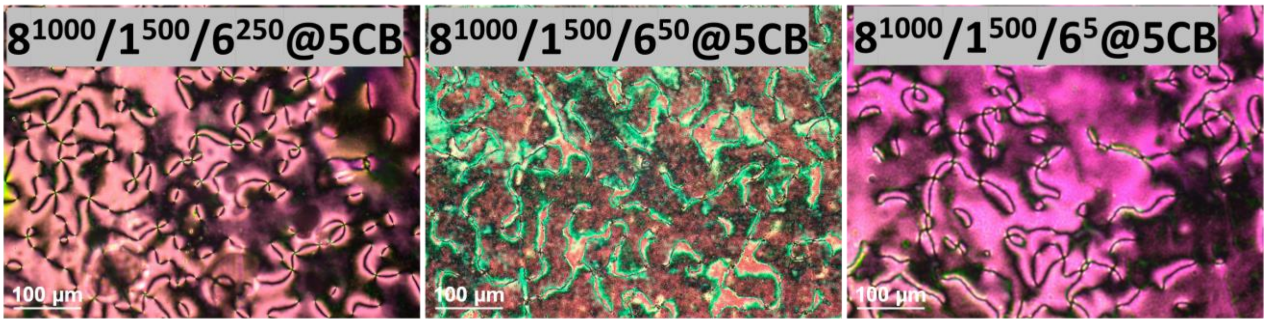

Figure S188. POM images of **$8^{1000}/1^{250}/6^{p}$@5CB** at 20× magnification.

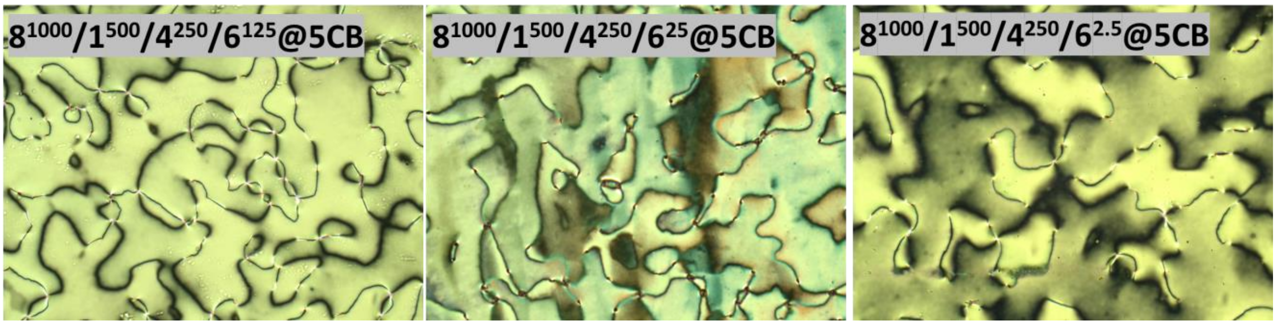

Figure S189. POM images of 8/1/4/6**@5CB** at 20× magnification.

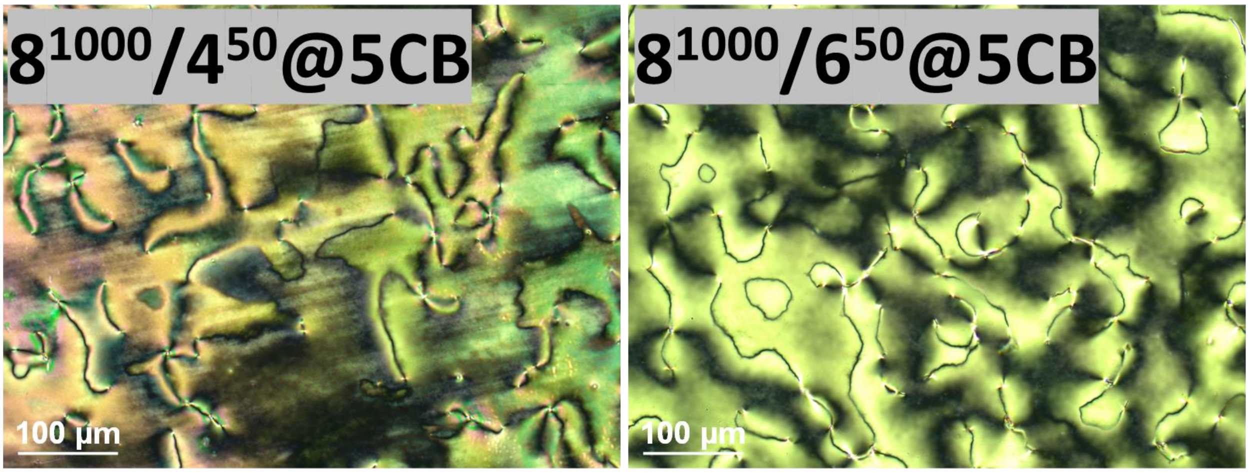

Figure S190. POM images of **$8^{1000}/4^{50}$@5CB** and **$8^{1000}/6^{50}$@5CB** at 20× magnification.

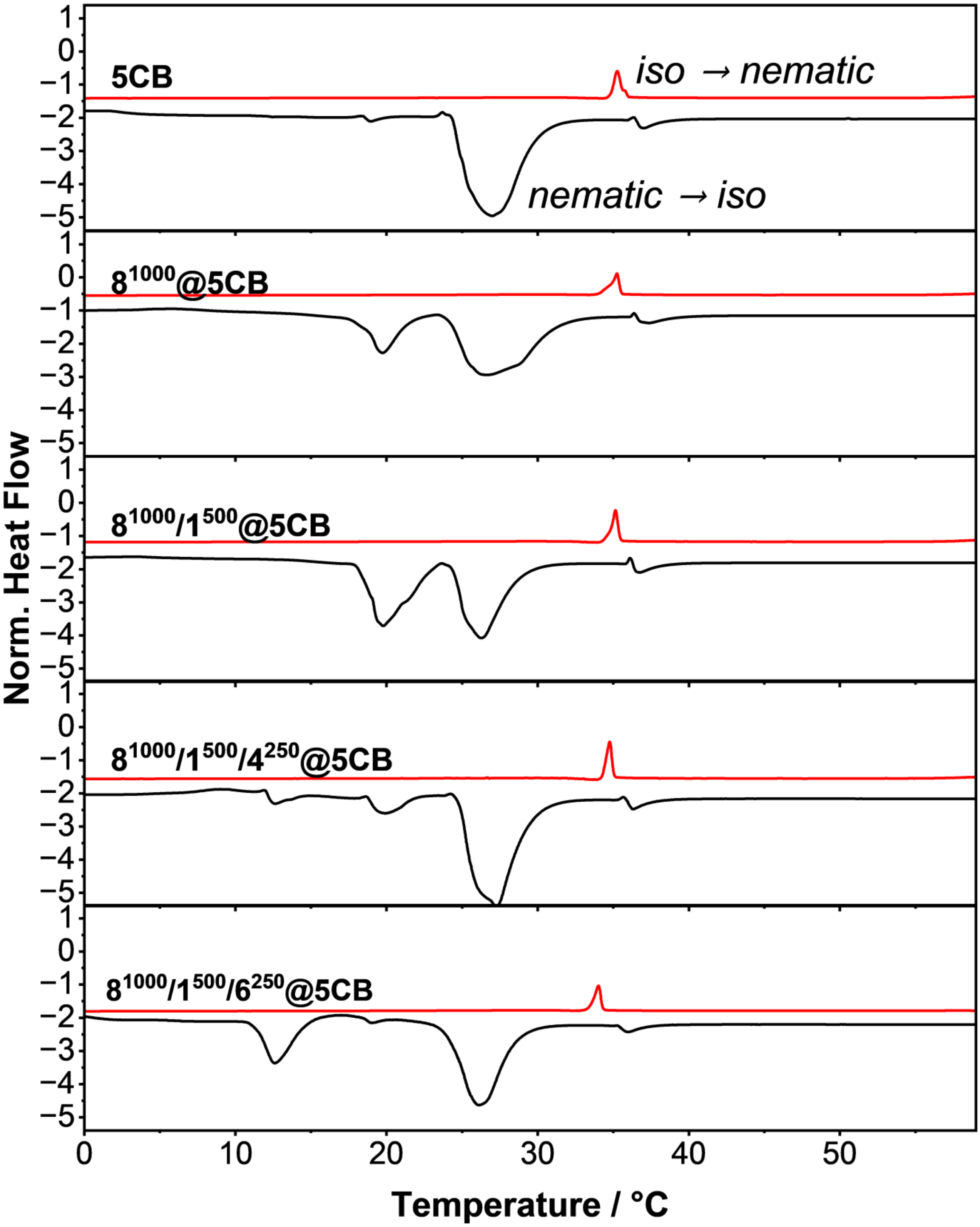

Figure S191. Differential scanning calorimetry of various systems at cooling (red) and heating (black) cycle.

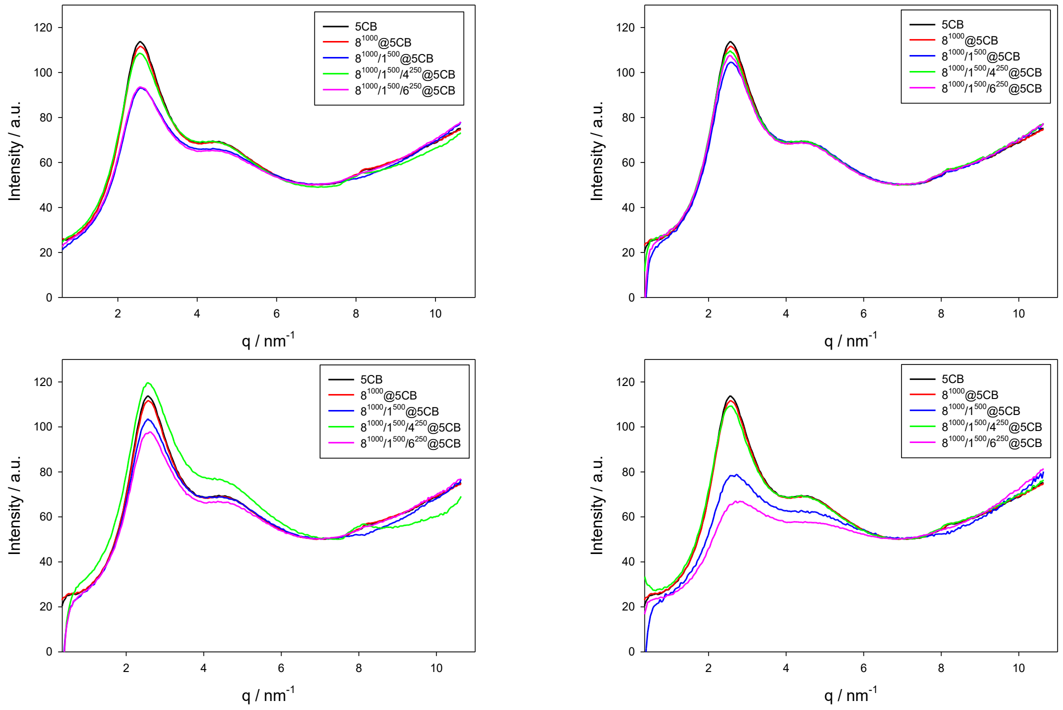

Figure S192. Small angle X-ray scattering (SAXS) profile of **5CB** (black), **8^{1000}@5CB** (red), **$8^{1000}/1^{500}$@5CB** (magenta), **$8^{1000}/1^{500}/4^{250}$@5CB** (green), and **$8^{1000}/1^{500}/6^{250}$@5CB** (magenta), four replicates.

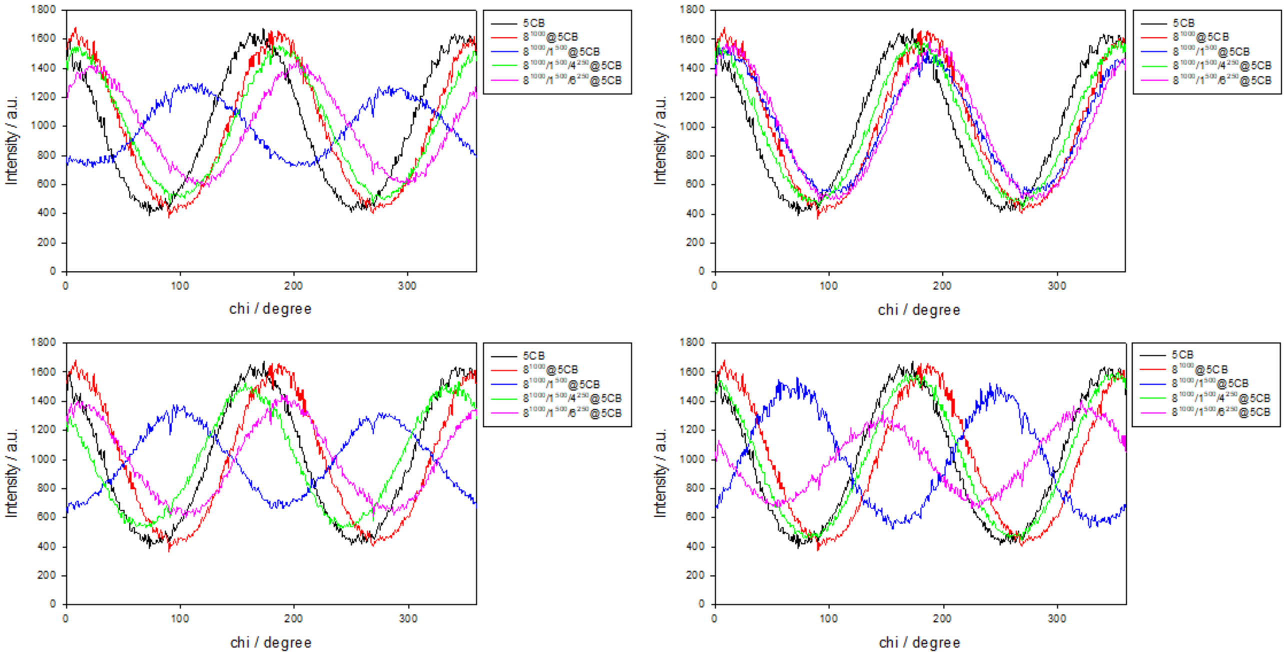

Figure S193. Chi-dependence (orientation distribution) profile of **5CB** (black), **8^{1000}@5CB** (red), **$8^{1000}/1^{500}$@5CB** (magenta), **$8^{1000}/1^{500}/4^{250}$@5CB** (green), and **$8^{1000}/1^{500}/6^{250}$@5CB** (magenta), four replicates.

5. Crystallographic data

Bond precision:	C-C = 0.0019 A		Wavelength=0.71073
Cell:	a=25.3924(15)	b=25.3924(15)	c=10.2317(6)
	alpha=90	beta=90	gamma=120
Temperature:	100 K		

	Calculated	Reported
Volume	5713.3(8)	5713.3(8)
Space group	R -3	R -3
Hall group	-R 3	-R 3
Moiety formula	C36 H26 O2	C36 H26 O2
Sum formula	C36 H26 O2	C36 H26 O2
Mr	490.57	490.57
Dx,g cm-3	1.283	1.283
Z	9	9
Mu (mm-1)	0.078	0.078
F000	2322.0	2322.0
F000'	2322.98	
h,k,lmax	32,32,13	32,32,13
Nref	2870	2774
Tmin,Tmax	0.977,0.986	0.735,0.998
Tmin'	0.974	

Correction method= # Reported T Limits: Tmin=0.735 Tmax=0.998
AbsCorr = MULTI-SCAN

Data completeness= 0.967 Theta(max)= 27.320

R(reflections)= 0.0398(1829) wR2(reflections)= 0.1055(2774)

S = 1.048 Npar= 174

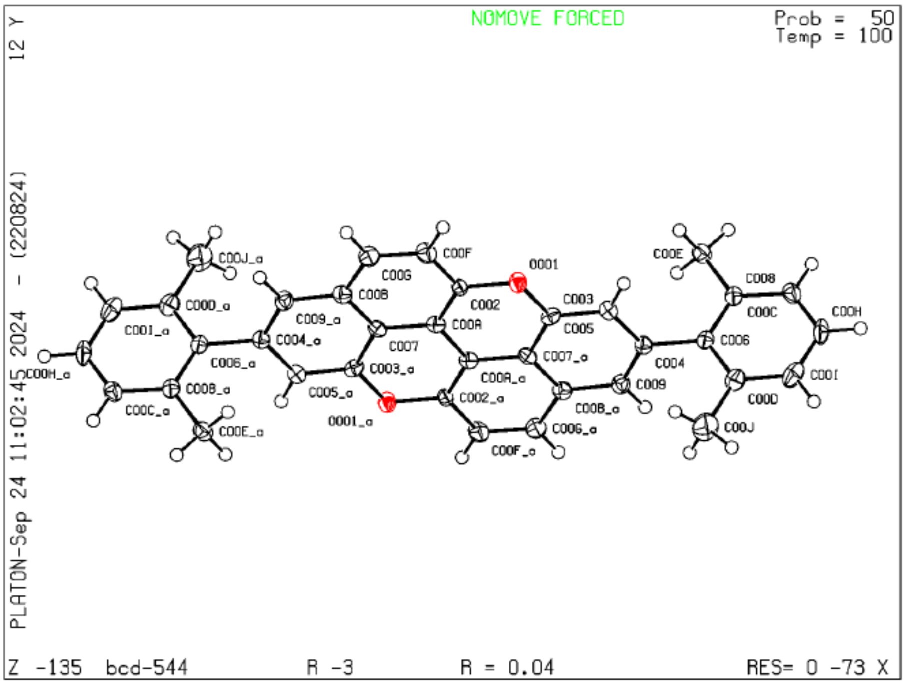

Figure S194. Crystal data and structure refinement for **8**.

Bond precision: C-C = 0.0049 A Wavelength=0.70000

Cell:	a=10.927(2)	b=13.088(3)	c=15.800(3)
	alpha=90.98(3)	beta=99.47(3)	gamma=100.93(3)
Temperature:	100 K		

	Calculated	Reported
Volume	2185.7(8)	2185.6(8)
Space group	P -1	P -1
Hall group	-P 1	-P 1
Moiety formula	2(C48 H48 B2 O6), C H2 Cl2	2(C48 H48 B2 O6), C H2 Cl2
Sum formula	C97 H98 B4 Cl2 O12	C97 H98 B4 Cl2 O12
Mr	1569.89	1569.89
Dx,g cm-3	1.193	1.193
Z	1	1
Mu (mm-1)	0.128	0.128
F000	830.0	830.0
F000'	830.62	
h,k,lmax	13,16,19	13,16,19
Nref	8991	8841
Tmin,Tmax	0.992,0.997	
Tmin'	0.987	

Correction method= Not given

Data completeness= 0.983 Theta(max)= 25.999

R(reflections)= 0.0827(5704) wR2(reflections)= 0.2519(8841)

S = 1.054 Npar= 545

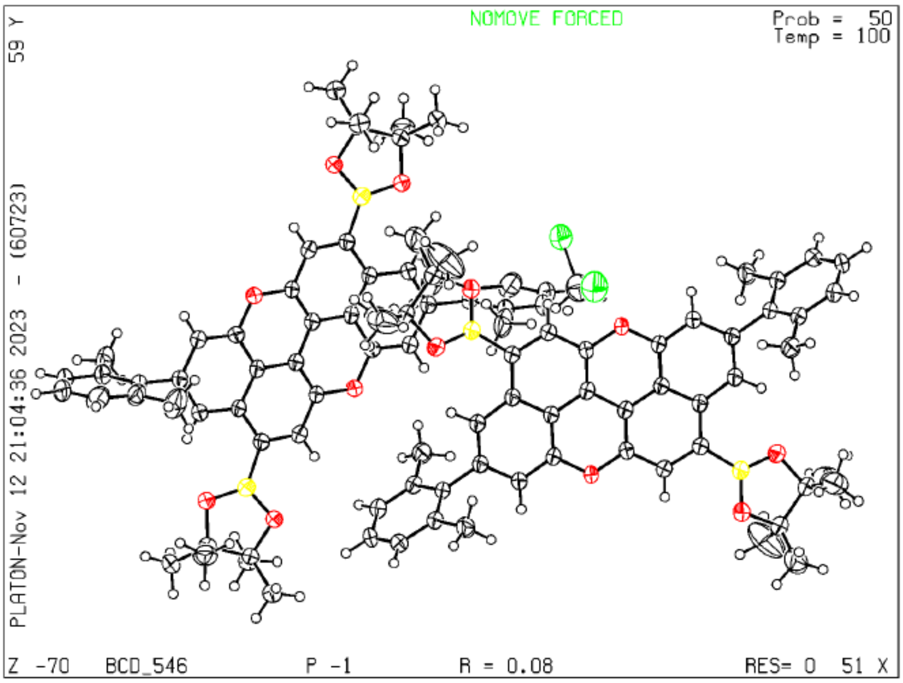

Figure S195. Crystal data and structure refinement for **10**.

Bond precision: C-C = 0.0100 A Wavelength=0.71073

Cell: a=31.260(4) b=6.5387(7) c=15.846(2)
alpha=90 beta=114.133(10) gamma=90

Temperature: 100 K

	Calculated	Reported
Volume	2955.8(7)	2955.9(7)
Space group	C c	C c
Hall group	C -2yc	C -2yc
Moiety formula	C43 H28 O3	?
Sum formula	C43 H28 O3	C172 H112 O12
Mr	592.65	2370.61
Dx,g cm-3	1.332	1.332
Z	4	1
Mu (mm-1)	0.082	0.082
F000	1240.0	1240.0
F000'	1240.54	
h,k,lmax	37,7,19	42,8,21
Nref	5399[2701]	4414
Tmin,Tmax	0.981,0.998	0.991,0.999
Tmin'	0.965	

Correction method= # Reported T Limits: Tmin=0.991 Tmax=0.999
AbsCorr = MULTI-SCAN

Data completeness= 1.63/0.82 Theta(max)= 25.348

R(reflections)= 0.0565(1975) wR2(reflections)= 0.1420(4414)

S = 0.730 Npar= 419

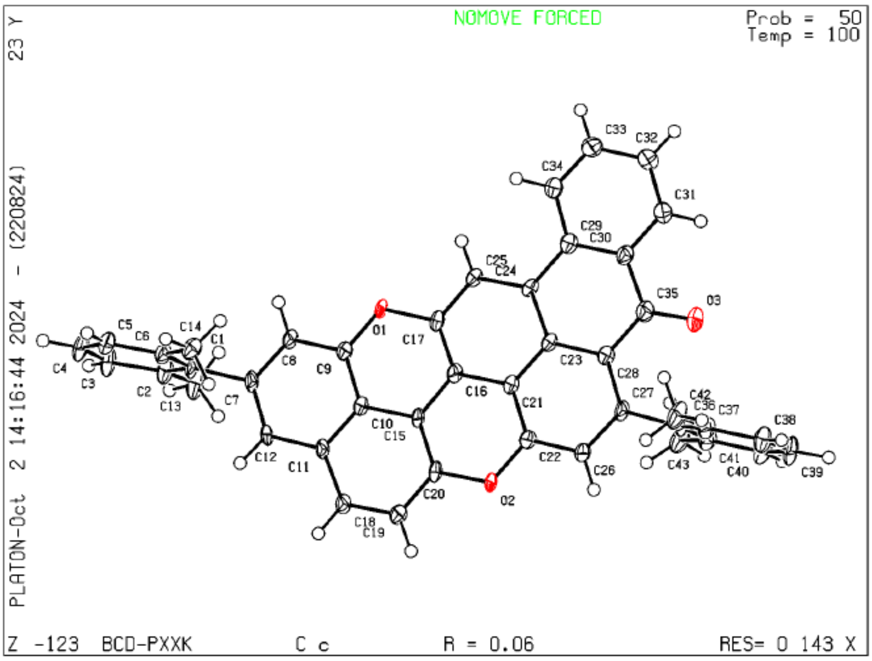

Figure S196. Crystal data and structure refinement for **1**.

```
Bond precision:   C-C = 0.0042 A           Wavelength=0.70000

Cell:              a=29.453(6)      b=6.8700(14)       c=19.095(4)
                   alpha=90         beta=99.18(3)      gamma=90
Temperature:       100 K

                 Calculated                    Reported
Volume           3814.2(14)                    3814.2(14)
Space group      C 2/c                         C 1 2/c 1
Hall group       -C 2yc                        -C 2yc
Moiety formula   C50 H30 O4 [+ solvent]        C50 H30 O4
Sum formula      C50 H30 O4 [+ solvent]        C50 H30 O4
Mr               694.74                        694.74
Dx,g cm-3        1.210                         1.210
Z                4                             4
Mu (mm-1)        0.073                         0.074
F000             1448.0                        1448.0
F000'            1448.59
h,k,lmax         36,8,23                       36,8,23
Nref             3928                          3882
Tmin,Tmax        0.996,0.999
Tmin'            0.993

Correction method= Not given

Data completeness= 0.988          Theta(max)= 26.000

                                                wR2(reflections)=
R(reflections)= 0.0696( 2492)                   0.2323( 3882)
S = 1.029                  Npar= 247
```

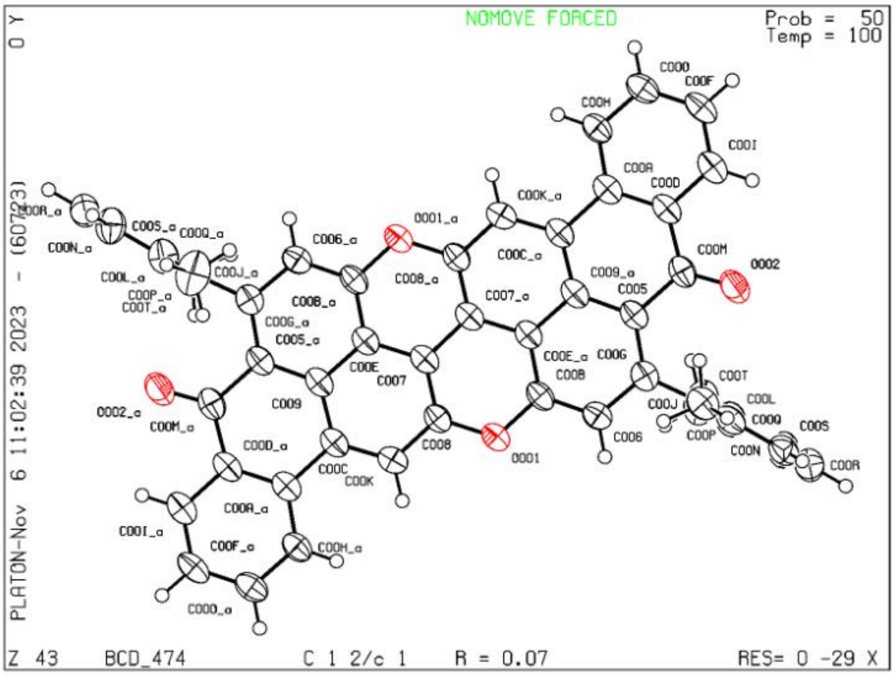

Figure S197. Crystal data and structure refinement for **2**.

6. Computational studies

Cartesian coordinates for optimized geometry of **1** in solution (CH_2Cl_2).

C	-5.2013099748	-1.4586999939	-2.5457899870
C	-5.8697099547	-1.7668699925	-1.2249699935
C	-7.1589899495	-2.3118999899	-1.2046099957
C	-7.8037799421	-2.5857999857	0.0000900000
C	-7.1590399567	-2.3116399893	1.2047499948
C	-5.8697699634	-1.7665899889	1.2250399931
C	-5.2014599754	-1.4580199906	2.5458199861
C	-5.2281299722	-1.4924699931	0.0000200000
C	-3.8031899813	-1.0130299971	-0.0000100000
C	-2.8322199836	-2.0368499916	0.0000000000
C	-1.4840699928	-1.7312999917	-0.0000100000
C	-1.0642299929	-0.3784199981	-0.0000300000
C	0.3185899984	-0.1057799992	0.0000100000
C	0.7758599956	1.1979899941	0.0000300000
O	2.1288399879	1.4786999925	0.0001100000
C	3.0422699865	0.4346099978	0.0000500000
C	2.6135299879	-0.9159599949	0.0000500000
C	1.2303699929	-1.2025899940	0.0000400000
C	0.7728999953	-2.5029199867	0.0000200000
O	-0.5842399966	-2.7676299841	-0.0000100000
C	1.6894999930	-3.5754399797	0.0000200000
C	3.0463399837	-3.3092599810	0.0000300000
C	3.5604299847	-1.9754899893	0.0000400000
C	4.9400399732	-1.6407999944	0.0000200000
C	5.3491799783	-0.3156999987	0.0000100000
C	6.8086599794	0.0328299999	0.0000100000
C	7.4891899657	0.1984899988	1.2255499929
C	6.7760099573	0.0255799999	2.5486999854
C	8.8506299404	0.5269599976	1.2054799948
C	9.5301899448	0.6906999943	0.0000000000
C	8.8506299404	0.5268699973	-1.2054899962
C	7.4891999671	0.1983999990	-1.2255499929
C	6.7760299602	0.0253699999	-2.5486899893
C	4.3876499780	0.7339599983	0.0000200000
C	-0.1412599991	2.2527199913	-0.0000100000
C	-1.5227399930	2.0203899875	-0.0000600000
C	-2.4924199890	3.1234199835	-0.0001000000
C	-3.8782399833	2.8328299873	-0.0000300000
C	-4.8180799781	3.8776399810	-0.0000100000
C	-4.4126999782	5.2033999761	-0.0000800000
C	-3.0425599862	5.5003299756	-0.0002000000
C	-2.1020699871	4.4792599774	-0.0002200000
C	-2.0186699922	0.6718399969	-0.0000400000
C	-3.4136199813	0.3429399982	-0.0000300000
C	-4.4030499767	1.4392099951	-0.0000100000
O	-5.6235599483	1.2388599956	0.0000100000
H	-5.8296999773	-1.7758299892	-3.3834999819

H	-4.2311499780	-1.9600299924	-2.6433099857
H	-5.0168199772	-0.3827799981	-2.6533299879
H	-7.6580399880	-2.5231199878	-2.1474999880
H	-8.8036299554	-3.0120799876	0.0001200000
H	-7.6581199467	-2.5226399870	2.1476699915
H	-5.8296099642	-1.7755299934	3.3835799830
H	-5.0176499757	-0.3819799981	2.6533299879
H	-4.2309899813	-1.9587399907	2.6432799866
H	-3.1424899849	-3.0762499844	0.0000200000
H	1.3182299939	-4.5950399765	0.0000100000
H	3.7492399839	-4.1373599802	0.0000200000
H	5.6809199531	-2.4355699894	0.0000200000
H	6.3737299727	-0.9881099946	2.6615499885
H	5.9266199472	0.7123499939	2.6440899876
H	7.4567599755	0.2131899987	3.3843999854
H	9.3784999751	0.6550099974	2.1474599875
H	10.5866899658	0.9460999972	-0.0000200000
H	9.3785299795	0.6548799944	-2.1474499914
H	7.4567899798	0.2128799987	-3.3843999854
H	5.9266599530	0.7121499967	-2.6441599872
H	6.3737399742	-0.9883299947	-2.6614399884
H	4.7092699785	1.7703599902	0.0000200000
H	0.2559699987	3.2603199847	0.0000200000
H	-5.8692099881	3.6103899806	0.0000500000
H	-5.1475399770	6.0031899842	-0.0000600000
H	-2.7084699884	6.5341299876	-0.0002900000
H	-1.0516599968	4.7476299761	-0.0003300000
C	-5.2013099748	-1.4586999939	-2.5457899870
C	-5.8697099547	-1.7668699925	-1.2249699935
C	-7.1589899495	-2.3118999899	-1.2046099957
C	-7.8037799421	-2.5857999857	0.0000900000
C	-7.1590399567	-2.3116399893	1.2047499948
C	-5.8697699634	-1.7665899889	1.2250399931
C	-5.2014599754	-1.4580199906	2.5458199861
C	-5.2281299722	-1.4924699931	0.0000200000
C	-3.8031899813	-1.0130299971	-0.0000100000
C	-2.8322199836	-2.0368499916	0.0000000000
C	-1.4840699928	-1.7312999917	-0.0000100000
C	-1.0642299929	-0.3784199981	-0.0000300000
C	0.3185899984	-0.1057799992	0.0000100000
C	0.7758599956	1.1979899941	0.0000300000
O	2.1288399879	1.4786999925	0.0001100000
C	3.0422699865	0.4346099978	0.0000500000
C	2.6135299879	-0.9159599949	0.0000500000
C	1.2303699929	-1.2025899940	0.0000400000
C	0.7728999953	-2.5029199867	0.0000200000
O	-0.5842399966	-2.7676299841	-0.0000100000
C	1.6894999930	-3.5754399797	0.0000200000
C	3.0463399837	-3.3092599810	0.0000300000
C	3.5604299847	-1.9754899893	0.0000400000
C	4.9400399732	-1.6407999944	0.0000200000

C	5.3491799783	-0.3156999987	0.0000100000
C	6.8086599794	0.0328299999	0.0000100000
C	7.4891899657	0.1984899988	1.2255499929
C	6.7760099573	0.0255799999	2.5486999854
C	8.8506299404	0.5269599976	1.2054799948
C	9.5301899448	0.6906999943	0.0000000000
C	8.8506299404	0.5268699973	-1.2054899962
C	7.4891999671	0.1983999990	-1.2255499929
C	6.7760299602	0.0253699999	-2.5486899893
C	4.3876499780	0.7339599983	0.0000200000
C	-0.1412599991	2.2527199913	-0.0000100000
C	-1.5227399930	2.0203899875	-0.0000600000
C	-2.4924199890	3.1234199835	-0.0001000000
C	-3.8782399833	2.8328299873	-0.0000300000
C	-4.8180799781	3.8776399810	-0.0000100000
C	-4.4126999782	5.2033999761	-0.0000800000
C	-3.0425599862	5.5003299756	-0.0002000000
C	-2.1020699871	4.4792599774	-0.0002200000
C	-2.0186699922	0.6718399969	-0.0000400000
C	-3.4136199813	0.3429399982	-0.0000300000
C	-4.4030499767	1.4392099951	-0.0000100000
O	-5.6235599483	1.2388599956	0.0000100000
H	-5.8296999773	-1.7758299892	-3.3834999819
H	-4.2311499780	-1.9600299924	-2.6433099857
H	-5.0168199772	-0.3827799981	-2.6533299879
H	-7.6580399880	-2.5231199878	-2.1474999880
H	-8.8036299554	-3.0120799876	0.0001200000
H	-7.6581199467	-2.5226399870	2.1476699915
H	-5.8296099642	-1.7755299934	3.3835799830
H	-5.0176499757	-0.3819799981	2.6533299879
H	-4.2309899813	-1.9587399907	2.6432799866
H	-3.1424899849	-3.0762499844	0.0000200000
H	1.3182299939	-4.5950399765	0.0000100000
H	3.7492399839	-4.1373599802	0.0000200000
H	5.6809199531	-2.4355699894	0.0000200000
H	6.3737299727	-0.9881099946	2.6615499885
H	5.9266199472	0.7123499939	2.6440899876
H	7.4567599755	0.2131899987	3.3843999854
H	9.3784999751	0.6550099974	2.1474599875
H	10.5866899658	0.9460999972	-0.0000200000
H	9.3785299795	0.6548799944	-2.1474499914
H	7.4567899798	0.2128799987	-3.3843999854
H	5.9266599530	0.7121499967	-2.6441599872
H	6.3737399742	-0.9883299947	-2.6614399884
H	4.7092699785	1.7703599902	0.0000200000
H	0.2559699987	3.2603199847	0.0000200000
H	-5.8692099881	3.6103899806	0.0000500000
H	-5.1475399770	6.0031899842	-0.0000600000
H	-2.7084699884	6.5341299876	-0.0002900000
H	-1.0516599968	4.7476299761	-0.0003300000

Cartesian coordinates for optimized geometry of **2** in solution (CH_2Cl_2).

C	5.7759804472	-1.7177893409	-2.5460799021
C	6.3907304255	-2.1229293272	-1.2253399095
C	7.5822704374	-2.8574292926	-1.2050199122
C	8.1781104799	-3.2256192739	-0.0002899171
C	7.5824304606	-2.8575692917	1.2045600784
C	6.3909004502	-2.1230593248	1.2251200787
C	5.7763704508	-1.7179893434	2.5459800728
C	5.7987204089	-1.7550093426	-0.0000599171
C	4.4643404187	-1.0628693830	0.0000700829
C	3.3474404499	-1.9258294071	0.0000400829
C	2.0633104395	-1.4163694421	0.0001200829
C	1.8588004034	-0.0157694540	0.0001900829
C	0.5342004018	0.4682905089	0.0002600829
C	0.2861103650	1.8263304973	0.0003000829
O	-1.0118796400	2.3006404589	0.0003900829
C	-2.0633096140	1.4163704369	0.0002100829
C	-1.8588095793	0.0157604485	0.0001000829
C	-0.5341995710	-0.4682895146	0.0001800829
C	-0.2861095363	-1.8263394986	0.0001000829
O	1.0118804708	-2.3006394641	0.0001400829
C	-1.3509195085	-2.7316095266	0.0000000829
C	-2.6783795118	-2.2876995626	-0.0000699171
C	-3.8070294848	-3.2280695866	-0.0000799171
C	-3.6294594475	-4.6272595764	0.0001900829
C	-4.7161094193	-5.4914595897	0.0002000829
C	-6.0237294154	-4.9868796334	-0.0000599171
C	-6.2204794594	-3.6142396495	-0.0003499171
C	-5.1310494870	-2.7272496250	-0.0003499171
C	-5.4350195066	-1.2690696376	-0.0007099171
O	-6.6090295180	-0.8832496731	-0.0013899171
C	-2.9611195504	-0.8760295740	-0.0000699171
C	-4.2878095532	-0.3368796132	-0.0002299171
C	-4.4643495894	1.0628703726	-0.0000299171
C	-5.7987196151	1.7550303351	0.0000600829
C	-6.3909196064	2.1225903204	1.2253600765
C	-5.7764396142	1.7170303367	2.5460900729
C	-7.5824596182	2.8571202848	1.2050600768
C	-8.1780796289	3.2256702654	0.0003400829
C	-7.5821996335	2.8579802825	-1.2045199085
C	-6.3906796245	2.1234603194	-1.2251099133
C	-5.7759495961	1.7187003353	-2.5459699020
C	-3.3474396191	1.9258204005	0.0002000829
C	1.3509103378	2.7316105214	0.0002500829
C	2.6783803426	2.2876905560	0.0001800829
C	3.8070203088	3.2280605800	0.0000600829
C	5.1310503178	2.7272306169	0.0000100829
C	6.2204803061	3.6142306429	-0.0001599171
C	6.0237302621	4.9868606307	-0.0002899171
C	4.7161102448	5.4914505937	-0.0002199171

C	3.6294602730	4.6272505698	-0.0000399171
C	2.9611203759	0.8760305688	0.0001700829
C	4.2878003826	0.3368806080	0.0001300829
C	5.4350103518	1.2690606310	0.0001800829
O	6.6090203632	0.8832106674	0.0003500829
H	6.3502004257	-2.1248293273	-3.3837499018
H	5.7538404136	-0.6263793514	-2.6538399030
H	4.7420304451	-2.0691593664	-2.6441299034
H	8.0435604542	-3.1418092778	-2.1478599079
H	9.1018004626	-3.7984592429	-0.0003799171
H	8.0438604447	-3.1420392741	2.1473000702
H	4.7422604467	-2.0688993658	2.6439900684
H	5.7547303838	-0.6265993515	2.6539800715
H	6.3504404604	-2.1254893277	3.3835400671
H	3.4948704743	-3.0004193985	-0.0000299171
H	-1.1119694819	-3.7878695127	-0.0000299171
H	-2.6331194403	-5.0541595465	0.0004100829
H	-4.5452593928	-6.5642695930	0.0004300829
H	-6.8730094096	-5.6637996416	-0.0000399171
H	-7.2178894895	-3.1885296773	-0.0005899171
H	-4.7423096179	2.0678603634	2.6442300714
H	-5.7548395531	0.6256003443	2.6537400685
H	-6.3504996224	2.1242603190	3.3837800649
H	-8.0438996038	3.1412102703	2.1479000725
H	-9.1017596631	3.7985302426	0.0004400829
H	-8.0434496445	3.1427602727	-2.1472499042
H	-6.3498895870	2.1263803193	-3.3835199002
H	-5.7543195836	0.6273303410	-2.6541899061
H	-4.7418296171	2.0696303606	-2.6437399051
H	-3.4948696488	3.0004203933	0.0003700829
H	1.1119703074	3.7878605061	0.0002600829
H	7.2178902832	3.1885206707	-0.0001899171
H	6.8730102563	5.6637906456	-0.0004399171
H	4.5452702198	6.5642605970	-0.0002999171
H	2.6331202711	5.0541505399	0.0000000829

Cartesian coordinates for optimized geometry of **3** in solution (CH_2Cl_2).

C	5.1206399733	-5.5727399918	-2.5484599877
C	5.2729299718	-6.2930599765	-1.2278199938
C	5.5201499904	-7.6707799822	-1.2079499929
C	5.6445699768	-8.3605399832	-0.0035200000
C	5.5204399795	-7.6716599510	1.2014399966
C	5.2732099753	-6.2939499468	1.2223699924
C	5.1210599760	-5.5746299482	2.5435599867
C	5.1469299733	-5.6074899478	-0.0024600000
C	4.9882699777	-4.1124899796	-0.0019200000
C	6.1973299674	-3.3854699817	-0.0018300000
C	6.1893699780	-2.0034699907	-0.0013600000
C	4.9588299764	-1.3010099960	-0.0010600000
C	4.9924399735	0.1077799996	-0.0005900000
C	3.8187299792	0.8380099960	-0.0002700000
O	3.8375899819	2.2190399893	0.0002200000
C	5.0548299770	2.8848399840	0.0005900000
C	6.2799899874	2.1734499917	0.0003000000
C	6.2603099929	0.7609099986	-0.0003400000
C	7.4308299747	0.0326799999	-0.0006100000
O	7.3955099891	-1.3487499931	-0.0012100000
C	8.6764699422	0.6951999956	-0.0002700000
C	8.7101799592	2.0773599886	0.0003700000
C	7.5194099531	2.8683099856	0.0006800000
C	7.4917999735	4.2875999779	0.0013400000
C	6.2869199863	4.9742999746	0.0016100000
C	6.2624899913	6.4745799830	0.0023000000
C	6.2463199763	7.1741099711	1.2282199935
C	6.2601699726	6.4397599639	2.5510199883
C	6.2190599945	8.5743299567	1.2087599938
C	6.2067899626	9.2737999361	0.0036000000
C	6.2216299436	8.5754999675	-1.2022099918
C	6.2489399856	7.1752999848	-1.2229699947
C	6.2655399571	6.4422399529	-2.5464599889
C	5.0539399750	4.2629699804	0.0012200000
C	2.5911299855	0.1718499994	-0.0004100000
C	2.5175499849	-1.2275199926	-0.0008700000
C	1.2311799939	-1.9357799918	-0.0009500000
C	1.2201199944	-3.3556499834	-0.0010700000
C	0.0000400000	-4.0295499826	-0.0007900000
C	-1.2200599963	-3.3556699810	-0.0004600000
C	-1.2311399934	-1.9357899879	-0.0006300000
C	-2.5175199859	-1.2275499917	-0.0004700000
C	-2.5911099879	0.1718099989	-0.0012100000
C	-3.8187299792	0.8379599941	-0.0008000000
O	-3.8376099795	2.2189899873	-0.0015900000
C	-5.0548499746	2.8847699845	-0.0010000000
C	-6.2799999889	2.1733599892	0.0004100000
C	-6.2602999914	0.7608199962	0.0010900000
C	-7.4308099718	0.0325799998	0.0023600000

O	-7.3954699833	-1.3488599931	0.0029200000
C	-8.6764599408	0.6950799941	0.0030700000
C	-8.7101899606	2.0772399871	0.0024400000
C	-7.5194299560	2.8682099870	0.0010500000
C	-7.4918499807	4.2874899778	0.0002200000
C	-6.2869799950	4.9742099775	-0.0012700000
C	-6.2625699500	6.4744899699	-0.0023700000
C	-6.2410999606	7.1754099478	1.2226799950
C	-6.2493599935	6.4425399435	2.5463399874
C	-6.2140199520	8.5756099834	1.2015299938
C	-6.2070099944	9.2737199774	-0.0044600000
C	-6.2270399868	8.5740599705	-1.2094099928
C	-6.2543799802	7.1738299834	-1.2284799942
C	-6.2766099738	6.4393099516	-2.5510699849
C	-5.0539799755	4.2628999808	-0.0018800000
C	-4.9924299774	0.1077199994	0.0004400000
C	-4.9587899759	-1.3010799956	0.0010900000
C	-6.1893299722	-2.0035599879	0.0022700000
C	-6.1972599573	-3.3855499827	0.0028000000
C	-4.9881999729	-4.1125599792	0.0021000000
C	-5.1468399761	-5.6075599579	0.0026600000
C	-5.2723999743	-6.2931699925	1.2280399939
C	-5.1197599728	-5.5728799591	2.5486599849
C	-5.5196099650	-7.6708899453	1.2082099935
C	-5.6444599609	-8.3606099405	0.0038100000
C	-5.5207699744	-7.6716799539	-1.2011799960
C	-5.2735499717	-6.2939799511	-1.2221499923
C	-5.1218399726	-5.5746099982	-2.5433599894
C	-3.7483199829	-3.4395699849	0.0009800000
C	-2.4652299856	-4.1712499809	0.0001500000
O	-2.3954299878	-5.4049699672	0.0000100000
C	-3.7261999840	-2.0061699904	0.0005400000
C	0.0000200000	-1.2652499942	-0.0008300000
C	3.7262499807	-2.0061199885	-0.0011800000
C	3.7483799810	-3.4395199830	-0.0015600000
C	2.4652999852	-4.1712199818	-0.0014500000
O	2.3955199903	-5.4049499643	-0.0016200000
H	5.2924699619	-6.2553799663	-3.3860899816
H	5.8234699740	-4.7367899768	-2.6451099872
H	4.1118799812	-5.1560599735	-2.6573999850
H	5.6155899575	-8.2038099710	-2.1510299916
H	5.8388899862	-9.4299299672	-0.0039400000
H	5.6161099799	-8.2053799340	2.1441099889
H	5.2936199698	-6.2577399909	3.3806599831
H	4.1120799784	-5.1586699760	2.6531599844
H	5.8234099653	-4.7383199762	2.6405099874
H	7.1453599850	-3.9125099808	-0.0020900000
H	9.5913699740	0.1118899994	-0.0004900000
H	9.6709599724	2.5840899866	0.0006500000
H	8.4282199490	4.8385599775	0.0016500000
H	7.1631099645	5.8282199745	2.6643699844

H	5.4062199896	5.7585399835	2.6454599851
H	6.2231899580	7.1445399720	3.3870499831
H	6.2068299684	9.1169699623	2.1509499906
H	6.1850099812	10.3604999415	0.0041100000
H	6.2113699913	9.1190499463	-2.1438999902
H	6.2292699925	7.1477899668	-3.3818599824
H	5.4123399769	5.7603599826	-2.6428899883
H	7.1691899460	5.8315899866	-2.6589699850
H	4.1117899787	4.8013899777	0.0014200000
H	1.6953799932	0.7806899971	-0.0001000000
H	0.0000400000	-5.1127999749	-0.0007700000
H	-1.6953699917	0.7806699942	-0.0021400000
H	-9.5913499711	0.1117599996	0.0040900000
H	-9.6709799753	2.5839599889	0.0029800000
H	-8.4282699562	4.8384499774	0.0007500000
H	-5.3943599641	5.7623199492	2.6384799895
H	-7.1512499390	5.8301599910	2.6635799864
H	-6.2100899645	7.1482799849	3.3814399850
H	-6.1977999826	9.1193099310	2.1430499887
H	-6.1852899688	10.3604199299	-0.0052800000
H	-6.2208099835	9.1165399529	-2.1517399887
H	-6.2412999896	7.1438399764	-3.3873799833
H	-7.1819899489	5.8306099504	-2.6602199863
H	-5.4252799472	5.7553599460	-2.6492699869
H	-4.1118399807	4.8013299743	-0.0031200000
H	-7.1452799734	-3.9126099794	0.0037000000
H	-5.8230099603	-4.7373399771	2.6457599862
H	-4.1111899817	-5.1556599737	2.6571299882
H	-5.2907899724	-6.2556899583	3.3863199832
H	-5.6146799843	-8.2039699413	2.1513099899
H	-5.8387699688	-9.4300099788	0.0042500000
H	-5.6167599683	-8.2053799340	-2.1438299906
H	-5.2945799502	-6.2577199880	-3.3804299815
H	-4.1129299799	-5.1585599760	-2.6532399854
H	-5.8243099899	-4.7383699782	-2.6400899847
H	0.0000100000	-0.1844099993	-0.0008300000

Cartesian coordinates for optimized geometry of **4** in solution (CH_2Cl_2).

C	1.4598805194	5.7047399655	-2.5472191941
C	1.0517805381	6.3181100085	-1.2267491984
C	0.2415305746	7.4594799992	-1.2068791975
C	-0.1622394068	8.0323000394	-0.0023592041
C	0.2420205747	7.4607600260	1.2026207921
C	1.0522805365	6.3194199867	1.2233707882
C	1.4609205167	5.7074199835	2.5443107833
C	1.4546705211	5.7492000057	-0.0014692041
C	2.4187804825	4.5953799623	-0.0010392041
C	3.7812304872	4.9593499194	-0.0011992041
C	4.7708204529	3.9932098967	-0.0008392041
C	4.4217104161	2.6204099108	-0.0004292041

C	5.4577703694	1.6647498881	-0.0000692042
C	5.1667103475	0.3130099031	0.0003507958
O	6.1737603087	-0.6321401226	0.0007507958
C	7.4979903414	-0.2192101604	0.0008007958
C	7.8378503817	1.1562398227	0.0003507958
C	6.8080303643	2.1231898464	-0.0000892042
C	7.0976904321	3.4710798312	-0.0005192041
O	6.0786004352	4.4058698562	-0.0009392041
C	8.4393404492	3.9070097923	-0.0005192041
C	9.4571204151	2.9711397695	-0.0000792042
C	9.1987203796	1.5651497838	0.0003707958
C	10.2001703072	0.5591297601	0.0008407958
C	9.8567302773	-0.7846202227	0.0012807958
C	10.9193702355	-1.8440102497	0.0017707958
C	11.4135002490	-2.3407902609	-1.2235991970
C	10.8951502709	-1.8217802507	-2.5469291892
C	12.4032502273	-3.3316002869	-1.2030292005
C	12.8970501929	-3.8256902946	0.0027307958
C	12.4026001860	-3.3310702842	1.2080307876
C	11.4128502606	-2.3402702596	1.2276307918
C	10.8938002340	-1.8206302481	2.5504507841
C	8.4886702957	-1.1774201828	0.0012607958
C	3.8343603393	-0.1069500566	0.0003507958
C	2.7792803707	0.8153699694	-0.0000392042
C	1.3790303649	0.3838400105	-0.0001592042
C	0.3444604000	1.3554800370	-0.0001392042
C	-0.9936796059	0.9626600740	-0.0001192042
C	-1.3790396414	-0.3838499071	-0.0001892042
C	-2.7792896472	-0.8153698636	-0.0001192042
C	-3.8343596143	0.1069501609	0.0003007958
C	-5.1667096225	-0.3130197992	0.0003107958
O	-6.1737695693	0.6321402232	0.0007507958
C	-7.4979996020	0.2192102647	0.0007707958
C	-7.8378496408	-1.1562397168	0.0003307958
C	-6.8080296764	-2.1231897458	-0.0001192042
C	-7.0976896912	-3.4710797307	-0.0005492041
O	-6.0785997472	-4.4058697557	-0.0009892041
C	-8.4393497098	-3.9070096865	-0.0005392041
C	-9.4571296757	-2.9711396637	-0.0000992042
C	-9.1987296402	-1.5651496780	0.0003507958
C	-10.2001796207	-0.5591196529	0.0008207958
C	-9.8567395908	0.7846203285	0.0012507958
C	-10.9193695476	1.8440103555	0.0017407958
C	-11.4126894966	2.3415603672	-1.2236491989
C	-10.8935095570	1.8232903531	-2.5469391906
C	-12.4024494763	3.3323503903	-1.2031291991
C	-12.8970494520	3.8257004019	0.0026307958
C	-12.4033895067	3.3303203872	1.2079407905
C	-11.4136495299	2.3395003650	1.2275807898
C	-10.8954195163	1.8190903530	2.5504107836
C	-8.4886695548	1.1774202886	0.0012307958

C	-5.4577796829	-1.6647497823	-0.0001192042
C	-4.4217096912	-2.6204098102	-0.0005192041
C	-4.7708297293	-3.9932097909	-0.0009192041
C	-3.7812397584	-4.9593498136	-0.0012692041
C	-2.4187897537	-4.5953798564	-0.0012092041
C	-1.4546697909	-5.7491898985	-0.0015792041
C	-1.0519898118	-6.3183498845	-1.2268191980
C	-1.4603497938	-5.7052498806	-2.5473391904
C	-0.2417298479	-7.4596999253	-1.2068691960
C	0.1622501338	-8.0322899321	-0.0023092041
C	-0.2417898482	-7.4605098839	1.2026207921
C	-1.0520598114	-6.3191698975	1.2232907872
C	-1.4604297895	-5.7069098566	2.5442007832
C	-2.0416297212	-3.2350598747	-0.0008192041
C	-0.6260297134	-2.8216999142	-0.0006992041
O	0.3112202586	-3.6275299404	-0.0008592041
C	-3.0590096873	-2.2243598501	-0.0004892041
C	-0.3444696738	-1.3554799312	-0.0003792042
C	0.9936803309	-0.9626599735	-0.0003392042
C	3.0590104123	2.2243599559	-0.0003492042
C	2.0416204448	3.2350499791	-0.0005792042
C	0.6260204422	2.8217000201	-0.0001892042
O	-0.3112295319	3.6275200448	0.0000807958
H	1.0850905394	6.2999899754	-3.3852991897
H	1.0596204942	4.6888300008	-2.6518591927
H	2.5491805101	5.6301799552	-2.6472791905
H	-0.0722994113	7.9008400381	-2.1499191957
H	-0.7884393793	8.9207000408	-0.0026992041
H	-0.0714394110	7.9031199981	2.1453207834
H	1.0858405370	6.3031300071	3.3819407772
H	2.5502805108	5.6336599303	2.6443107815
H	1.0613404895	4.6913399994	2.6498307824
H	4.0610605168	6.0072998919	-0.0015692041
H	8.6553404784	4.9703597793	-0.0008492041
H	10.4890304178	3.3102297353	-0.0000792042
H	11.2471403229	0.8495397249	0.0008607958
H	9.8127202493	-1.9677202179	-2.6433891929
H	11.3801502313	-2.3352402609	-3.3824791885
H	11.0777902809	-0.7465102613	-2.6590291894
H	12.7867601904	-3.7167402958	-2.1448091960
H	13.6647901706	-4.5951203162	0.0031107958
H	12.7856201839	-3.7158102933	2.1501607863
H	11.3785302611	-2.3335302618	3.3865007766
H	9.8113602639	-1.9667302173	2.6465107828
H	11.0761603093	-0.7452602601	2.6620107801
H	8.2212302731	-2.2290801690	0.0015907958
H	3.6418403103	-1.1729300454	0.0006807958
H	-1.7242795776	1.7624100922	-0.0000892042
H	-3.6418395854	1.1729301512	0.0006507958
H	-8.6553497390	-4.9703596788	-0.0008792042
H	-10.4890296769	-3.3102296347	-0.0000892042

H	-11.2471495835	-0.8495396244	0.0008307958
H	-9.8110895368	1.9696303201	-2.6428191897
H	-11.3782695355	2.3369403644	-3.3825191890
H	-11.0757195576	0.7480103623	-2.6595691935
H	-12.7853594583	3.7180703986	-2.1449091946
H	-13.6647794283	4.5951304182	0.0029807958
H	-12.7870294887	3.7144803969	2.1500607877
H	-11.3804994882	2.3316703627	3.3864607813
H	-9.8130095505	1.9649303216	2.6471207813
H	-11.0780695807	0.7437103635	2.6613607812
H	-8.2212295322	2.2290802749	0.0015807958
H	-4.0610697880	-6.0073097875	-0.0015792041
H	-2.5496697820	-5.6310498696	-2.6473591915
H	-1.0854098132	-6.3004698862	-3.3853791855
H	-1.0604097673	-4.6892198933	-2.6521091919
H	0.0719301377	-7.9012498859	-2.1498791952
H	0.7884601071	-8.9206899335	-0.0025892041
H	0.0718401379	-7.9026799344	2.1453507877
H	-1.0856698085	-6.3027799035	3.3818607815
H	-2.5497497831	-5.6325798267	2.6441707824
H	-1.0603197649	-4.6910298963	2.6497007847
H	1.7242803026	-1.7624199879	-0.0005092041

Cartesian coordinates for optimized geometry of **5** in solution (CH_2Cl_2).

C	10.9853013235	-5.5410304763	-2.5454193725
C	11.1715801147	-6.2535094837	-1.2249287071
C	11.4776787020	-7.6192635756	-1.2040906559
C	11.6342440872	-8.3014310478	0.0009840584
C	11.4835559558	-7.6170471168	1.2055086867
C	11.1777382028	-6.2511541688	1.2253365334
C	10.9993494967	-5.5361311421	2.5456115220
C	11.0192960907	-5.5726655895	-0.0000521871
C	10.7971155363	-4.0858552472	-0.0007274312
C	11.9735643138	-3.3078129829	-0.0013471771
C	11.9062203227	-1.9272117396	-0.0017662352
C	10.6467164415	-1.2781901973	-0.0013884980
C	10.6199672745	0.1307103482	-0.0016546366
C	9.4159482586	0.8103532213	-0.0012197609
O	9.3757305791	2.1907160385	-0.0014692082
C	10.5635023201	2.9080076427	-0.0022143477
C	11.8177489315	2.2496037620	-0.0026768226
C	11.8585554794	0.8374648271	-0.0023789474
C	13.0592205701	0.1599783607	-0.0028030615
O	13.0828007594	-1.2215850374	-0.0024784822
C	14.2754838470	0.8750593064	-0.0035590503
C	14.2498500797	2.2573321729	-0.0038582800
C	13.0263147967	2.9967180844	-0.0034278354
C	12.9382702399	4.4134536059	-0.0036915012
C	11.7051687559	5.0481735574	-0.0032283207
C	11.6163868965	6.5459904364	-0.0034917131

C	11.5716989396	7.2449986558	1.2220157559
C	11.6195923345	6.5128436227	2.5452380288
C	11.4833276687	8.6426797020	1.2016587638
C	11.4385795973	9.3400275750	-0.0039705646
C	11.4823118602	8.6422022254	-1.2093599750
C	11.5706694253	7.2445116540	-1.2292370880
C	11.6175202882	6.5118398265	-2.5522102507
C	10.5037240813	4.2848013660	-0.0024818443
C	8.2182288329	0.0923069697	-0.0004873076
C	8.2048274730	-1.3091641035	-0.0002315396
C	6.9505346644	-2.0718189211	0.0005760278
C	6.9998358294	-3.4904147758	0.0005544207
C	5.8099950585	-4.2166362824	0.0012686213
C	4.5624774433	-3.5956140300	0.0019773213
C	4.4911221460	-2.1779323396	0.0019574524
C	3.1745681834	-1.5249484982	0.0025207152
C	3.0434156446	-0.1314966992	0.0027228287
C	1.7857019117	0.4765611254	0.0030139891
O	1.6959895418	1.8544811849	0.0031561067
C	0.4624379895	2.4599965659	0.0032525428
C	-0.7364715104	1.7071973722	0.0032365921
C	-0.6441021628	0.3000587587	0.0031880929
C	-1.7856945985	-0.4765743463	0.0031497865
O	-1.6959817841	-1.8544947213	0.0031350450
C	-3.0434083949	0.1314844419	0.0030498290
C	-3.1745609072	1.5249364436	0.0030025779
C	-4.4911145947	2.1779224440	0.0026002096
C	-5.6910567658	1.4545706019	0.0018609670
C	-6.9505277851	2.0718153280	0.0012822743
C	-8.2048230279	1.3091655482	0.0003802961
C	-8.2182278274	-0.0923051959	0.0002784437
C	-9.4159486820	-0.8103485222	-0.0006893463
O	-9.3757340717	-2.1907114664	-0.0007709678
C	-10.6199656340	-0.1307026201	-0.0015385123
C	-11.8585551090	-0.8374545663	-0.0025688411
C	-13.0592184534	-0.1599655175	-0.0034281227
O	-13.0827952560	1.2215979070	-0.0032419466
C	-14.2754832120	-0.8750434893	-0.0044889187
C	-14.2498526197	-2.2573164669	-0.0046495892
C	-13.0263191889	-2.9967050719	-0.0037755449
C	-12.9382779129	-4.4134409216	-0.0038814552
C	-11.7051781753	-5.0481637465	-0.0029810291
C	-11.6164004963	-6.5459809112	-0.0030705843
C	-11.5702770404	-7.2446224637	-1.2287319778
C	-11.6166904325	-6.5120801258	-2.5517921848
C	-11.4819318051	-8.6423115534	-1.2086884385
C	-11.4386039924	-9.3400188436	-0.0032160159
C	-11.4837507459	-8.6425523820	1.2023297975
C	-11.5721236043	-7.2448691661	1.2225204058
C	-11.6204510303	-6.5125845905	2.5456552691
C	-10.5037318602	-4.2847942539	-0.0019379992

C	-10.5635069768	-2.9080004088	-0.0018165925
C	-11.8177518949	-2.2495935489	-0.0027226510
C	-10.6467114143	1.2781980503	-0.0014031427
C	-11.9062134963	1.9272216617	-0.0022421784
C	-11.9735550003	3.3078230691	-0.0020415216
C	-10.7971063287	4.0858640262	-0.0010446090
C	-11.0192898464	5.5726745326	-0.0008341473
C	-11.1707340662	6.2532293372	-1.2259752556
C	-10.9836251018	5.5404565307	-2.5461937440
C	-11.4768545085	7.6189920548	-1.2056570363
C	-11.6342612855	8.3014353341	-0.0008514233
C	-11.4844060261	7.6173287979	1.2039380676
C	-11.1785911306	6.2514483914	1.2242859526
C	-11.0010610145	5.5367132899	2.5448298215
C	-9.5291352417	3.4665981130	-0.0003079657
C	-9.4454592015	2.0355197353	-0.0004378047
C	-6.9998255634	3.4904107964	0.0014572410
C	-8.2792940835	4.2526409228	0.0005344033
O	-8.2606568326	5.4880532760	0.0003250448
C	-5.8099832578	4.2166294560	0.0022626998
C	-4.5624670926	3.5956044201	0.0028265704
C	-3.3532369830	4.4636372904	0.0034944975
O	-3.4750626253	5.6921680380	0.0040821060
C	-1.9979278067	2.3561905718	0.0032132358
C	-2.0378954500	3.7881001652	0.0033880134
C	-0.8286591226	4.5143758542	0.0034565817
C	-0.7326006214	6.0147286403	0.0035670989
C	-0.6337861177	6.7046162259	1.2290381121
C	-0.7581155302	5.9793819312	2.5499754031
C	-0.4404266074	8.0908820731	1.2084909866
C	-0.3427332312	8.7843762131	0.0037519614
C	-0.4405014574	8.0910609350	-1.2010727371
C	-0.6338686165	6.7047868856	-1.2218049687
C	-0.7582951806	5.9797669605	-2.5428529265
C	0.4113357439	3.8399363113	0.0033525313
C	0.6441094919	-0.3000720701	0.0030857860
C	0.7364788395	-1.7072105910	0.0029617028
C	-0.4624306646	-2.4600103192	0.0030190395
C	-0.4113266674	-3.8399502392	0.0029427816
C	0.8286687906	-4.5143892159	0.0028224107
C	0.7326106176	-6.0147428222	0.0027465845
C	0.6338880743	-6.7047840809	1.2281384526
C	0.7583116962	-5.9797086452	2.5491553424
C	0.4405287095	-8.0910477056	1.2074344843
C	0.3427378435	-8.7843902362	0.0026167749
C	0.4404111427	-8.0909233489	-1.2021287684
C	0.6337824400	-6.7046478178	-1.2227045330
C	0.7581023061	-5.9794688750	-2.5436745959
C	2.0379047370	-3.7881124579	0.0027209210
C	3.3532475665	-4.4636474876	0.0026271321
O	3.4750747381	-5.6921780394	0.0030185645

C	1.9979354851	-2.3562030074	0.0027358575
C	5.6910621105	-1.4545773277	0.0012891203
C	9.4454657633	-2.0355140149	-0.0007351241
C	9.5291449786	-3.4665917153	-0.0005760280
C	8.2793076304	-4.2526393300	-0.0004820943
O	8.2606797989	-5.4880519001	-0.0014732343
H	11.1738411831	-6.2187659836	-3.3834170440
H	11.6590127027	-4.6820863781	-2.6484259069
H	9.9622924518	-5.1585385919	-2.6469548736
H	11.5934591826	-8.1490286998	-2.1467236375
H	11.8740245992	-9.3615520167	0.0013837512
H	11.6039773205	-8.1450574894	2.1485433932
H	11.1886579333	-6.2135230546	3.3837106950
H	9.9784643190	-5.1491433840	2.6511359451
H	11.6770160522	-4.6798290828	2.6447626247
H	12.9433983374	-3.7935321957	-0.0015326079
H	15.2144998071	0.3314413569	-0.0038944303
H	15.1880135882	2.8048109432	-0.0044419283
H	13.8503477992	5.0037606033	-0.0042624831
H	12.5476841191	5.9398421277	2.6567660837
H	10.7951945702	5.7964438885	2.6422071910
H	11.5550566339	7.2160420260	3.3808924401
H	11.4487179976	9.1849720382	2.1434866869
H	11.3692300775	10.4247207288	-0.0041556961
H	11.4469031315	9.1841205921	-2.1513736975
H	11.5516423825	7.2146563226	-3.3880811431
H	10.7934643194	5.7948923939	-2.6479694863
H	12.5458477154	5.9393665032	-2.6645358233
H	9.5394316014	4.7824880059	-0.0021121777
H	7.2974727673	0.6626690966	-0.0001054495
H	5.8575140076	-5.2988151141	0.0012084479
H	3.9112766910	0.5158686936	0.0026250928
H	-3.9112689068	-0.5158816267	0.0029793027
H	-5.6454344937	0.3747693247	0.0015983066
H	-7.2974731907	-0.6626696628	0.0009754006
H	-15.2144975845	-0.3314231913	-0.0051631919
H	-15.1880171866	-2.8047931522	-0.0054674433
H	-13.8503566894	-5.0037457017	-0.0046772447
H	-10.7926261557	-5.7951139075	-2.6473339656
H	-12.5449988622	-5.9396494542	-2.6644958598
H	-11.5504988835	-7.2149748343	-3.3875725192
H	-11.4462120790	-9.1843226849	-2.1506370828
H	-11.3692587589	-10.4247122619	-0.0032711857
H	-11.4494559882	-9.1847521651	2.1442225025
H	-11.5562099756	-7.2157034055	3.3813993442
H	-12.5485693796	-5.9395548903	2.6568149267
H	-10.7960723693	-5.7961907831	2.6428329113
H	-9.5394407562	-4.7824832486	-0.0012243943
H	-12.9433881772	3.7935436894	-0.0026945419
H	-11.6571687847	4.6814004533	-2.6493600846
H	-9.9605166390	5.1580657774	-2.6471102083

H	-11.1718004641	6.2179699953	-3.3844528396
H	-11.5919972777	8.1485372529	-2.1484913804
H	-11.8740486768	9.3615548213	-0.0008629203
H	-11.6054759504	8.1455568210	2.1467680672
H	-11.6789988262	4.6806094604	2.6438547419
H	-9.9803259644	5.1494950328	2.6509441554
H	-11.1906199107	6.2143603717	3.3826660727
H	-5.8575007782	5.2988082878	0.0023697880
H	-0.0231883888	5.1715825244	2.6475191194
H	-1.7500055117	5.5239818684	2.6594068420
H	-0.6130814192	6.6686550362	3.3871675716
H	-0.3657599509	8.6275950294	2.1513079367
H	-0.1899154171	9.8604469005	0.0038325857
H	-0.3658797286	8.6278975071	-2.1438222805
H	-0.6130792760	6.6691291790	-3.3799388786
H	-1.7502857058	5.5246043925	-2.6523932278
H	-0.0235539571	5.1718118274	-2.6404932177
H	1.3353088496	4.4079650752	0.0033534887
H	-1.3352993350	-4.4079797387	0.0029719198
H	0.0232723343	-5.1720372357	2.6469210539
H	1.7501458601	-5.5241673450	2.6584986733
H	0.6135091161	-6.6691166904	3.3862763684
H	0.3659387837	-8.6278780863	2.1501906054
H	0.1899184853	-9.8604607649	0.0025731231
H	0.3657124774	-8.6276427612	-2.1449390085
H	0.6127168531	-6.6687118698	-3.3808293939
H	1.7501202268	-5.5243834610	-2.6532872409
H	0.0234206390	-5.1714359158	-2.6411154984
H	5.6454362399	-0.3747761606	0.0012494834

Cartesian coordinates for optimized geometry of **6** in solution (CH_2Cl_2).

C	8.8695199792	-5.7883199600	2.7506399878
C	8.6243599575	-6.5406899858	1.4619599954
C	8.0497299725	-7.8168199798	1.4966399941
C	7.7991299562	-8.5234199342	0.3220499982
C	8.1227099655	-7.9536599670	-0.9079499930
C	8.6983499381	-6.6799999605	-0.9834199976
C	9.0206299707	-6.0754099729	-2.3313099877
C	8.9457199626	-5.9741699590	0.2115299989
C	9.6632999736	-4.6541899761	0.1591599992
C	11.0700299501	-4.7393299745	0.2010399990
C	11.8484099352	-3.5965899809	0.1649299993
C	11.2343499510	-2.3222699899	0.0910299995
C	12.0603999653	-1.1806499947	0.0597499997
C	11.5077999450	0.0851699996	-0.0101899999
O	12.3075299550	1.2104699929	-0.0430399998
C	13.6869899483	1.0694499926	-0.0043200000
C	14.2919799410	-0.2094499991	0.0688599997
C	13.4741699567	-1.3608499951	0.1019399994
C	14.0248499104	-2.6228099887	0.1728899993

O	13.2112899599	-3.7402699805	0.2047399991
C	15.4258899090	-2.7830399843	0.2135999988
C	16.2378798966	-1.6644599942	0.1816899993
C	15.7063599149	-0.3393999985	0.1084199992
C	16.4888799179	0.8442499955	0.0724999996
C	15.8867099106	2.0915799909	0.0001500000
C	16.7184499028	3.3398199859	-0.0385799998
C	17.0643099130	3.9885599795	1.1664099948
C	16.6169999114	3.4459299806	2.5060799896
C	17.8381699310	5.1545999736	1.1099099930
C	18.2629698984	5.6737599738	-0.1114999995
C	17.9158899230	5.0279799752	-1.2965299924
C	17.1435299107	3.8596699803	-1.2801599960
C	16.7808999044	3.1785699854	-2.5816499875
C	14.4683499419	2.2043099872	-0.0386099998
C	10.1191999501	0.2314899991	-0.0467899998
C	9.2678199691	-0.8814699972	-0.0159699999
C	7.8103799461	-0.7370299987	-0.0463199998
C	6.9896099561	-1.8944599885	-0.0295099998
C	5.6002699598	-1.7752899903	-0.0575099995
C	4.9563299739	-0.5327499958	-0.0970799994
C	3.4980199836	-0.3890799983	-0.1177199992
C	2.6502999880	-1.5034599930	-0.1208499993
C	1.2619099917	-1.3515799957	-0.1291699996
O	0.4533999978	-2.4719299882	-0.1322299991
C	-0.9120099940	-2.3307399897	-0.1342999996
C	-1.5290799911	-1.0571399972	-0.1320099995
C	-0.7046999965	0.0874599996	-0.1315499995
C	-1.2619199932	1.3515999933	-0.1291999992
O	-0.4533999978	2.4719499858	-0.1321999995
C	-2.6502999880	1.5034799906	-0.1209399997
C	-3.4980299851	0.3890899982	-0.1178899995
C	-4.9563399753	0.5327599973	-0.0972599996
C	-5.6002699598	1.7752999918	-0.0576999995
C	-6.9896099561	1.8944599885	-0.0296599999
C	-7.8103799461	0.7370399948	-0.0464199998
C	-9.2678199691	0.8814699972	-0.0160399999
C	-10.1191999501	-0.2314899991	-0.0468199998
C	-11.5077999450	-0.0851799994	-0.0101899999
O	-12.3075199536	-1.2104799944	-0.0430099998
C	-13.6869899483	-1.0694699955	-0.0042600000
C	-14.2919799410	0.2094299988	0.0689299998
C	-13.4741799581	1.3608399936	0.1019899993
C	-14.0248599118	2.6227999873	0.1729399992
O	-13.2112999084	3.7402599790	0.2047699992
C	-15.4258899090	2.7830199867	0.2136899992
C	-16.2378798966	1.6644399914	0.1818099993
C	-15.7063599149	0.3393799982	0.1085299993
C	-16.4888799179	-0.8442799945	0.0726299994
C	-15.8866999091	-2.0915999885	0.0002800000
C	-16.7184399014	-3.3398399835	-0.0384299998

C	-17.1435499136	-3.8596999793	-1.2799999940
C	-16.7809499116	-3.1785999845	-2.5814999869
C	-17.9158999245	-5.0280099743	-1.2963499927
C	-18.2629498955	-5.6737899781	-0.1112999996
C	-17.8381299252	-5.1546199765	1.1100899926
C	-17.0642699072	-3.9885799824	1.1665799930
C	-16.6169299013	-3.4459399820	2.5062299901
C	-14.4683399405	-2.2043299901	-0.0385299998
C	-12.0603999653	1.1806399933	0.0597599996
C	-11.2343599524	2.3222699899	0.0910099998
C	-11.8484099352	3.5965799848	0.1649099991
C	-11.0700299501	4.7393299745	0.2009999990
C	-9.6633099750	4.6541999775	0.1590699994
C	-8.9457299640	5.9741799604	0.2114099990
C	-8.6984199482	6.6800099620	-0.9835599967
C	-9.0207499351	6.0754099729	-2.3314299892
C	-8.1227799756	7.9536699685	-0.9081199964
C	-7.7991499591	8.5234299356	0.3218799984
C	-8.0496899667	7.8168299813	1.4964799921
C	-8.6243199517	6.5406999872	1.4618199910
C	-8.8694199647	5.7883299615	2.7505099849
C	-9.0239899813	3.3977499833	0.0801199996
C	-7.5556299634	3.2754299837	0.0220199999
O	-6.7962899859	4.2506899787	0.0151199999
C	-9.8205299524	2.2056099903	0.0509199998
C	-7.1655099419	-0.5062599976	-0.0878099996
C	-5.7766599636	-0.6250599962	-0.1107099993
C	-5.2087999755	-2.0059999922	-0.1463199995
O	-5.9663199855	-2.9807899827	-0.1785599992
C	-2.9419099838	-0.9385099979	-0.1296499993
C	-3.7373599821	-2.1305299894	-0.1380399992
C	-3.0964299826	-3.3884499849	-0.1411599994
C	-3.8094699813	-4.7119099748	-0.1441099994
C	-4.0865799818	-5.3494799688	-1.3705199941
C	-3.8063199799	-4.6659599779	-2.6897499848
C	-4.6527799781	-6.6294499902	-1.3522399908
C	-4.9384199766	-7.2708399664	-0.1484099992
C	-4.6591699781	-6.6308299785	1.0574899950
C	-4.0925199801	-5.3508899615	1.0802399953
C	-3.8161399795	-4.6709199770	2.4023599867
C	-1.6882999937	-3.4740899817	-0.1375699993
C	0.7046999965	-0.0874499998	-0.1315099995
C	1.5290799911	1.0571599948	-0.1319099993
C	0.9120099940	2.3307599874	-0.1341999994
C	1.6882999937	3.4740999831	-0.1374099994
C	3.0964299826	3.3884699825	-0.1409699994
C	3.8094699813	4.7119299777	-0.1438099993
C	4.0925299816	5.3507999485	1.0805899931
C	3.8161399795	4.6707199745	2.4026599879
C	4.6591899757	6.6307399655	1.0579499929
C	4.9384499756	7.2708499678	-0.1478899995

C	4.6527999757	6.6295599532	-1.3517799930
C	4.0865899780	5.3495899848	-1.3701599949
C	3.8063099837	4.6661899794	-2.6894499890
C	3.7373599821	2.1305399908	-0.1378399993
C	5.2088099716	2.0060199899	-0.1460599994
O	5.9663199855	2.9807799865	-0.1789899991
C	2.9419099838	0.9385199941	-0.1294899994
C	5.7766599636	0.6250699976	-0.1105399996
C	7.1655099419	0.5062699974	-0.0876999995
C	9.8205299524	-2.2056099903	0.0509799997
C	9.0239799799	-3.3977399819	0.0802199998
C	7.5556199619	-3.2754199822	0.0221799999
O	6.7962799844	-4.2506799773	0.0153400000
H	8.5956499772	-6.3994399945	3.6158799825
H	8.2770399473	-4.8660299738	2.7909699852
H	9.9204899395	-5.4968999547	2.8630999873
H	7.7990799489	-8.2571499753	2.4588899876
H	7.3553899428	-9.5146899740	0.3650299981
H	7.9286199365	-8.5009799630	-1.8274099924
H	8.7934999690	-6.7789099542	-3.1379499831
H	10.0776199568	-5.7964299712	-2.4152599882
H	8.4352599638	-5.1643599751	-2.5059699895
H	11.5516299469	-5.7093099928	0.2618999987
H	15.8478399396	-3.7811399820	0.2692499986
H	17.3162699148	-1.7907999891	0.2128499992
H	17.5722299141	0.7674999959	0.1021599996
H	15.5244199005	3.3827899849	2.5734099893
H	16.9682499090	4.0855499807	3.3212599844
H	17.0009499384	2.4339199884	2.6808599876
H	18.1072099209	5.6571099950	2.0358799886
H	18.8624098805	6.5800099691	-0.1398299993
H	18.2451799132	5.4319299584	-2.2508599863
H	17.1775699226	3.7358599817	-3.4354999825
H	15.6947099199	3.0964899860	-2.7069199860
H	17.1800299087	2.1584999894	-2.6291799858
H	13.9983099271	3.1807099834	-0.0952499993
H	9.7198299639	1.2370199936	-0.0996999997
H	5.0431999743	-2.7040499881	-0.0425799998
H	3.0498999863	-2.5100199891	-0.1158799993
H	-3.0498999863	2.5100299852	-0.1159499994
H	-5.0432099757	2.7040699857	-0.0427899998
H	-9.7198299639	-1.2370199936	-0.0997499995
H	-15.8478499410	3.7811199791	0.2693499989
H	-17.3162699148	1.7907799915	0.2129999992
H	-17.5722199127	-0.7675299949	0.1023299993
H	-17.1800799159	-2.1585299884	-2.6290199891
H	-17.1776399327	-3.7358999822	-3.4353399805
H	-15.6947599272	-3.0965299865	-2.7067999845
H	-18.2452099175	-5.4319699642	-2.2506599891
H	-18.8623899306	-6.5800399734	-0.1396199996
H	-18.1071499122	-5.6571299979	2.0360699897

H	-16.9681498945	-4.0855599822	3.3214199811
H	-17.0008799282	-2.4339399860	2.6810099882
H	-15.5243399419	-3.3827899849	2.5735299855
H	-13.9982999257	-3.1807299863	-0.0951699994
H	-11.5516399483	5.7092999913	0.2618599987
H	-8.4353999841	5.1643499737	-2.5060999872
H	-8.7936399363	6.7788999528	-3.1380799861
H	-10.0777499756	5.7964399726	-2.4153399893
H	-7.9287299525	8.5009799630	-1.8275799906
H	-7.3554099457	9.5146999754	0.3648299982
H	-7.7989999373	8.2571599767	2.4587099880
H	-8.5955199584	6.3994499430	3.6157399833
H	-8.2769399328	4.8660399753	2.7908299861
H	-9.9203899250	5.4969099562	2.8630199863
H	-7.7236299684	-1.4343899951	-0.0998899997
H	-2.7536299872	-4.3753899793	-2.7876799846
H	-4.4015999783	-3.7506199829	-2.7947399865
H	-4.0527899797	-5.3233699950	-3.5288499801
H	-4.8696399784	-7.1245799898	-2.2958399908
H	-5.3751799891	-8.2661099509	-0.1500999991
H	-4.8806499759	-7.1271299890	1.9993899921
H	-4.0675999776	-5.3299599975	3.2386799866
H	-4.4087699802	-3.7539799830	2.5085299901
H	-2.7629999852	-4.3836399790	2.5051299895
H	-1.2058799945	-4.4454999797	-0.1380499991
H	1.2058799945	4.4455199773	-0.1378799993
H	2.7630199881	4.3833499793	2.5053599858
H	4.0674999790	5.3297399656	3.2390299844
H	4.4088399797	3.7538399838	2.5088199898
H	4.8806699735	7.1269499629	1.9998999919
H	5.3752199949	8.2661099509	-0.1494999995
H	4.8696599760	7.1247599630	-2.2953399872
H	4.0528699807	5.3236399812	-3.5284899808
H	2.7536099843	4.3757199795	-2.7874399869
H	4.4015299787	3.7507999825	-2.7944899873
H	7.7236299684	1.4343999913	-0.0997999994

Cartesian coordinates for optimized geometry of **8** in solution (CH_2Cl_2).

C	6.0430899457	-0.0068799999	-2.5483199886
C	6.7706899801	-0.1038099995	-1.2252599932
C	8.1597199844	-0.2830799984	-1.2055399929
C	8.8530499736	-0.3743299980	-0.0001900000
C	8.1594299424	-0.2869599987	1.2052799922
C	6.7703999910	-0.1077499995	1.2252399956
C	6.0424999660	-0.0148999999	2.5484299886
C	6.0754099729	-0.0146399999	0.0000500000
C	4.5865699767	0.1725799994	0.0001800000
C	3.7454799841	-0.9766899959	-0.0010600000
C	2.3753099877	-0.8276399960	-0.0009500000
C	1.7979699910	0.4686299978	0.0003900000

C	0.3925899979	0.5993499956	0.0004900000
C	-0.2078299987	1.8404199887	0.0017900000
O	-1.5853199923	1.9671699900	0.0018800000
C	-2.3753099877	0.8276799965	0.0007600000
C	-1.7979699910	-0.4685799974	-0.0005800000
C	-0.3925799981	-0.5992999989	-0.0007500000
C	0.2078399991	-1.8403699920	-0.0020200000
O	1.5853299937	-1.9671199881	-0.0021700000
C	-0.5894899954	-3.0047299860	-0.0032000000
C	-1.9685399928	-2.8928699836	-0.0030500000
C	-2.6251399877	-1.6255099905	-0.0017300000
C	-4.0343399782	-1.4436299954	-0.0014900000
C	-4.5865599752	-0.1725199991	-0.0001900000
C	-6.0754099729	0.0146299999	0.0000600000
C	-6.7705399583	0.1073699995	-1.2250899950
C	-6.0427599508	0.0143699999	-2.5483299900
C	-8.1595899656	0.2864399985	-1.2050199916
C	-8.8530799779	0.3740499982	0.0005100000
C	-8.1596199699	0.2831999983	1.2057999935
C	-6.7705599612	0.1040599997	1.2254199952
C	-6.0428099580	0.0076299999	2.5484299886
C	-3.7454799841	0.9767399926	0.0009600000
C	0.5894999969	3.0047799827	0.0030400000
C	1.9685499889	2.8929199855	0.0029600000
C	2.6251499892	1.6255599925	0.0016400000
C	4.0343499797	1.4436799921	0.0015000000
H	6.7395899709	-0.1213399994	-3.3843399820
H	5.5358199859	0.9586299927	-2.6604599893
H	5.2706599762	-0.7791899967	-2.6432799866
H	8.6984099468	-0.3519399981	-2.1476399872
H	9.9309899266	-0.5141199974	-0.0002900000
H	8.6978999787	-0.3588299981	2.1472799879
H	6.7387899608	-0.1320999993	3.3842499848
H	5.2699199748	-0.7873699968	2.6407899856
H	5.5353699736	0.9503399978	2.6634899892
H	4.1788199773	-1.9716699913	-0.0021100000
H	-0.1081499993	-3.9775899825	-0.0042100000
H	-2.5749999869	-3.7941999802	-0.0039600000
H	-4.6830099776	-2.3155299868	-0.0023600000
H	-5.2704499722	0.7870799971	-2.6409799867
H	-6.7391699630	0.1311499992	-3.3841099857
H	-5.5353299678	-0.9507299961	-2.6631799866
H	-8.6981799664	0.3579799982	-2.1469799868
H	-9.9310399338	0.5136899975	0.0006800000
H	-8.6982099707	0.3522199985	2.1479399883
H	-6.7393999434	0.1211899994	3.3844999840
H	-5.2712399756	0.7807999972	2.6435199844
H	-5.5344499990	-0.9573099972	2.6603599854
H	-4.1787999797	1.9717199879	0.0019900000
H	0.1081599997	3.9776399792	0.0040600000
H	2.5749999869	3.7942499822	0.0039300000

H	4.6830099776	2.3155799888	0.0024500000

HOMO -1 and LUMO +1 profiles at B3LYP/6-31G(d) level of theory (Gaussian16) of molecule **1**-**6** and **8**.

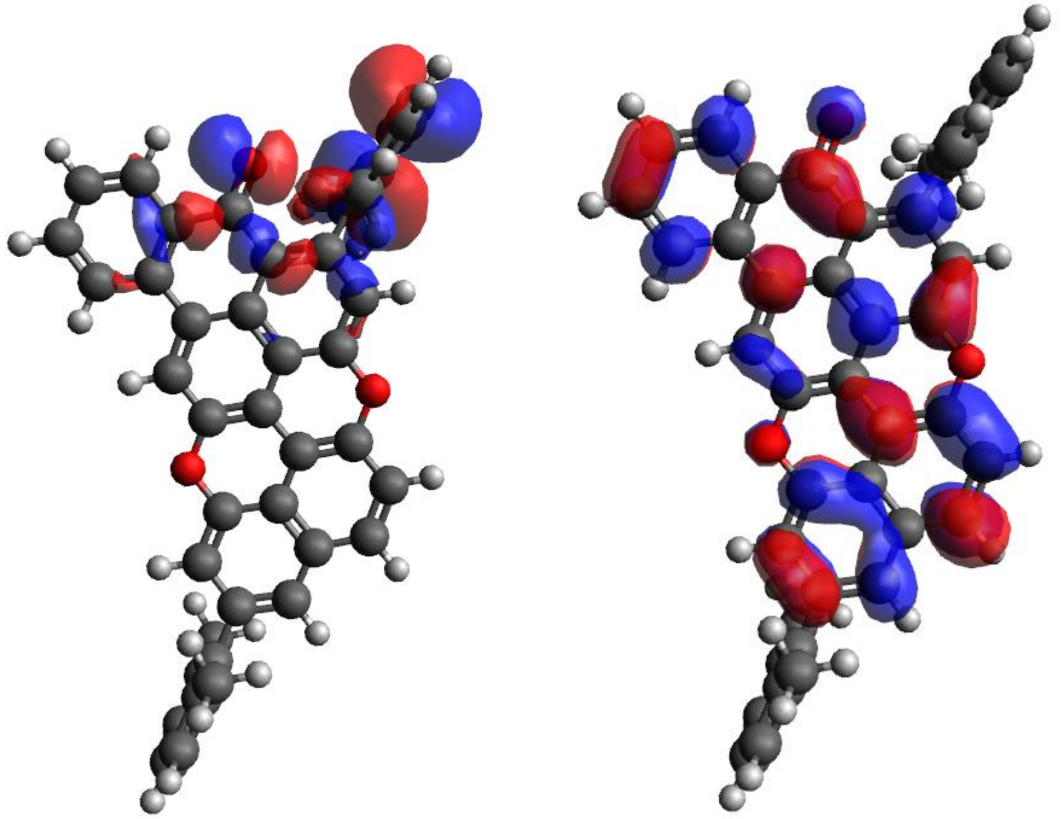

Figure S198. Left, HOMO−1; right LUMO+1, of molecule **1**.

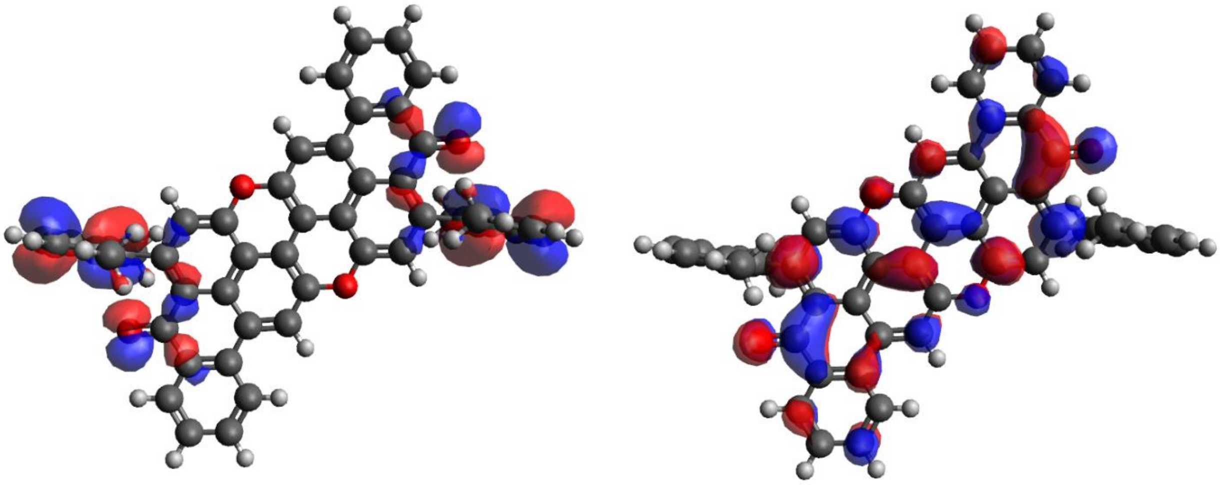

Figure S199. Left, HOMO−1; right LUMO+1, of molecule **2**.

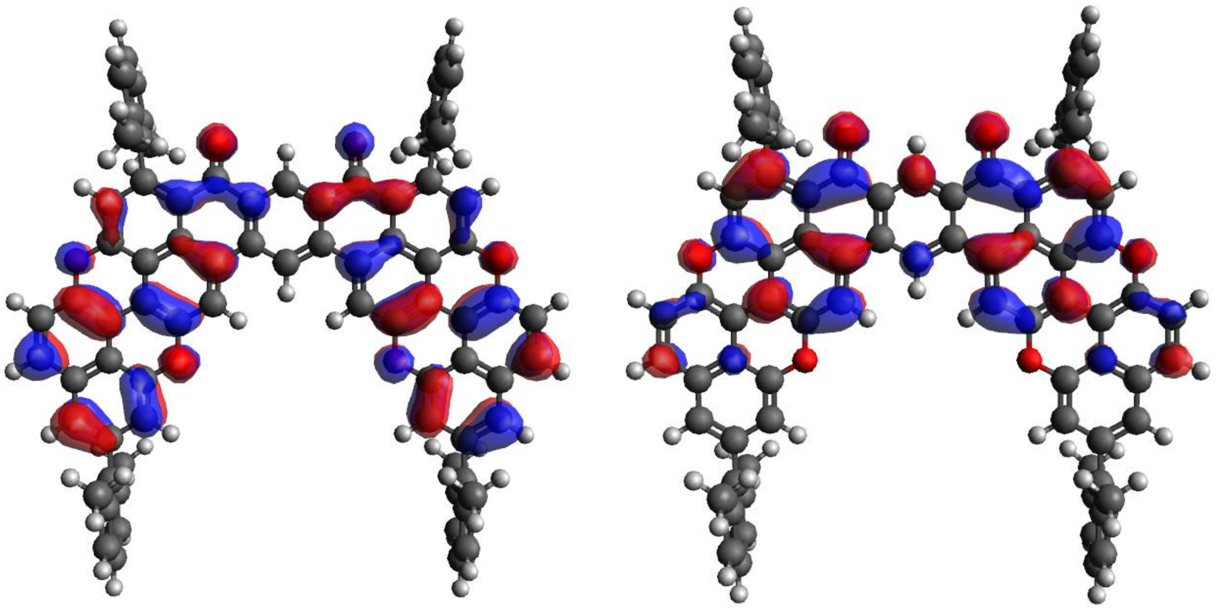

Figure S200. Left, HOMO−1; right LUMO+1, of molecule **3**.

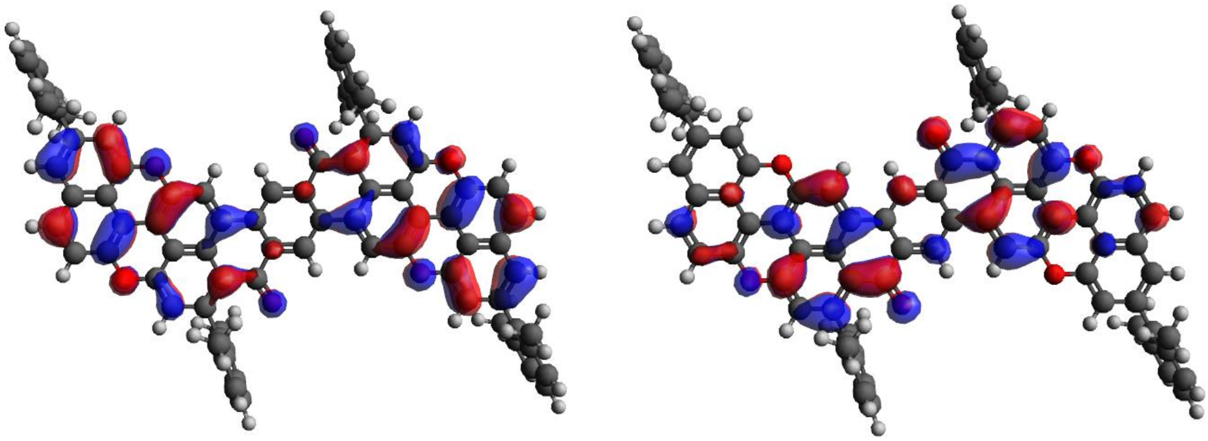

Figure S201. Left, HOMO−1; right LUMO+1, of molecule **4**.

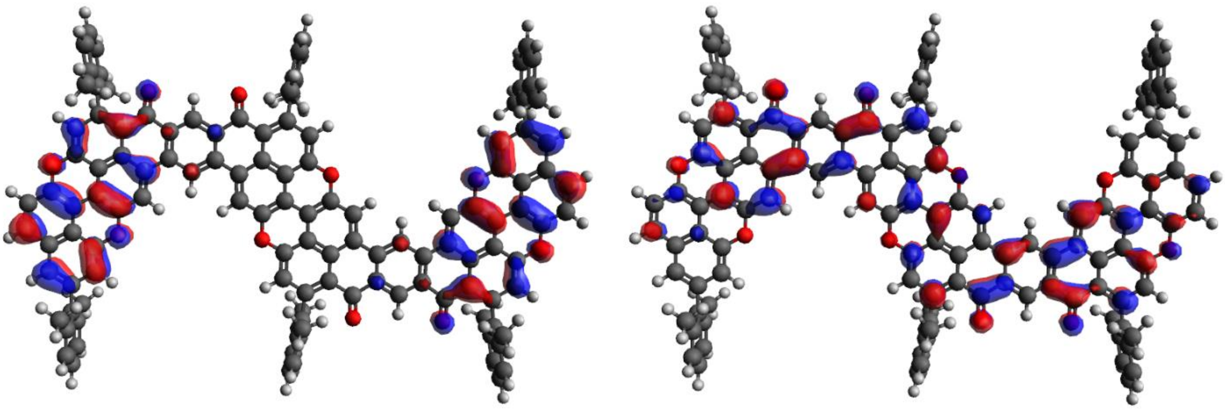

Figure S202. Left, HOMO−1; right LUMO+1, of molecule **5**.

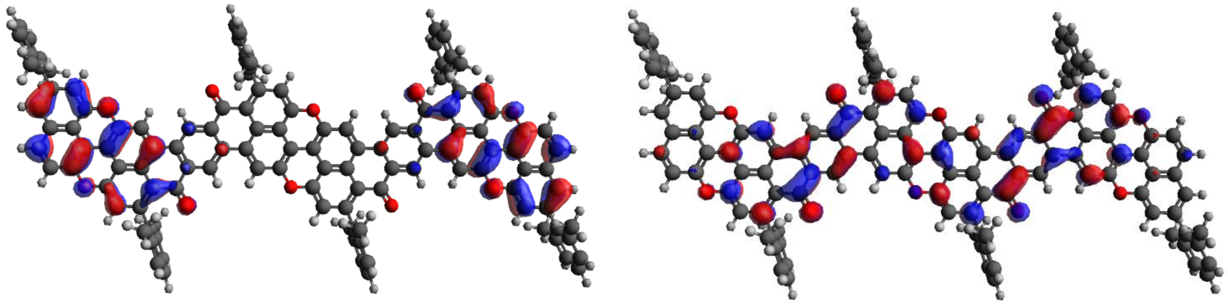

Figure S203. Left, HOMO−1; right LUMO+1, of molecule **6**.

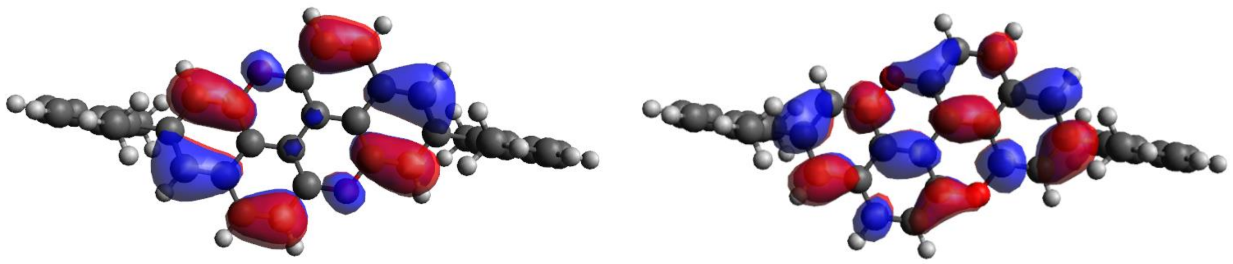

Figure S204. Left, HOMO−1; right LUMO+1, of molecule **8**.

Calculation for the Lewis-acid adducts NIR emitters

In addition to compound **1** and its complex **1·BAr$_3^F$**, excited-state calculations were also performed for compound **2** and its corresponding complexes. Since **2** contains two ketone moieties, it can in principle coordinate up to two **BAr$_3^F$** units. Experimentally, only a single equivalence point was observed during titration, leaving it unclear whether one or both binding sites are occupied upon addition of the boron compound.

We initially hypothesized that coordination at one ketone could shift the electron density away from the second, thereby disfavoring further complexation. However, analysis of the ground-state electron density and atomic charges did not fully support this idea. The calculated binding free energies for the first and second coordination steps are –12.7 and –10.1 kcal/mol, respectively, indicating that the second binding event is only slightly less exergonic. This finding is consistent with the initial hypothesis, yet it suggests that under sufficiently high **BAr$_3^F$** concentration, the doubly complexed species should still form.

No distinct species could be resolved spectroscopically, implying either that only one complex predominates or that both coexist but display nearly identical optical signatures. The computed absorption spectra displayed in Figure S205 support the latter interpretation, showing strong overlap between the singly and doubly complexed forms, particularly for the main S_1 transition. Since both species exhibit nearly identical near-infrared absorption features, the number of coordinated boron centers appears to have little influence on the photophysical properties of the system.

The comparable association energies indicate that the two binding sites behave largely independently, such that formation of the first complex does not significantly alter the electronic properties of the second ketone. Consequently, the equilibrium constants for both complexation steps are likely similar within experimental uncertainty.

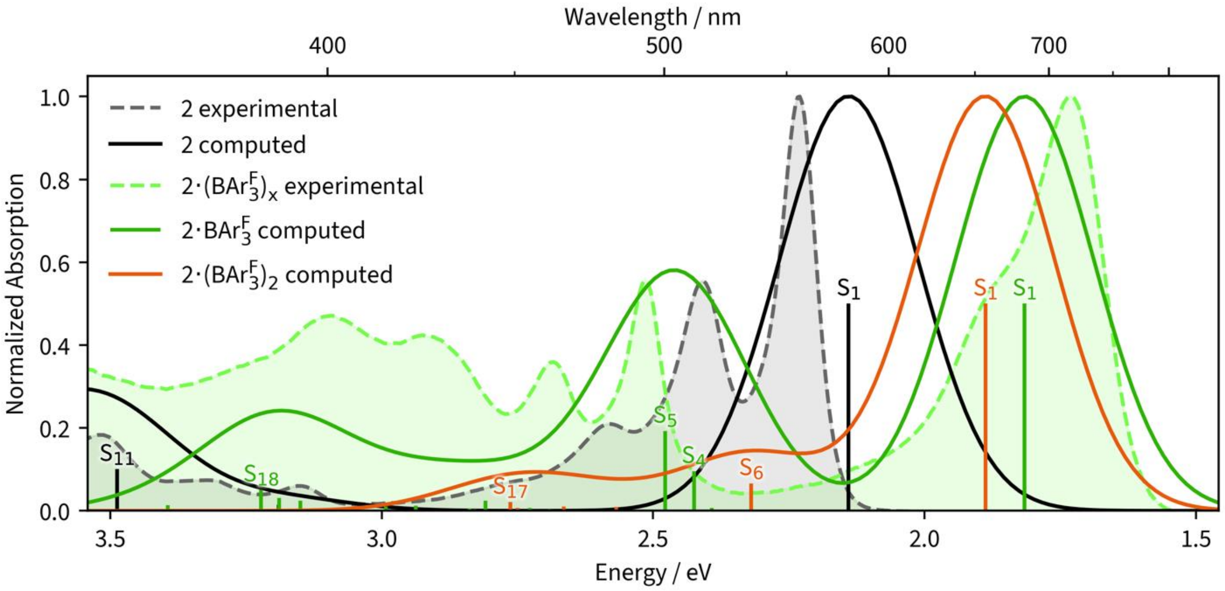

Figure S205. Comparison between the experimental absorption spectra and the computed spectra obtained at the B3LYP/6-311G(d) level of theory with PCM(DCM) implicit solvation for compound 2 and its complexes.

Table S1. Lowest 20 excited singlet states for the different calculated compounds. Excitation energies and corresponding oscillator strengths were obtained at the B3LYP/6-311G(d) level of theory, employing PCM implicit solvation to account for the DCM solvent.

	**1**		**1·BAr$^F_3$**		**2**		**2·BAr$^F_3$**		**2·(BAr$^F_3$)$_2$**	
state	dE(eV)	f_{osc}	dE(eV)	f_{osc}	dE(eV)	f_{osc}	dE(eV)	f_{osc}	dE(eV)	f_{osc}
S_1	2.204	0.545	1.784	0.532	2.140	1.144	1.816	0.865	1.888	1.330
S_2	2.789	0.000	2.464	0.013	2.439	0.000	2.392	0.011	2.174	0.000
S_3	2.857	0.099	2.494	0.122	2.709	0.000	2.410	0.000	2.245	0.006
S_4	3.330	0.031	2.805	0.014	2.713	0.000	2.425	0.165	2.245	0.004
S_5	3.397	0.141	2.819	0.054	3.184	0.000	2.478	0.333	2.295	0.000
S_6	3.536	0.074	2.882	0.061	3.190	0.033	2.691	0.006	2.319	0.176
S_7	3.597	0.023	2.890	0.132	3.198	0.004	2.728	0.011	2.564	0.000
S_8	3.657	0.016	2.937	0.001	3.266	0.000	2.776	0.000	2.568	0.024
S_9	3.713	0.000	2.956	0.015	3.270	0.000	2.808	0.043	2.665	0.028
S_{10}	3.733	0.002	3.009	0.023	3.318	0.000	2.838	0.008	2.666	0.000
S_{11}	3.770	0.100	3.034	0.001	3.488	0.230	2.895	0.005	2.669	0.004
S_{12}	3.815	0.000	3.044	0.016	3.492	0.000	2.938	0.021	2.671	0.000
S_{13}	3.873	0.000	3.151	0.104	3.583	0.029	2.992	0.019	2.691	0.004
S_{14}	3.966	0.111	3.281	0.010	3.605	0.000	3.066	0.012	2.694	0.000
S_{15}	4.082	0.347	3.472	0.070	3.624	0.000	3.150	0.043	2.740	0.000
S_{16}	4.164	0.037	3.511	0.036	3.656	0.000	3.189	0.054	2.749	0.019
S_{17}	4.171	0.064	3.565	0.111	3.661	0.134	3.196	0.005	2.763	0.056
S_{18}	4.256	0.000	3.653	0.015	3.662	0.000	3.222	0.088	2.792	0.000
S_{19}	4.344	0.094	3.762	0.037	3.778	0.057	3.319	0.001	2.817	0.000
S_{20}	4.414	0.001	3.803	0.002	3.815	0.000	3.394	0.023	2.829	0.011

Table S2. Most important orbital contributions (>5%) to the lowest 20 singlet excited states listed in Table 1 for the different calculated compounds. The orbital indices corresponding to the HOMO and LUMO of the investigated compounds are: **1** (155, 156); **1·BAr$_3^F$** (279, 280); **2** (181, 182); **2·BAr$_3^F$** (305, 306); **2·(BAr$_3^F$)$_2$** (429,430).

	**1**		**1·BAr$_3^F$**		**2**		**2·BAr$_3^F$**		**2·(BAr$_3^F$)$_2$**	
state	trans.	contrib.	trans.	contrib.	trans.	contrib.	trans.	contrib.	trans.	contrib.
S_1	155→156	98.8%	279→280	99.2%	181→182	99.2%	305→306	99.2%	429→430	99.1%
S_2	154→156	89.4%	278→280	97.6%	181→183	97.7%	303→306	97.0%	429→431	96.7%
S_3	155→157	97.4%	277→280	97.0%	180→182	68.6%	304→306	89.4%	428→430	69.0%
					179→183	23.8%	304→307	8.7%	427→430	18.4%
									428→431	10.5%
S_4	153→156	83.9%	275→280	85.4%	179→182	67.7%	301→306	90.8%	427→430	69.0%
	152→156	12.0%			180→183	24.7%	305→307	5.6%	428→430	18.4%
									427→431	10.5%
S_5	152→156	69.4%	279→281	68.3%	178→182	88.4%	305→307	91.4%	426→430	85.4%
	155→158	13.0%	270→280	13.0%	177→183	10.3%	301→306	5.7%	425→431	10.9%
	155→159	8.0%	271→280	9.9%						
	153→156	5.5%								
S_6	155→159	64.6%	273→280	73.7%	181→184	88.9%	302→306	97.3%	425→430	78.5%
	152→156	10.5%	276→280	9.8%	177→182	6.7%			426→431	17.4%
	153→156	5.4%	275→280	6.2%						
	155→158	5.0%								
S_7	155→158	74.7%	271→280	39.3%	177→182	80.4%	300→306	92.2%	424→430	87.4%
	155→159	14.6%	270→280	21.9%	178→183	10.1%			423→431	7.2%
			279→281	20.7%	181→184	7.8%				
			272→280	7.8%						
S_8	149→156	81.7%	276→280	77.9%	180→183	62.9%	304→307	78.2%	423→430	84.1%
			273→280	11.7%	179→182	31.0%	304→306	10.3%	424→431	8.2%
			272→280	6.5%			304→308	5.8%		
S_9	146→156	69.5%	272→280	61.9%	179→183	63.9%	296→306	38.4%	420→430	50.3%
	154→157	12.2%	271→280	23.7%	180→182	30.0%	297→306	26.0%	418→430	32.8%
	154→156	9.0%	276→280	10.3%			298→306	20.6%		
							299→306	7.8%		
S_{10}	151→156	94.7%	270→280	54.3%	176→182	77.8%	299→306	78.1%	422→430	45.1%
			271→280	20.3%	181→185	14.5%	297→306	8.7%	427→431	19.2%
			272→280	11.0%					419→430	13.6%
			269→280	10.0%						
S_{11}	155→160	75.3%	274→280	97.5%	175→182	81.6%	298→306	67.2%	421→430	53.0%
	148→156	8.5%			181→186	6.9%	296→306	21.1%	428→431	21.5%
	145→156	5.8%							422→431	5.6%
S_{12}	150→156	99.3%	269→280	77.7%	181→185	54.6%	297→306	52.0%	422→430	29.8%
			270→280	6.8%	174→182	18.4%	296→306	31.7%	417→430	20.8%
					176→182	15.3%	299→306	6.4%	419→430	18.8%
							294→306	5.8%	416→430	13.1%
S_{13}	154→157	78.3%	268→280	86.9%	178→183	77.2%	295→306	63.5%	428→431	47.0%
	146→156	17.7%			177→182	9.9%	294→306	21.2%	421→430	32.2%
					175→182	6.1%	297→306	5.3%	427→431	8.2%
S_{14}	148→156	55.2%	267→280	92.4%	177→183	83.6%	294→306	53.9%	427→431	49.1%
	153→157	11.6%			178→182	9.8%	295→306	20.7%	417→430	13.1%
	155→160	10.0%					305→308	7.7%	422→430	9.8%
	147→156	7.5%							428→431	8.5%
	152→157	7.0%							419→430	7.5%
S_{15}	147→156	73.8%	266→280	53.7%	174→182	66.0%	305→308	84.9%	419→430	48.4%
	148→156	8.5%	279→282	21.0%	181→185	19.7%			417→430	31.2%
			279→283	18.6%	175→183	6.5%				
S_{16}	145→156	68.1%	265→280	86.3%	171→182	61.2%	293→306	75.4%	420→430	35.3%
	153→157	10.2%			170→183	23.9%			418→430	29.7%
					179→183	7.2%			426→431	16.4%
S_{17}	153→157	51.9%	279→282	44.4%	176→183	69.1%	302→307	90.4%	426→431	56.7%
	148→156	18.1%	266→280	33.1%	173→182	7.5%			418→430	18.8%
	152→157	11.3%	279→283	11.1%	174→183	5.1%			425→430	12.4%
	145→156	6.4%								
S_{18}	152→157	74.8%	279→283	49.5%	170→182	59.7%	292→306	48.2%	425→431	57.2%
	153→157	21.0%	279→282	31.1%	171→183	25.1%	293→306	13.2%	417→430	15.7%
			264→280	9.8%	180→183	7.4%	290→306	13.1%	416→430	9.3%
							291→306	10.0%	426→430	7.9%
S_{19}	155→162	40.0%	264→280	50.8%	181→186	56.2%	303→307	96.6%	416→430	58.8%
	149→157	38.6%	263→280	18.6%	176→183	14.3%			425→431	17.6%
	145→156	10.7%	279→284	10.9%	168→182	12.4%			414→430	5.5%
			279→283	7.2%	174→183	11.8%				
S_{20}	151→157	92.8%	278→281	97.6%	181→187	30.4%	301→307	89.8%	415→430	76.5%
					172→182	28.4%				
					175→183	28.3%				

DFT-Optimized Geometries

The geometries optimized at the B3LYP/6-31G(d) level of theory with PCM(DCM) implicit solvation were used for the TDDFT excited-state calculations. All corresponding atomic coordinates are provided in XYZ format in *optimized_geometries.zip*.

Methods

The molecular structures were initially built using both GaussView[15] and Chemcraft[16]. Subsequently we performed a conformational search using CREST 3.0.2[17] with the completely automated, partially polarizable generic force field GFN-FF[18] in the non-covalent interaction mode and an energy threshold of 7 kcal/mol. The resulting conformers were optimized at the B3LYP-D3BJ/6-31G(d)[19–23] level of theory with PCM[24] implicit solvation to account for the DCM solvent, as implemented in Gaussian16.[25]

For the single and double complexes of compound **2**, the initial conformer search yielded more than 100 structures. Therefore, the most promising candidates were preselected prior to the full optimization by single-point energy calculations at the DFT level of theory. The optimized conformation with the lowest energy was then used for the TDDFT excited-state calculations, which included 20 excited states at the B3LYP-D3BJ/6-311G(d)[26] level of theory with PCM(DCM) solvation. Excited-state analysis and density data generation were performed using Multiwfn 3.7.[27,28]

7. References

[1] A. Lausi, M. Polentarutti, S. Onesti, J. R. Plaisier, E. Busetto, G. Bais, L. Barba, A. Cassetta, G. Campi, D. Lamba, A. Pifferi, S. C. Mande, D. D. Sarma, S. M. Sharma, G. Paolucci. *Eur. Phys. J. Plus* **2015**, *130*, 43.

[2] W. Kabsch. *Acta Crystallogr., Sect. D: Biol. Crystallogr.* **2010**, *66*, 133–144.

[3] O. V. Dolomanov, L. J. Bourhis, R. J. Gildea, J. A. K. Howard, H. Puschmann. *J. Appl. Crystallogr.* **2009**, *42*, 339–341.

[4] G. M. Sheldrick. *Acta Crystallogr. Sect. A: Found. Adv.* **2015**, *71*, 3–8.

[5] G. M. Sheldrick. *Acta Crystallogr. Sect. C. Struct. Chem.*, 2015, **71**, 3–8.

[6] Gaussian 16, Revision A.03, M. J. Frisch, G. W. Trucks, H. B. Schlegel, G. E. Scuseria, M. A. Robb, J. R. Cheeseman, G. Scalmani, V. Barone, G. A. Petersson, H. Nakatsuji, X. Li, M. Caricato, A. V. Marenich, J. Bloino, B. G. Janesko, R. Gomperts, B. Mennucci, H. P. Hratchian, J. V. Ortiz, A. F. Izmaylov, J. L. Sonnenberg, D. Williams-Young, F. Ding, F. Lipparini, F. Egidi, J. Goings, B. Peng, A. Petrone, T. Henderson, D. Ranasinghe, V. G. Zakrzewski, J. Gao, N. Rega, G. Zheng, W. Liang, M. Hada, M. Ehara, K. Toyota, R. Fukuda, J. Hasegawa, M. Ishida, T. Nakajima, Y. Honda, O. Kitao, H. Nakai, T. Vreven, K. Throssell, J. A. Montgomery, Jr., J. E. Peralta, F. Ogliaro, M. J. Bearpark, J. J. Heyd, E. N. Brothers, K. N. Kudin, V. N. Staroverov, T. A. Keith, R. Kobayashi, J. Normand, K. Raghavachari, A. P. Rendell, J. C. Burant, S. S. Iyengar, J. Tomasi, M. Cossi, J. M. Millam, M. Klene, C. Adamo, R. Cammi, J. W. Ochterski, R. L. Martin, K. Morokuma, O. Farkas, J. B. Foresman, and D. J. Fox, Gaussian, Inc., Wallingford CT, **2016**.

[7] C. De Luca, D. Zanetti, T. Battisti, R. R. Ferreira, S. Lopez, A. H. McMillan, S. C. Lesher-Pérez, L. Maggini, D. Bonifazi, *Chem. Eur. J.* **2023**, *29*, e202302129.

[8] P. E. Eaton, G. R. Carlson, J. T. Lee, *J. Org. Chem.* **1973**, *38*, 4071–4073.

[9] M. Noji, M. Nakajima, K. Koga, *Tetrahedron Lett.* **1994**, *35*, 7983–7984.

[10] T. Kamei, M. Uryu, T. Shimada, *Org. Lett.* **2017**, *19*, 2714–2717.

[11] T. Kamei, S. Nishino, A. Yagi, Y. Segawa, T. Shimada, *J. Org. Chem.* **2019**, *84*, 14354–14359.

[12] R. Kuhlman, G. Whiteker, **2013**, US8592615B2.

[13] S. Mattiello, M. Rooney, A. Sanzone, P. Brazzo, M. Sassi, L. Beverina, *Org. Lett.* **2017**, *19*, 654–657.

[14] B. Mu, X. Hao, X. Luo, Z. Yang, H. Lu, W. Tian, *Nat. Commun.* **2024**, *15*, 903.

[15] GaussView, Version 6.1.1, Roy Dennington, Todd Keith, and John Millam, Semichem Inc., Shawnee Mission, KS, **2019**.

[16] G. A. Zhurko and D. A. Zhurko, "ChemCraft, Tool for Treatment of the Chemical Data." http://www.chemcraftprog.com

[17] P. Pracht, F. Bohle, S. Grimme, *Phys. Chem. Chem. Phys.* **2020**, *22*, 7169–7192.

[18] S. Spicher, S. Grimme, *Angew. Chem. Int. Ed.* **2020**, *59*, 15665–15673.

[19] A. D. Becke, *J. Chem. Phys.* **1992**, *96*, 2155–2160.

[20] S. Grimme, S. Ehrlich, L. Goerigk, *J. Comput. Chem.* **2011**, *32*, 1456–1465.

[21] S. Grimme, J. Antony, S. Ehrlich, H. Krieg, *J. Chem. Phys.* **2010**, *132*, 154104.

[22] W. J. Hehre, R. Ditchfield, J. A. Pople, *J. Chem. Phys.* **1972**, *56*, 2257–2261.

[23] P. C. Hariharan, J. A. Pople, *Theoret. Chim. Acta* **1973**, *28*, 213–222.

[24] G. Scalmani, M. J. Frisch, *J. Chem. Phys.* **2010**, *132*, 114110.

[25] Gaussian 16, Revision C.01, M. J. Frisch, G. W. Trucks, H. B. Schlegel, G. E. Scuseria, M. A. Robb, J. R. Cheeseman, G. Scalmani, V. Barone, G. A. Petersson, H. Nakatsuji, X. Li, M. Caricato, A. V. Marenich, J. Bloino, B. G. Janesko, R. Gomperts, B. Mennucci, H. P. Hratchian, J. V. Ortiz, A. F. Izmaylov, J. L. Sonnenberg, D. Williams-Young, F. Ding, F. Lipparini, F. Egidi, J. Goings, B. Peng, A. Petrone, T. Henderson, D. Ranasinghe, V. G. Zakrzewski, J. Gao, N. Rega, G. Zheng, W. Liang, M. Hada, M. Ehara, K. Toyota, R. Fukuda, J. Hasegawa, M. Ishida, T. Nakajima, Y. Honda, O. Kitao, H. Nakai, T. Vreven, K. Throssell, J. A. Montgomery, Jr., J. E. Peralta, F. Ogliaro, M. J. Bearpark, J. J. Heyd, E. N. Brothers, K. N. Kudin, V. N. Staroverov, T. A. Keith, R. Kobayashi, J. Normand, K. Raghavachari, A. P. Rendell, J. C. Burant, S. S. Iyengar, J. Tomasi, M. Cossi, J. M. Millam, M. Klene, C. Adamo, R. Cammi, J. W. Ochterski, R. L. Martin, K. Morokuma, O. Farkas, J. B. Foresman, and D. J. Fox, Gaussian, Inc., Wallingford CT, 2016.

[26] R. Krishnan, J. S. Binkley, R. Seeger, J. A. Pople, *J. Chem. Phys.* **1980**, *72*, 650–654.

[27] T. Lu, F. Chen, *J Comput. Chem.* **2012**, *33*, 580–592.

[28] Z. Liu, T. Lu, Q. Chen, *Carbon* **2020**, *165*, 461–467.
